# Supplementary material for: Physicians’ perspectives on continuity of care for patients involved in the criminal justice system: A qualitative study
Source: PLoS One. 2021 Jul 14;16(7):e0254578. doi: 10.1371/journal.pone.0254578 (PMC8279398; doi:10.1371/journal.pone.0254578)
Supplement: S1 File — (DOCX) [file pone.0254578.s001.docx]

**Appendix E**

**Project**: Identifying and Addressing Disparities in the Criminal Justice and Health Care Systems—A qualitative examination of provider perceptions

**Interview #:**

**Time of interview:**

**Date:**

**Place:**

**Interviewer:**

**Duration of interview:**

**Interview Protocol:**

Thank you for taking the time to participate in today's interview. This interview is part of a larger project with the University of Minnesota and Hennepin County, examining the intersections of health and community supervision. The goal of this interview is to gain an understanding of your perceptions of the criminal justice system, as well as experiences you may have had treating patients with a history of criminal justice system involvement.

I want to begin today by getting a general overview of what you know about the justice system:

**Knowledge of CJS**

1. To start us off, could you tell me what you think of the current state of criminal justice practices in the US?
2. Next, I would like to discuss some criminal justice system terminology. Could you explain to me what comes to mind when you hear the following term: [PROBE each individually]
   1. Prison
   2. Jail
      1. PROBE: What do you know about the similarities and differences between prison and jail?
      2. PROBE: How do you distinguish jail vs. prison?
   3. Probation
   4. Parole
      1. PROBE: What do you know about the similarities and differences between probation and parole?

**Education/Training**

1. During medical school did you receive any training (formal or informal) on working with justice-involved populations (like youth in detention, or patients in jail or prison or on probation)?
   1. PROBES:
      1. If yes, could you describe what that training was?
         1. Was this an experience all [medical students] received?
         2. Is there additional training or education that you think would have been helpful to you?
      2. If no, what training or education would have been helpful to you?
         1. [If no training would have been helpful] Could you explain why that is?
2. ...what about residency?
   1. PROBES:
      1. Any formal training on this or informally through your patient experience during residence?
3. ...what about fellowship?
4. …what about at your current or past places of employment?

**Screening & Ascertainment**

1. During visits, do you ask patients about their current or past involvement with the justice system?
   1. If yes, how do you gather that information? OR if yes, how do you ask questions about current or past justice-involvement?
   2. If yes, how does that information inform your care of that patient?
   3. If no, could you explain why not? Have you thought about asking?
2. What do you think the benefits are of asking your patients about their justice system involvement? Are there any risks or challenges to asking them about their justice system involvement?

**Patient Characteristics**

1. Tell me a bit about your overall patient population.
   1. PROBE:
      1. How would you describe the income levels of your patients?
      2. How would you describe their insurance status?
      3. What about their disability status?
      4. Do you/have you noticed particular challenges or barriers faced by patients from particular racial and ethnic minority patient populations? Can you tell me about them? OR Can you give me an example?
2. If you have treated patients with justice system involvement, what was that experience like for you as a provider?
   1. PROBE:
      1. Were they specifically referred to your care?
      2. How do you think justice system involvement may have impacted your patients' ability to access and receive health care?
      3. How did you approach that patient's treatment plan?
      4. Do you communicate with their parole officer/probation officer/the court system about their treatment?
         1. If so, what information is exchanged?
3. Aside from possible justice system involvement, what else are your justice-involved patients dealing with socially?
   1. What about medically?
      1. PROBE:
         1. What are their physical health needs?
         2. What are their mental health needs?
         3. What are their substance use treatment needs?
4. Are there any resources or services that your patients need that are not available to them? OR is there anything you wish you could offer/suggest/refer them to but can't because it's not available?

**Concluding Questions**

1. Thinking broadly, are there any changes to health care delivery that you would suggest to better meet the needs of patients with a history of justice system involvement?
2. Thank you for your time today, before we wrap up today's interview, is there anything that I did not cover today that you would like to add?
3. Are there other providers that you would suggest we also interview?

**Thank you/Wrap-up**

1. Thank you again for participating in today's interview! We will be interviewing additional physicians from Hennepin Healthcare, NorthPoint Health and Wellness, and Community-University Health Care Center over the next [time frame]. When we have reports or publications available from the study, would you be interested in receiving a copy of those?

**Interviewer debriefing notes:**
