# Supplementary material for: Physicians’ perspectives on continuity of care for patients involved in the criminal justice system: A qualitative study
Source: PLoS One. 2021 Jul 14;16(7):e0254578. doi: 10.1371/journal.pone.0254578 (PMC8279398; doi:10.1371/journal.pone.0254578)
Supplement: S2 File — (ZIP) [file pone.0254578.s002.zip › Clean/Participant_10_Audio1_LJ_deidentified.docx]

I: I just like to put them in different areas of the room-

P: Okay.

I: Just in case one, one position is a little better than the other.

P: Okay. Sound coordinator.

I: (laughs). Okay, so again, thanks for taking the time to speak with me today. This interview is part of a larger project. It's a collaboration between us at [health system], the folks at the [University], as well as [County], um, where we're exploring this intersection between health and being on probation and parole, um, so and the criminal justice system more broadly.

And this interview is designed to assess what you know and your perceptions about the criminal justice system, as well as any experiences you may have had with treating patients that have some type of justice system involvement.

Um, and so I want to begin by getting a general overview of what you know about the criminal justice system. Could you tell me a bit about what you think about the current state of the criminal justice system in the US?

P: (long pause) Uh, so that ... I don't even know how to approach that question. Um, so if somebody is um, thought to violate a law, then they can be arrested and charged with some infraction, and then they spend some time in jail, and then they get a lawyer, they go to court. You know, the court makes some kind of decision, and they could get a sentence, and from there they go, you know, to a jail or workhouse or a prison, depending on the extent or the charge. And they may have a judge trial or they may have a jury trial. I mean the mechanics of it I, I don't feel like I'm an expert on.

I: Mm-hmm (affirmative).

P: What I just said to you is kind of what I know. I don’t know, from paying attention, or something like that. Um, and then I also know just a whole interpretation about what works in that system and what doesn't work in that system. I mean, it's like, all of our systems, people who don't have money and are at a disadvantage, or people who have money can get lawyers or you know, find ways of making it work in their favor.

Uh, the people who don't have money often include, um, people of color, African Americans, Native Americans, um, Hispanic, Asians people. Um, so I mean, I, I would never use the word justice or fair. I would use the word that it follows kind of legal protocols, and, and those come down, you know, harder or heavier on a lot of people then ... It, it isn't distributed fairly or equally. So that, I mean, that's my opinion.

I: Mm-hmm (affirmative).

P: Um, and it's gathered from knowing some people, um, and then also just hearing stories and then what I hear in the press or what I read in the newspapers.

I: So next I'd like to discuss some criminal justice system terminology.

P: Mm-hmm (affirmative).

I: Um, could you explain to me what comes to mind when you hear the term prison?

P: So prison, I think of somebody who has a felony charge, um, in either state law or federal law.

I: Okay. And how about the term jail?

P: I think of that as somebody who's been arrested and may be awaiting trial. Or if they get a misdemeanor, they may be assigned to a county or city jail for less than a year, or to a workhouse.

I: And how do you distinguish between jail and prison?

P: How do I distinguish?

I: Mm-hmm (affirmative).

P: Um, so what I just said (laughs). So there's prisons, you know, so Oak Park Heights. There are still a lot of prison where people. I mean, those are people that have been convicted and so, um, and they're in there for usually a long time. And then jails, people are not yet guilty. You know, they just got arrested so they can be ... supposedly they're supposed to get a, you know, fast trial. So I see jail as kind of a holding place. Again, for misdemeanors I think of those going more to like workhouses or, um, yeah.

I: And what comes to mind when you hear the term probation?

P: So, probation, to me, is a, um, resolution of the court that they may make that somebody has a crime, um, and then instead of being put into jail or workhouse, they're given probation. Meaning they have to meet with a parole officer and meet certain terms of their probation. Um, and if they violate the probation, then they can go to jail or go back to prison.

I: And then what comes to mind when you hear the term parole?

P: So, parole, to me, is when somebody has, um, has served a certain amount of their time and it's decided that they can spend the rest of their time, um, outside. And so they're paroled or let out. And then they have to meet with their probation officer and the terms of parole.

I: Let's see. And then, switching now to your background in education and training. During medical school, did you ever receive any training, whether it was formal or informal, on working with folks that have justice system involvement?

P: No.

I: Is there anything during that time that you think would've been helpful to you?

P: Um. Huh. Um. I mean, so the bigger question is, is ...the bigger question is, are we training healthcare providers and doctors to work, um, w-w-where they're needed within that in the community, not just hospitals and clinics. So, um, so I would put correctional health as part of the, as I would put school health, or nursing home health, or places where people are, um, and they need healthcare. I believe we should be trained in that. And, because community health, again, and I'm a family medicine doc. And so we emphasize community health a lot. So I think we should all be educated in community health, which would include correctional health. And then the word health, I would apply the same meaning to that as I would apply to school health. It isn't just taking care of disease, but is providing prevention services and education services.

I: And thinking back to your time during residency, is there any training on working with criminal justice involved folks that you received then?

P: When I was a resident?

I: Mm-hmm (affirmative).

P: No.

I: Do you think that there would have been a place for that then?

P: So I mean it's the same speech.

I: Uh-huh (affirmative).

P: I mean, it's the idea that ... So, um, so in my mind, the healthcare system, um, it really belongs to, you know, the public. Or belongs to our citizenry. Or belongs to our public institutions. It shouldn't just belong to healthcare in hospitals and clinics. So yeah, I mean, I would like to have been trained in all of those different areas of care.

I: And did you complete a fellowship as part of your training?

P: Uh ... no.

I: Okay. And so, now, moving forward to your current place of employment or any past places of employment, were there any types of training that were provided to you then?

P: Well sort of like on-the-job training, because, um, because when I was, um, I worked at [county hospital] before it was [new name]. And so, our department, family medicine, ran the correctional clinics at the jails, [county jail], and the workhouse. And sometimes went out to the prisons. So we ran it as a faculty and then we brought our residents with us, for the reasons I just mentioned.

I: Mm-hmm (affirmative). So could you tell me more about the, what types of the on-the-job. Was there any formal, was it more informal? [crosstalk 00:09:06]

P: No, there wasn't any formal. Um, and it was, um. Yeah. I mean it's so. Um. So it was sort of like, uh, transposing what I knew about health and disease from a clinic setting into a jail or into a workhouse. So it was trying to take the same kind of medical paradigm or knowledge and just bring it inside.

What was missing, um, was everything we're talking about now. What, what does it mean to people when they're in the criminal justice system? How did they get there? Um. If they do their time and then they're released, is their life any different? Or, you know, how do I, um ... So the kind of work that I believe, um, family medicine does is more along the lines of healing and recovery. Um. It is illness treatment, yes, but as the illness, you're dealing with illness, you're always thinking about how will somebody recover or return to their full life and full functioning.

That was missing. I mean, I will say, that was missing in my approach. I didn't think about it a lot and I didn't have any, therefore, any kind of conceptual or practical structure to provide the care to the people in, um, corrections. Nor did I provide that kind of, um, curriculum other than the most informal conversations. They weren't bad informal conversations, but it was just based on what I knew and what the resident knew and what we were trying to do.

I: Mm-hmm (affirmative).

P: So I think it was good intentioned, but it wasn't really well-structured didactics.

I: Okay. And so, during your day-to-day visits with your patients, do you ever ask them if they have any current or past justice system involvement?

P: I don't. Um. It comes up a fair amount. Um. And I, um ... So it's one of those things that I think of as a privacy issue. Um. So when people want to disclose to me, you know, I pay attention to it. Um. But I also ... So to me, um. So to me, I view the correctional system, um, as some form of trauma. It's either, you know, overt trauma that's not pretty at all. Or it's kind of covert trauma. You know, being put in bars, you know, having our freedoms taken away, that's traumatic.

So it's like, any of the traumatic events in my patient's life, um, I'm careful about how I enter that area. And also if they're immigrants, um, I don't ask, you know, "How did you get here?" Um. "Was it an easy trip or a hard trip," do you mean. So I, um, because I don't want to re-traumatize. And then I also don't feel, there's a lot of traumatic events that I don't have remedies for. So again, if somebody self-discloses, um, I will then ask health-related questions.

I: Mm-hmm (affirmative).

P: But I don't feel like I'm really dealing with the experience.

I: So what types of health-related questions do you then ask those folks?

P: Oh. Injuries, infections, um, medications, testing, kind of the ... I mean then, when I shift to those gears I can do the same job I would do if somebody told me they just came from, you know, whatever, Florida. So I'd ask well, you know, what care did you get in Florida?

I: Mm-hmm (affirmative).

P: So once they reveal that they've been in some kind of correctional institution, I feel okay in asking the medical-type questions.

I: Are there any benefits that you see to either asking or like knowing this information about your patients?

P: Well the benefit to knowing ... So I think it is good that we know as much as we can. Um. The challenge and it isn't only with correction but as I just mentioned, um, yeah. So I mean, yeah. So, so, how do we find out what we can find out about our patients in a respectful way that doesn't re-traumatize them? And also, says I'm gonna do something with this, do you know what I mean? So this is important. And um, that, that's a hard thing to do in a—

I mean, so is sexual assault. Whether in prison or outside of prison, that's a hard thing to know. It's a very important thing in people's lives, but I don't, I mean I don't ask that. Um. I do ask, "Do you feel safe now?" So I try to, in a current way, find out if something dangerous is going on. But I don't ask, "Have you been a victim of sexual assault?" It, unless I think it might be pertinent to what's going on.

So I feel kind of the same way about somebody having an experience in the correctional system, you know. So again, there's likely to be underlying trauma. Doesn't have to be or it doesn't have to be big trauma. But I would, um, I'm reluctant to go there unless I know what good it's going to bring to the patient and to me.

I: And can you tell me a bit more about your overall patient population that you see on a day-to-day basis?

P: Here?

I: Mm-hmm (affirmative).

P: Well, my per ... So I, I would say half of my patients are Somali men that are over 50 or 60. So I just, I try and fit a lot of patients like that.

I: Mm-hmm (affirmative).

P: So I have a lot of patients whom, by the way, I don't know their involvement in the correctional system or penal system or something, you know, in Somalia, either, do you know what I mean. And there's all of this stuff of the war trauma. So, so that's a big thing with those people. Um. Then the rest of my patients, I would say, are a mix of many outpost patients. You know. Race, some African Americans, some native, some mostly Mexican or Hispanic, some Hmong. Uh, the ages that go from kids, I see some kids, teenagers, young adults. Um. And, and most of them have some kind of economic disadvantage. That's why they're here. They don't have insurance and we're a sliding fee. But it doesn't mean, um, it doesn't mean that they're necessarily in trouble. I mean, they, they all are struggling, at least with the economic part of it.

I: Mm-hmm (affirmative).

P: And then they might have had all the other disadvantages that go with it. I mean, they may have lesser education. They may have had, oh, lesser employment opportunities. You know, so all those things that happen to people that have been discriminated against by race or economics. They usually have some of that.

I: Okay. And then you mentioned a little bit about the sliding fee. Could you tell me a bit more to help me understand the, like, the income levels or the, perhaps the insurance status of the majority of your patients here?

P: I can give you the aggregate.

I: Mm-hmm (affirmative).

P: So about, um, about 60% of our patients have some kind of public assistance like Medicaid and/or MinnesotaCare.

I: Mm-hmm (affirmative).

P: About 20% have no insurance, so they would be eligible for the sliding fee.

I: Okay.

P: About 10% have private insurance. So, um, the sliding fee, people, um, who, who aren't on a public program or maybe they're applying or something. So then we look at what their income is. We ask them to help us with some kind of, um, verification of their income. But or whatever or however it happens, then they get put into, I think we have four tiers. Um. And so it, um, at the minimum we asked them to pay $5. Um. Some of the sliding fee it's $20. And then we pretty much do everything we can for them. And we try to get reimbursement from any of the insurance for public or private.

I: Mm-hmm (affirmative).

P: So it could be a $5 obstacle or it could be a $20 obstacle. But it's kind of in that range.

I: Okay. It, could you tell me a bit more about the disability status of your patients?

P: Ability or disability?

I: Yes, both.

P: Well, depending on how you, um, so ones that have verified or are already substantiated like, SSI or SDI. So I would say probably 20 or 30% of our patients have some form of disability. Um. Often mental health disability. Um. Did I mention that about a third of our visits to our clinic ... We have both dental, medical, and mental.

I: Mm-hmm (affirmative).

P: So about a third of our visits here are mental health, about a third are medical, and a third are dental. So, so we, we have a pretty large, um, seriously and persistent mentally ill population, so they get that designation.

I: Mm-hmm (affirmative).

P: So along with that often comes some kind of disability rating. So yeah, disability ... The reason I hesitate is that even for our patients who don't get a disability rating, they've had things that have disabled them or have taken away their abilities to function fully, so.

I: Mm-hmm (affirmative).

P: So to me that goes along with trauma. I mean, some people recover and heal pretty well from their trauma and they might not be disabled, but many people have some kind of residual trouble or problems that makes it harder for them to, to function.

I: So now thinking about your patients that you've treated, who have disclosed to you that they have some type of justice system involvement, could you tell me more about that experience for you as a provider?

P: So, so, where I sit today, because I worked in corrections for a number of years, um, so in the beginning, the charge sheet would be in the chart so I could read the charges. Um, and after about a year I quit doing that, 'cause I felt that it, um, it prejudiced me or I couldn't, it would bias me. Um, and that wasn't good, or, and it wasn't easy. So I quit reading the charges. So I hold that same kind of opinion now so when somebody says that they're out after, you know, two years or five years or 10 years, so I don't ask, "Why were you there?"

I: Mm-hmm (affirmative).

P: Um. So, my attitude is, um, sort of like what, wherever their life was before that, now they have, trying to get back to that life or something better. But they also have now, three years or ten years or, of incarceration. So I view it as another, um, risk factor or burden or something.

I: Mm-hmm (affirmative).

P: Um. And that's not easy. You know, it's not easy. Um. As people, as patients have more burdens, it gets harder, um, to figure out how am I gonna put all this together? How can I help? You know? What resources do they have? So, by harder I mean the, um, stakes feel higher. You know? I have to do everything really well or this person could fall off the cliff again. So it's sort of like working in the ICU, I don't know. You know, people are real sick and they're on the edge. So, our people are kind of in the social ICU.

I: Are there any barriers that you see justice-involved patients facing in terms of accessing care?

P: Well, again, I mean if, um, so like I said a lot of people are public insurance. So, if they've been able to get public insurance, either while they were getting out or soon after their getting out, that helps a lot. Um. You know, just getting medications, I mean ... So the, the thing about medical assistance is that it's pretty good paying for everything. So you can pay for your clinic, you can pay for your lab tests. You can go to the hospital and pay for x-rays and you can get your medications. So if somebody has medical assistance, I love that. I mean, that really helps me.

Um. So, yeah, I would say the biggest barrier is getting the resources and, to me, that kind of means medical assistance or it's, you know, they're not gonna have a job when they get out, so they're not gonna have employee-based insurance. So it kinda means, yeah, getting medical assistance. And the sooner the better, and then we can get the prescriptions refilled or we can get the new tests ordered, et cetera.

I: And do yourself ever interact with probation or parole officers or the courts at all?

P: No. Not, not here, I don't.

I: Okay.

P: Um. And then I'll say, if I think really hard, maybe I've been here 14 years, 15 years, maybe a half a dozen times I've talked to parole officers.

I: Mm-hmm (affirmative). And what types, what type of information are you sharing or discussing [crosstalk 00:23:48]?

P: Well they, they come in together so I, I view the patient as my patient. You know? Whoever they wanna bring in, it's up to them. So it's often, you know, "I wanna get a urine test," "I wanna get a U-Tox test," or, "My parole officer wants me to." I say, "Fine, do you want to?" You know, so it's something where, if the person is involved, from my perspective, it's at the invitation of the patient.

I: Mm-hmm (affirmative). And then, aside from possible justice system involvement, what else are these patients dealing with socially, that you're seeing?

P: Oh my gosh. Um. So we, we ask about living situations. I mean, we, we try to do that for most of our patients. And um, and it always surprises me about how they're, um, in a naïve way, I would think that they would be able to go back to some family or some friends or their husband or wife or something. And that doesn't seem to happen easily. So people get out and they're looking for a place to live or they're, and they end up in one of the homeless shelters, or it doesn't feel smooth or connected. It feels a little bit ominous. You know, like, they don't have housing.

Um. And then in the whole idea about the job search. Um. So a lot of times people come out with um physical, um, disabilities or inabilities or, you know, bad backs, bad shoulders, bad everything. And, and now they have to find a job, and how is that going to work? So even the idea about job hunting, um, is again, not clear how that's going to happen.

So it, it, it's ... And then the idea about staying clean. I mean, if they stayed clean in prison, then they get out and, "So are you back in the old neighborhood?" "Yeah." "Are you seeing any of your old friends?" "Yeah." I mean, so how, what can happen to be sure or to help them stay clean? That's always kind of lurking in my mind.

So it, it's, it's all of those things that could be or should be better or in place so that bad things don't happen and doesn't feel often like there's been a real good path for, you know, for housing, for job, for more education, for staying clean, staying in recovery.

I: And then what are you seeing them dealing with medically?

P: Um. Anything or everything. You know, high blood pressure, diabetes, hepatitis C, um, STDs, some HIV, some uh tuberculosis, some, I mean, the whole range of medical problems that I see from my other patients. Um, that's where they came from (laughs). You know, and they went into jail or prison and it, it wasn't better there, do you know what I mean?

I: Mm-hmm (affirmative).

P: And now they're coming out, so, so it's all that stuff.

I: And are there any mental health needs that you're seeing?

P: So um, so even in the area of, um, mental health, I, I have no idea what diagnosis were put, I, they can tell me, but I don't have the records. Usually I don't know what diagnosis was put on them. And then I don't know, was that an accurate diagnosis? Um, so, so one of the things that I would like is that they, whenever they land and feel like, you know, they've got their feet on the ground, to have some kind of mental health evaluation. Um. You know, just to see how they're doing.

I: Mm-hmm (affirmative). And are you referring to mental health diagnoses that occur in other clinics in general or within prison or jail specifically?

P: I would say in general.

I: Mm-hmm (affirmative).

P: But, but I think all of that gets magnified, um, in a jail setting. Um. Not to mention, I think a lot of people get arrested because whatever happened, it, you know, could've been totally exacerbated by a breakdown in their mental health stability or capacity or stuff. Um. How much insight they have into that, I don't know either. Do you know what I mean, like ... So it's kind of a big, black box and yet, um, and yet I think it's there. Do you know what I mean?

I: Mm-hmm (affirmative). Are there any substances use needs, that you're seeing?

P: Well, so, um ... So that's part of our, remember when I said, um, that I don't, um, I'm careful when I'm going into people's lives around areas of trauma or abuse? Um. I, I feel a real important area to go into people's lives (laughs) all the time, is around chemicals. You know, "What chemicals are you using?" So I ask the question. How they answer me is up to them. Do you know what I mean?

I: Mm-hmm (affirmative).

P: So I do ask the question, you know, "Are you using any drugs?" And depending on how they answer, I might ask specifically. You know? "Are you using heroin? Are you using cocaine? Are you using?" Um. And depending on how they answer me, um ... It's, I would say, the first interview that I would have with somebody after they've gotten out of prison, it's not an easy interview, usually. I think, um, they're a little suspicious of me or, they don't know who this doctor is. And I don't really have any rapport with them. I'm just another white coat, white guy, um, asking them questions, you know? So it's not an easy first interview.

I: Mm-hmm (affirmative).

P: And then people where we do establish rapport and come back, I feel we get to a more helpful thing. "How can I help you? What do you need? No I can't do that. I can do this." So, I think that can happen okay. It's not easy to get there.

I: And, in terms of any resources or services that your patients need, are there any that they need but that aren't available to them?

P: So it's connected to what you asked about mental health. So, so, so, our clinic can make a connection of a case manager. When somebody has a mental health diagnosis, okay? I believe that the, the service that would be most helpful, I’m talking to me, is that if I knew, um, people who were just getting out, if they had a case man or call 'em whatever you want, navigator, case man, not parole officer because they have, they're court or they're legal requirements. I'm talking about somebody on the health side. So it would really be great if people had six months worth of a case manager when they get out. Who would help them find the right pharmacy, find housing. Um, are they understanding their medications? Do they need more tests?

So that, so good case man ... or call them community health workers, um, we all want that for all of our patients 'cause it's so helpful, but especially when somebody's, you know, coming out back into the world again. It's, it would be especially great.

I: Mm-hmm (affirmative). So somewhat related to that last question, but thinking broadly, are there any other changes to healthcare delivery that you would suggest to better meet the needs of folks that have some type of justice system involvement?

P: So, can I say anything?

I: Mm-hmm (affirmative). (laughs) Go for it. Yeah.

P: No, but, so I really think that the clinics that should be in all of our correctional institutions should be connected to public health clinics, and not, they're mainly [inaudible 00:32:41] clinics, not correctional clinics. Especially I would say in the jail when, as we mentioned, people are not yet convicted, it means so they're, they're waiting, um, so it, so I believe that, um, if, if, the city or the county ran clinics in jails, um, that would be really good because then they would have the connections already to the health establishment outside of the jail. So there'd be a good connection including being able to share medical records. Including being able to think, not just beyond when is this person going to trial, but what are their healthcare needs beyond that? Do you know what I mean?

I: Mm-hmm (affirmative).

P: So to see it as part of a continuum of care. Um. And to really try to find how it is there and natural primary care clinic for this person. Have they been going to [community clinic] for a long time? Have they been going to [community health care clinic]? So to try to keep that connection, even while they're in jail or then when they get out, to reopen that connection. Along with, as I mentioned, case management services. That could do all that case managing.

I: Mm-hmm (affirmative).

P: And that ... So even something like pharmacy services, you know? Yep, it would be good if the case manager and the client could learn how to go to the pharmacy and do all of that running around. That's good to know how to do it. But in those first couple of weeks or months maybe, maybe a lot of that stuff is brought to the person. Um, 'cause they're busy with housing, they're busy reuniting with their families, or something, or something.

You know, so you could, um, the same way that the best healthcare for seniors is not dragging them all to our clinics every month. And trying to figure out some way, how do we meet seniors where they're at. Kinda like in their home or in their nursing home. How do we meet people that have been incarcerated where they're at?

I: So thank you again for your time today. Um. Before I wrap up today's interview, is there anything that I didn't bring up that you'd like to add?

P: So I don't, um ... So, I know why we're talking about it this way, you know, because what I mentioned that incarceration is a big deal. But in some ways, that isn't the way I would like to define people. I mean I know we're talking about it because they've been in jail or prison, but, but how do we get beyond that, and not, and take that label off? Or not make that the first thing, but just say, "Oh, we'd like to offer you the best healthcare we've got, how do we do that?" So, so, some way trying to offer special, it's sort of like with people who are handicapped. They don't want to be known as their handicap. (laughs) You know what I mean? But they do want to get the best services they deserve.

I: Mm-hmm (affirmative).

P: So how do we imagine getting the best services to people, who have been incarcerated, without making their incarceration the point. So that, I don't know where you're going with your study, or I don't know where they're going (laughs), but that would be my hope about where we go. Um. So it's, I don't know. I mean, if you're asking, um.

So the way the #metoo movement has fought back against, "We were violated, we were abused, and we're not taking that." I mean, "We're not sitting here being victims anymore, we're kind of demanding that we get beyond that." So in some ways, the same way that people who have been traumatized by the corrections system, um, the better that they could find some way to reclaim their dignity. To reclaim their, you know, sense of real justice. You know, he, "Yeah, maybe I did something, I did my time, now, you know, here I am." You know? "I'm me."

So that idea about how do you get true, um, healing and recovery? Um. Which I believe kinda gets directed by the people themselves, so the #metoo movement could never have been started by white coats. And justice for the criminal justice system couldn't be started by, um, medical establishment. But we can certainly support it. You know what I mean, like if there were, if there were a justice movement to bring justice back to those who have been through the criminal system, that would be a great thing.

I: Well thank you again-

P: Sure.

I: ... for sharing your thoughts today. Um. Are there any other providers that you know of that you'd suggest that we'd reach out to, to interview as well?
