# Supplementary material for: Physicians’ perspectives on continuity of care for patients involved in the criminal justice system: A qualitative study
Source: PLoS One. 2021 Jul 14;16(7):e0254578. doi: 10.1371/journal.pone.0254578 (PMC8279398; doi:10.1371/journal.pone.0254578)
Supplement: S2 File — (ZIP) [file pone.0254578.s002.zip › Clean/Participant_11_Audio1_LJ_deidentified.docx]

I: So again, thanks for meeting with me. Um, this project like I've said a little bit about it a joint partnership between [investigator] and I at [health system].

P: Yep.

I: Folks here at the [University].

P: Uh-huh.

I: As well as [County]. And we're looking at the relationship between health and being in probation or parole and the criminal justice system more broadly. Um, and so the question that I'm going to ask you today are designed to assess what you know about the criminal justice system.

P: Uh-huh.

I: And gather information about experiences that you've had with treating patients that have been involved with the justice system. Um, so, to, I want to begin by getting a general overview of what you know about the criminal justice system.

P: Okay.

I: To start us off could you tell me a bit about what you think of the current state of the criminal justice system here in the United States.

P: Um, so I guess I, I think it's problematic. I think that there is you know, just a multitude of examples where uh, the criminal justice system um, first of all, we don't do enough to focus on prevention. Sort of starting from the work I do with adolescents, there's kind of risks for adolescents to disproportionally end up within the criminal justice system primarily based on race.

Um, and um, then once people are within kind of ... I don't know, the tentacles of it, it seems like it becomes harder and harder for people to get out and once um, someone has had that first experience um, particularly if anything ends up on their record, um, just that cascade of employment and um, challenges, housing challenges, all of that transitioning out of jails and prisons and back into um, society becomes harder and harder. So I think that at sort of every step along the way our system is problematic. Yeah.

I: Next I'd like to discuss some criminal justice system terminology. Could you explain to me what comes to mind when you hear the following terms, and I have a few.

P: Okay.

I: Um, and the first is prison.

P: So, prison, um, so, so, um, prison is (laughs) uh, so, what I know about it is that there's federal and there is state prisons and that the um, prisons, people often come from the county and local jail systems and into the prisons for a longer sentencing. Kind of how that plays out and how that transition plays out, I really don't understand or know much about at all but I think of the prison system as being that kind of longer term people who have convictions that are going to be, you know, beyond the kind of smaller, you know, kind of misdemeanor-y, civil sort of, kind of waiting for the next step sort of situation that I think people are in in the jails and so it's convictions and longer term incarcerations.

I: And so you mentioned jail a bit.

P: Mm-hmm (affirmative)-

I: So what comes to mind when you think of jail?

P: So my bit of experience with jails is as a resident, we did rotate through the [County Jail] system and so I had just a tiny bit of exposure. Um, and so I think about those being um, more city or county based. I think about them either being lower level crimes but it, I believe that they're intended to be quite, be time limited but the fact is that I think people end up spending more time in sitting in county jails um, then is probably intended or desirable. Um, for reasons that I don't think that I could really tell you. (laughs) yeah.

I: And so next, what comes to mind when you hear the term probation?

P: So, probation um, from what I understand is um, after someone has come, you know, completed their um, you know, I think about it more associated with prison but maybe it can be associated with jail time as well. Um, that kind of uh, period of time where they're still in contact with the criminal justice system on a very regular basis.

Um, maybe they're getting some um, referral to services but I think about it primarily still being in that kind of punitive kind of monitoring kind of mode rather than uh, helping with the transition out and from like a couple of conversations I've had over the years, I think that what I've heard is that a lot of that depends on the probation officers and how active the probation officers are, kind of individually in trying to support people and connect them with resources to assure that they're kind of making uh, um, kind of a successful transition back into society. So, that's all I know. (laughs)

I: And then finally how about the term parole. What comes to mind when you hear that?

P: Um, I guess I don't know a lot about the difference between probation and parole. Maybe it's parole officers that I'm thinking of rather than probation officers. I'm not, I'm, to tell you the truth I'm sure of the difference. Mm-m.

I: Thank you. And so next I'd like to talk a bit more about your background in education and training.

P: Mm-hmm (affirmative)-

I: Um, thinking back to medical school, did you ever receive any training whether it was formal or informal about working with justice involved populations?

P: No.

I: No. Do you think that there is anything during that time period that would have been useful to you?

P: Um, I, I, I mean I think that you know, we, I think that there needs to be, so yes. I think that we need to do a better job of um, really um, helping people, helping medical students understand how to provide care for a much broader array and type of patient and I think that um, um, that students have a few opportunities and I think students who kind of choose particular tracks have opportunities but I think that generating opportunities for students to think about what, what are the skill sets?

What are the orientations to providing care for folks? For instance, um, uninsured, who are you know, the kind of patients that we see at [health care clinic], or who are you know, um, and what I'm really thinking about is maybe kind of the next thing that you would ask about it is like a residence, is my residency training program, which did offer kind of these very clear opportunities. Um, because it was kind of framed for caring for quote, unquote, "underserved" populations and so we did do rotations in health care for the homeless clinics, we did rotations, we were a regular medical provider in the jail system in the [city name] uh, or [county jail]. And we provided um, you know, a lot of care for homeless youth.

I mean, and so sort of having, giving people a frame for understanding how do you approach health care? How do you do patient orientated care? In, for folks who are socially marginalized um, as opposed to you know, kind of the main stream population that's insured and maybe more middle class? And how do you need to understand that differently and how do you need to think about delivering care differently? So, so I think that, I think that ... kind of setting up those opportunities um, would be, and framing that, those approaches, are really important for med students and I don't think we do enough of them.

I: Mm-hmm (affirmative)-And in terms of your residency, could I ask where did you complete residency and a little bit more about the program that you were in specifically?

P: Yeah, so unfortunately that, that residency program doesn't exist any more. It was the [county name], um, uh, it was out of the, when, when before um, [hospital] was a [health system] hospital it was the [county hospital] and so um, there was a you know, kind of a county residency program, family medicine residency program affiliated with, with that hospital. And um, it was, you know so it was kind of that county hospital model.

I: Mm-hmm (affirmative)-

P: And um, so a lot of the faculty that were really core faculty at that point are now at [health care clinic], or at the [University] or at [county health system] and the program really trained, um, the people who were for a good chunk of time, medical directors at a lot of the local FQ's and it really sort of did produce people who were oriented and understood how to provide systems and care for you know, kind of folks who are socially marginalized.

I: Thank you for that. And did you complete a fellowship at all as part of your training?

P: I did, it was a research fellowship.

I: Okay.

P: Yeah.

I: So not related to the criminal justice system at all?

P: No.

I: Okay.

P: No.

I: And so now thinking about your current place of employment or past places of employment ...

P: Mm-hmm (affirmative)-

I: Have there been any trainings whether it was formal or informal that were provided to you around working with justice involved patients?

P: Mm-m. Nope. (laughs) yeah.

I: Yeah. Okay. Do you think that in your current or maybe in a past place of employment there was a place for that type of training and do you have an opinion on what that might look like ideally?

P: Um, for people who are already out in practice you mean?

I: Mm-hmm (affirmative)-

P: Um, so I guess it would depend on the frame. It would depend on um, I think, I do think having a better understanding um, like particularly for those of us who you know, are working at FQHC's, having a better understanding of um, uh what services are available, what we can connect uh, patients with who are you know, kind of coming out of the um, out of prison. Um, this is kind of thinking more about adults and then I do think um, for those of us providing adolescent care, I think that that's a really important piece. Is having a understanding of that juvenile justice system.

The kind of school to prison pipeline. I think all of those sort of things are really important for adolescent health. Um, so, um, I think that, that if the point is really to sort of think about how do we best support our patients as they come to us that those are the kind of things you know, kind of having a better scope of understanding of what um, patients are experiencing. Um, probably what are the things that we as in terms of social determinants of health that we might be able to support in terms of recidivism, Um, and um, then there's the whole like are we going to provide care within the you know, criminal justice system, I think is a whole other sort of set of topics that is probably for a very specific kind of population of providers who might want to do that kind of work. Yeah.

I: So now, um, focusing on your day to day visits with your patients-

P: Mm-hmm (affirmative)-

I: Do you ever ask them about whether they are currently or have in the past been involved with the justice system?

P: I do. I do because I find that it makes a really big difference in terms of, in terms of um, well, in terms of some of the things I think about in terms of screening. Um, you know, hepatitis, HIV, you know those kind of things. But it also I think is related to asking about kind of substance abuse, it is related to asking about kind of questions about um, um, ... you know, stability in terms of, of employment and housing and those kind of things and whether patients are going to be able to ... be compliant, right, with their medications and those sort of things. Be able to afford their medications, be able to um, you know, be in a stable enough place to be able to take their medications.

Um, so it is something that I, I ask my patients about, new patients about, regularly. And I have a number of patients who you know, kind of see me, end up in jail for a while and are back to, you know, see me again. And so you know, the stories about you know, how and why they get arrested are um, um, you know, sometimes pretty problematic and um, so just kind of asking about mental health needs and trauma and those kind of things too. And trying to connect people with resources that way.

I: Mm-hmm (affirmative)-And when you do bring up this topic with your patients, how do you broach that or begin that conversation with them?

P: Um, you know, I, so, um, like for a new, a brand new patient it's probably not something that I'm going to ... I ask a general question about sort of, um, for new patients that have a number of you know, that are establishing care with me that have kind of a number of chronic problems. So, you know, I often ask in a general sense at that first visit, you know, are there any challenges that you can, you know that you see that might come up with being able to take your medications? Are you, and then I do kind of screen about housing and those sort of things.

And if there's, if something, if anything kind of comes up in that first visit that makes me think maybe you know, anything that you know might generally suggest to me that maybe someone has been arrested for whatever reason in the past, then I might go down that path a little bit more in that first visit. But I kind of um, if not, you know that's something that I might over time kind of try to talk to patients more about as we're talking about, you know, how is treatment going? What are barriers they're experiencing? How can we sort of you know, kind of with a motivational interviewing kind of approach. What are things that have worked for them and how can I support them in doing more of those things that have worked?

Then I might go, go down and ask more specifics but I would say that it's, it's, you know I also don't want to, you know, stigmatize people by making sort or any assumptions about them or have them perceive that I'm making any assumptions about them and so I do, I do think about it as being a question that I ask. It comes up often. People, people talk about it often when they're talking about barriers or challenges. You know, they're like I'm having trouble with a job, I got out of jail, nobody wants to hire, I mean, so I think people, people talk about it often.

Um, but it's not something I would necessarily raise on the first visit if, unless I'm given an opening. But maybe on the second visit or the third visit it would be something that I would sort of say, you know, tell me more about why you think it's been hard to you know, afford your medicines. So and kind of go down that path about employment and what's made that hard. So.

I: Could you speak to whether you see any benefits about asking patients about this?

P: I, I, you know, I think it's such an important social determinant of health. Um, I mean largely from that socioeconomic perspective, um, but sort of secondarily I think, and I got to say I don't explore this as much. But that trauma perspective, I think are kind of the two pieces where I really see it play out. I mean I think that people, people um, you know, kind of, socioeconomic circumstances are all often what sort of, kind of I think make people more prone to even you know being targeted by the criminal justice system in the beginning. And then, and then just recovering from that becomes really challenging and so um, you know, trying to help patients as much as I can around that area whether it's you know, kind of talking to a social worker about housing.

Or whether it's you know, um, or about you know, employment opportunities or that sort of thing. Um, but then, but then the other one is, the other one is, is, is, I think that trauma. And again, I think it's tied in with a whole bunch of other experiences but I, I do think that it, you know, I do think that it, there's a lot of trauma that's related to those experiences, yeah.

I: Mm-hmm (affirmative)-And are there any challenges or risks that you see to bringing this topic up with your patients?

P: Well, I think what I mentioned before. I don't want, I don't want people to, um, have a perception that because of their skin color I'm assuming that they've been involved with the criminal justice system. And so I really um, I really try to create a opening but ... try to make sure that I'm not raising that kind of perception or concern from patients. And so that is the one kind of area that I think is, um, that I'm pretty sensitive to. Um, are there any other risks? Um, ... I mean another potential risk and I've kind of um, I've had a couple patients, is if someone ... kind of um, shares with you some pretty horrible crimes that they've committed and I've had patients share, particularly you know, um, people who are, have been, who are sex offenders.

And um, and so I've had patients, I haven't, I've had, have patients who are you know, registered sex offenders and you know I, I've, I have a strong ethical belief that everyone deserves the best health care that we can provide but you know, um, I think that there are potentially, they're, they're probably providers out there that kind of um, if they know someone has done, committed a crime that’s particularly horrible to them, it may change how they interact with that patient or how they perceive that patient or their empathy for that patient. So I mean I guess, I suppose that's a potential downside as well.

I: And so could you help me understand your general patient population a bit more and just who are the patients that you're seeing on a day to day basis?

P: Mm-hmm (affirmative)-Um, so I have, so at our clinic we have you know, um, I would say probably three quarters of my patients are, you know, seen at our clinic because of our, um, our reputation and our availability of interpreters for doing um, uh, strong work with immigrant refugee population. So I would say that's about three quarters of my patients are immigrants or refugees. And then about a quarter of my patients are kind of underserved, um, sort of ... live in the neighborhood that we're serving.

Um, or are uninsured. Um, and so um, coming to the clinic because they don't have access to health insurance.

I: I see. And so what proportion would you estimate are uninsured versus have other types of insurance?

P: You know right now I think because of the Affordable Care Act, um, most of my patients are insured. I have um, a chunk of undocumented patients that are not insured and then probably um, you know, so I would say maybe an eighth of all my patients are uninsured right now. Um, something like that.

I: Mm-hmm (affirmative)-

P: Just a total guesstimate but, yeah. That, it's very different from pre-ACA.

I: I see. And then how much or how many would you estimate are probably on public insurance versus private insurance?

P: I would say probably 95% of our patients are on public insurance. That might be high but I bet ... eight, for sure more than 80%.

I: Mm-hmm (affirmative)-

P: Mm-hmm (affirmative)-

I: And how would you describe the disability status of many of your patients?

P: Well that's interesting. Um, I think it depends on how you define disability, right. So, um, I have a few, I have a um, so officially disabled, I have uh, my adult patients I have I don't know probably, I have a good chunk that are um, um, legally receiving you know SSI disability. Um, because so many of my patients are you know kind of in that immigrant, refugee category they're, I mean probably not as many as some other um, community clinics but I don't know, again, maybe an eighth. Something like that of my patients I would guess. Yeah.

I: And so you know, you mentioned that many of your patients are refugees and immigrants.

P: Yeah.

I: Um, do you see any barriers that they're facing their maybe different from some of your other patients?

P: Um. barriers to care?

I: Barriers to care, mm-hmm (affirmative)-

P: Um, so, um, so, ... that's kind of, that's kind of hard to answer because we have such a um, a mix of patients at [health care clinic] because we have a large behavioral health clinic so I do have a, a, a pretty, um large chunk of patients who are um, you know, refugees and have kind of some pretty significant behavioral health problems and some significant medical problems. And so I think that that mix makes, can make caring for patients pretty hard. They um, that, both the kind of um, cultural um, sort of barriers to care um that may come from having a different world view about prevention and that kind of thing.

Plus some of the language. Even with we have very good um, interpreters but you know, some of the language barriers plus um, you know, significant, um, mental illness. Um, those people have lots of barriers to care. So that, that group of people um, are, for my undocumented patients it's access for sure. Um, so it kind of, varies depending on who exactly we're talking about, yeah.

I: Okay, thank you.

P: Mm-hmm (affirmative)-

I: And so now thinking specifically about patients that you're serving that have some type of justice system involvement-

P: Mm-hmm (affirmative)-

I: Um, you've talked a little bit about that experience for you already. Could you tell me a bit more about if knowing that information and how you use that information to influence your treatment plan and how you approach care for that patient?

P: Um, so um, I think it comes in sort of two buckets that I sort of mentioned earlier. One is thinking about because someone has been in jail, what do I need to be screening them for? That whole, you know, trying to find out what kind of evaluation and treatment and sort of what happened when they were incarcerated can be really hard to get those medical records out and figure out what actually if a patient isn't absolutely clear about what happened to them in terms of you know, patients with chronic medical problems who got some sort of treatment or evaluation just kind of understanding what happened to them when they were in prison can be really hard.

So that's, that's something I've definitely experienced with patients and then, and then um, just trying to find out what do we need to be thinking about in terms of screening? Um, that's a whole other set. And then I think it's that whole big social determinant of health piece that I um, that again, is kind of that more comprehensive approach to the person and how are you supporting them in order to, as a person? Um, in order to support their, their ability to take care of their health issues. So that is that housing, employment, you know, if they need to apply for disability, what, you know, kind of making sure they're, they have those connections.

Um, I had some very rare cases where I've interacted with and I'm trying to remember what that was. I, I can only think of one where I've actually interacted with a probation officer. Um, and now I'm really having trouble remembering the circumstances because it was five or six years ago but um, I think it was one of those probably stellar probation officers who was really trying to understand what the barrier, how to help this person kind of integrate well, um, back into society.

And so she had reached out to me and I can't remember what it was about but she reached out to me to try to understand something about this person's health and, and whether he was getting what he needed. And um, but that's only happened once. Um, uh, um, but I can imagine how, like having more of a collaboration and that way could be really helpful for um, care delivery and for people transitioning out of, out of prison.

I: Yeah, that interaction with the probation officer is really interesting. Um, if you can recall, could you tell me a bit more about the information that was shared, and what you were able to share given HIPPA regulations and things like that?

P: Yeah, well we went, I remember having a whole HIPPA conversation with the patient and this was something, I can't remember what it was about now. Because I remember having that whole conversation and wanting to make sure that this was all really kind of well-intentioned and it, and the patient was very, you know really felt like this woman was supporting him. And I, I want to say it was something to do with, with diabetes. It was something to do, I want to say it was something to do with diabetes but I can't remember specifically.

I: Mm-hmm (affirmative)-

P: Um, and um, I, I, I do think it was something, I think it was around medication access and I can't remember what the concern was but um, it was, it was somebody very much reaching out to be helpful and seeing how, and trying to problem solve. And so but that's yeah, I guess that's only it. I remember once.

I: Yeah. So that trying to make sure that I'm understanding correctly. The probation officer reached out to you and then you had that conversation with the patient to make sure that it was okay to share that information?

P: Right, right, right.

I: Okay.

P: Yeah.

I: That's interesting.

P: Yeah.

I: Let's see. And then in addition to having a hard time getting a hand on those medical records from a jail or prison facility-

P: Mm-hmm (affirmative)-

I: Are there any additional barriers to care that you see for justice involved folks?

P: Well, you know, um, like again, I don't, I haven't had someone coming right out of the jails recently and so I don't have a strong sense of um, like I have a number of, of long term patients who have been in and out of jail over time but they mostly have maintained their health insurance and so I'm, I'm really not, I'm really not sure what kind of health insurance, if that continues to be a barrier for people. I know it was for a while.

I: Mm-hmm (affirmative)-

P: Um, that was a big issue for a number of people that I saw coming out of jail is that they didn't have, there was no health insurance and they came out with some pretty you know, significant health care needs. Um, and that we were trying to manage, um, um, without health insurance. Um, but I haven't, you know I haven't in the last maybe two or three years I'm not sure that I've had anyone who is like sort of straight out of prison. Um, so I can't speak to how much that's still again, post ACA. How much that's still an issue for people.

Um, and uh, but just in general I think their barriers to care are really around socioeconomics. So, um, it's really about kind of trying to find their way back into society and trying to find um, you know, employment and housing. You know, coming out and I, I can think of a couple of people who you know have um, you know, come, had housing, gone into you know, even like just jail for a short period of time and come out homeless.

So you know, um, and then you know, people's, so, so all of those kind of I think social issues and challenges really make it hard for people to access care and really make their health care a priority.

I: Mm-hmm (affirmative)-

P: Um, because they're just struggling with so much just to kind of get by. So I'd say that's the biggest barrier.

I: Mm-hmm (affirmative)-

P: Yeah.

I: And among your justice involved patients, what are you seeing them doing with medically?

P: I mean, so there's, there's I think like the rest of our society. So there's the um, kind of, specific exposures. You know, there's the Hepatitis C, um, but it's mostly chronic conditions. You know, it's mostly what the rest of my patients are dealing with. Diabetes, hypertension, that's really what I'm, what I'm seeing now.

I: Mm-hmm (affirmative)-In addition to some of those physical health needs are you seeing any mental health or substance use needs?

P: Yeah, so um, mental health for sure. Um, I think that the men that I've cared for and who've been incarcerated, um, mental health as well as substance use is a big problem. Um, you know, sometimes, off, for a good chunk of them, that's what got them into jail in the first place. Um, and but I really find them to be quite resistant to really tackling mental health stuff. Substance abuse, a little bit more. Definitely more open to, to talking about how, you know, more awareness and insight into how that's been a problem for them and um, but mental health.

It tends to be a real hard sell to really get the men, in particular, to be thinking about what role kind of mental health problems and you know trauma or whatever else might have been contributing to the whole picture. Um, is ... that's like a, that's been, that's been a challenge.

I: Mm-hmm (affirmative)-

P: Yeah.

I: And are you seeing the same thing among women? Is it different there?

P: I, I feel like women um, that, so and I, and I have to say, I haven't, I don't, haven't had as many women patients who've been in and out of jail. Some, but not as many. Um, and um, they seen to be a little bit more open. I think like women in general, to kind of um, thinking about mental health as being kind of a, a component of their overall health needs and um, a contributor or sort of part of maybe you know, what's been challenging for them, you know in terms of staying sober and um, getting into jail and all of that. So um, they're, it's a little easier to, to um, at least get them to think about trying, you know, medication or therapy or something.

I: Mm-hmm (affirmative)-And are there any resources or services that you wish you could refer your patients to but you found aren't available?

P: Um, well I think probably like everyone, I just think that our um, the options for housing are just so challenging right now and so um, we have social workers but you know, they're referring people to kind of um, the standard resources um, that a lot of our patients already know about. And men you know, a lot of folks don't want to go into shelters and just finding stable housing is such a big challenge.

Um, so, resources in terms of big R resources, I think that's kind of, that's probably our biggest um, challenge right now. I mean locally and I think nationally too is affordable housing. Um, you know, more, I got to say that the options, the training options, the employment resources options are pretty opaque to me so I've had some patients that have accessed different sort of, you know, training programs but I'm never sure if those are helpful and it's, and I don't know very much about who's eligible for what and how do people get in and what are the kind of things that um, you know that they can, that they can come out with in terms of job possibilities, through some of those things. So that's certainly a resource that um, I don't feel like I have any, I have very little knowledge about in terms of where we can send people for that, um.

Um, and then just, so those are, I think those are the primary things in terms of you know, what I would like to offer patients as kind of, kind of set of resources that might help them with their transition.

I: Mm-hmm (affirmative)-

P: Yeah.

I: So thinking broadly, are there any changes to how we deliver health care that you would suggest to help better meet the needs of patients that have justice system involvement?

P: Well I can certainly come up with like a dream program (laughs) you know, I mean I think, boy I think people need, if we really were to be committed to kind of um, uh, integrating people back into society in a way that is going to really decrease you know their likelihood of offending again, we would need a comprehensive sort of coordinated kind of program um, that would involve like a probation officer, and a primary care provider and a set of resources that people could access. Um, and uh, boy a community health worker that really knew how to work with you know, kind of help coordinate all of that would be kind of ideal.

Socially I'm not sure that we're really, I think, I don't know that we have the will to make sure that, even though I, you know, I can guarantee you something like that would save money over the long term but I, that, that is my guess would be um, whether we have the political will to make something like that happen. But I really think that people probably need that kind of wrap around approach. Yeah, and health care would have to be part of that.

I: Alright. So thank you again for your time today.

P: Mm-hmm (affirmative)-

I: Before I officially wrap up I just wanted to ask, is there anything that I didn't ask you about today that you think is important and you'd like to add?

P: Well, the only thing is um, you know, I think really thinking about adolescents in this and I um, and um, strongly from the prevention perspective and how can primary care providers be involved in sort of some of those prevention pieces for youth. That's a whole other conversation. It's less. I think it's less about um, kind of some that, it's a different set of issues, similar but different set of issues and some of the adult issues, it's really about you know, what's primary care's role and sort of understanding, being part of trying to understand you know, mental health for youth and um, truancy and um, kind of school attendance. How all of those things, you know, again, trauma, how do all of those things fit together?

And who's really um, who's really responsible? Who is the point organization or entity for trying to make sure that kids either don't get involved in, you know, with the juvenile justice system in the first place? Or if they do, that all of those needs are addressed. Substance use, um, getting kids back in school, and I don't, I think that the schools don't have the mental health or health care resources tied in closely but um, primary care doesn't necessarily have the close enough relationships with the schools or the juvenile justice system and so I think kids really fall through the cracks in terms of who's, which entity is really responsible for making sure that kids are doing, that we're addressing their needs from that prevention, primary or secondary prevention perspective. So.

I: Alright. Thank you.

P: Mm-hmm (affirmative)-

I: I'll turn these.
