# Supplementary material for: Physicians’ perspectives on continuity of care for patients involved in the criminal justice system: A qualitative study
Source: PLoS One. 2021 Jul 14;16(7):e0254578. doi: 10.1371/journal.pone.0254578 (PMC8279398; doi:10.1371/journal.pone.0254578)
Supplement: S2 File — (ZIP) [file pone.0254578.s002.zip › Clean/Participant_12_Audio1_LJ_deidentified.docx]

I: So thanks again for your time today. Um, this project is a partnership between us at [health system], The [University] as well as [County]. So we're working together to look, explore this relationship between criminal justice system involvement and health and how those two things interact. And so these, the interview today will include questions that are designed to assess what do you know about the criminal justice system and how any experiences you've had with treating patients with justice system involvement and how that may have impacted them.

And I'd like to begin by getting a general overview of what you know about the criminal justice system. And so to begin, could you tell me about what you think about the state of the criminal justice system in the U.S.?

P: Um, that's a big, okay that's a big question. Um (long pause) so my sources of information for this are, um, so obviously the news media and kind of what's portrayed there. Um, some ah the last few years I've been asking patients a little bit more about their experiences and so hearing from their perspective. Um, ah you know personally my father was a police officer, um and so I have that kind of perspective from growing up. Um, and um and then I actually did a rotation in medical school in the um ah on a prison service basically.

Um so those are like the sources of information and from that, you know, I know that there's a great degree of kind of discrepancy and disparity in terms of who is um incarcerated, the reasons for incarceration, um and, you know, there's I think the system is much, much more human than I think I thought it was when I was much younger and it is therefore much more, uh, I guess biased and unfair in some ways.

Um, and so that's just the perspective I have and actually also I listen to a Serial podcast recently, the Cleveland and that was pretty eye opening. Um, so yeah and I don't know if that answers your question. It's such a broad question.

I: Mm-hmm. Yeah could you tell me more about the podcast you listened to and.

P: Sure, so it was the third season of Serial. Um and they spent a month in a, um, a courthouse in Cleveland and followed multiple cases and um talked to people in all kind of aspects of the criminal justice system. Um, including defendants and lawyers and judges, um and then just the, the kind of the way things played out was really striking to me in terms of how, well how much power a judge has for example, um in terms of things like sentencing and doing um, ah kind of forcing people to do certain things and how much time people are on, um probation I think, parole can't remember the term. Um, so it was that yeah that was the podcast.

I: And so next I'd like to talk about some criminal justice system terminology. Um, could you explain to me what comes to mind when you hear the following terms? I'm going to go through several and the first is prison.

P: So prison is, um, in my understanding is it's the, um, longer term incarceration that happens after somebody has been, um, sentenced. I think.

I: The next term is jail.

P: So that is, ah, I think that is, um ah, the place where someone is incarcerated pending, um, either for either for short term, ah, I guess lesser offenses or pending, um, their actual trial and sentencing. I think.

I: And then when you think about prison and jail, how do you distinguish between the two?

P: Um, um, other than what I just said I think, so I have some and this is in the, I mean I think the, um, the bodies or the entities that oversee the two entities are different and for some reason I think of jail more in terms of, um, relating to the to the Sheriff and the, um, and then prison being more related to kind of corrections officers, um, I don't really think about I mean the condition, like the actual conditions of these places I think of as you know small rooms with bars and limited freedom of movement and freedom of you know decision making on the part of, um, people who are incarcerated.

I don't know how to distinguish them otherwise.

I: Mm-hmm. And then what comes to mind when you hear the term probation?

P: So probation I think of as, um, that the person is still under a kind of supervised, I guess oversight or custody by the criminal justice system and it isn't necessarily that they are kind of housed in prison or jail. Um, but that they need to be regularly that there needs to be regular check-ins and their violation of probation leads to more time in prison. [Laughter]

I: And then my next term is parole.

P: In parole I think is what is if somebody is, um, after they finished their sentence and they are, ah, out of prison or jail then they, or I guess prison, I don't know. Um, that they then, ah, have a period of kind of again just kind of supervised oversight. Um, and I don't actually know what happens to them if they violate. So actually I guess I don't really know the differences very well.

I: Mm-hmm. So you would say you don't really know the difference between probation and parole?

P: Not in any way that made me sound any more intelligent than I just did. So no. [Laughter]

I: Okay. So next I'd like to shift a little bit to your background in education and training.

P: Mm-hmm.

I: Um, during medical school, um, did you receive any training? Whether it was formal or informal on working with justice involved populations? I know you mentioned your rotation and if there were other opportunities that you had we'd love to hear about that.

P: So there's no, there are no, there were no curricular elements at all. Um, it was all, um, just based on the environment that we were working in at the time. Um, and the rotation that I did was a psychiatric, it was my psychiatric rotation, um, and it was in on the, um, the psychiatric service, um, for the, the prison. Um, yeah in all, all my experiences, um, like viewing life on the inside of that was just from that.

I: And was that an experience that all medical students did? Or was it just.

P: No, it was one of many places that people would do a psychiatry rotation and it happens to be that I was, that was where I was, um, assigned to.

I: Mm-hmm and then how about during your residency? Did you receive any training on working with justice involved populations then?

P: No. Not that I can think of.

I: Mm-hmm and did you complete a fellowship as part of your training at all?

P: No.

I: Okay and so now thinking about your, either your current place of employment or past, did you receive any training?

P: Um, well we, we so we have had people come and give talks about different kind of aspects.

I: Mm-hmm.

P: But, um, nothing cohesive and nothing kind of, it's all random basically.

I: Okay. Are there any particular topics that you remember from these talks or what where they focused on?

P: Um, one of them, so ah, there's there were a few by, um, that were by lawyers who were coming in and talking about kind of the process leading up to and actually I didn't even really talk about immigration and I mean I think that's, that's probably considered separate, but maybe not. Um, but anyway so there's, there’s lawyers who were talking about immigration.

Um, I think there was I think we had somebody come in and talking about, um, the kind of the approach of the criminal justice system part of the talk was on, um, commercial sexual exploitation. Um, and that had an aspect of people being, um, you know charged or not charged based on, um, their roles. It was just like little, little pieces of it. There was nothing cohesive and it's only kind of, it's only thinking about it that's makes me even realize that it had anything to do with the criminal justice system.

I: Are there any forms of training that you think would have been helpful to you? Whether that's during med school, residency, or at a current place or past place of employment.

P: Um, yes. Um, I mean I think you know something just even like simple to read that is like a primer or a primer, I don't know, which ever way you pronounce it. I type it. I don’t say it. But just like a basic outline of how things work, um, would be really nice. Including definitions, like the ones I mangled earlier would be really helpful.

Um, and I think we get a lot of kind of the statistics and data on, on like kind of the back end of like this is what the population looks like and this is why they may be incarcerated, but there’s really nothing that I learned about process. Like how does it actually, like what is actually happening and to whom and how is that, how are things, how are things different for different people? Um, but I think just like something to read over would be really helpful. I don't think there needs to be any like fancy online modules. Um, at least I don't think so.

I: And during your day to day visits with your patients, um, do you ask them about their current or past involvement with the justice system?

P: Uh, yes so I have been doing that the last year or so.

I: Uh-huh.

P: Um, and it's not uniform, um, and um, it's usually, um, I usually let it, I usually try to get at it through, through other means rather than asking directly. Um, because I've been in situations where patients have said like why do I need to know that or why am I interested in that? And, um, and so I just I kind of get at it other ways. But if somebody does kind of mention something that I know is related to this, um, ah then I'll ask, you know, have you been, did, usually I just say you know have you been in jail or prison? Um, and how long ago was that? Um, yeah.

I: So, what are some of those indirect ways that you might get at that with your patients?

P: Yeah, so I'll ask, often I'll ask about, um, when I ask about housing situations I'll say like where have they been living? And I'll do a little bit of a housing history, so I usually don’t just say, you know do you have stable housing? I'll ask, you know, where have they been, where are they living now, where were they, how long have they been living there, where have they been living recently.

Um, and that will sometimes get at this and also will sometimes, um ah, in there it serves other purposes too. It gives me a sense of kind of how chaotic their life is and how difficult it might be for them to get to appointments for example. And I'll ask about any kind of lapses in medical care. You know, especially if they have a chronic disease and that's uncontrolled I'll talk about you know have you run out of your medications or did you not have access to your medications?

And often people will say, they're like, “yes when I was, um, in jail I didn't, I didn't have access to my medications” or “when I left like they disappeared, I didn't know where they were and I needed to get them again.” Um, those are kind of the two typical ways.

I: Okay.

P: Sometimes I will, well, actually the other way is if I talk about substance use with a patient, one of the things I'll always ask is, is have they run into any kind of legal difficulties with their use? And part of that is just kind of making the diagnosis of a substance use disorder and part of it is just trying understand to what extent it is affecting their lives.

Um, so that would be another pretty systematic way that I might, that I ask.

I: And so you mentioned using that information to inform whether like how chaotic your patient’s life may be and whether they may be able or not able to make it to appointments. Are there other ways that this information informs your care?

P: Um, yes. So, um, certainly it would for people with chronic diseases it kind of makes it, so there's a couple things. So one is that my patients, my experience is that if they've been involved at all, if they're criminally justice involved, then they're kind of risk for getting, getting to kind of having to go, so violating parole or probation. I can't remember which one. Um, and, you know, going having short stints in jail or then having court dates.

Um, so that they are, there are things that are mandated that are put upon them I guess that, um, that my other patients may not necessarily have. So no, so understanding that, you know, having to drop urine on a regular basis for drug screening. You know that is a burden that that patient has to deal with that my other patients may not necessarily have to.

Um, certainly housing situations become very difficult if somebody has a felony and um kind of working through that and trying to get people housing if they don't have it, um, is a challenge and it's and, you know, it kind of goes without saying that if somebody is homeless it's very difficult to treat pretty much anything until we can get them a place to stay and live and be safe.

Um, I think I talk about especially for my patients who, who have a opioid use disorder. Um, we definitely talk about, um, I think that the risks go up and I know this from just the data, um, the risks of overdose, of relapse go up significantly in that time after they, um, come out of jail or prison. And so, just being aware of that and getting them into clinic for example, or seeing them even if they're really late, um, you know being a little more accommodating and understanding, um, for folks like that.

I think it's just more about providing context on, on the patient's life. Um, to help care for them.

I: In addition to some of those benefits that you already mentioned, are there any other benefits that you see to asking your patients about this and knowing this information?

P: Uh, well it allows me to, I mean from like a, um, mini epidemiologic perspective it allows me to get an understanding of my patient population and where they are. If I don't ask, I will never know. I also, it gives me an opportunity to learn a great deal from patients, um, and so learning about what their experiences are.

Um, those are two like I guess meta benefits of asking, um, yeah and I can't think of other things.

I: Mm-hmm.

P: I'm sure there are.

I: Are there any challenges or risks that you see to asking your patients about this?

P: So there's the distrust piece, I mentioned earlier. Um, and that, um, ah people asking why that would be, um, there is a kind of lack of understanding necessarily of, um, of, of kind of confidentiality and, and, and privacy and that their information is not going to be shared. It's just that kind of piece I think is not well understood and that's particularly true for, um, ah people who are undocumented, um, and getting at kind of that status is very tricky.

Um, there's a lot of distrust and there's a lot of concern, um, and to be honest it's getting, this is getting a little aside, but I mean I talk about this with my colleagues. I wouldn't be surprised if at some point, um, you know medical record requests are done for people without social security numbers for example, by ICE or Homeland Security, and so that just doesn't feel good.

And so, um, that, that makes me, so I'll ask and sometimes I won't actually write anything in the note for example, or I'll kind of peripherally allude to things because I'm not really sure I don't know, I don't know how I get about that.

Um, so it's distrust. I think there's a, um, ah a concern for how they're, how they're going to be treated by kind of the staff more broadly in the clinic. Um, if they, if they say and that they, you know, will be made to wait or they'll be treated differently somehow or um, um or given or not given as much leeway I suppose. Um, I think the other risk frankly is that it’s, it may actually not be terribly relevant to the, to the care at hand and that, um, it might be a distraction and, um, you know, it actually has nothing to do with the reason that they're in the clinic and, um, and I'm asking more for broader history or to you know, I don't think it would be, I don't think I ask to satisfy my curiosity but more for kind of broader history.

And I’ve run into that issue with other patients too that have nothing to do with this and they'll ask like why am I asking them, um, you know a series of questions about their past health for example.

I: Mm-hmm.

P: Because they don't see it as relevant to what they're doing there at that moment. Um, yeah.

I: So could you tell me a bit more about your overall patient population that you see as a provider?

P: So, um, so now I see a lot of patients with opioid use disorder. Um, so that's a sizable portion of my patient panel. Um, otherwise my other patients are typically from around the clinic I work in. Um, it's a community clinic and um, fair proportion of immigrants and refugees, non-English speaking patients, um, ah a fairly diverse group of patients in terms of race and ethnicity as well.

Um, fair, a considerable number of people who are uninsured, it's about a fifth of the patients that I see are uninsured. Um, and then, um, a fair number of patients who are homeless as well. And, and under the, I guess I don't even, this goes to me it goes without saying, but it shouldn't which is that many people are at or under the poverty limit.

I: And would you say in terms of insurance, in addition to those folks that are uninsured, are they, are the others on public insurance? Are there some that are on private?

P: Yes.

I: Do you have a sense of that?

P: So, and I know this just from the data from my clinic, um, so about 51% of patients are on, um, on public, so Medicaid essentially. Um, about 20, 23% are uninsured. Um, and the other are a mix of a variety of other insurances.

I: Mm-hmm and how would you describe the disability status of the patients that you see?

P: Oh, that's a good question. So, there's a considerable number of patients who are disabled on mental health grounds. Um, you know, I didn't even mention the mental health aspects that are just so important. And play such a role here. Um, I guess that would be another piece of the, um, going back to asking what I ask about, patients. Part of that is, um, getting at to what extent is their mental health, um, contributing to their, ah, kind of, being arrested and charged and also, or as a result of, um, this.

Um, I have had patients who've had extended periods of, um, solitary confinement and did some reading on this, um, and it the psychiatric symptoms of solitary confinement are very similar to some of the psychiatric symptoms of other, um, psychotic disease disorders. So schizophrenia, um, so going back to disability, other than mental health, I don't have a lot, I mean, otherwise there's the next group of patients other than folks who are disabled for mental health reasons or would be disabled for, um, often occupationally related injuries.

So like low back pain, um, something to that extent.

I: Mm-hmm.

P: Um, that's kind of the extent that, of patients that I see.

I: And then in terms of patients that are from racial and ethnic minority populations, do you see any unique barriers that they're facing in terms of accessing healthcare?

P: Yes, many. Um, and is this, you're asking about like all comers, like everybody who’s coming to my clinic?

I: Mm-hmm, yeah.

P: Um, yes. I mean anything from just how to, how to like physically access and navigate this, the system that we have and even like the phone trees if you speak another language. It's impossible, essentially. Um, and um to, you know, we know that there's kind of implicit bias involved in all sorts of care and I don't think we systematically do a great job of addressing that. Maybe individuals make attempts at it, but broadly no.

Um, there's explicit biases in our system. Um, in terms of assumptions that are made about people based on what they look like and what that means for their disease processes and you know what their genetics susceptibility is and all that and that's just baked into kind of our medical education systems and that's just, that’s all there.

Um, there's also kind of explicit biases in terms of how we craft policies that we don't really think through. So one, you know, very concrete example is in the hospitals that I work in and actually in the clinic that I work in, there's, there's a sizable kind of security presence there and in fact that the security officers are the first people that you see when you walk into one of our hospitals.

Um, and I'm not sure that that's been thought through in terms of how that might affect, um, people who are not white. So, um, yeah, I mean there's lots of barriers.

I: And then shifting back to patients specifically that you know have had some type of justice system involvement, could you speak more to what that experience like is for you? Or what that experience is like for you?

P: Say that again.

I: So for your justice involved populat-, or patients that you're treating, could you talk more about what that experience is like and how it may differ, if it does, from patients that don't?

P: Right. So, um, for people who, so I've had several people who had, who had extended amounts of time and they were incarcerated for, for decades. And that effects them in kind of every way. I mean in terms of, um, their mental health, in terms of how they interact with me. Um, in terms of their jobs and their homes. I mean it's just, it's, it's a scar that, that is left on people that just, I don't know if it's life long, but I can't imagine it's not.

Um, and so I find myself talking, like that ends up being an aspect of our visits almost every time for in terms of how that is affecting them and how they are able to kind of function in society after being essentially out of society for so long.

For people who were in for lesser periods of time, it is often a, my experience is it's often, ah, there's, it feels like there's always something. There's always something that's kind of coming up or going on and often I end up having, so I end up writing, practically speaking, like a lot of letters.

I write probably more letters for, to somebody. To their parole officer or to the, to somebody, um, about, you know, the medications they're on, or why it is that they are not coming to clinic or why it is that they are not making their other appointments. Um, and how their health affects their ability to do these things and how their health affects work.

So I feel there's kind of more, there's more there that's, um, the vast majority of time is completely legitimate medical issues and, but there's a proportion of the time where I feel like I'm being asked to do something because they want like a get out of whatever free card and they think I can give it to them, um, in that way.

Um, and even in those situations I know I don't like that like my letter doesn't mean anything to in the grand scheme of things. So I will write it in support of my patients. Um, because that's the perspective I come from is they are my patients. Um.

I: And can I ask a follow up about that? Are your patients requesting these letters or is it coming from the probation officers or parole officers at all?

P: So it's the patients are requesting these kind of things, letters, um, and to give to their P.O.'s or sometimes to they'll sometimes say a judge wants a letter, um, I don't actually, I don't actually know who gets the letters. They're always like to whom it may concern types of things.

Um, but there's just, there's just some more of that and, um, for my opioid use disorder patients, I go to kind of great extents to try to point out that the urine toxicology screens that we do in clinic for example, are not something that we should, that they're just, they're medic-, they’re screens for medical purposes and that they really have no role in the legal system.

Um, and yet they will often want us to submit these, um, to, for proof of that they're clean or whatever. Um, and that always makes me feel a little uncomfortable because they’re, the, they’re, they’re often, there’s a lot of false positives on them. They're just not really great tests and they require like a contextualized interpretation of what's going on.

Not just being like, oh it showed cocaine on there. You're in violation and so we are very kind of resistant to that. But, but sometimes the patients will ask for those to be sent, um, to the judge or to the, to kind of show that they're doing what they're supposed to be doing.

I: Mm-hmm.

P: Um, but other than, other than that, other than that so other than the like there's seems to be something hanging over people's heads a lot. And that kind of affects their interaction with healthcare and their interaction with me. I don't kind of feel the influence of their involvement, um, as much and I feel like that's because, that's yeah, I don't feel the influence quite as much.

I: And do you get patients that are specifically referred to your care for certain things? I know that you said you worked with a lot of folks that have opioid use disorder.

P: Mm-hmm.

I: Are those people getting referred to you?

P: So those folks are, yes. To our clinic, I mean not necessarily to me explicitly. Um, I don't have, so there was a period of time, a bunch of years ago, a few years ago where all of a sudden we started seeing people who were had like lengthy, um, ah, prison sentences who had were like very violent crimes, who started showing up to our clinic.

And it turned out that somebody in some aspect of the system was referring them to us.

I: Mm-hmm.

P: Because they thought we had like some kind of special, um, capacity, capability I suppose for caring for these folks. I mean we actually didn't at that, actually we don't I don't think. Um, but that was happening for a period of time. But that has since stopped. So it's only really patients with opioid use disorder that we get specific referrals from.

I: And then you mentioned this, um, patient that had been incarcerated for about a decade and how that interaction was a little different. Could you, tell me a little bit more about that and how that differs from other patients.

P: So, right. So, I mean, he was in for, he was in for, um, 23 years. Um, and, and so um, it was, it was just very interesting to hear him talk about, um, so he would talk since that was a huge part of [inaudible 34:50] he would talk about his experiences there and they were, you know, largely traumatic, but they were oddly also, there were parts of it that he felt very comfortable in and, and felt like that was a place that he understood and a lot of our discussion was like he was now in a world that he didn't understand as well and like how to actually navigate that world.

And even just like social norms and how to interact with other people is something we ended up talking a fair amount about. Um, you know, simple things like accessing his medications and um, ah, like going to the pharmacy and getting his medications and like these were all important because when he, when he and he's not old, so he went in when he was very young and so he had no health problems or anything like that when he was younger.

And then all of the sudden it was like he was put in this time machine and dropped off and was like here fend for yourself. Um, and, and so he was learning all these things that many of my other patients would have had to learn you know decades beforehand.

So, going to the pharmacy, picking up medications, like taking medications, that kind of a thing. Um, the social norms were a piece, um, and he also had significant mental health, um, diagnoses. That, you know, would decompensate and get better and he was, you know, clearly two different people at, when those happened.

And, um, and it was very difficult to, to unpack what of this was underlying mental health disorders. What of this was from his prolonged solitary confinement? And then, and then he was, he, I felt like he was very honest with me about kind of the things he had done before.

Both before going to jail and then prison. And then aft-, and then during, um, but at the same time it was also, it was clear that he wasn't kind of telling me everything that had happened to him or that he had done. Um, and so that, there's just, there’s just a lot of unpacking to do and a lot of things to talk about.

And physically he was relatively healthy. Um, I think he just had like high blood pressure. Um, but most of his medications were psychiatric. Um, and then we had to get him into psychiatry, and you know that's a long wait. And so in the mean time I was caring for pretty advanced psychiatric illnesses that I know, I can do that.

But I'm not like the ideal person to be doing that. Um, and managing him for many months until we were able to get a psychiatrist to kind of take over the management. Um, and during that time he had several episodes of decompensation that related to him not taking his medications or the, or his medications being changed by others. Um, so it was pretty challenging.

Notetaker: Sorry to interrupt, it’s 11:40, just so you know.

I: Thank you. So aside from justice system involvement, um, what else are you seeing your justice involved patients dealing with socially?

P: Um, so, I mean, the big thing is housing and just it is, it's just very difficult to, for people to nail down housing. I know I've come back to that several times, but it's just it's so core and so difficult and yet just very difficult to actually care for someone or for have them care for themselves for that matter without adequate housing, um, so that was one piece.

Um, I think I am surprised by the amount of kind of what I perceive as hoops that people have to jump through. Um, that you know that they're having to jump through all these hoops. They're, you know, meeting with lawyers and um, having court appointments and then having appointments with me and then, and then they're trying to and everybody, I mean virtually everybody just kind of wants to do the right thing. They're wanting to get a house and get a job and, you know, support themselves and their family and doing it is just very difficult.

So even just like getting a job and, and the lack of skills. Both how to do that in a formal way and also the difficulty in terms of doing that with any kind of criminal record layered on top of that the difficulty with doing that with all these other appointments that they have to do.

Um, it just seems like a very, um, it's a big hill to climb. Um, most of the patients that I see now have, again, they are largely have a opioid use disorder. Typically, they don't have a whole lot in the way of other medical conditions and so we are able to, from our part, we're able to limit to the degree where we're asking them to come back and kind of the burden that we're putting on to people.

But I have a few people who, um, who do have significant medical issues and it's very challenging balancing all these things. Um, and it's not, kind of, life organization and having like a little, like to do list. I mean that is not, that's not taught anywhere. You kind of learn that, I don't know. I guess you learn that in high school and college or just figuring things out.

But like it is, it's you know that balancing a checkbook, I mean that is more important than, um, yeah, I mean, that's just really, that stuff is really important, that I feel like people don't have a good handle on. Just like really basic stuff, like how to get from, how to get through a day with your calendar and actually get to places on time. It's challenging.

I: Could you tell me a bit more about the medical conditions your justice involved patients are dealing with?

P: Um (long pause) so I mean again it's mostly opioid use disorder for me. Um, and so the, practically what that means is the, um, the importance of, of continuous buprenorphine treatment and the kind of the bad things that happen when people when that's interrupted by, um, going to jail for example.

The, um, hepatitis C is another piece that we see a lot of. Um, and that is more just, it's just a waiting game in terms of you know, maintaining, kind of getting enough sobriety under the belt in order to qualify for treatment. Um, it just happens to be prevalent in this population, but I'm not sure that kind of being criminal justice involved alters that in any way.

Perhaps it makes the wait longer, I don't know. Um, I have a couple people who, um, have diabetes. And that ends up being, um, sometimes difficult to manage but partly because of the lack of, um, access to medications at times during their life. But more because of the chaos of their lives and it's just difficult to manage diabetes when you don't ready access to food and, or a refrigerator to store your insulin or, or um, or a house. That kind of thing. I keep coming back to that.

I: Are there any mental health conditions that you're seeing?

P: Um, yes. So, um, probably a little bit mor-, I mean there's kind of depression, anxiety, kind of all around just like as a baseline, that is so prevalent. And I can't actually tell if it's more prevalent in my patients who are justice involved. Um, but I feel like, um, the psychotic disorders like schizophrenia and bipolar disorder do feel like they're more prevalent and more and I think because when those symptoms are unmasked, they are, um, they are the types of symptoms that in society are more likely to get you in trouble with the legal system than necessarily depression or anxiety would.

Um, and so I think it's an unfortunate combination of, of that kind of fact along with them being, and well especially schizophrenia being a little more harder to manage the symptoms. Bipolar disorder's actually is something that is often quite manageable and when well managed, there are no issues. Um, that's not always true for schizophrenia.

I: And are there any resources or services that your patients need but aren't available to them?

P: Um, huh. Um, I feel like everybody should have, I mean should have some kind of like care manager or life coach, kind of just assigned to them or in, in a way that is, it is not for the purposes of you know catching them or getting them in trouble or anything like that but just, just to help navigate all these different things and to, and to perhaps either provide them a skill set that they don't have or to just help them to do it them, just like don't even give them the skill set.

Just kind of help them do it. Get a job, get a house, um I think that would be helpful. Some of our, some of my patients have care management, some don't. I'm, my understanding of like who gets it and how they get it and under what circumstances is pretty confusing. But also there's a lot of people who like they're just, they're, young healthy. I mean they're almost always men, but they're young healthy men who don't have medical issues that would necessarily qualify them for care management.

But they could certainly use somebody who, would be considered I guess a social worker or that might be just like a life navigator, like I said. Um, so that kind of very broadly speaking. Um, I think clear, I think the what happens to people's insurance and their medications is very unclear. Both to me and I think to my patients when they're in the different stages of the criminal justice system.

Um, and so clarity around that would be useful. Um, assistance in housing, I sound like a broken record but that would be really important. That's really important. Um, and then assistance with, um, I guess related to that, but assistance with, um, getting people into an environment conducive to living their lives, but also sobriety. And, you know, putting people back into the situation from which they came is a great way of having people do the things that got them in trouble in the first place.

And, and that's just not a great system. So some way of kind of moving them out of that for a period of time. But specifically around sobriety.

I: And now thinking broadly, are there any changes to, additional changes to healthcare delivery that you would suggest to better meet the needs of folks that have criminal justice system involvement?

P: So I think more training in the, so I think training, um, around this patient population both. I mentioned at the beginning kind of just like for me, just reading, but for, I guess for, I can always learn more. But specifically for medical students and residents, having the opportunity to, um, to kind of go to court see what's that's like.

I did go to an immigrant court a couple of weeks ago as a court observer. Um, which was just an eye opening experience and really kind of fascinating and it was only an hour and a half. But I was able to kind of see some of the imaginations of this system that is otherwise kind of a black box to me.

So having, you know, seeing people, seeing the system from that perspective. Seeing the, you know, understanding and seeing what it's like in the different stages of, you know, in jail and in prison and juvenile detention and just kind of understanding the lived context of that. And then some kind of, like what happens after that? Just and understanding of what happens down the road and I think that's all part of medical education.

Um, and I think that, you know, old crusty doctors can learn that as well in terms of, I mean, you don't have to be a med student to, to participate in these things. But there just needs to be some mechanism for doing it. Um, again the immigrant court thing was a really cool example of being able to do that. Um, what was the, can, what was the question again?

I: Are there any changes to healthcare delivery that you would suggest [crosstalk 00:48:37]

P: So that's the medical education part and then the healthcare and then the healthcare delivery and certainly being able, having kind of un, like just, you know, health is either a human right or it's not. And if it is, then it doesn't matter what your kind of legal status is. And so, having access to medications, treatment, you know, while people are serving time, would be really important.

Um, and then um, and then interestingly a better, I think a, I think and this sounds weird, but um I think having clinics and, and, and groups who care for folks have a better understanding of who can vote or not. Um, and just you know the fact that in Minnesota you can vote if you are no longer on probation or parole. I can't remember.

But basically that even with a felony people can vote and just like that simple piece is often misunderstood. Um yeah.

I: So thanks again for your time. Before I wrap up is there anything that I didn't touch on today that you'd like to add?

P: (long pause) I don't think so.

I: Okay, thank you.
