# Supplementary material for: Physicians’ perspectives on continuity of care for patients involved in the criminal justice system: A qualitative study
Source: PLoS One. 2021 Jul 14;16(7):e0254578. doi: 10.1371/journal.pone.0254578 (PMC8279398; doi:10.1371/journal.pone.0254578)
Supplement: S2 File — (ZIP) [file pone.0254578.s002.zip › Clean/Participant_13_Audio1_LH_deidentified.docx]

I: All right, so thanks again for taking the time to meet with me today. Um, like I said, the, these questions are designed to get a sense of what you know about the criminal justice system and your experiences with working with patients who have some type of involvement with the justice system, um, and I'd like to begin by getting a general overview of what you know about the criminal justice system, so to start us off could you tell me a bit about what you think of the current state of criminal justice involvement here in the United States?

P: For sure. Um, I, I think big picture there are, uh, a, there's a dichotomy of who is incar- incarce- incarcerated versus who isn't. Um, I think the, uh, uh, uh, there's not a level playing field, uh, with who has the resources to fight arrests, et cetera, so I definitely think there is bias within the system, um, I guess to answer that first question.

I: Mm-hmm (affirmative).

P: Uh, do you want me to tell you more about what I think I know?

I: Sure, go ahead. Yeah.

P: Okay. Um, I don't know a lot. I think, you know, someone goes to “jail.” I know that means different things. Does it mean, um, you know, short term kind of jail, does it mean something a little bit longer term like a workhouse, does it mean prison? Um, I know that when it comes to healthcare and medications, uh, you know, county jails are run by the county and have certain regulations, et cetera, while prisons, uh, and workhouse may be federal I think. Um, that may be the extent that I know without more prompting.

I: Mm-hmm (affirmative). Yeah. Um, could you tell me a bit more about maybe some of the differences that you know in terms of distinguishing jail versus prison?

P: Um, so jail, I think of that as something short term. Uh, how short, short term I don't exactly know. I think it has to do with, um, the type of charge and, uh, maybe even, uh, when a court appearance is, if someone is being held in a jail until there is a c- uh, a court appearance and they're sentenced. Then if they are sentenced to something longer term, then does the, you know, workhouse versus prison come into play, but the exact details of that I'm not sure.

I: Okay. Um, so could you also tell me a bit about what you know about probation?

P: Um, I, all right. I think that probation is one form of legal repercussion, so if someone has been found guilty of something, depending on severity they will be put on, uh, uh, some form of monitoring system in which they have to, you know, have a probation officer, follow certain rules, have certain monitoring, maybe drug monitoring or, um, other monitoring depending, and if they, uh, break that agreement then that could mean time not out of the, uh, jail system I suppose.

I: Mm-hmm (affirmative). And then, could you tell me a little bit about what you know about parole?

P: All right. So I think parole means someone who has, uh, been sentenced, has had some sort of legal monitored time. I don't know if that means it has to be, uh, in a jail, or in a prison or a workhouse, um, or just probation, but then it's, afterwards there's a duration of time where they have special monitoring, um, and then if they, um, fall through with that plan, then there are legal repercussions.

I: And could you tell me a bit more about how you distinguish probation and parole?

P: I'm not really sure. Just based off of the terms I think, uh, I would think probation would come before parole, uh, that someone could be on some sort of probation monitoring but that’s a part of their sentencing, and that parole is after they've completed their sentence, but I'm not sure.

I: Okay. So now I'd like to learn a bit about your background in education and training. Um, during medical school did you receive any training, whether that was formal or informal on working with justice involved populations?

P: No.

I: No? Do you think that there would have been any training during medical school that would have been helpful to you?

P: I think, um, it, it, I definitely think it could have been helpful, yeah.

I: Mm-hmm (affirmative).

P: I think, um, especially the way medical school currently is during first and second year, stuff we learn that is real life applicable, because we're cramming for tests it may not sink in, and so it, it, I think it would be highest yield after we've al- already been, uh, you, you know, taking care of people during a third or fourth year.

I: Mm-hmm (affirmative). And then during your residency was there any training on working with justice involved populations that you received?

P: Uh, none officially.

I: Mm-hmm (affirmative).

P: I think there likely have been times in my clinic or in the hospital that, where there was someone who was justice involved, um, and maybe a supervising provider would have known a little bit about it and would have told me, but nothing that was, like I said, a true, um, uh, besides on the fly.

I: Mm-hmm (affirmative).

P: So noth- nothing curricular based.

I: Yeah. Is, um, is there a specific example that comes to mind when you would have encountered, um, that when working face to face with a patient?

P: Um, I think most of it's with hindsight.

I: Mm-hmm (affirmative).

P: Um, you know, I'm sure there was times, uh, during my residency when I was seeing patients at my clinic and someone may have had, you know, uh, been on probation or parole, um, and, you know, in hindsight me not knowing the difference and not knowing exactly how things could impact them, um, uh, uh, uh, and so by not really knowing I probably wasn't asking the right questions about what kind of supports they needed for housing, et cetera. Um, also more recently, learning a little bit more about housing insecurity, and finding out what, um, uh, you know, uh, uh, being involved in the justice system can do to obtaining housing. I'm just starting to learn a little bit about that.

I: Mm-hmm (affirmative).

P: Um, but it is difficult for me to think of any concrete examples from my training.

I: Mm-hmm (affirmative). And so, in terms of I guess, like your example about learning about housing insecurity, how, is that part of your job training, where is that coming in?

P: That's just trying to take care of people.

I: Mm-hmm (affirmative).

P: Um, and not a part of my training at all.

I: Okay.

P: Uh, but, you know, taking care of people, um, you know, if someone, uh, you know, it's tough to care of your, to improve your health if you don't have stable housing, and so, you know, my role is to try and help people improve their lives, so if that involves writing letters, et cetera, if someone has an eviction notice on them or if, um, uh, uh, someone has, uh, uh, you know, had some sort of legal challenges, um, I may not, you know, currently I don't have the background knowledge to know exactly how to help, but my clinic has extra resources, um, legal, um, resources, et cetera, to help people.

I: Mm-hmm (affirmative). And then, rewinding a little bit and, and going back, um, did you complete a fellowship at all as part of your training?

P: Nope.

I: No, okay. And then, so you mentioned your current place of employment and learning about housing insecurity, are there any other types of on the job trainings or just learnings that have come up around this topic?

P: Um, around-

I: Justice in-

P: Just-

I: Working with justice involved populations?

P: I mean, I think, I think more recently as I have been more involved with substance use, et cetera-

I: Mm-hmm (affirmative).

P: Um, you know, if I have someone that I am providing care for and there's certain medications that I'm prescribing, um, in the, uh, non justice system setting and then all of a sudden they, uh, you know, have jail time coming up, um, scrambling to figure out a plan that won't negatively impact their health. Um, so opioid dependence is a big one.

I: Mm-hmm (affirmative). And so, during your day to day visits with your patients do you ask them about their possible justice system involvement?

P: Probably way less than I think I do.

I: Mm-hmm (affirmative).

P: Um, I know when I have patients who are on, uh, a certain, you know, cer- certain medications for opioid dependence, I now do ask them if they have any, um, upcoming time in the justice system that they would be worried about, because I, you know, what I worry about them is withdrawing, um, decreasing their tolerance, overdosing, et cetera, um, but in my general clinic population, um, ye- yeah, I think far less than I think I do.

I: And when you do ask about it how do you phrase that question?

P: Let's see, do you have any upcoming legal troubles or challenges?

I: Mm-hmm (affirmative).

P: Um, that's, I usually ask it that way I think.

I: Okay. And do you only ask about future? Do you ever ask about the past?

P: Um, I'll ask, um, and not with everybody, but, uh, with, uh, so in patients, uh, patients that are within our, um, uh, you know, uh, uh, the, uh, opioid addiction dependence clinic, uh, we try and ask at the first visit if people have had prior challenges with the justice system, um, but mostly as a means of finding out is there anything we can be helping with, social work, et cetera-

I: Mm-hmm (affirmative).

P: Or to identify upcoming challenges, which again could negatively impact their health if a medication prescribed would lead to withdrawals, et cetera.

I: Mm-hmm (affirmative). And so could you give me an example of, or describe what you would do differently from someone who ha- who knows that they're going to have some type of upcoming legal trouble?

P: Yeah. So, um, can I talk specifically about suboxone? Is that okay?

I: Sure.

P: Um, so up until, uh, I mean, very, very, very recently, like my last day in clinic, uh, up until that moment if I had someone who was on suboxone, um, I would try and remind people “Hey, please let us, if you do have any legal challenges let us know,” so if someone has a court appearance and they're not sure if they're going to have jail time or not, or they're not sure what county, um, they're going to be, or if they're not sure if it's a suboxone friendly county, um, a care coordinator will try and do some leg work so we can find out do we need to do a taper, so someone's on, um, a lower dose, so that way if they're not able to get their suboxone, uh, wh- while they're, uh, in, in jail, uh, uh, you know, so they, the withdrawals will be, um, less severe. Um, I will make sure to prescribe re- medications to help with withdrawals, like Clonidine, Zofran, et cetera. Um, so that was kind of the standard, um, uh, and, and then really making sure that people knew if they were going in and it was going to be more than a week their tolerance would be dropping, and so I had to make sure they had our, uh, number, so that way they could be coming directly to clinic when the left jail.

I: Mm-hmm (affirmative).

P: Um, but very, very recently, um, uh, I now have access to the providers there, and I believe it is becoming possible to continue people in some of the counties on their suboxone.

I: And are there any challenges that you see to asking patients about this topic and whether or not they've been in ja- involved with the justice system?

P: (checking phone) Sorry. Um, I think as I've gotten more comfortable asking the question-

I: Mm-hmm (affirmative).

P: Um, that was always the barrier. Um, you know, I still struggle, when I don't know the solution it's tough for me to know the question. Um, you know, again, it's only been in the past couple years that I've gotten a little bit more knowledge in, when it comes to addiction, and so I've become more comfortable, uh, comfortable asking the questions that often surround people who have dependence on substances, um, but yeah, I, I think the biggest challenge again is if I don't have a solution on how to help it makes it difficult for me to know what questions to ask. Um, and again, if it's a day that, uh, you know, I don't have a social worker available or someone who may know a little bit more of the possible options that I can, I have to help someone, then it becomes even more of a challenge.

I: Mm-hmm (affirmative). And are there any benefits that you see to talking to your patients about this?

P: Uh, yeah, rapport. Yeah.

I: Mm-hmm (affirmative).

P: Yeah, just again, I always, uh, uh, uh, my main goal is for people to, you know, and 'cause I do care, to feel like I care. I think a lot of people don't have that, um, so part of that responsibility is trying to find out, I may not know the answers, but who can I help get the answers to?

I: Mm-hmm (affirmative). So could you tell me a bit about your just overall patient population? Who do you see on a day to day basis?

P: Cool. So I kind of work in two roles. I work in the hospital here-

I: Mm-hmm (affirmative).

P: Um, at the University, um, and so that's kind of a broad spectrum of people. A lot of people, um, you know, this is referral center, kind of tertiary care hospital, so, uh, transplant population, people who have the ability who, uh, you know, get very, very sick because they're immune compromised, um, but, uh, then, you know, the past couple of years in the hospital we have started to see more and more people admitted who are housing insecure, um-

I: Mm-hmm (affirmative).

P: Uh, who have, uh, substance use dependence, um, and as we are starting to ask questions about it we're getting more yes answers to trying to figure out how to help. In clinic, um, I work, um, I'm lucky enough to work at a clinic that, I guess is, is able to take people whether or not they have insurance, um, and so I have, you know, patients who, um, y- you know, come from anywhere, um, uh, but a lot of underserved, [neighborhood name], um, housing insecure, um, uh, uh, people of color, people who identify as Native American, um, people who don't have documents to obtain a lot of services, um, yeah, including insurance.

I: Mm-hmm (affirmative). And then, how would you, and for both of the places where you're working, how would you describe the income levels of your patients and how it may differ between locations?

P: Um, uh, so I've, I mean, just within the, uh, you know, you know, the university system here, I think it's quite variable. Again, you know, there's, um, you know, people who, uh, you know, that don't have any housing and are unemployed and have minimal resources or family support, but then people who are high up in the business world, so, uh, you know, pretty spread, um, but in clinic I would say most people are pretty low income.

I: Let's see ... And then following up with that, how would you describe their insurance status?

P: So I started as a resident, um, uh, in 2009, and I would see a lot of different people without health insurance right when I started. Um, now most patients that I have who have the ability to get insurance-

I: Mm-hmm (affirmative).

P: Do get some form of insurance, um, and so, uh, you know, uh, a lar- a lot of people have, um, uh, you know, state, state insurance, medicaid, um, et cetera.

I: Mm-hmm (affirmative).

P: Um, in, in clinic, um, but there's, you know, again, I have, I do have a, um, you know, there's a decent population of people who are not able to get insurance, um, uh, y- you know, not due to cost but due to documentation, et cetera, but then also, uh, there's a subset of people who legally could get insurance but aren't able to afford it.

I: Okay. And in your experience are you noticing any particular challenges or barriers that racial ethnic minority patients are facing?

P: Definitely. Um, you know, I think both in the hospital and out of the hospital.

I: Mm-hmm (affirmative).

P: Most of the people in the hospital who I am taking care of, uh, you know, to help with substance use disorder, um, the majority who get admitted, um, for infections have these infections due to lack of access to clean substance use supplies, and that comes with, uh, less social support system, less, uh, housing security, um, uh, uh, you know, less finances, no car. Um, um, what was the question again?

I: Oh. Do you see any, um, unique challenges or barriers that racial ethnic minority patients are facing?

P: Oh. Yes, okay. And so in the [cities name], um, you know, there's a much higher, uh, proportion of people who, uh, of color who are housing insecure, um, identify, you know, Native American, African American, um, et cetera, and so definitely I, uh, I see that kind of there.

I: Mm-hmm (affirmative). And then, how would you describe the disability status of the patients that you see?

P: Can you tell me a little bit more about what you mean by disability?

I: I, it's very broad, and leaving it open for you to-

P: Okay.

I: Define it for how, for yourself and your patients.

P: Um, so I guess it, it, there's the people who are ... That's a tough question to answer, because I think a lot of-

I: Mm-hmm (affirmative).

P: If, if I look at the word disability and just think of it as challenges towards, you know, uh, being able to successfully take care of their health, that's different than someone who has brought in a form f- uh, a medical opinion form or is on, uh, social security, um, and so most of the patients I take care of who have substance use dependence have, um, you, you know, some sort of challenges towards their health, like I said, housing, f- family support, et cetera, um, but then, uh ... Sorry. Can you say the question again?

I: How would you describe their disability status?

P: Okay. Um, I think, uh, uh, uh, you know, people who are housing insecure, um, are they officially, uh, in, in, you know, medical terms labeled as being disabled? Uh, probably not, but I think a lot of people have, you know, either, uh, you know, mental health, um, that's undiagnosed, PTSD, historical trauma, et cetera. So I, I guess to answer that question simply I would say a huge percentage of people have undiagnosed disabilities.

I: Mm-hmm (affirmative). And then, if you could put a number, maybe a percentage on those that would qualify for social security income or some type of, um, I guess more, um, like defined-

P: Mm-hmm (affirmative).

I: Or what's recognized as being a disability that would qualify you for additional assistance?

P: Um, I, I think, you know, I take care of a lot of people who, you know, when I make, and when I'm able to make the assessment, so not for social security, but for-

I: Mm-hmm (affirmative).

P: Medical opinion forms, I have a lot of people, uh, so probably 60% of people that I see who, you know, have, uh, you know, housing instability, et cetera, um, I think qualify for, uh, that.

I: Okay. All right. And for your patients that have some type of justice system involvement, whether it was past or coming up in the future, could you tell me a bit more about that, um, experience for you, what that was like for your as a provider?

P: To kind of find out they're, um-

I: Mm-hmm (affirmative).

P: So I think, again, still not, uh, not fully, uh, understanding what people go through and not knowing what questions to ask that I, I still think I'm missing a lot of different ways I potentially could help, um, but I know, um, you know, challenges finding employment, finding housing, um, those are some of the biggest challenges, um, and then that leads to, um, y- y- you know, having health insurance, um, uh, making to, it to appointments, um, leading to flare ups in their mental health, et cetera.

I: And are there any patients that are being specifically referred to you?

P: There prob- uh, from the hospital, yeah.

I: Okay.

P: Yeah.

I: Okay. And then, I'm thinking also just in terms of you mentioned you're an opioid per- um, treatment provider, are there any opioid dependent patients that are being specifically referred to your or your clinic for that service?

P: Yup. Yeah, and so-

I: And how-

P: So there's-

I: How are they finding you, you know?

P: Um, so there's a couple of different ways. People can, um, locate a couple of us by name on different websites.

I: Mm-hmm (affirmative).

P: Um, but then a lot of grassroots, so, um, the detox center know- will refer patients to us, the hospital here, um, uh, people who would benefit, um, and are interested in maintenance, um, can get referred, so those are the main ways.

I: Okay. And then, how do you think that justice system involvement may have impacted your patient's ability to access their needed healthcare?

P: Um, how do I think the justice system impacts their ability to get healthcare?

I: Mm-hmm (affirmative).

P: So I think if, I mean, so there's a cycle. If people are not able to get stable housing, then, you know, I think that's one of the most important things of having a stable life. If they don't have stable housing and if the justice system has anything to do with that, it's difficult for them to, you know, uh, uh, uh, have secure enough sleep patterns, uh, uh, you know, financial support, et cetera to make it to clinic. Um, so again, I think housing is, um, high up there as for one of the most important barriers people face.

I: And in terms of, you've mentioned, um, maybe making changes to substance abuse treatment plans, are there any other ways that having this information impacts your treatment plan for a patient?

P: Having the information about someone's upcoming justice?

I: Upcoming or past-

P: Or past?

I: If you do know about it?

P: Um, uh, uh, this happens probably less frequently than I think, but again, if I know that someone has had prior, you know, justice system issues, asking about housing security and financial security, and, you know, occupation security. Um, if someone is a patient of mine and they're having a challenge with any of those, with a landlord or whatever, you know, I offer to write letters. Um, uh, you know, we, like I said, we have a free legal clinic I can refer people to, so if I know that people are having challenges-

I: Mm-hmm (affirmative).

P: That's the first step, even if I don't have the answers, and so trying to make sure I'm asking so I can find those out.

I: Okay. And do you communicate with parole officers, or probation officers or the courts at all?

P: Pretty rarely myself, um-

I: Mm-hmm (affirmative).

P: Directly. Um, there have been a few times where someone will have had something happen and so I'll have, uh, you know, uh, one of our care coordinators trying to reach out to either someone's lawyer, um, that's usually more common. We have a couple people who have parole officers and our patient requests that we send, um, a certain test to their parole officer, and so my care coordinator will do that.

I: Mm-hmm (affirmative).

P: Um, so r- rarely am I directly doing it.

I: Okay. Could you tell me a bit more about, I guess, the information that's been transferred between the care coordinator and maybe the lawyer?

P: Yeah. Well, so if it was a parole officer, for example-

I: Okay.

P: Uh, so if, if we have a patient who's on parole, um, and the patient requests that, um, our, you know, we do urine tox screens, um, uh, uh, to help assess stability within our clinic, and so if the patients request that we send stuff directly to the parole officer, then there's kind of, uh, um, a release of information signed on both sides, and then, um, per the patients request with ea- each urine screen we will send it to the parole officer as the patient requests. So-

I: And then, aside from possible past or upcoming justice system involvement, what else are your justice involved patients dealing with socially? Because I know you've mentioned housing, but are there other things that you're seeing in their social lives that's also coming up?

P: I mean, I think it's, uh, housing, um, social supports, um, you know, uh, what type of social support do, uh, do people have, um, you know, chemical health goes along with, um, undiagnosed, uh, mental health, um, uh, PTSD from, uh, leading up to their, uh, you know, time in the justice system, during or after, um, making sure I'm asking more about, uh, uh, you know, depression, anxiety, if someone has been, uh, you, you know, is now in a new situation compared to where they recently were.

I: Mm-hmm (affirmative). And could you tell me a bit more about any of the physical health needs that you might be seeing among patients?

P: I don't, not a lot comes to mind.

I: Okay. Mainly the, more of chemical dependency, mental health-

P: Yeah.

I: Um, issues coming up. And are there any resources or services that you're seeing that your patients need but that aren't available to them?

P: Um, you know, I know offhand not that long ago we had a patient who had, um, recently been incarcerated, but I'm not sure, I think it had been for a while, and was really struggling. He had stable housing lined up, uh, and was really struggling job wise, and, uh, I can't remember the name of, uh, our care coordinator was able to work with him and find the name of a group that helps, and he was able to find a job, but it took a lot of effort on our parts of searching.

I: Mm-hmm (affirmative).

P: So again, uh, our clinic not having a great “This is where to go, this is ...” And, uh, doing it often enough to recall exactly the steps.

I: Mm-hmm (affirmative).

P: Uh, so I would say, um, there probably is finger at my, uh, uh, you know, know- the knowledge at my fingertips, but knowing exactly where to look.

I: Mm-hmm (affirmative). And that's for finding jobs, is there anything else that you're-

P: Jobs, housing.

I: Jobs, housing.

P: Yeah. And actually, jobs, housing, and, you know, we have, we're lucky at our clinic that we have a very robust, um, mental health support system. We have psychiatry-

I: Mm-hmm (affirmative).

P: We have therapy, but the wait takes a while. Um, it's much easier to get in to see me, um, to establish care than it would be to see a psychiatrist or a therapist, and so additional resources for therapy, specifically, that is both, uh, situation specific and culturally specific.

I: Mm-hmm (affirmative).

P: So, um, you know, therapy that, uh, you know, that understands what someone may have gone through recently to, you know, be in the justice system, et cetera.

I: And thinking broadly are there any changes to healthcare delivery that you would suggest to better meet the needs of people who have had past or upcoming criminal justice system involvement?

P: So I think, uh, you know, better access, um, uh, to chemical health needs period-

I: Mm-hmm (affirmative).

P: And mental health needs period, um, before, during and after someone was within the justice system, so, uh, better linkage.

I: Mm-hmm (affirmative).

P: Um, uh, you know, I think there's another great example. Uh, you know, if someone is, you know, has Hepatitis C, meaning down the road they could develop a very expensive cancer to take care of or need a liver transplant, if they're incarcerated, currently there's not a way to allow them to be treated, and you only need eight weeks, and so if someone's in a space for eight weeks or more they should easily be able to take a daily pill, um, and so lack of a, you know, healthcare system that would enable that is a challenge.

I: Mm-hmm (affirmative).

P: Um, and I'm assuming that, uh, that same idea would go for, uh, mental health, PTSD, et cetera.

I: Yeah. So thanks again for your time today. Before we wrap up is there anything that I didn't talk about or ask you about today that you would like to add?

P: I mean, I think, you know, the, the lack of training from medical school, and after, uh, not knowing what questions to ask, uh, et cetera are, you know, super important. You know, I, I go back to two and half years ago before I, you know, got waivered for Suboxone, I'm sure there was patients asking for me help and it just went over my head. Even though I view myself as empathetic I just missed it, and it was because I didn't have the solution, I didn't ask the question and I missed it.

I: Mm-hmm (affirmative).

P: And so I'm sure that's happening now when it comes to my patients who, you know, have, uh, justice issues, et cetera.

I: So thanks, that's the end of my questions.
