# Supplementary material for: Physicians’ perspectives on continuity of care for patients involved in the criminal justice system: A qualitative study
Source: PLoS One. 2021 Jul 14;16(7):e0254578. doi: 10.1371/journal.pone.0254578 (PMC8279398; doi:10.1371/journal.pone.0254578)
Supplement: S2 File — (ZIP) [file pone.0254578.s002.zip › Clean/Participant_14_Audio1_LH_deidentified.docx]

I: All right so to start us off, I'd like to get a sense of um, your, a general overview of what you know about the criminal justice system. Um, could you tell me what you think about the current state of criminal justice practices in the US?

P: Wow. Uh, I don't know you know how to answer that. I mean I think, I guess I'm in this um, maybe um, sector of politics that believes that we're overly incarcerating people. And that the rate of incarceration in America is way higher than in other countries. Uh, that does not seem right, something happened there. Um, so that's one thing. Um, two is, I mean I do think there's a lot of importance and there's a lot of good that come from law enforcement officers that keep a lot of order and safety, and I think there's ... we need to have law enforcement, so I'm not like, I'm not an anarchist by any means. Uh, and we, for many years, had sheriffs here at the hospital and I felt safer with their presence, so I appreciate law enforcement, but I do think uh, we've had a bit of and it's gone a little too far.

I'm very worried about mass in- mass incarnation, re incarceration and also um, private uh, prisons makes me really unsettled. And then the quality of healthcare one gets when incarcerated, I have some questions about that as well. So I, I'm a little unsettled by the criminal justice system and then of course, um, how we go to trial, how um, people with wealth can afford a higher quality defense than people without wealth. Those things, uh, make me a little uneasy about it as well. So that'll be my answer.

I: Mm-hmm (affirmative).

P: Is that fair?

I: Yeah.

P: Okay.

I: Thank you and next I'd like to discuss some criminal justice system terminology and so could you explain to me what comes to mind when you hear the following terms, and I have a few that I'm gonna ...

P: Okay.

I: ... go through, and the first is prison.

P: So there's jail and there's prison. (laughs) Uh, I feel like prison is after you have been convicted and sentenced uh, and then you go to prison, and I think jail is when you're waiting to go to trial, but I'm not totally sure.

I: Mm-hmm (affirmative). And then could you tell me a bit more about maybe some of the characteristics about prison and jail and how you distinguish between the two?

P: Yeah, I'm not [crosstalk 00:02:52] I'm not good at this. Uh, next you may ask me about parole and probation and I'm gonna screw that (laughs) too. Uh, I, my understanding is that jail, for example, the county has a jail. People go to jail when they're being held for example for trial and they're not released on bail or they can't afford their bail, and they're being held in jail. Um, but that's separate from prison which is they've already been sentenced and now they're doing time, but I don't really know.

I: Mm-hmm (affirmative).

P: And so, I would say, I think of jail as being with the county, people go to uh, you know, they're arrested and they're held in jail. Uh, and that's the county building a few blocks from here. Um, whereas prison is like they're incarcerated, they're convicted, so I, but I'm not sure.

I: Yeah, and you're right. My, so my next term is probation.

P: (laughs)

I: So could you tell me what comes to mind when you hear that term?

P: Uh, well the probation is parole. And one of them is like your uh ... parole I think is you been released from incarceration and now they're keeping an eye on you. And probation maybe something like not relating to released from incarceration but um, there was a charge, you were convicted uh, and probation is like they're keeping an eye on you, while they're, as an alternative to being incarcerated or in some other way some less uh, so it's sort of monitoring, but they're free to move about. Um, so yeah, I don't really ... I'm not sure I got it, I got that right, but it's like I have many patients who are on probation. Um, I believe I got that they're on probation, but they haven't ... because they've been either convicted of a crime or they have been charged with a crime.

I don't know if they need to be gotten convicted to be on probation, but they rather ... but I think they've been convicted, they're on probation, they haven't been incarcerated but they have to check in with their probation officer, and if they something goes wrong, they get violated and then they incarcerated or they can have their probation extended, something like that. I think that's it. And then parole, I always get these confused. Parole is that they're released from incarceration and someone is keeping an eye on them after release. Uh, and in both cases, they get sometimes referred to that as their PO, so then I get confused too, 'cause it's either a probation officer or the parole officer.

I: Mm-hmm (affirmative).

P: But I don't really know.

I: (laughs)

P: I don't think you [inaudible 00:05:53].

I: So that's it. Like to shift a little bit to your background in education and training, during medical school, did you ever receive any training, whether it was formal or informal on working with justice involved populations?

P: I don't think so. No.

I: Mm-hmm (affirmative). And do you ...

P: Medical school, is that what you're talking about?

I: During medical school.

P: Yeah, okay, yeah, no. [inaudible 00:06:16] no.

I: Yeah, do you think that there would of been any training during that time that would of been helpful to you?

P: Um, yes, sure. Yeah, I think it would of been helpful. Um, it's medical school is weird, 'cause there's so many competing interests and so much competing curriculum, but yes.

I: Mm-hmm (affirmative). And are there specific topics or things that pertaining to criminal just- the criminal justice system that you think would of been particularly useful to know or to learn about then?

P: Um, well that's a good question. I think it ... a- after medical school, I got a lecture from the uh, one hour lecture from the um, medical director of uh, I think a prison system, not jail, but a prison system talking about what kinds of health services and healthcare and why are offered to people when they're incarcerated, and that was new to me. So what kinds of healthcare and health services are made available to people who are incarcerated, that's I think important information given the percentage of people that basically are insured by our prison system. Um, if I'm not mistaken uh, that's one way of putting it. I think that's worth learning about. Uh, I think ... I now think of incarceration as a health outcome of addiction. I think there's a lot of study on that uh, in one view, incarceration can be viewed as a health outcome to be minimized and then also with addiction, especially opioid addiction, I do addiction work, um, released from incarceration is a very important moment in one's health, not just in one's civil liberties.

So I think the importance of the week after release of from incarceration, especially prolonged incarceration for people with addiction, the importance of that, that's a very consequential moment for their health, where their very lives are at stake. So I think, um, what is offered in prison, in terms of health services, how incarceration is a health outcome, how release from incarceration can affect one's health, even uh, cause them to die, I think those are all issues um, that would be generally important for docs to know about. Um, there's some other kind of niche issues that happen sometimes. Docs will sometimes give more and more medicines to patients who don't take them, and are too shy to say, "I'm just not taking this medicine, doc, it's not working for me." So then they pile more and more medicines on top, and then the person gets arrested, incarcerated, usually jail, and jail looks at the medical record and just resumes all the medicine that was on the record, and then they become really sick in jail, because they were never taking those medicines to begin with.

And then when they are finally put in a structured setting, where someone is delivering the meds, they will sometimes go along and take their meds on record for them, but those were never meds they took, if you know what I'm saying.

I: Mm-hmm (affirmative).

P: So that's like a really important level of coordinating care, where we've had some very serious events because the medical record didn't reflect the patient's reality. Then they went to jail and those meds were started. So that's a healthcare. It sounds kind of niche, but for the county hospital, it happens frequently. Um, and what else? Health issues related to incarceration. Um, but there's other things like ... yeah, I'm gonna ... subset of what's offered in j- in jail or prison is two things. One, um, do we or don't we offer addiction treatment and how, and what does that, what makes up and why do we choose what addiction treatments we offer to people in prison, and that's very important to me. And then also um, pregnant women, some of the issues with pregnancy and addiction, and incarceration, and that overlap requires a lot of energy, and there's, I don't know prisons and jails don't always handle that ideally well. It causes a ton of drama. Uh, so those are some of the issues that come to mind in terms of why docs would benefit to know more about incarceration.

I: Mm-hmm (affirmative). And so that first, that one lecture that you had, w- what was the context of that? You said it happened after ...

P: Yeah, it was just ...

I: ... medical school.

P: ... you know, [inaudible 00:10:59] here. You know, know almost every week we have a one hour lecture and they go around and they recruit speakers, and it was just someone recruited this guy and he showed up and he gave his grand rounds and he left. And so it wasn't a structured curriculum, it was just someone though he'd be a good lecturer and brought him in.

I: Okay.

P: That was it.

I: Yeah. And so a similar question related to your residency. At that point, was there any training that you remember either in patient experience ...

P: Um ...

I: ... or ...

P: Boy, I mean we had a lot of casual training in the sense that we saw a lot of instances and we debriefed with our teachers, or mentors, or staff, but I don't know if there was formal curriculum, I don't think so.

I: Mm-hmm (affirmative).

P: I don't believe there was formal curriculum in the residency.

I: In some of those, um, debriefings that you had, could you give me an example? Was it related to the criminal justice system or and a patient that had some type of involvement [crosstalk 00:11:56]?

P: Yeah, we often have patients who admitted to the hospital ...

I: Mm-hmm (affirmative).

P: ... for medical evaluation, either from jail or sometimes from prison, or sometimes they haven't even ... they've been charged with a crime but haven't even been taken to court, and then the, the law officer will literally stay in the room with them. And then we have to negotiate the medical care we're giving that individual with the law officer in the room with them, and then how do we do that? And, and you know, and I'm a young impressionable resident, I just ask whoever is my supervisor at that moment and I hear his or her take and I ... that's, so that's how you learn is just ...

I: Mm-hmm (affirmative).

P: ... by hearing how the supervisors would handle it and going along with it.

I: Okay.

P: And, and then learn on the fly. That's about ... and then yeah, that's about ... and then talking to patients, and patients tell you stories.

I: Mm-hmm (affirmative).

P: And then you just, you learn a little bit more about it through that, but there's not formal curriculum.

I: Okay. And did you cre- uh, complete uh, fellowship at all, as part of your training?

P: Addiction medicine.

I: Mm-hmm (affirmative). And was there anything included in that fellowship related to criminal justice involvement?

P: It's interesting. A lot of patients, and a lot of same kind of thing talking to my supervisors, but intensely more so because the rates are higher, but uh, again, I don't think there was formal curriculum either.

I: Mm-hmm (affirmative).

P: Interesting. It should of been for sure, addiction medicine should have a formal curric- curriculum on this.

I: (laughs) And have there been any other opportunities here uh, provided by the health system?

P: Well the only opportunity, the one that I listed ...

I: Mm-hmm (affirmative).

P: ... might of happened after residency.

I: Okay.

P: And it was already during uh, when I'm a staff, and they just bring the speaker in.

I: Mm-hmm (affirmative).

P: Besides that, I just you know, I happen to know [doctor name], who I think is one of the um, the jail doctors.

I: Mm-hmm (affirmative).

P: Uh, I don't know what her formal title is and so I talk with her about it, and then I happen to know [doctor name] who's an addiction doc, who's very interested in uh, some of the death rates that happen in people with opioid addiction after release from jail. So I'm aware ... I have connections that educate me, but again, uh, informal, not formal curriculum.

I: Okay. And so now thinking about your day to day visits with your patients, do you ever ask them about their current ...

P: Oh yeah.

I: ... or past involvement ...

P: Oh yeah.

I: ... and what does that look like?

P: Yeah, so I mean when I'm dealing with a- addiction, not always general medicine, but when I'm dealing with addiction, I routinely ask them if they have legal issues.

I: Mm-hmm (affirmative).

P: Um, and there's a variety of reasons for that. Um, but the simple answer for that is that for patients who are in a supervised, in a supervised setting, so to speak, a probation, or parole, or whatever, they have, they are trying to prevent incarceration. They're trying to um, provide appropriate urine drug s- (laughs) drug samples to their probation officer, so they ... and this is important to understand their motivation, one, for why are they trying to maintain sobriety in this moment. Um, and then I can be an explicit ally with them on um, remaining out of in- you know, in- out of incarceration.

Um, uh, so I'll ask them about you know, is there any legal issues uh, are you currently being monitored? How often do you see a probation officer? Do you get along with him or her? How often are they testing urine drug screen? Are you, do you have any concerns about that? Are you um, using any drugs? You'd be surprised. There's people that are regularly using drugs, even at the risk of violating their probation and being incarcerated um, and then they just, they're hoping they can outsmart the system. Maybe they can, (laughs) but I don't know. Um, so I will try to both, be their allies um, also it helps me know that sometimes if they're resistant to giving a urine drug screen for me, it may be because they know that it's going to have a drug in it that they don't want their probation officer to know, and if it gets documented objectively then the probation officer can leverage them by demanding a release of information, and then access these. So sometimes the patients are resistant to giving drug screens to me, and knowing that they're on probation gives me a clue.

And then I can have a conversation with that patient about what's really going on. So that's another issue. Um, I also want to know for people with opioid addiction, they need to understand the risk after release from incarceration of relapse and [inaudible 00:16:51], so I um, someone heading towards incarceration has to understand what will happen after release. And I need to make sure they understand my policy, which is as soon as they're released they, they need, you know, [inaudible 00:17:04] priority, I'll get them back in if they need anything. They could even call me and talk by phone and I could arrange for some things to help keep them safe in the interim. So that's another issue. And then um, the other issue is for those that are on medications that would cause withdrawal when incarcerated. And the prison system or the jail system doesn't offer those medicines. We have to have a plan in advance of their ... they'll often have this court date, where a sentencing date and they don't know if they're gonna get jail time or not, a little or a lot, they don't know.

And they're on this medicine that they want to stay on, but if they're incarcerated, they'll be forced off of it. Um, and so I have to work with them to make a contingency plan for how do you want ... and if they relapse before the court date and are caught, they're much more likely to have a bad sentence. So it's this like thread and this needle complicated things where they um, we gotta keep them sober, but we gotta lower their medicines or discontinue their medicines, because if they happen to be incarcerated, then they're gonna be taken off abruptly and then they're gonna ve- feel terrible for the first couple weeks in prison and et cetera. So all those things are sort of complicating issues ...

I: Mm-hmm (affirmative).

P: ... um that I had, that I talk about routinely with my patients who have addiction. Uh, the general population of patients I don't routinely talk about it but it's kind of in my repertoire of questions I might ask. Um, when I work with patients trying to quit smoking cigarettes, they will say, "Oh yeah, I quit cigarettes once for nine months." Right, and then, then I may, I may delicately inquire what was happening during those nine months and sometimes they were incarcerated and there was a no smoking policy while incarcerated. And then I'll reflect with them about that nine months of non smoking while incarcerated on the one hand can boost their confidence that they can do it. But on the other hand, it's not like not smoking out in the, out in the real world where there's cigarettes everywhere and they have more freedom.

So I'll process with them their past non smoking time in light of the fact that they were incarcerated during their non smoking time. So I'll talk about that occasionally and then, yeah that's about it. I mean I'll ask the general average patient about their incarceration history sometimes, but not always. But ...

I: Mm-hmm (affirmative).

P: ... if it seems relevant, um, I'll do it. Um, like if I sense that they're not taking their medicine and there may be a court date coming up, I may explain to them that um, if they're not taking their medicine and then they become incarcerated, their jail or prison may try to institute al- all these complicated meds that they're not even taking and that could harm them. Um, so I guess those are all issues.

I: Mm-hmm (affirmative). And are there any benefits that you see to having these conversations with your patients?

P: I mean I, uh, it's hard to know that I'm benefiting my patient in like a vigorous scientific way.

I: Mm-hmm (affirmative).

P: Um, however I feel like it is professional and ethical for someone with an opioid addiction to clearly warn them about the dangers of overdose death after incarceration and offer services. Um, and do that in as clear way possible. Um, I think that's professional and ethical, but I can't prove that that conversation has a definite benefit to them. I believe it does, but I don't know. Um, and then besides that, I think if someone has been incarcerated and they realized that they can talk to you about it in a nonjudgmental way, I do believe that that is relationship building. Um, such that they realize that you are truly just trying to take care of their health. And it doesn't matter to you if they've been incarcerated or might, maybe about to be incarcerated or they've broken the law. It doesn't matter to you either way. You're just trying to take care of their health. And I think when they gather that, they um, I think most ... my sense is most patients are relieved when they can talk about it and they don't feel judged.

And but I may be biased on that, and so I do think it helps um, the interview go more smoothly. And uh, that they'll be more forthcoming with other struggles they're having. That's my belief, but I don't know.

I: And are there risks or challenges that you see when bringing this up with any of your patients?

P: You know, I suppose I slow down and I try to use words carefully. Um, I there's al- I've had ... I can't tell you how often patients have corrected me on jail versus prison or probation versus parole. So and I don't take it personally but uh, we'll actually get corrected, I think, because we don't know what we're talking about (laughs) with some of those things. Um, but I slow down and try to put my words in a nonjudgmental way. I think that's a bit of a risk to say things in a judgmental tone.

Um, if it's not relevant, um, I ... there are some people that still harbor resentment for what happened to them, and it brings up a really painful memory. They might become emotional or feel defensive. That's pretty rare actually, but o- occasionally some people might get a little defensive and you know, aggressive toned, like this has nothing to do with that you know? So why are we talking about that? Um, you know, I'm here for my health not for a legal conversation. That's pretty rare, actually surprisingly. Um, I get, you know, not to be cynical but waste of time. Like am I, is it, if I'm busy, how do I value my own time and does this conversation about anything, not just about criminal justice, name the topic. Is it moving my day forward? Is it helping this person or am I just wasting, spinning my wheels with this. And there are sometimes where someone's here for a pneumonia and I'm getting them antibiotics and they were incarcerated four years ago. It just doesn't matter.

Uh, I'm giving you antibiotics either way. If you want to talk about it, you can bring it on, but it just doesn't affect my plan, so I kind of probably not ask that question in that setting, 'cause it feels like a waste of time to me.

I: Mm-hmm (affirmative).

P: Um, so that's kind of my thoughts on that.

I: Yeah. And when patients do get a bit defensive, how do you respond to that in that situation?

P: I mean i- it depends, if it, if it is uh, someone who's got opioids addiction who's looking at incarceration, I would just say that this doesn't ap- I don't, I'm not saying this applies to you. I'm not judging you in any way, I just want you to know that many people struggle in the first couple weeks after incarceration and if they relapse, they're at very high risk of death. And I just want you to know that, and I want you to know that you, you could always come you know, see me or call me if anything happens, and then drop it.

Uh, just to like as succinctly as possible deliver that safety message to him or her, but otherwise if they don't want to talk about it, I think the other setting would be um, if they are being monitored in some way, I might say, "Look, uh, the reason I brought it up was that I um, I want to help you stay sober and stay out of trouble with your probation officer. I want to help you with that way. Um, I will," you know, and be clear that I will not release your private information unless there's a release, but I have heard of some probation officers demanding releases be signed. "So I respect your need or desire to privacy, um, I want you to know that my goals are probably similar to your goals, um, but I also want you to know that sometimes the probation officers can be a little pushy about release of information. Um, so I want you to know about all that." And then let them make their own choices.

And then if it's just random, like if it's a ... they try to quit cigarettes and they say, "Five years ago, I quit for 12 months," and then I ask them, you know, I im- I gently inquire were they incarcerated and they seem defensive, I just drop it. I'd say, "I'm sorry, you're right." That we don't have to talk about that." That's totally your business um, uh, you know, and if they seem like they need an explanation, I would explain why I asked, but I don't know, I'd just drop it if they're not comfortable with it. It's pretty rare. In fact, it's surprising how often you could ask ... well I rarely ask people what they were incarcerated for. It's pretty rare. Um, but I have been surprised that when I do ask, people just seem to tell you, and they may be lying, but they don't seem to get defensive. They seem to just say, uh, this is what happened, you know? And, and so I'm surprised at how little defensive people are, um, but I also I think, you know, I try to be careful about wording it well.

I: Mm-hmm (affirmative).

P: Yeah.

I: And so could you now tell me a bit about your overall patient population and like who are you seeing on a day to day basis?

P: Hm. Eight weeks a year I do general internal medicine, hospitalized patients at the county hospital. The rest of the time I do either opioid addiction clinic or tobacco addiction clinic, or people in our county hospital pain clinic whose got, who are having some problems with their medications they're overusing them or something like that. So it's a high risk population, a lot of opioid addiction, tobacco addiction, pain patients. And then eight weeks a year, the general medicine patients.

I: Okay.

P: I have 10% of my times in a rural, rural county and the rest is up here.

I: Okay. And how would you describe the income levels of your patients?

P: Hm, predominantly low income, predominantly, I, I don't know if this is a bad word but indigent patients. Patients who do not have private health insurance and are either Medicare or Medicaid. I have a few patients who have um ... a lot of disabled patients. I have a few patients who have um, private insurance. I view patients who have six figure salaries but that's the minority.

I: Mm-hmm (affirmative). Lets see. And then when you say have a lot of disabled patients.

P: Mm-hmm (affirmative).

I: Could you expand on what you mean by disabled?

P: Both mental health disabilities ...

I: Mm-hmm (affirmative).

P: ... traumatic brain injury disabilities, and especially in the pain clinic physical disabilities um, so it's a lot of the ... if you ask, if I ask my patients what their source of income is uh, uh, what's it called, SSI, uh, disability is a common answer. And they'll have had uh, motor vehicle accident or some other kind of physical incident that caused physical trauma to their body or sometimes severe mental illness, or maybe they're on dialysis or something else. So um, there's a medically ill group of patients I would say, and then they're just you know, I was just refle- I think when I said that, reflecting on the fact that uh, many of them are not gainfully employed. Right, they're in or yeah, they're um, they're struggling to uh, do what I think stereotypically we call normal lifestyle. (laughs)

I: And have you noticed any particular challenges or barriers faced by patients from racial or ethnic minority populations?

P: Hm, I think ... I mean I've been here for since 2002, so that's nine, 17 years. So I've gotten more comfortable talking to people from a variety of different racial backgrounds, um, but even so I would say where do I struggle, I think I probably struggle the most with Native American folks. Um, I could guess why, but um, I just don't think I have as much cultural competency. Um, I, we have many patients who are Native American, and I do my best. I think I do fine, but I think I could do better. Um, I uh, I think I do okay. I don't know if I do okay. I think I maybe not great, but okay, talking to African American populations um, here um, in particular about the criminal justice system and I think uh, I probably have a little bit more ... I mean I've just read more and I have a little more understanding about um, criminal justice disparities when it comes to African Americans than I do when it comes to Native Americans.

We don't see many uh, people uh, Mexican Americans or I guess we have some Ecuadorian patients in this hospital. So I don't see or talk to many patients um, you might call Hispanic about criminal justice issues um, and they have a pretty low rate of attending the addiction clinics. So we don't have many in our addiction clinic. And then um, we have a large Hmong population but they tend to be either less criminally justice involve, criminal, criminal justice involvement or uh, we're just not talking about it with them. So it may be happening but we're just not talking about it. All of my encounters with Hmong patients usually have a culturally competent Hmong interpreter drug counselor with me, who's probably helping me navigate some of the cultural issues.

So uh, and maybe steering me away from some. So and then who else? And then you know, Caucasians, I will talk pretty comfortably with them about criminal justice issues. So I would say most of my conversations are Caucasians or African Americans. I feel a little uncomfortable communicating with Native Americans about criminal justice issues. And I think I'm probably just not seeing it in the Hmong patients or the Hispanic patients.

I: Mm-hmm (affirmative).

P: That's my breakdown.

I: And so, I guess when patients, when they are coming here um, whether you know, Native American, African American, Hmong or, are you seeing them having a difficult time just accessing care?

P: Um, meaning people who are criminally in- criminally justice involved patients or just generally?

I: Generally.

P: Um, I don't know if it's a, it's so funny like I don't ... it's hard for me to guess what their boundar- what their barriers are.

I: Mm-hmm (affirmative).

P: Um, I have been here for 19 years and I really hope that we're a place where people ... I've, I love walking through the lobby and seeing the exciting mix of people from all sorts of backgrounds and nations and whatever, and I hope they feel welcome, and I hope ... I mean there's county hospital craziness, but that's irrespective of one's race. There's so- the call center may not know your race. They're still hard to navigate, the call center. I don't know if you've ever tried. Uh, so I, I don't think that's a racial issue. That's just our hospital frustrating sometimes. So there's definite frustrations with the hospital but I hope that those frustrations are equal for people of color as they are for Caucasians. I don't know that, but I hope that.

Um, on some occasions, I've had patients uh, become angry and point out their race and be concerned that they're not being treated equally because of their race. That's fairly rare, but if you know, one in 100 person say it out loud, how many people are thinking it? Uh, I don't know the answer to that um, but yeah, it's, it's happened. People have said that. Um, it's surprisingly rare. I don't know the barriers that ...

I: Mm-hmm (affirmative).

P: ... people of color face when they try to navigate the healthcare here and whether it's worse or better than elsewhere. I don't know I just I, so I don't know. It's a long way of saying I don't know. (laughs)

I: And so for your patients with justice system involvement specifically, could you tell me a bit more about what experience is like, if you could expand on some of the things that you've already talked about?

P: I mean I, I honestly I don't want to dwell on it.

I: Mm-hmm (affirmative).

P: Like tell me about your ... the trauma that you've experience while incarcerated or I certainly steer way clear of, "Tell me about the moral decision making you made in whatever act that you led you to be incarcerated." I steer way clear of that. Like so I'm not trying to ... some people actually want to be declare that they were innocent or that they ... or something was done to them wrongfully and I will listen and you know, express uh, remorse or like, "I'm sorry that happened to you," kind of thing. But I'm not, I'm not gonna, I'm gonna steer way clear of, tell me about ... I don't want to fetisize their incarceration experience. I want to stay as focused as I can on the medical aspects and giving them good advice to my knowledge, but I don't need to hear details about you know, they were wrongfully committed or they were wrongfully uh, convicted or how terrible it was or um, what they specifically what they did. I usually won't ask.

Um, I just don't care what [inaudible 00:36:09]. I mean I ... maybe I cared 15 years ago (laughs) but I'm just trying to like deliver medical care. So how does this affect my medical decision making? I try to keep it on that, and I try to make s- but if they want to talk about what happened I'll listen, you know?

I: And do you get patients that are specifically who are justice system involved that are specifically referred to your care?

P: Yeah. Oh yeah. One of the priorities for our addiction clinic is people rec- with opioid addiction recently released from incarceration. We will ...

I: Mm-hmm (affirmative).

P: ... basically our three hot spots are HIV positive, pregnant, and just released from incarceration. And those folks are high risk each in their own way, and we will fast track any of them. So yeah, we're ... and then we have arraignment with a um, uh, with the, the jail. Um, if a pregnant woman is opioid addicted is incarc- is incarceration in jail, we have arraignments. We can deliver methadone to jail. There's lots of issues around continuing methadone or discontinuing methadone for people who get incarceration, and we work with all that. So there's a lot of talk about that.

And then they get out of jail and they return to the clinic and they say, "Gosh, it's been 12 days since I've been you know, since I've gotten any of my methadone doses, and now what do I do? And how do I start things up again," and that kind of thing.

I: Mm-hmm (affirmative).

P: Really common when people get sober in the next 18 months all the legal stuff shakes through. I mean it's, you think you get sober everything is done. As you get sober and then you got more than a year of those charges that are hanging over you and how do you ever, when do you ever get sober and clear of it all. And it takes years for some of these folks. So you, so it's very common per people in sobriety dealing with legal issues including ... it's frustrating to me, but they've been sober for 16 months and now they have to go do 30 days in some prison from a charge that happened four years ago, uh, when they were still using. And they've come this far and there's a risk that they ... everything gets thrown out, and then we have to talk to them about that. So yeah.

I: And you mentioned um, probation officers and some being pushy about um, getting releases of information. Do you ever communicate ...

P: Yeah.

I: ... with probation, parole, and what does that look ...

P: I u- ...

I: ... like?

P: I usually don't communicate with them. Uh, but I would say one out of 10 times, either the patient will want me to advocate for them in some way and will ask me to call to explain the how well he or she's been doing in treatment and to say what my relationship with the patient consist of, how many visits have seen that patient and how they appear in the clinic and yada, yada, yada. Um, so there ... about half of the time ... so very frequently do I speak myself speak to the probation officer, but it's not impossible and when I do, half the time it's by the patient's request to advocate for them. Half the time the probation officer will call to confirm something or verify something or to reveal some information to me that I didn't know, or um, something like that. Or some ty- you know and a lot of these POs aren't that terrible to people. They seem like they're uh, often, not always, but often they actually care about the per- the patient.

You know, and they are willing to engage with me or they just let me do my thing and let them be on the medicine they need to be on and leave them alone basically and just try, hold them accountable for these few things and then uh, ho- hoping not to cause problems for the patient. Um, I've heard of some POs that have, seem vindictive though. And I don't know if that's just the patients telling me or if it's really true or a little bit of both. You know?

I: Mm-hmm (affirmative).

P: So I've heard about, I've heard about that as well.

I: And in cases when probation officers are reaching out to you to share information with you um, could you give me an example what that is? I don't want to get into any like potentially confidential information ...

P: Yeah, no, I wouldn't say any patient's names.

I: Yeah.

P: But I would say like they may say, um, recently I had a guy who's under supervision and he um, worked with his probation officer to check himself into a more structured addiction center and then to transition to a sober living arraignment and it's all in coordination with the probation officer and she called me to inform me, "Hey, Mr. So So, is, has chosen to check himself into treatment 'cause he's struggling on more than he led on, and then he's gonna transition." So she actually called me to tell me his plan.

I: Mm-hmm (affirmative).

P: Um, and this guy is kind of enough of a knucklehead that he wasn't good at communicating, so it was nice for me to hear from someone to say, "Here's the actual plan going forward." And then she even said, "Can you stay involved in his case? And here's what you need to know, blah, blah, blah." So that was nice. Uh, so that was a call from the probation officer. Um, I had um, one guy who was real troubled, really struggled, had some violent impulses, had some violent crimes in his past with ongoing drug use, traumatic brain injury, real troubled guy and um, the PO was somewhat alarmed by how he was do- how he was doing because he wasn't doing well, and she called me and she was just a little ... she had a release and she was just a little alarmed. "It seems like Mr. So and So is not doing well right now, what do we need to uh ... is that what you're observing? And what, what if anything can we do about this?"

And um, yeah, so that uh, sometimes I've had them call me to verify uh, so they'll get a drug screen and it'll be positive for a medicine that I'm prescribing. The patient will say, "Dr. So and So, you know, Dr. So and So is prescribing it," and then they'll be suspicious, so then they'll call me just to verify.

I: Mm-hmm (affirmative).

P: And so that's a common one. And um, those are all instances where I've talked to a few POs. Um, I think [inaudible 00:43:11] trying to think if there's anything else. Those are most of them.

I: Yeah. And then aside from being involved with the justice system in some way, what else are your patients dealing with socially?

P: I mean I ... there's a lot of who is their family and I don't necessarily mean biological family, but who is their family, their support network. So we, with an addiction that keeps going, what happens is when you don't have an addiction, I mean you got your college friends and you've got your work friends, and you've got your family, and you've got your you know, romantic life, and you've got I don't know the dudes you watch football with. You got all these different networks of friends, but then when you get addicted, you start losing those networks and you have fewer and fewer connections. And then you one day you realize, "Yeah, I got fired from work and my family doesn't want to talk to me and all my silver friends have all ditched me long ago, and my only social outlet is um, other drug users. And sometimes my probation officer or my addiction doctor are like the literally the only sober support I have in my life."

Um, some, and so understanding how badly the damage their network of healthy support and whether there's anyone around anymore and how to rebuild those and it sometimes if they don't feel like they can rebuild those, where to find healthy support network. And literally it's not uncommon for patients to say that I'm the only sober person in their life. Well that's not gonna work for them, (laughs) 'cause I'm their doc. I'm not like their companion. Uh, and um, I, I would not be surprised whether a PO plays a parental role in essence for some of our patients. Um, accountability, structure, sobriety, and they may never of had properly in their life. Uh, so but in some cases it, some of these folks have a really resilient network. Uh, people that despite all the tribulations have stuck with them and keep picking them up and supporting them and I, and so I want to know how resilient their network is, social network is. I want to know what their financial situation is.

Uh, uh, I can't ... I don't routinely, but I will not infrequently ask them if they are in debt to anyone, including a drug dealer, and what do they expect to happen with that debt, how are they gonna pay it back. And do they feel threatened? Do they feel safe? Um, uh, yeah, how they choose their sexual partners and what sex is for them. That's another thing I'll talk about and um, uh, sort of depending on the circumstance I might ask about if they're safe in their relationship. If that's a healthy relationship. What else? I mean I, I want to know, do they work? What kind of work they did do? What kind of work they did do? What kind of work they would want to do? What's holding them back from working? Um, mental health stuff as well. That, that's a list.

I: Yeah.

P: Probably more. Can't think of it but ...

I: (laughs) Well in terms of mental health needs, what are you seeing there?

P: I mean there's a ... obviously there's a ... well and part of the reason I think I don't connect as well with the Native American population is, it's a lot of trauma. And I don't know that they are as ready to talk about the trauma they've experienced with me a white man. Uh, I just ... that's my assumption. I don't know but there's tons of trauma that's un- untreated. Not just among Native people but broadly, um, but that group in particular I think is probably a little more traumatized and a little less addressed those traumas. Um, so I think the trauma is a big deal. Um, I think depression ... so schizophrenia and bipolar are major mental illnesses that kind of jump out, jump out at you in their own. Like you could see those usually and then they have treatments available that we can try to get the patients into treatment. Depression and anxiety are very common, um, they can be very severe, but they also um, lots of doctors know how to treat depression and anxiety.

Um, so for different reasons the extreme ends, schizophrenia, bipolar, and the milder end, anxiety and depression are less of an issue, but trauma sometimes eating disorders uh, sometimes OCD um, those can kind of get into this sort of negative space is uh, shady area between the obvious things with treatment and the things that are common that everybody can treat. And they get kind of lost or it's hard to know how to address trauma or some of those other, um, but they're actually pretty common. So uh, I can't remember the question but (laughing) uh, but uh, yeah, I mean I think, I, I think the PTSD ... how to talk to a patient who is dealing with PTSD to get them moving in the right direction. That's a huge thing in the addiction world.

I: Mm-hmm (affirmative).

P: Huge. And um, the only other thing I would say, this isn't psychiatry but the, the brain injuries. I mean I can't tell you how often, when you ask a person they've gotten in some kind of altercation. They got hit in the head. They got knocked out and nobody's assessed them for permanent effects of brain injuries, concussions, things like that. Uh, so that's another kind of unmet need.

I: Okay. And are there any other medical related needs that you're seeing among the justice involved population at all?

P: I mean you know, people say [inaudible 00:49:21] are a really common thing, but that's just kind of traveler that go with um, with drug abuse. Um, I don't know this but I would be shocked if just the routine medical issues like tobacco use, high blood pressure, um, cardiovascular risks, risks for cancer, poor diet, I would be shocked if those things weren't worse in the criminal justice involved population. I'd be shocked. So I assume that's true, but that's less my field.

I: And then are there any resources or services that your patients need, but you see that aren't available to them?

P: Well I mean lots. I mean I think ready more ready access to mental health care clearly um, if you're talking about criminal justice, medication assisted therapy while they're incarcerated, that's uh, we will look back on this and say we incarcerated a huge portion of our population and set them up to die after release from jail or prison. And I think we're gonna look back on it and this is gonna look terrible, so it's from a civil right's point of view. Um, I, I, I think it's shameful that they get incarcerated and not given medications that they were stable on taking everyday without any issue and they still get cut off just 'cause the jail or the prison doesn't want to deal with that. I don't know. The uh, logistical headaches of giving a control substance for that reason, or they've got a different ... anyways so I think those are issues. I think um, yeah there's lots of housing needs. Um, yeah.

I: And then thinking broadly, are there any changes to healthcare delivery that you would suggest to better meet the needs of patients that are justice involved?

P: Well I mean, like I said, being able to continue a medication route, start a medication for addiction while incarcerated, uh, I, I don't think evidence is 100% in, but I think it's pretty clear it would save lives. I think that's pretty clear. I think all, a lot of circumset- cir- uh, circumstantial evidence have suggested that this would save lives. So appropriate evidence based treatment of addictions for incarcerated people for sure. Um, you know, say the question again. Um ...

I: Um, thinking broadly, big picture, are there any changes to healthcare delivery that you would suggest to better meet the needs of patients with criminal justice system involvement?

P: Yeah, I wonder how many criminal justice situations are the culmination sort of unmet mental health [inaudible 00:52:32] needs to begin with, that they're out there, you know, maybe not tonight but they're out there downtown [city name] in an, in a unstable mental position for whatever reason and they're drawing a lot of attention to themselves and along comes an officer of the law. Uh, those cops, they don't want to deal with that either. I mean and then well, you know, it's like we start at the point where someone is um, a little unstable in public and the police officer approaches, and how do we go from there? But can we just back it up and stop that interaction? Where someone's in public and uh, is in a vulnerable position and the person they have to interact with is uh, an officer of the law and not a police officer. That seems like nobody's winning. Once that happens, nobody's gonna win.

The best you, the best outcome is ... I don't know what the best outcome is. Uh, the worst outcome is bad. Uh, so maybe uh, beef up our sort of uh, community crisis resources, beef up our addiction of mental health resources, beef up um, housing options. Those are some ideas to try to prevent that event from ever happening. That doesn't always go well so that's just a thought. Um, and then yeah, I guess that's what I would say.

I: Okay. So thanks again for your ...

P: Yeah.

I: ... time today.

P: No problem.

I: Before I officially wrap up, is there anything that I didn't ask you today that you think would be important to add?

P: I don't know. I'm curious the difference between jail and prison ...

I: (laughs)

P: ... information for all. You'll tell me and then I'll forget me in five minutes, but that's okay. I still want to know.

I: (laughs) All right, so I'll ... yeah, we can turn off the audio re-
