# Supplementary material for: Physicians’ perspectives on continuity of care for patients involved in the criminal justice system: A qualitative study
Source: PLoS One. 2021 Jul 14;16(7):e0254578. doi: 10.1371/journal.pone.0254578 (PMC8279398; doi:10.1371/journal.pone.0254578)
Supplement: S2 File — (ZIP) [file pone.0254578.s002.zip › Clean/Participant_15_Audio2_deidentified.docx]

I: Okay, so thanks again for taking the time to meet with me. Um, so part of … This interview … the goal today is to understand what you know about the criminal justice system like I said, um, and how it impacts your patients, any experiences you've had, um, as a physician treating patients with criminal justice system involvement. And I wanna begin by getting a general overview of what you know about the criminal justice system. So to start us off, could you tell me what you think about the current state of criminal justice practices in the United States?

P: (laughs) That's a great question. Um, well I'm concerned about them. Um, maybe I should, um, give you some pertinent background. Uh, my father was a litigator, who for a number of years was first, uh, an assistant county prosecutor and summarily became a public defender for three counties in [state name], which is where I'm from. So, I grew up (laughs) with a lot of exposure to the criminal justice system. Um, not all of it necessarily favorable even though (laughs) my father was quite enmeshed in it. Um, I guess the other … I don't know whether it's a bias I have is, um, I spent a fair amount of time in law school as well as going to medical school. I was actually gonna be a forensic psychiatrist so the law has always been an area of interest then I potentially, um, opted out of that for reasons that probably aren't pertinent to your discussions.

So, um, years past not recently I read a lot about the interface between law and psychiatry, both in a kind of statutory way and also in terms of policy kinds of issues. Um, so there's that whole piece. Um, a second issue that's probably pertinent to your discussion is I was a psychiatric consultant to the [psychiatric hospital]. Are you familiar with that?

I: Could you tell me more about it?

P: Yeah, okay. So there is a facility which, um, is for people who are mentally ill and, um, incarcerated. And it's sometimes called the end of the line (laughs) in the sense that, um, a lot of the people … it only has I think about 225 beds, um, but it's an end stage facility in the sense that a good fraction of people who are there have committed capital crimes. Um, usually, they'd killed somebody. And in the system you get adjudicated mentally ill and dangerous a D, do you know about that?

I: No. Tell me more.

P: Okay. So, um, in the, in the judicial system you can be committed as mentally ill, um, and that allows a number of things to happen. I mean, you can be held. You can be committed to treatment. Medications can be forced on you, all those sorts of things. But, um, for some people who are more (laughs) problematic in the system, there is an adjudication process where they're given the letter D meaning dangerous. And a whole different set of rules govern their release from mental institutions, essentially that they can't be released just on the say so of a treating psychiatrist. The court has to endorse that they're ready to be released.

So a disproportionate number of people in the [psychiatric hospital] are adjudicated mentally ill and dangerous and they're people who have been assaultive, very violent, often killed people, so it is the last stop in the system. Okay. Um, and a number of people where a judgment is made, that they're not competent to stand in trial, um, will wind up at [psychiatric hospital] partly initially for an evaluation. And then sometimes there's a fraction of those people who are just indefinitely institutionalized there. So for eight years quite a while back I was a psychiatric consultant to the [psychiatric hospital]. I would go down there twice a month, a long sidebar. The original reason I got there was one of my residents became the medical director, a fellow I had trained.

I: Mm-hmm (affirmative).

P: And they had a gentleman who had been in five point restraints for a year because when they let him out of the restraints, he took out one of his own eyes, inoculated himself. He was very assaultive and the question was medications didn't seem to corral him and the question was what could they do about it. And the proposal was psychosurgery, um, have a neurosurgeon actually institute brain lesions to try to corral his behavior, um, which … I could talk to you forever.

There was a whole year in the United States where lobotomy and psychosurgery was the only mode or the principal mode of treatment and it was very crude and it didn't work very well. Um, probably somewhere in the 1980s, psychosurgery got a lot more st- sophisticated, done stereotactically with considerable precision in which the lesions were very small and precise. It's actually a very effective treatment.

So the question in this case was, could this guy be a candidate for psychosurgery. And, um, I at that time agreed and I was principally based at the university at that point and so the guy was the … one of the neurosurgeons actually went ahead and did the procedure and it, it worked very nicely. He was allowed, released from the restraints, but that was beginning of my spending some regular time down there.

So more than the average [inaudible] and that's probably the most proximity I've had because most of those people, um, were there because of criminal acts for which the judgment was they really weren't competent to stand trial. Um, of the 200 and some people institutionalized there, there was by the way in fairness a small women's unit, maybe 20 beds and the rest were men. Subsequently there's a women's … Shakopee, the women's correctional facility, you know, gets most to those people.

So [psychiatric hospital], we used to call the end of the line because it was the really the last stop for a lot of people. And, yeah, um, I spent a fair amount of time with people who had been very violent at one point or another in their career and, um, I was very interested in that. Um, interested in what actually triggered those kinds of behavioral patterns, um, and what was in their head when they did things.

Um, years ago, there was, um, a guy in Minnesota who was called a Pipe Bomber. He put pipe bombs in people's mailboxes. Um, he killed a number of people by doing that and so he was there and I got to talk to him, talked to a number of other people who frankly murdered people. Um, and yeah, I guess for me trying to understand something about what drove that behavior, um, was, fascinating, uh, so that's probably as much interest as I've ever had in the criminal justice system.

Um, and most of those people weren't going anywhere. I mean, it was a question of, um, you know, trying to manage them effectively with medications so that the aggression and the violence didn't manifest itself any longer. Um, I'm trying to think what else about that would be interesting. Um, so in that process there were many times that I was called upon to testify about the people that I'd seen and go to court so yes I've (laughs) appeared in court a number of times. I kinda got tired of that. That's part of the reason I opted for medicine over the law. Um, anyway that's a long side bar.

But, um, and then when I moved over to [County] from the university proper, um, we had a lot of dealings with the courts again in terms predominantly of commitment procedings, um, what are called, uh, Sheppard Price proceedings. It's about being able to involuntarily medicate people, um, who are out of control or problematic. Um, we also provided, um, the hospital still does psychiatric support to the [County Jail]. Um, we had psychiatrists who, uh, routinely conducted clinics in the jail, uh, and would see people. And that became, um, a point of some contention. Um, I don't know how much of the whole history about the sheriff and, uh, the struggles about the mentally and the jail system, and yeah.

I: [crosstalk] yeah.

P: Okay. Um, it's pretty complicated, um, sequence in which a lot of the mentally ill people are taken by the police to jail. Um, and, um, at any given time maybe almost 50% of the people in jail have major mental illness. And the sheriff, um, became very unhappy (laughs) with having so many mentally ill people, um, that he was responsible for. And despite the fact that we would have a couple psychiatrists kind of embedded there in the sense of running clinics, um, the sheriff became disgruntled about the fact that they were spending so much time dealing with mentally ill people.

And in fairness, they are very problematic in the jail. You're aware of all this, um, you know, aggression, um, some very, uh, problematic behaviors, uh. I don't wanna be too graphic but, um, not an uncommon one is about smearing fecal material all over jail cells and things like that. So, um, the sheriff at the time who's since been displaced. He lost the recent election and different fellow was elected. Um, the sheriff actually began to petition for legislative relief and he got the state legislature. He was very effective. He got the state legislature to pass a law that allowed the jail to lateral people from the jail directly to the state hospital. Um, the state hospital for the metro area is [local County], the [local county psychiatric hospital].

I: Okay.

P: Now, you don't know any of this. Okay. Um, yeah the state hospital for [County] …

I: Okay.

P: … is out in [local county]. And it at one point was a pretty large facility that probably in its prime ran 400 or 500 beds and it's huge, and a very institutionalized facility. And like as happened many other places in the country, the state hospital system sort of, uh, was sharply reduced and minimized, um, because the state didn't really wanna be in the business of taking care of the mentally ill, didn't wanna spend the money to take care of the mentally ill. And so the budgets the legislature would make decisions to cut back.

And so over a period of, I don't know, 15 to 20 years, any number of psychiatric institutions in the state were closed and finally got down to a bare number of facilities. Um, I should know the percentage that were closed, but so they're, they're not that many state hospitals left at this point and, and, and it's not unique to Minnesota. It's a national phenomena. Um, partly out of costs and partly out of policy issues that maybe you'll get to about how interested the state really is in providing treatment and care for the mentally ill. Um, and they … many places, Massachusetts for example closed all its state hospitals. Um, a number of other states have downsized sharply.

Um, so [local county psychiatric hospital] had gradually reduced its bed capacity and now even though physically there was ample room to run twice the number of beds, they're down to I think less than 200 beds at this point for the entire metropolitan region. Um, here at [health system], we run 105 psychiatric inpatient beds, which for the most part are usually full. I mean, the demand is very substantial. And the state has incentivized the hospital for years about, um, trying to care for some of the psychotic patients and obviate the need to lateral them to the state hospital. So there's actually program constructed between [county], [local county psychiatric hospital] and this hospital to take in psychotic patients under a contractual arrangement and treat them at a pretty good price per day in lieu of sending them to the state hospital, another way of obviating the need to run all those extra beds.

Um, so with 105 beds that are generally filled, some fraction of the people that we would accept we couldn't turn around in the 28-day period that was allocated. That was … The idea was if we could treat them aggressively, medicate them instead of simply warehousing them in a state facility, that it would be a service to everybody, that we could provide much more vigorous short-term care as opposed to where you go out to the state hospital and there's less staffing and the care is more just custodial so to speak, okay.

Um, but some fraction of the people we would treat, say 20%, we couldn't resolve things in 20 some days, okay, and so we would need to move them on to the state hospital. Okay, so you got that. And then you've got a kind of group of people out there who are there for some longer period of time, and now you've got the sheriff in the jail who wants to get the mentally ill out of his jail because this was the guy's perspective was that he really wasn't in the bus- business of running a jail to take care of the mentally ill. It's an interesting argument, okay. It's very pertinent to what you're talking about.

So he went and managed to convince the legislature of his plight. And maybe six or seven years ago, the legislature in order to help him out passed what was called the 48-hour rule, an arrangement whereby (laughs) if the sheriff raised his hand, he could lateral a mentally ill patient out to the state hospital and the front door was compelled legislatively to open and accept those patients, okay. Um, and to some extent, it helped him a bit, okay. What it also did was, um, made it impossible for the rest of those running inpatient psychiatric units to get people in to the state hospital when we needed to. Hence, an enormous tension, okay, uh, between the sheriffs, just get these crazy folk out of here, um, and I mean, he was a [inaudible]. He went to the legislature and got them (laughs) to pass a law that mandated that the front door of the state hospital open at his best anytime he just sort of said, you know, I've got somebody at … who's psychotic and I need to send them to you.

Have to also understand that we would normally take a number of those people out of the jail into our hospital, okay. But it's kind of an interesting example of the tensions in the system. Um, try to …

I: Yeah, that's a lot of interesting background but I'm hoping I can dig into …

P: Okay.

I: … um, with some of my next questions. Um, but to get us …

P: Right, you have other questions, right, yeah I'm just laughing, sorry.

I: Yeah. So, um …

P: You have to cut me short I can, yeah, I can wander on forever.

I: Well, my next question, um …

P: So let me try to come back to your original point for a minute.

I: Yeah.

P: Um, I think there remain lots of things in the criminal justice system at least as I have experienced it as a psychiatrist that could be enormously improved upon. Um, and also not to say about more broadly, the national level things are going on about, yeah, so.

I: Right. So next, um, I wanna dive in to some criminal justice system terminology, so could you explain to me what comes to mind when you hear the following terms and I have a few that I'm gonna go through …

P: Free association.

I: Um, and the first is prison.

P: Prison, um, place you don't wanna be is what comes to mind for me. Um, I've been in a number of them. I've seen several prisons in the state and, uh, frankly, they're (laughs) scary might be a, a … and even the ones that are … I mean, I- I've also seen them at times when they were pretty unpleasant physically. I think they're better in some ways now, but, um, I don't have much in the way of positive connotations about the term prison, um.

I: What about the term jail?

P: Well, probably I'm biased in terms of thinking about the [county jail] which I have some familiarity with and you know, I think of it more as a holding area as opposed to longer term incarceration facility. So I think of it as a short-term facility.

I: Okay.

P: Um, still pretty stark, and funny things continue to go on over there … this is probably some of my bias. I mean, um, historically we got an issue about a number of people killing themselves in jail, in the [county jail]. Recent issue where a guy managed to escape from the jail. Um, did you see that?

I: Mm-hmm (affirmative).

P: Yeah, it was an acute event. Um, I mean there's also a whole issue about and we've had it in the hospital too and this probably shapes part of my response. Um, in any psychiatric institution, there are problems with security, um, and staffing, um, and we in this hospital had a number of problems for several years about the security personnel. And their problem with kind of overdoing it, being a little too aggressive, um, which also unfortunately resulted in a number of injuries to patients, um, and even a couple of deaths, uh, where people were actually asphyxiated in holding area seclusion rooms of the hospital because they were violent and then the restraint process, um, yeah.

I: And what …

P: Two, two people were actually smothered to death. So, (laughs) I know I'm probably way off field again in terms of your question but, um, so yeah, I just have a probably, you know, I'm way down the track. I've had a lot of experiences and a lot of them frankly haven't been very good. But there, there is a problem and it continues presently. I mean, we have it over at [community health care clinic] right now about how do you provide adequate security, uh, in a medical care facility, um, without getting into issues of security personnel being abusive or punitive or overdoing it. And that's a very common theme, whether it's in the jail, in the [psychiatric hospital] or even in the com- inner city community clinic.

I: Mm-hmm (affirmative).

P: So it was real, real problems because security tends to attract people, um, who kind of enjoy exercising power and control over others and sometimes they carry it too far so then an enormous tension.

I: So my next term is probation. So what comes to mind when you hear that?

P: Um, I guess I don't have all that much experience with probation. I mean, um, I don't know exactly what my associations with that are, um, in, um, from the psychiatric perspective, probably the more pertinent thing that's parallel is called a stay of commitment, where somebody who's under a commitment proceeding is deemed to have improved well enough to be released back into the community with a proviso and the proviso is that their case manager or some other person who's directly involved in their management and care has the authority to revoke the provisional discharge. So that's a sort of form of psychiatric probation, which I guess I'm a little more conversant with.

I: Mm-hmm (affirmative).

P: And it, it- it's an interesting problem because oft times the judgment about whether somebody is ready to be provisionally discharged in the psychiatric system, um, is questionable. Um, and I mean, I presume in some ways in the criminal system the same issues come up about who's eligible for probation and who qualifies and under what terms. The other problem that probably is different between the two is the willingness to revoke a provisional discharge in the psychiatric system is there's no consistency. It's widely variable. Um, it's left very much to the individual judgment of the case manager. And sometimes the participating clinician will just make a judgment or authoritative decision.

But, um, and the issues in some ways are the same in the sense the risk about putting somebody back out there is the idea that they can reoffend or that they can hurt somebody and that's sort of what you're trying to figure out. I mean, it also maybe about their … from the psychiatric perspective about the issue or are they gonna hurt themselves, okay. So I mean, a little different in that regard. I mean, with probation, um, and people who've been I guess incarcerated for felonies and assaultive kinds of things, the issue is, are they likely to reengage and hurt other people. From the psychiatric perspective, we have that. And you also have the issue about self-injurious behavior which is a more common theme. Um, so I don't … that's yeah, probation.

I: And then how about parole?

P: (drumming on table) Probably fuzzy on that.

I: Mm-hmm (affirmative).

P: Um, I don't … I'm hard-pressed to think of a lot of people that I've seen were on parole. I mean I think, I think more people who have, um, to check in with their probation officer, um, on a regular basis and have usually a finite period of time, um, during which, you know, they’re got to comply with all that or they get yoked. So I guess in my mind I don't make a big distinction between parole and probation but I mean technically I'm sure there is one. I just couldn't tell you.

I: Mm-hmm (affirmative). So next I'd like to dive into your background in education and training, um, to learn a bit more about if there were … was any, um, formal or informal training that was provided to you during medical school on working with criminal justice involved patients at all?

P: Well, I'm sorry once again. I'm gonna be a little complicated. Um, so when I went to medical school, um, I took a part of my senior year and took law school classes. I was at the [University] and so again I was very interested in the interface between law and psychiatry and so, um, I took a criminal justice course at the [University Law School]. So my classmates didn't have that experience. It, it wasn't, um, routine but I spent a good part of the last year of medical school at the law school, um, with, uh, somewhat a renowned criminal justice lawyer and professor named [professor name], um, and to leap ahead, but you wanna just know about medical school. Um …

I: Well, I am planning to ask …

P: Okay.

I: … about residency and …

P: Yeah, well …

I: Yeah.

P: The other thing in medical school that, uh, we were taken, um, to state hospitals, um, as part of the routine medical student experience. Um, don't remember being taken to a forensic, um, facility but we went to state hospitals to kinda get some idea, um, about how they work. And let's see. That's a long time ago. You know, I think more of it was my own interests rather than anything that the curriculum explicitly provided. Um, yeah, that's all I'd say about that.

I: Yeah, and so was there any training that was provided during your residency program?

P: Um, yeah, we had, we had some courses about, um, where more under the category of law and psychiatry and we had lectures about stuff like that. And again, I, uh, kind of went a little further. I was trained at [teaching hospital] in [city name]. It's one of the [University] hospitals. And, um, my last year of residency was predominantly spent at [University Law School] and, I maybe you know the name, [American lawyer].

I: I don’t think so.

P: He's, he's kind of a … He was boy wonder at [University Law School] and he's kind of a talking head on TV. Um, and he, um, okay, he has two claims to fame. He was the youngest full professor in the history of [University Law School]. He was a full professor at age 29. He's an inordinately bright and articulate guy. Um, and so he taught a law and psychiatry course at [University] conjointly with the psychiatrist named [name] who'd been the president of the American Psychiatric Association. So, you know, small potatoes guy.

The two of them conjointly taught an elective at the law school called law and psychiatry and it went through, um, a lot of very basic stuff about law and psychiatry, um, about competence, decisions and insanity rulings and so on, and up to much more progressive decisions that had a bearing on mental health issues, uh, particularly out of the court of appeals in Washington. There's a very famous judge named Bazelon, David Bazelon, who at the time was still on the bench, anyway.

These two guys would conjointly teach and argue with each other and, um, [American lawyer] just tied [psychiatry professor] up in knots. (laughs) That was sort of the end of my interest in law and psychiatry. It, it was really … anyway, yeah. [American lawyer] is still around. Unfortunately he's kinda gone to the dark side here. He always, um, had a pension for getting involved in high publicity things. There was a very famous guy named [name], who was accused of murdering his wife and [American lawyer] somehow got involved in his defense when most people thought he really killed her. [American lawyer] was there as his advocate. He just is a publicity hound and he's an egomaniac, just, you know, loves all the stuff.

But anyway, um, so yeah, residency, last year of residency I spent … I took some basic courses in the law school, some first year courses towards some constitutional law and then I took this law and psychiatry. So yeah, in the course of my residency, I had probably more exposure to law and psychiatry, at least in the academic abstracts but, um, no, I didn't spend a lot of time in the courts at that point, um.

I: And then did you complete a fellowship as part of your training?

P: No, I never had a fellowship, um, (laughs) it's interesting. Um, no I went right out of residency into the navy for a couple of years. I was a navy doctor in Vietnam. Um, I served at Disney World. Yeah. I'm serious. I was in Orlando, which is the navy's east coast training facility for two years during the Vietnam War.

I: Yes, so thinking back to your past or current places of employment, has there been any training around working with justice involved populations at all?

P: Well, a little bit. Um, I mean when I … after the navy, I came to Minnesota, to the university proper and, uh, I'm trying to think what … My work at the university was predominantly with medical-surgical patients and psychiatric care of people, you know, actually on the in-patient medical-surgical units. So the legal stuff didn't come up a lot. Um, it came up in terms of issues about competency. I’d often be asked about people's ability to make an informed and knowledgeable decision about consenting to some treatment or surgical intervention, um, often involved with people who were cognitively impaired, who had brain injury or were delirious from drugs or infection or something else like that.

But, um, in the beginning no, there wasn't a whole lot, um, involvement with criminal justice system, the first many years I was over there. Um, I’m trying to think. So after 16 or, yeah, 16 years over there, I came over here and there's a lot more about the court system and the law because we were routinely committing people. And in the first several years psychiatrists would have to go to court around commitments and testify and that just got to be too burdensome and then they moved it to kind of, um, Skype type things where you could just be online and testify. And then finally, they just accepted kind of electronic reports rather, you know, because it's very disruptive. I mean it's sort of like you're working in an inpatient unit and you're suddenly supposed to be at court at 11:00 for an indefinite period of time and all the vagaries of what happens in court, and it doesn’t ever run methodically and so on. So yeah, that, that all gave way.

Um, we did have, um, over the years a number of different interactions. Um, there were … from time to time there would be a judge who was particularly interested in mental health issues and eventually it led to things like the mental health court in [County] that was established, um, by a judge with a particular passion about trying to get the mentally ill out of the primary judicial system and creating an explicit setting for them in the sense that they get a fair shake in a mental health court than they would in kind of routine court.

Um, a secondary issue came up about training the police. Um, it was largely precipitated by an event and say it's probably in late '80s but I could be wrong, where, um, a woman in [local city], uh, was in an apartment building. I think if I remember this correctly, she was in the landing of the building naked wielding a knife. And the police were called. And they came in and killed her. And that precipitated an enormous response from the community, um, and led to a number of changes. One of them was the idea about trying to routinely train any number of police officers, um, about mental illness and I mean, it was really an issue about, you know, there got to be a better way than shooting somebody to control somebody who's aggressive and so on.

Um, and that theme of course has persisted. I mean (laughs) it goes on, you know, takes some different guises but it's always the same thing about, um, police being, uh, impulsive and, um, too quick to act and, um, I'm sure you're aware of all the problems in the metro area in the last several years with people being shot by the police under various circumstances.

Um, so the hospital took … this goes back probably 15 years, tried to routinely provide classes to educate the police about some basics about the mentally ill so that they wouldn't be as quick to respond with force and at least in my recollection, um, a lot of it was triggered by the event where this woman in [local city], who was white, by the way, um, and they just … she was wielding a knife true, okay, and they just killed her and people went bonkers about it. Um, that's a long time before the new rash of police issues.

So yeah, the hospital, um, and our department in particular conducted routine training sessions for the police. I think they still go on by the way. Um, so once again I've strayed off topic, um.

I: Well, I'm thinking I'd like to learn a bit more about your day-to-day interactions with your patients. Um, do you talk about or ask patients about whether or not they are or have been involved with the justice system at all during visits?

P: You mean presently?

I: Mm-hmm (affirmative).

P: Yeah, I do. Um, it doesn't necessarily bear a lot of fruit. Um, so over at [community health care clinic], um, a number of the people I see have, um, problems with the law and are embroiled in different kinds of issues, mostly, um, I mean and I guess there's two parts to it. There's people who've been arrested for various offenses, um, and then the second is people who are brought to court usually because of disputes with family members and, you know, custody issues or what I call as estate wars, you know, about fighting about inheritance issues or, yeah, custody, um, support payments, yeah, ok, um.

And then I have a number of people I see over at [community health care clinic], yeah, who are invariably in court because they've stolen something or they've assaulted somebody and yeah, I try to talk to them about those things because it's like the currency of what's happening with them and you can't really make reasonable decisions about even medicating them in terms of helping them cope more effectively unless you understand kind of what's on their plate and what they're dealing with.

Um, so I … and I, I try to be reasonably methodical about that, about, um, each time I write a note, uh, I kind of report on what they've told me about court dates, outcomes, what the issue is, kind of where they are, so yeah, I try to talk to my patients about it. Some of them are very forthcoming and others, it's like pulling teeth. You just can't get a straight story about those things and then at that point, you know, a lot of these people come with case managers who often are able to fill in the gaps and provide the information that the patient, uh, either chooses not to share.

And in some ways, it is an uphill battle because a lot of people don't like to talk about what they're charged with and what they've done. Uh, they don't necessarily see that that's some critical factor in a judgment about how you're gonna medicate them or what you're gonna recommend. But, uh, I'm always interested in information that helps me, you know, kind of fill out the picture, um.

I: Could you provide an example or like maybe like an individual patient where knowing this information has impacted how you approach their treatment plan?

P: Boy, that's a good question. Um, well maybe, maybe I can give you yesterday's example of where, um, it's not a judicial thing, no. Um (long pause) okay, um, I have a woman that I've been treating for probably on and off for two years who is bipolar and when she gets manic often, goes out of control and, um, has all kinds of restraining orders that have been issued against her. Um, and she's violated them and so she goes back to court for that. And at the same time, she's embroiled in a very nasty divorce action in which her husband, um, tried to, uh, take the children away from her. And probably understandably in the sense of he was very apprehensive about, um, her potential to go out of control and what influence it would have on the kids.

And so he, um, literally locked her out of the house, changed the locks, um, told the two children not to have anything to do with her and she in response has inst-, you know, got an attorney and has instituted all sorts of actions, um, to try to have some of their conjoint funds and capital and assets shifted over to her. And she in fairness has had a hell of a time doing that. Um, partly because the husband flogs her with being mentally ill. You know, she's a crazy woman and uh, you wouldn't do this. Okay.

And so she's constantly in court, um, trying to get access to her children, trying to get some of their conjoint equity, um, and, um, trying to kind of reestablish herself, and so each time she comes, there's a whole litany of what her legal actions have been. Um, and yeah, she also, I, this is kind of goofy, what she has been preoccupied with for sometime is getting her record purged so that it doesn't reflect any of her diagnoses of her mental illness because, and I think she right to some extent, what happens is those become weapons that her ex-husband or about to be ex-husband flogs her with and gets the upper hand in judicial actions.

Um, and when she's not manic she's a very reasonable woman, um, who's educated and actually is a teacher. Um, but when she's manic she’s just hell on wheels, you know, and so yeah, a lot … and all my documents wind up in court. And so, um, she and I have had an interesting go around where she wants the diagnosis bipolar purged from all her medical records. I said, you, you just can't do that. You can't go back and erase. “Well then you should write out, you know, that it's corrected or changed.” I said, you can debate whether it's the correct diagnosis and frankly, it is the right diagnosis but she doesn't wanna acknowledge it, so, um. I don't know if that's much of an answer to your question but that's an interesting case.

I: Mm-hmm (affirmative).

P: And you know, um, yeah, the things that you encounter as a psychiatrist you can't make this stuff up, I mean, the things. The corners people get themselves into and some that happens, so. Let's push on.

I: All right, so coming up to 12:29, right at the …

P: Yeah, I'm okay with the time.

I: Okay.

P: I just … I have a colleague in the rehab unit I need to go see.

I: Okay. Um, so my … I wanted to ask next, are there any benefits that you see to asking the patients about their justice system involvement?

P: Sure. I mean, you, you have to understand what they're wrestling with. You also have to understand to what extent their perception is that they've been badly treated, that they've got an axe to grind, that they've been abused or discriminated against, which in fairness, uh, happens more often than it should.

Um, so I was trying to think I had an interesting example, um, oh, I know. A friend and I were talking about, um, there was somebody in the news recently who committed suicide and, um, I was quite interested in how the person committed suicide and my friend says, well, what the hell difference does it make, you know? Gee, I thought that was pretty important information to understand. So we argued back and forth about why would it matter, you know? So I always think the more information you have, the greater the likelihood that you'll actually discern with some accuracy what's going on. You know, if you're left to just rudimentary information, you're gonna not necessarily come to the correct conclusion.

Um, I can tell one story and myself to kind of point this out. Um, for a couple of years, I've been treating a, um, a Hmong woman, um, who has 12 children. And in the beginning … and she's got a refractory depression that just hasn't responded to much of anything. Um, I'd see her with the help of an interpreter. She speaks no English. I don’t speak any Hmong. Um, her daughter always comes with her and her daughter is, um, was raised in this country and is very Americanized. Um, and her daughter generally sits in the corner and smiles and doesn't say anything. Um, and I've been trying unsuccessfully for quite some time to resolve this lady's depression.

And what I learned early on was that her depression dates to the time in her language that her husband left her, okay. And this is an example of not having the pertinent information. Um, so I assumed that what that meant was that he simply departed, okay. And I unfortunately never chased that piece of information. About a year and half goes by and we come to the anniversary of his departure, I mean, ok, and she's, um, more despondent than ever. And this time, um, she emphasizes again about his leaving. And so I said, "Why don't you explain that to me? What do you mean he left?"

At which point, the daughter, not the patient, and the daughter has been through all the sessions, okay. The daughter says to me, well, what actually happened is he killed himself after an event in which she, the daughter, brought home a classmate, um, and they were both, I don't know, maybe 11 or 12 or something, okay, and apparently the father in some fashion, um, made some sort of sexual advance to the girlfriend, okay and the girl went home, told her parents and the police came and interviewed him or talked to him. And when they left, he shot himself and killed himself.

So here's a whole powerful matrix of what has triggered this woman's depression, okay. I didn't have the correct information. I mean, okay. And so the issue is beyond medication. She needs a therapist to talk to her about the baggage that she's carrying and what I eventually understood is she believed that the husband actually did assault the daughter's friend. The daughter is sitting there for all these session never mentions it, okay. It's sort of like having your hands tied behind your back. I mean, yeah, medications are helpful in treating depression, but in the best of all worlds, you get a combination of some talk therapy and medication in this instance. And it's just a little example about where you don't have the right gestalt, you're not gonna be able to solve the problem.

I: Mm-hmm (affirmative). And then on the flipside, are there any challenges that you see to asking the patients about this?

P: Yeah, people, um, are often unwilling or uncomfortable discussing matters that are highly emotionally charged. I mean, that's … I mean what they wanna do as this lady did is just kind of, you know, box it up and put it away. Um, there's a whole theory about posttraumatic stress disorder, I don’t know if you ever heard this? Um, that if you take somebody who's just been through a trauma acutely and you medicate them liberally with a drug called propranolol, it's a beta blocker, that you can enormously reduce the subsequent incidents of posttraumatic stress disorder and the succinct explanation is that somehow the beta adrenergic blocker precludes the encoding of the memory.

And so, um, the idea is we used to think that the thing to do in the wake of an acute trauma was to have people verbalize as much as possible about it. To the contrary now people think that's not a clever idea and the analogy is that it's like you wanna put the trauma in little box, close it up and put it up on the shelf. And then if you wanna take it down subsequently and open it up, you do that with the help of medication. It's different over the years, different ideas about what trauma and how you acutely manage it. So it's an interesting problem about, um, people who make it very clear to you if they don't wanna talk about something. I mean, find interesting ways to deflect your questions and go someplace else.

Um, on the other hand, there are some people I mean who really wanna, you know, ventilate and try to get it out and the belief for a long time has always been that ventilating is cathartic, you know, and it's actually reconstructive and a helpful thing for you. So it’s an interesting kind of balance. Yeah, there are downsides. And people are usually signaled or distressed. I mean, you have to be respectful about when that comes up, although it's a tricky business. I mean, if you wanna get to really what the heart of the problem is, you know, sometimes you have to push a little bit but they'll tell you if you're overreaching usually. And you just have to be patient sometimes it, it takes … you know, there's issues about relationship and trust and time, so.

I: Could you tell me a little bit more about your overall patient population that you currently see, just some general overall characteristics?

P: Um, well, the majority of people that I see at [community health care clinic] I guess the majority are Somali. Um, and they fall into interesting groups. Um, the men that I see are generally suffering from psychotic illness, a major psychotic illness or they, they wouldn't be in clinic. Um, and they're usually brought by family or um, community members, you know, because their behavior has been increasingly unacceptable as a byproduct of their delusional or disturbed thinking.

So the majority of men I see, uh, have an ongoing psychotic illness process and the task is really try to manage them with anti-psychotic medications in order that they can, um, remain outside of the hospital and in the community. The women I see from the Somali community are, um, almost invariably depressed. Um, and they're inclined to somatize. They convert, they convert their psychological issues into physical symptomatology because that's acceptable.

I mean, it's acceptable to complain about physical dysfunction but it's not very acceptable to complain about your husband's disinterest or the frustrations you're having with your children who are angry about their parents and you know, I mean, it's like the younger generation is increasingly Americanized so to speak and the first generation immigrants, um, you know, majority of them don't speak English. Um, it's, it's much harder adjustment to give up the old ways, you know, if you're in your 50s and 60s and come to a new country with a new language and a whole different cultural matrix. It's very hard. The kids, you know, it's like technology. I mean, the kids pick it up very quickly and the old folks struggle enormously with it.

So, um, yeah, um, and the other thing that's different over at [community health care clinic] than here is, um, a lot of what people need are very basic social service and programmatic offerings and benefits. They need assistance with housing, assistance with healthcare coverage, um, food stamps, case management and so on. So it's enormous amount of time directed to enrolling people in different programmatic services and sometimes trying to determine if they're eligible for disability benefits and most of the guys with the major psychotic illnesses are disabled and entitled to benefits.

Um, there's also all kinds of issues around immigration status, um, green cards and applications for citizenship and all the documentation that goes into that. So that's the Somali population. Along the way, there's, um, some Native Americans. There are, um, some Asian, a number of Latino patients, um, and a small number of African Americans as well, so, it's quite a mixture.

I: Mm-hmm (affirmative). And then thinking specifically about patients with some type of justice system involvement that you're currently treating, could you tell me a bit more about that … what that experience is like for you with those patients?

P: It's frustrating generally because, um, it's not like … right, here on the inpatient service, when you’d have people who are in the criminal justice system, you would have the records in front of you. I mean, the … in the inpatient unit, you get a hold of the commitment orders, um, and there's a lot of clarity about, um, what they've done, you know, what they've been charged with or what the terms of their commitment are and what the directives from the court are.

Um, over at [community health care clinic], we don't as easily have access to those documents. Now in fairness, if the patient has been hospitalized here, it's much easier, okay. I mean, you can get those records. But generally, we rely on the case managers to tell us what's going on in the judicial system with the patient about, you know, they usually go to court with the patient and depending on how good or how vocal the case managers are, you get some clarity, sometimes no clarity, um, about where the judge is or kinda what's going on.

The usual process is the patients come in and will tell you, they have a court date six weeks from now. And then you say, well, what's the court date about? And it gets fuzzy at that point. Okay, um, and eventually, um, you know, maybe you'll find out that they've been charged with a, b, and c, but it's, it's not like you have a document that says arrested or arraigned with this pending charge and you know.

So it- it's a struggle, um, in the community clinic setting to get a lot of clarity and um, again, in the hospital, you have social workers who interact regularly with the criminal justice system, know who to call to get the documents they need and get the clarifications about when is the hearing, what are the charges and so on. Um, out in the community clinic it's, it's harder. It's much harder to get the clarity about that.

And the other thing that happens is court dates always get postponed or delayed where they happen. You don't really understand what transpired in the court. I mean, there's an information problem there.

I: Mm-hmm (affirmative).

P: So, we don't … the patients unfortunately are not always brilliantly reliable historians particularly when it comes to matters of what did the court decide and what are the terms. I mean, all these issues about custody and divorce and support payments is very hard. And it's even harder when people who've been arrested for other things like terroristic threats, um, you know, generally ‘cause they're psychotic and they've made terroristic threats, well, you're not gonna have much of a discussion with somebody in that group, um, about what they said or, you know, what the result of the judge is, um, 'cause their perception isn’t terribly reliable. I don't know, does that help you at all?

I: Yeah, I wanted to follow up about, um, you mentioned, um, wanting to know the charges, so how does … how does having information about that impact how you, um, approach a treatment plan for a patient?

P: Well, you got to, you got to know what the ballgame is. I mean, um, if the guy has been arrested simply for possession of drugs, that's one deal, but if he's assaulted somebody and you know, you need to know how and to what extent and, um, it's like flying blind if you don't know what the patient is actually being at court for.

So and as I say, the problem is sometimes you don't get a very accurate representation either of what the reason for being in court is or what the outcome of it is. And you know, we get a kind of couple weeks later some downstream thing. I mean, they occasionally … oh, I'll give you a very complicated one.

I have one patient that I followed for about three years who's a Somali male who was a trucker, long distance driver for trucking company. Um, and he was in LA with an 18-wheeler and he got out of the cab and he got hit by an 18-wheeler going the other way. And he had severe injuries. He had, um, massive brain trauma that required multiple neurosurgical interventions, left him with a skull deficit, um, and significant cognitive impairment. He had been fluent in English but after the event, he kind of lost his capacity for English. He's cognitively quite limited.

Um, and very complicated business about the trucking company of course had … he had a workman's compensation policy. Um, and so the trucking company, um, absorbed all the costs associated with his medical care. Um, and then a number of people came forward because in the beginning he was incompetent and there was a distant family member who was interested in kind of litigating on his behalf, um, and I got peppered with requests from different attorneys, um, about did I think he was capable of making a decision, um, about his care, and kind of interesting example.

Um, I was very loathed to get involved in responding to inquiring attorneys particularly when I didn't understand who they were and whose interest they actually represented. It took a long time and eventually, um, I heard from a woman representing the trucking company in [city name]. The trucking company was based in Colorado, so he was covered under a Colorado workman compensation act, um, and her question to me was about the possibility two years later of his returning to some form of work.

Now, um, (laughs) so I wrote back and we had kind of an interesting exchange in which I said to her, "Look, um, at this point, I don't have the benefit of understanding exactly, um, what benefits he actually has under your firm's coverage. And before I say anything about, um, what I think about the idea of his working, I think you oughta clarify for me essentially what the arrangement is.” Ok, this is like a year and a half later. She writes me and tells me also about somebody here, a cousin of his, who's got his medical power of attorney and I her wrote back, I said, I've never seen this person in all this time, okay, the other thing is this guy slowly got better.

I mean, he's not great but, okay, he's, um, I mean, he was for three months in the beginning, he was completely delirious and he was really a wild man. And now, now, he's just, you know, he's very civil and he's, you know, I mean he's cognitively impaired but it … he's improved, enough so that he's fired pretty much everybody. (laughs)

And the couple of distant family members who tried to capitalize on his unfortunate situation have been banished. But anyway, I had this whole correspondence and that ultimately comes down to the question about what do I think about he's going to work and I said, "I'd like to understand what the implication of his going to work in any capacity has to do with his long-term benefits." I’m paranoid because what I'm thinking is they're trying to figure out how they can reduce their burden over time if he could work somewhat, then it's going to lessen the idea of what their obligation is.

Well, as soon as I wrote that back up, never heard from her again. And then he started with a therapist a number of weeks ago. Damned if she didn't send the same set of forms to the therapist who was smart enough to circle ‘em around, okay. But so I mean, there is a whole bunch of legal complexities and it's, it’s about money. It's about what are the benefits that he's guaranteed under his workman comp and she said, "Well, you know, well, this is just temporary work and you know, and it could affect his temporary benefits.” My question was what about his long-term benefits. That's what really matters, not his temporary benefits.

So, um, you know, I have a daughter who's an attorney, um, a litigator with a big firm in New York so (laughs) and my father was a litigator so I'm a little sensitive to legal issues but there’s kind of a very complicated example. And this is going on for 18 months, okay, about who's really running the ship here and what are they obligated to provide him and are they really trying to crap out on it, which is my interpretation, so.

I: So how do you think justice system involvement may have impacted your patients' ability to access and receive healthcare?

P: (long pause) Well, so maybe now you're talking about the few patients I've seen who were actually on parole or probation. Um, okay, here's another thing we haven't talked about at all but I'll just mention. Um, the workhouse up in [city name], so a lot of our patients, um, would short term spend time up in the workhouse and we also provided psychiatric support in the workhouse but the same issue came up is that we couldn't give them enough time and effort to deal.

And when you go into any correctional facility there is an enormous amount of mental illness, um, I have this patient, a psychiatrist who worked in the corrections institute in Iowa for several years and I mean, he would tell me horror stories about the things that went on in the correctional facility. Um, so I have an impression, a little bit more second hand than first hand that, um, correctional facilities do not necessarily render brilliant medical and psychiatric care to the patients involved, um, partly 'cause it's ofttimes hard to find people who are willing to work in those settings. Um, even though sometimes the renumeration’s getting better. Um, not everybody’s thrilled about working in a correctional setting. So I think people get shortchanged, but you have to understand a larger bias.

The mentally ill get shortchanged in the medical care delivery system because they frighten people and so the majority of medical providers are uncomfortable enough with the mentally ill that the quality of medical care that the mentally ill receive is lousy, okay. It takes a relatively unusual medical provider to be willing to engage a mentally ill patient in a vigorous and full way. And there's actually literature about how the mentally ill gets shortchanged medically in terms of their care. And it's even operative in this hospital. Um, a lot of medical providers want nothing to do with mentally ill people and some of them will actually say things like, “I didn't go to medical school to do this shit,” okay, so they just sign off.

And I, I have, um, a kind of rule which is 20% of medical providers are psychologically minded and sensitive to psychological and psychiatric issues. For 20%, an [inaudible]. They want absolutely nothing to do with mental illness or psychological issues. And then you've got the group in the middle and they slide depending on recent experiences, who it is they're interfacing with from the psychiatric side, okay, and like the unwashed in the middle.

And so if you're effective, you can sometimes pull more of those people, but you have to understand the basic notion that only a small fraction of people are gonna be terribly interested or really psychologically minded enough to engage somebody with mental illness and in equal number. And in terms of a criminal justice population and medical providers, I would purport that the same thing is true. There's a fraction of medical providers that want nothing to do with criminal justice people, like yeah, I don't wanna be there.

And then there's a, a group of people who I think are really service oriented or empathic for a variety of different reasons. And then there's a group in the middle that depending on the tone of the environment and where the leadership is directing things can kind of be pushed and pulled one way or the other.

I: And so thinking about this interaction between mental illness and the criminal justice system, in your point of view, what would you, uh, suggest that we change about healthcare delivery and how we're delivering care to folks, um, to better needs of folks that are wrapped up in the criminal justice system?

P: Well, um, we should probably wrap up in a couple minutes. Um, the thing that mentally ill people need the most is structure, and particularly housing. I mean, if I could redesign any state budget for the mentally ill I'd push an enormous fraction of it into structured housing settings in which they are safe and have some, however, modest degree of oversight that heightens the likelihood that they’ll take their medications because the biggest thing to staying out of the hospital if you're mentally ill is medication compliance.

So I want medication compliance, okay, housing, I mean, we have a ridiculous problem with homelessness, the entire country does, okay. Um, and when you provide a structured setting and some support, you heighten the likelihood that people stay on their medications, the likelihood that they're gonna do better is greatly increased when they stay on their medications, because without them, in a matter of time, they're gonna decompensate and wind up back in the hospital again.

So the kindest things in the mentally ill system and I don't think it's a lot different is housing, and some structure, you know, and obviously some sort of support system. I don't know that that's different for the mentally ill, or the people in the criminal justice system. Um, one sad note. One of my former trainees wound up in prison. He had what are called boundary problems. Do you know what I mean by that?

I: If you can elaborate a little bit, I think I …

P: Well, he, uh, had a relationship with a patient and yeah, he wound up going to prison for some period of time. He wound up losing of course his medical license. And when he came out, um, he's independently wealthy, so earning an income wasn't a big thing, but what he chose to do was to get involved with groups that try to help get people who come out of prison back into the world on track, both in terms of housing and jobs and so on and so he devotes a lot of his time for those kind of activities. Talking to him about his experience in prison was (laughs), he spent three years, um, in a couple of different prisons here in Minnesota um, for really, um, a sexual crime. Um, and yeah, what he talks about a lot is how tough it is for people coming out to get back in the world. It's hard to get a job. Um, it's hard to find housing, umm, and it's hard to have much of a support network.

So I don't know that those things are dramatically different, I'm extrapolating from my general ideas about the medically, uh, mentally ill are just badly mistreated in our system. Um, they really are second class citizens and I have the opinion that people coming out of jail are in the same category. And remember that a sizable fraction are the same people, okay, because yeah, they're … any, any estimate of the extent of mental illness in the jail and/or prison population is pretty high. So you got a big bridge to cross here.

I: Well, thanks again for your time.

P: Okay. I hope some of my babbling was helpful to you.

I: Yeah, I'll go ahead and …
