# Supplementary material for: Physicians’ perspectives on continuity of care for patients involved in the criminal justice system: A qualitative study
Source: PLoS One. 2021 Jul 14;16(7):e0254578. doi: 10.1371/journal.pone.0254578 (PMC8279398; doi:10.1371/journal.pone.0254578)
Supplement: S2 File — (ZIP) [file pone.0254578.s002.zip › Clean/Participant_16_Audio2_deidentified.docx]

I: So, thanks again for meeting with us today. Um, the goal of our interview is to again, understand what you know about the Criminal Justice System, and to hear more about your experiences, if you have any with treating patients that have some type of justice system involvement.

And I'd like to begin by just getting a general overview of um, what you know about the justice system, and so, could you tell me what you think about the current state of the Criminal Justice System in the United States?

P: That's a very good question. I honestly don't know a huge a lot about it. My experiences have either been through media, news, and then most recently last couple years have been with my patients that I do a lot of opiate addiction treatment. Um, I think my viewpoint of the justice system has been more negative of the recent years. Mostly what uh, my view of, I think racial justice and that ... the, the issues that I think people of color experience with the police in general, but I think my confusion and sometimes with the police and the justice system and how to separate that in my own mind. Um, I also, I think in the last few months with the Supreme Court and stuff like that. So the court system, justice system, and the police is all kind of a big blur to me.

And a lot of it, I honestly I think it's just been negative.

I: Mm-hmm (affirmative). And so next I'd like to discuss some Criminal Justice System terminology.

P: Mm-hmm (affirmative).

I: Um, could you explain to me what comes to mind when you hear the following terms? And I have a few that I'm gonna go through and the first is prison.

P: Uh, prison. I would say is something long term. Um, something uh, where people are separated from the rest of the society for whatever punishment they've done.

I: And what about the term jail?

P: Jail. I think of people who have done more like petty crimes, are in there for shorter periods.

I: See, and could you tell me a bit more about how you maybe distinguish between prison and jail?

P: Um, I don't honestly.

I: Mm-hmm (affirmative). And so, what comes to mind when you hear the term probation?

P: Um, something for someone that's not completely needs to be in jail or prison, and so they're watched closely with uh, an officer, um, so if they do something that is unlawful or big, break some sort of contract of theirs, they end up in prison or jail.

I: And then what comes to mind when you hear the term parole?

P: Parole. I'm assuming at that point they had been in jail or prison, and they received a condition where they could leave um, prison or jail for, for that period without again causing any problems and get, going back in.

I: Mm-hmm (affirmative). And then again, could you elaborate a little bit more on how you distinguish between probation and parole?

P: Uh, I think what I said was as far as I can probably go…

I: Mm-hmm (affirmative).

P: …In terms of definition.

I: Okay. And so next I'd like to shift into learning more about your background and education and training. Um, during medical school, did you ever receive any training on working with justice involved individuals, whether it was formal or informal?

P: Um, not much. I think during residency, I maybe had worked with one of the providers who did um, more of a ... with adolescence. So like, more from the juvenile standpoint.

I: Okay. And so just during ... that was during residency?

P: Mm-hmm (affirmative).

I: Okay. And, as part of your training, did you complete a fellowship?

P: No.

I: Okay. And so now thinking about your current or past place of employment, has there been any training provided to you on working with um, folks that are incarcerated or on supervision in the community ...

P: No.

I: …either on probation or parole?

P: No.

I: Okay. And so I'd like to um, shift now to your, thinking about your day to day visits with your patients. Do you ever ask if they are currently or have been involved with the justice system at all?

P: Yes.

I: Can you tell me more about how you ask that question?

P: Um, so all my patients who start treatment with buprenorphine for opiate um, addiction treatment. Um, on the first visit I ask right away. I may not use the word justice, overall.

I: Mm-hmm (affirmative).

P: I'd probably just say, "Have you um, been in jail or prison? Do you have a parole officer or you have been on probation?"

I: Okay. And then once you have that information, how does that inform your treatment plan for a patient?

P: Um, it let me know if I need to do certain things for that individual. Um, whether drug screens matter. If that is then reported to their parole officer. Um, it also lets me know what kind of um, support system they have. If they are recently out of jail, they might not have a strong network um, to help them, um, um navigate through um, their health.

I: And then, are there any benefits that you see in asking patients about this?

P: Um, I think for me, it helps with transparency, that they know that I will just get to ... I will just ask them frankly about all these questions as part of their social determinants of health. Um, that's, it's just a normal question for me to understand their, their life. And then um, often I, if I don't ask that, I won't ever know.

I: And then on the flip side, are there any challenges that you see to broaching this topic with your patients?

P: Yeah, it's clearly a sensitive topic. Uh, and when you're dealing with addiction in the first place, it is a stigma itself, and then to add another stigma on top of that, uh, I think patients always want to look the best and feel the best when they see a doctor, and so, to be able to reveal something that's um, hard like that, I think it makes them feel uncomfortable.

I: And so, could you tell me a bit more about your overall patient population and who you're seeing on a day to day basis?

P: Uh, so most recently in the last two years, with the opiate um, addiction treatment, it's probably been around 70% of my patients. Um, um, use um, either heroin or other forms of opiates. Um, the rest of the 25% um, are immigrant and refugees in general. Um, majority are probably people of color. Um, and then particularly with the heroin addiction, I have a lot of Native Americans um. There are a mixture of some white and um, some black patients too. Um, because I have more uh, opiate addiction treatments, I have less of um, and historically less of Myanmese and Asian patients too, and less of Somali patients.

I: And how would describe the income levels of the patients that you see?

P: Um, a good chunk of 'em are homeless. So, uh, the majority of them don't have much of an income at all, barely enough to have any food or any shelter.

I: And as a follow up to that, what types of insurance or, do they have access to health insurance coverage?

P: Uh, I think those who are savvy enough uh, do end up having insurance. Um, they're probably more insured compared to my patients who are the immigrants and refugees. Um, most of them who do have insurance would be on Medicaid.

I: And then how would you describe the disability status of the patients that you see?

P: Good question. I-I don't often ask that as much. Um, and then clearly, [inaudible] disability can be a lot of different things. If you're talkin' about emotional, mental versus something that's physical, too. I think most of 'em have mental health problems that's um, a comorbidity for sure. Physical disabilities, I-I other than me seeing that outwardly, I may not know. And then um, learning disabilities, I-I don't ask in general.

I: Okay. Let's see, and then you mentioned that the majority of your patients are people of color. Have you noticed any particular challenges or barriers that people of color that you're seeing are facing in terms of accessing healthcare?

P: Um, yes. I think uh, particularly the Native American community very, feel very marginalized. Um, there's a lot of distrust. Um, I, myself is not Native American, so that also is sometimes hard for them to want to feel open to me about it. I'm learning a lot more about the community. I think not knowing a lot about the culture makes it more difficult in terms of um, the words I use um, the things I ask.

Uh, I think in terms of the black community and the white community, that's a different experience. I-I feel more comfortable with that community, 'cause I've just worked with 'em more. Uh, I often do talk about race in my appointments, and I-I think I talk about it in a way that I can tell them I, I show a sense of empathy with they deal with from a day to day. Um, the fact that I have a hard time understanding the justice system, that I have a hard time understanding the healthcare system, um, I usually lend it, my lens in that way, saying that, "If I have a hard time, you must have a hard time too."

I: And, I now want to shift and focus on patients that have some type of justice system involvement that you're seeing, and could you tell me a bit about what that patient experience is like for you as a provider?

P: Ask that again.

I: Um, for patients that justice system involvement ...

P: Yeah.

I: Can you tell me what that patient experience is like for you as a provider?

P: Um, I think it sometimes it becomes a check mark to know that there's some sort of experience they've had. Um, for me, it's just an additional risk factor for them, so, when I find out if they're ... either they're homeless, um there's been domestic abuse, if they've been raped or if they've been in jail, or if they've had a partner in jail. I think it's just another way to have more insight of how hard their life is, and the fact that it would again tell me if they're network or their support system is strong or weak. Um, and then that would give me insight whether um, we would need more help with them in terms of therapy, psychiatry, or other support services they would need going forward.

I: And, do you ever get justice involved patients that are specifically referred to your care?

P: Um, I'm trying to recall if we've, we've had patients that come out of the county jail to us directly. There have been maybe some instances, but I-I don't know right off hand.

I: Okay. And, how do you think justice system involvement may have impacted your patients ability to access healthcare?

P: Um, the biggest thing for our patients is the lapse of uh, the buprenorphine. Um, a lot of the, the jails don't provide it. And so, once they're in that system, they're off that medication. Um, often I hear stories about patients still getting drugs through other ways, um, while they're still in jail, and then often when they are released, they have ... at that point, they're just eager to use again, and then I don't see them for a period of time. So, most of it is relapse in terms of my experience when they enter the jail system if they're already on buprenorphine.

I: Okay. Let's see, and then, are you communicating at all with parole officers or probation officers or the courts?

P: In general, no.

I: Okay.

P: Uh, once in a blue moon, a person would ask me for a letter or some sort of um, support. I think those are the individuals are at that point more savvy than others to ask for my support.

I: Okay. And then, aside from possible justice system involvement, what else are your justice involved patients dealing with socially?

P: Um, I-I think most of them, a lot of 'em don't have much income, and so, they either go back to finding ways to find that income. Uh, and so, whether it is stealing um, whether they are selling their body for, for money, or still doing, um, dealing drugs, and at the same time using um, drugs themselves. Um, and then that loops into, um the homelessness in general. Um, very rarely they are able to seek help in terms of getting more education or having tools to finding a, a job that is reliable, sustainable for them. Um, but I think mostly it's the homelessness has been the biggest issues for a lot of my patients.

I: And then, I want to circle back um, to your comments about buprenorphine and...

P: Mm-hmm (affirmative).

I: With not having access to that in jail. Could you tell me a bit more about the ... some of the health implications of not having that access and how that affects um, the patient's health?

P: Sure. Yeah. So, you know buprenorphine works in a way where it helps with um, physical dependence and withdrawal. So, if that medication is stopped, people will start experiencing withdrawal. So physically, they will experience flu-like symptoms. Um, so it could be fevers, chills, diarrhea, runny nose, and they would just feel miserable, and maybe at a point where they are so incoherent and not themselves, and maybe they're not even listening to those around them. That's just from a physical standpoint.

Emotionally, I think cravings will increase to a point where um, uh, they're just like out of their own skin. Um, often people who have this type of addiction don't do it 'cause of the euphoria that people think about, it's doing it because they don't want to have a withdrawal that's so bad, that hurts so bad. And that can last for a week or so. So that's just a miserable experience, in jail, just feeling tremendously sick uh, without the support of a medication like that.

I: Okay. And then, what does that ... so when they come back to you, what does that mean for you and how you then try to re-approach their treatment plan, and how does that, does that have any downstream effects once they're back in the community?

P: Uh, you know, I think a lot of my patients just know that's how it is.

I: Mm-hmm (affirmative).

P: They've, I think they've accepted that's part of the process. Um, so, either they know that they can get other drugs, so if they can get an opioid of some other sort in jail, that's what they'll do instead for the time being. Um, and I'm-I'm okay with that. Because they're just trying to survive at that point. Um, and I just appreciate that for them to even come back, because it is often a struggle when they are released, that they're first inclination, “I don't have suboxone or buprenorphine. The easiest thing I'm going to get off the street is heroin,” um rather than come to clinic, 'cause they might not be able to find me in clinic. Um, so, I just work them them, and we just move forward from there.

I: Thanks for those additional details. Um, I'd now like to dig into, what are you seeing patients with justice system involvement dealing with medically, aside from um, opiod use disorder?

P: Mm-hmm (affirmative). Um, yeah, it's not uncommon for-for patients to have diabetes or hypertension, uh or any of the other chronic issues.

I: Mm-hmm (affirmative).

P: Um, like particularly with depression, anxiety, or a whole slew of medical, uh, mental health issues too. Um, so I think ... I'm assuming with a jail system or any healthcare systems in general, a lot of medications get dropped off. They're, patients aren't aware of all their meds or all the names of their meds, and so, they might not be taking their other meds while they're in jail too. Um, and that, that happens for any healthcare system, so, if I sent a patient of mine, whether they're at [health system] or at the University system, they might not have access to um, what they're taking in clinic, and that is just disruptive for them too. So, if they're not able to take their diabetes med, medications or their depression medications, that causes a lot of problems too.

I: Let's see, and I know your realm of expertise is opioid use disorder ...

P: Mm-hmm (affirmative).

I: Are you seeing any other poly-substance use in conjunction with that?

P: Oh, for sure.

I: Yeah?

P: Yeah. Uh, it, meth is probably as big nowadays, so, and I think often people use meth if they can't find heroin. Um, benzos, um, marijuana. All, literally all my patients use marijuana, and in general, I think they use it because they're just so anxious, and it helps them calm themselves down. But, name the drug, they all use it. It's just a checklist for them.

I: Yeah. Okay. And, are there any resources or services um, that you wish were available to you to be able to refer your patients to, but you're just not finding, there's a gap?

P: Um, I think there are a lot of resources, but I think part of the problem is, it depends on the program and how it works for the individual. I-I don't think there's one program that fits for all individuals, and so, to understand that person's needs is really important. Um, so going back to like the Native American community, I think there's not a lot of programs are culturally aware of Native Americans, and so I can give them to a program that works for another person that's White and may feel um, they prefer a very private, very um, siloed experience versus a Native American want, may want a family experience or tribal experience. But, there's not a lot of programs that can do that. Um, or they've been through those programs, and they need to start anew with another program, and they've kind of exhausted everything like that.

Then the other part is the Rule of 25 um, has been I think, a deterrent for a lot of our patients. All my patients need treatment, and so, I just feel like a Rule of 25 is just um, this barrier for patients, that they need to have done in order to get any sort of treatment too. Um, so, I'm hoping things like that could be resolved, moved out of the way in the future.

I: Yeah. Could you elaborate a little bit more about that and the Rule of 25 specifically, and what are patients seeing as a barrier to that?

P: Yeah. So, you know, for me, what I understand with the Rule of 25, it's just literally assess their addiction needs and treatment needs.

I: Mm-hmm (affirmative).

P: Um, based on what county they're in. So, you know, I-I might have a patient come down from Red Lake, and they need a Rule of 25. I can't just ask them to do it down here. They have to go up to their county to do it. That barrier is huge, right? Or um, I might have a patient in the hospital, and I realize they have an addiction, and they need to get a Rule of 25. Very rarely I can get an assessor coming to the hospital to do it.

So instead, they sit in the hospital several, for several weeks getting IV antibiotics, can't get the Rule of 25, and I have to rely on them to get the Rule of 25 outside the hospital. Clearly, for them to get to that point, to get in the hospital where they're so medically sick, and they need IV antibiotics or even need surgery, they're not gonna maybe have the ability to do that. Somebody out of the blue, when I have not even really dived into their addiction treatment in the hospital, so they don't get it.

Or I have patients come into my clinic, and they want to get a Rule of 25, but I say, well, either you schedule it, and you have to drive and find transport to there, or you have to go wait in line and wait several hours at like, uh, Park Avenue in a hope to get interviewed that day to get the Rule of 25. Um, and then wait for several weeks for an answer until they get uh, treatment. So, it's just this ... the wheel spins so slowly for our patients, when I can see them, and they have that very desire, like, I need treatment now, I want that Rule of 25 now, but they won't get treatment for maybe two, three, four weeks, or even several, couple months.

I: Okay. Yeah. I wasn't aware that it took, that process took so long.

P: And maybe it's not that even the process, maybe it’s the patients that are not aware, and they're not even staying in touch, and maybe the paper's been filed, but they don't know the next step, so they don't have a phone number or they don't have an address, so they're just barriers of getting back to that person.

I: Mm-hmm (affirmative). Sure. And now thinking broadly, are there any changes to healthcare delivery that you would suggest to better meet the needs of folks that have some type of involvement with the criminal justice system?

P: Um, I think the biggest arching thing is first is, uh, healthcare for all, for everyone, access for everyone in general. Uh, two, removing things like the Rule of 25. It's a burden on our patients and the healthcare system. Um, three, providing drugs like buprenorphine in all jails. And a patient that's on a medication should stay on a medication while they're in jail. They should not be stopped at all. Um, and then the better communication, uh in general, uh, sharing records, communicating with providers. Um, me, I just need more education about the justice system. I, it's a black, blackhole to me, um, and that, understanding that would help me navigate our patient better or give them an idea of what they can do to navigate for themselves better.

I: Um, so thank you again for your time today. Before I officially wrap up, is there anything that I didn't ask you about today that you think is important to add?

P: Um, not offhand.

I: Okay. So, thanks again.
