# Supplementary material for: Physicians’ perspectives on continuity of care for patients involved in the criminal justice system: A qualitative study
Source: PLoS One. 2021 Jul 14;16(7):e0254578. doi: 10.1371/journal.pone.0254578 (PMC8279398; doi:10.1371/journal.pone.0254578)
Supplement: S2 File — (ZIP) [file pone.0254578.s002.zip › Clean/Participant_17_Audio1_deidentified.docx]

I: So thanks again for, um, participating in this study. Um, and the goal, like I've said, is to understand your perceptions about the criminal justice system and any experiences you've had with treating patients with justice system involvement and to start us off today I'd like to just get a general sense of what you know about the justice system and could you tell me a bit about what you think about the current state of criminal jus-, of the criminal justice system here in the Uni-, United States?

P: The current state. Well I think, um, it needs to be revised. I think there's probably far too many, um, individuals in the criminal justice system, um, at this time.

I: And then, next I'd like to discuss some criminal justice system terminology.

P: Mm-hmm (affirmative).

I: Um, could you explain to me what comes to mind when you hear the following terms and I'll go through several and the first is prison.

P: Prison is a place where people go when they have been sentenced for a crime that they've committed and in order to serve out that, uh, that sentence period.

I: And how about the term jail?

P: Jail is a similar, um, type of place where, uh, again people who have been accused of a crime and have been convicted or who are awaiting trial if they're, um, deemed to be dangerous and therefore held.

I: And could you tell me a little bit about how you distinguish between jail and prison?

P: In my mind, whether it's correct or not, I distinguish between jail and prison in that prison is kind of a place where people go more long term after they've been sentenced. Jail is, is a place, uh, where people are held, um, either awaiting, um, to be sent-, the sentencing period or, um, for, um, a lesser type of a, a crime.

I: And then what comes to mind when you hear the term probation?

P: Probation is a, uh, a period where an individual may have been convicted of a crime but has, uh, will be serving, um, some type of a sentence not incarcerated behind, um, behind bars.

I: And what comes to mind when you hear the term parole?

P: Parole, um, someone, again, who may be se-, who may have already been convicted of a crime and they're, they get to serve, um, the remaining length of their, of their, of their sentencing, um, not behind bars.

I: And, again, could you tell me a bit about how you distinguish between probation and parole?

P: Um, again, I think parole, in my mind, is attached to people who have been in prison and probation, again, kind of means to me that maybe that the crime wasn't as, as extensive or as serious.

I: And then next I'd like to shift to learn a bit more about your background and education and training. Um, during medical school, did you ever receive any training on working with justice involved individuals whether it was formal or informal?

P: Nope, never.

I: Do you think there would have been training that would've been helpful to you during that time?

P: Uh, I think so. I think training is, in general is never a, a bad idea. So it's, certainly I think that if I knew I was gonna be, be going into the career that I'm, that I'm currently in and, and have been in for the last, you know, 18 years, then yeah it would've been definitely helpful to me.

I: And then during residency, similarly, did you receive any training on working with justice involved individuals?

P: No. No, I experienced working with them given the environment in which I worked but we never had any formal training.

I: And then did-, as part of your training, did you complete a fellowship?

P: Did not, no.

I: So then you mentioned, um, you know experience with working, um, in your current job but in your current or if you had a past place of employment, have you received any on the job training on working with criminal justice involved individuals?

P: No. Only, only that we would be seeing those types of patients, um, that were in, in the system so to speak and some of the process, um, uh, processes involved whenever we actually were evaluating them in, in the environment in which I work.

I: Um, could you tell me a little bit more about that, um, in terms of involved with the system, are you meaning involved with the criminal justice system?

P: Involved with, um, people that are coming to us for medical care that are, that are in-, incarcerated.

I: Okay. And I, I think that's something that I think, um, hasn't come up, what do you mean by, I guess, coming to you from the criminal justice system?

P: So I, um, I work in, uh, the emergency department. I'm an emergency physician and so, um, while my understanding is there are some healthcare services provided in jail by jail nurses and sometimes phys-, physicians that, um, we'll see sometimes patients, um, in jail and also certainly in the prison system. Occasionally patients will, um, need an evaluation in, in the emergency department because of their, their illness or their injuries arer, um, beyond the scope of what, of the services that can be provided on site, either in jail or in, in prison. And they will come to our hospital. So that has, that's been the case of the hospital that I used to work at and the emergency department and also the one that I currently work at.

I: And then, so during visits, um, and, I guess you, like knowing your unique position in that there are patients coming from the jail and prison this may, um, may not be as applicable but in your day to day, um, aside from those types of patients are you asking about criminal justice system involvement?

P: That's a difficult question to answer. I, I don't necessarily think so. Um, no. I, I think whenever we, we see patients that are, it's pretty obvious to us when we, um, see patients that are brought in. They're accompanied by a sheriff's deputy or the police. Um, there are times, though, whenever maybe, uh, a patient has been the victim of, um, of a, an assault or some type of a, a crime that was perpetrated against them, that we, we ask the patient if there's been any involvement of the, the criminal justice system in, in general meaning the police. Does that answer your question?

I: Mm-hmm (affirmative), yeah. Let's see. And, um, wondering a bit more about this, um, for, um, patients that are coming from jail or prison, um, what that experience is like and how it may differ, I guess, from some of your other patients.

P: Well I think, again, whenever those types of patients are brought from jail or, or prison, um, it, they're easily, um, um, they're identifiable basically because they have guards with them. Um, and I think that there's probably, I won't say, I won't use the word stigma but there's probably some, um, special attention that, that are, that are paid to, to those patients when they come in and they're, and they're on their guard and there's usually a, um, decision at some point whenever you're evaluating a patient where you have to determine, just like any other patient in the emergency department, you have to decide whether or not the patient will need to be admitted to the hospital or can be discharged back to jail or prison. And so that's actually a pretty important decision, not just for the patient and their health but also because of the logistics of, um, the guards that are with the patient. They have to figure out, you know, how they're going to, to manage, um, and continue to kind of guard someone that's already in their system, while they're being admitted to the hospital.

So tha-, those are things that we think about. Um, and then as an emergency physician who’s seeing that type of a patient, um, will I need to, am I able to discharge them out of the emergency department back to where they, where they came from and am I gon-, going to need to coordinate some type of care for, um, care to continue, um, during their incarceration?

I: And so could you, um, tell me a bit more about your overall patient population? So I just get a picture of who you're seeing, pre-, uh, pretty typically on a day to day basis?

P: Sure. So we are, um, we are arguably the largest safety net hospital in the country. We see probably more Medicaid and Medicare patients than any other safety net hospital in the whole country, certainly in the state. Um, we are the busiest emergency department in the state. We're a level one trauma center, pediatric and adult. We see around 110,000 patients per year and they're pretty diverse, uh, population. African American, um, we see more American Indians patients than any other emergency department. We see Somali patients, we see Hmong patients. Um, we see patients that are, um, again, um, the majority of them are, are Medicaid, um, patients. Um, but we also are a referral center since we're so specialized in our trauma care and our hypobaric oxygen, uh, uh, therapy care, burn care, that a lot of patients are transferred to us from, um, critical access hospitals in our state and surrounding states as well as the community. So we see a very diverse population of, uh, patients.

I: And focusing in on your patients that are from racial ethnic minority backgrounds in particular do yo-, are you noticing any, um, unique challenges or barriers they're facing in terms of accessing healthcare?

P: Oh I, there's definitely. Yeah. When you talk about social determinants of health. Yes.

I: Could you give me an example or maybe a couple examples?

P: I think when you talk about social determinants of health and you think about things like, um, you know, having support both financial, um, emotional support, that sort of thing, um, access to healthcare, um, access to, to food. Access to healthy food. Um, access to transportation, housing. Um, those things are, are real for patients that live in certain communities that, um, that come to us for, for care.

I: And then how would you describe the disability status of your patients?

P: Meaning do we see a, a number of people with disabilities?

I: Mm-hmm (affirmative).

P: I would say yes. Yes. And that's across all different, um, you know ethnic races.

I: Okay and then thinking about, um, patients who are involved with the justice system in particular, um, have you noticed this having an impact on their access to care?

P: The ones that are currently in the, the criminal justice system?

I: Mm-hmm (affirmative).

P: And I guess I have to ask, do you mean, um, to clarify the term, being in the criminal justice system do you mean that they're currently incarcerated or that they, are they just somehow, what is your definition of being involved in the criminal justice system?

I: Yeah, so our definition is either being currently incarcerated in jail or prison or they may be on probation and parole in the community. So if you're, um, working with patients who are, have any of that type of involvement.

P: Okay. All right. Yeah. Um, I think the ones and this is my, my perception obviously, this is, you know, over you're interviewing me and my perception is that the people who are currently incarcerated that they have access to healthcare because it's provided, to certain degree, um, at the institution where they're currently being housed and again, uh, if they have a, an illness or an injury that, um, is outside the scope of what can be provided on site then they, they get brought in, um, to, uh, a facility, a healthcare facility like ours for, for treatment and care.

Now the ones that are in the community on probation and parole is probably a different, I'm guessing it's probably a different situation. Um, and wherever they are, they're currently living whether there in some type of a supervised setting or if they're in an unsupervised setting, um, you know that, those, that automatic healthcare might not apply, um, to those people. That's my feeling.

I: And do you have, um, some personal experience with working with patients that you know are on probation or parole?

P: Not necessarily. I mean I'm sure I, I, I mean I know I've taken care of patients like that but I don't really recall any specific, um, examples.

I: And, so for, um, patients who, um, and I'm gonna focus on given your unique perch and like working with a lot of patients that are coming directly from jail or, um, the prison system, um, how does that, could you speak a bit to how that impacts your treatment plan for that patient?

P: Mm-hmm (affirmative). Um, I think in general, someone comes in and they're, um, that, I don't know. I feel like at least I'll speak for myself that I feel like I need to be very, um, thorough. Not that I'm not thorough with other patients but I feel like that I need to be thorough for patients that I take care of, um, that are coming from that environment because if I, if I miss something, um, it may not get addressed in a timely fashion. Um, so I feel like I'm actually a little more careful in trying to maybe overturn as many stones as I can with that visit 'cause they may not have, necessarily, um, as much access as other people. That's kind of how I, I look at it.

Um, does it sometimes affect decision making of can I provide certain treatment to somebody and can I send them back to their institution? Where as, I may have admitted a patient, you know, with a similar who wasn't, wasn't, uh, in the criminal justice system, with a similar condition, does that occur? I mean it doesn't really, um, it's not something that I, I, I typically am thinking about doing but I think that that probably does occur, um, by other providers. I'm guessing it does. And, you know, we all have biases and I think that there's probably some bias out there against people that are in the criminal justice system unfortunately.

I: And so I have one follow-up question to that. Um, is there any communication that goes on between you and the prison or jail facility that patients might be, um, going back to in terms of following up with follow-up care?

P: Yeah, typically it's done kind of in a written, written form, kind of like our follow-up instructions. So there is that communication and that's the same, not, not just types of patients but with any patient that comes in our emergency department. Um, if there's a specific treatment plan that we, um, want to see followed through whether it be a course of medications or something of that nature or follow-up, um, then yeah that's, that's definitely communicated but it's usually done kind of in the written form.

I: And then are you ever communicating with, for instance, parole, probation officers or the court system about any of the patients that you're treating?

P: No.

I: And then aside from justice system involvement, um, for patients that have some type of involvement with the justice system, what are you seeing them dealing with socially in their lives?

P: Um, I don't, again that's kind of a hard question to, to answer, um, because we see them, um, at least ones that are incarcerated it's hard to, uh, unless you ask those questions, to get an idea what people might be experiencing. Um, and there are times when we have to, when we do ask those questions about, socially about, you know, do you have a support system? Um, um, are you currently, um, you know, do you have access to food, food insecurity and those types of questions that we ask. We always ask about, um, substance abuse and, um, mental, mental health as well. You know, if you're feeling depressed or you’ve thought of hurting yourself or, you know, do you use, um, illegal drugs or alcohol? You know, tobacco, those types of things. We ask those, uh, the questions to every patient and so, um, uh, it's hard to say whether or not the-, those patients that are involved in the criminal justice system are, um, more likely to respond yes to certain questions like that, like with substance abuse for instance or mental illness but, um, it su-, it sure seems that way.

I: And then for patients, um, who are involved with the justice system again who are coming to you, what are they dealing with medically?

P: Say that one more time?

I: For patients that are coming to you who are involved with the justice system, what are their, what are they dealing with medically? So what are some of the medical conditions that you're seeing?

P: Okay. Yeah. So I would say the overwhelming number of patients that we see in the criminal justice system are, would be considered uh, of a younger age. Younger than, younger than age 50 I would say. Um, and so many of them are a lot of times healthy. Um, do we sometimes see patients that have chronic medical problems like diabetes and asthma, um, those types of things? We, we do. Um, but I think, um, in, in general it's, it's, in general, I'm gonna say it's, um, a relatively healthy population. There of course are exceptions to that, to that, right? And you might see a middle aged or older person who, um, by virtue of their age, um, probably have, um, some, some medical problems but in general I would say most of the people are relatively healthy medically.

I: Mm-hmm (affirmative). And what are some of the mental health needs that you're seeing?

P: Well I think, again, um, there, there are a lot of patients in the criminal justice system that have, um, mental health issues, um, and also probably, um, concomitant, um, substance abuse issues and sometimes those things go together as you know. Um, so I would say that there's a lot of people that are suffering from, um, things like bipolar disease or depression. Um, and again that, that probably has a lot to do, um, many times with their, their substance, um, abuse disorders as well.

I: And are there any resources or services that you're seeing that your patients need but just aren't available to them?

P: Criminal justice patients you’re talking?

I: Mm-hmm (affirmative).

P: Um, again, like I said, I haven't really thought about that. I think, you know, we, we have a very active social services component at our hospital and in our emergency department and, um, that's a good question. I, I don't re-, recall myself ever like doing a social services consult on someone who comes in from an institution, you know what I mean? And I guess, there are times when we don't know if someone is on parole or probation 'cause they may not offer that information to us. So I, I don't, personally I don't routinely get like a social services consult on those people so I'm not really aware if they have, if there are any services that they may or may not need.

I: And so there is, I wanted to go back and follow up on, um, something that you mentioned, um, earlier, um, about guards being present which is something that's different from the everyday patient who's gonna come in and how does that, um, having a guard present when you're, um, treating a patient and having conversations with a patient, how does that impact, um, that experience?

P: Yeah. Um, it, it is a, it's a very, um, it's a different experience, obviously, and you have to acknowledge the presence of the guard, um, just like you would introduce yourself to the patient. I always introduce myself to the, whoever's guarding them as well. If I'm gonna have a, um, I, I typically say is it okay to, um, that, that I speak in front of this person, the guard, you know, and I don't think I've ever had it, I, I, somebody who is, um, a patient say no but I, I imagine that situation does come up and then there are, there are certain times when you may not, you know, if you think about, if you have a patient who's potentially dangerous, um, and, and you're, you're fearful that you could, um, you know being left like alone in a room with somebody even though they might have, um, handcuffs or shackles or whatever on, then that, that, obviously, you know, might make you think about, twice about, um, being in the, in the room with that, that person. So it does, again, unfortunately, make you think of those types of things.

Um, but personally I, you know, I, I typically ask if it's okay that I talk to them and talk about their medical needs and why they're, they're there for that visit, uh, with the guard present and I, I've personally never had anyone say no. But it's a, it's an interesting situation sometimes.

I: Yeah. Are there ever instances where the guard wouldn't be present? Where, just kind of curious. Are they always there or are there certain situations where you would ask the guard to leave or someone would ask the guard to leave the room when they're treating a patient?

P: Yeah I, I think, um, if someone is there and it's, it's, you know, it's for a very kind of personal type of a problem or if you have to, during an examination if you have to examine someone, um, in a very kind of personal part of their body then, um, then yes. I, we have the, the guards, you know, and they may be close by. They may be on the other side of the curtain but they're not there. They're like, you know, while we're doing our examination. And similarly, like I said before, there are times, um, whenever you would, if you were having a, a personal conversation or you wanted to have a very, kind of an intimate conversation about a personal problem, that's why I ask, you know, if, if it's okay for the guard to be there. Again I personally have never had, um, a prisoner or someone involved in the criminal justice system, um, say, say no but again that, that probably does happen.

I: And then thinking broadly, um, are there any changes to how we deliver healthcare that you would suggest to mee-, better meet the needs of patients who are involved with the criminal justice system?

P: Again that's a tough question to answer. Could go off in many different directions. Um, you know, like, I, I wish there was a way to kind of decrease the, the stigma and kind of that, that, you know, when someone comes in, um, I don't know, I can't think about a way that you would necessarily be able to do that though because if someone comes in and they're, they have guards with them and they're dressed in a certain way that identifies them as being in the criminal justice system, then, um, there's probably not a great way that you're going to get rid of that type of a stigma or automatically, um, have some of those biases that ca-, can come in before you're, before you're taking care of that patient, you know? Um, in terms of what other things we can do, I mean I, I think, and again I'm no expert on what services are provided.

I have somewhat of an idea of what, what services can be provided on site, um, that could probably be improved is my guess. And probably, you know, I know people are doing a lot of work in this, but probably, um, follow-up, um, for, for these people who are involved in the criminal justice system and how are they going to have follow-up for their medical conditions, um, their mental illness, their substance abuse problems, after they get out of the criminal justice system is probably the, the answer. That, that's the, that's the, the question that people need to answer is how do we continue to provide services for these people and to try to, um, you know make them a better person. Make them a healthier person.

I: And so thanks again for your time today. Before I officially wrap up, is there anything that I didn't ask you about that you think would be important to add?

P: I don't think so. This has been a good exercise, I think, for me. This, you know, you don't typically think about the definition of the different types of terms that you asked about, you know, um. So yeah that, I mean I think it was a, just to talk about these types of things 'cause we don't, we don't talk about them very much. You know, you have, like you asked me before we don't get education or training about, um, thinking about these types of patients other than just caring for them as we would any other patient. So no, I think it's been, it's been good.

I: All right. Thank you.

P: Sure.
