# Supplementary material for: Physicians’ perspectives on continuity of care for patients involved in the criminal justice system: A qualitative study
Source: PLoS One. 2021 Jul 14;16(7):e0254578. doi: 10.1371/journal.pone.0254578 (PMC8279398; doi:10.1371/journal.pone.0254578)
Supplement: S2 File — (ZIP) [file pone.0254578.s002.zip › Clean/Participant_18_Audio1_deidentified.docx]

I: All right. So thanks again for taking the time to meet with me today. This interview is designed to just get a sense of what you know about the criminal justice system, um, any experiences you've had with treating patients that have some type of involvement with the criminal justice system-

P: Mm-hmm (affirmative)

I: So, to start us off, I just want to get a general sense of what you know about the criminal justice system. So, could you tell me a bit about what you think about the current state of the criminal justice system in the U.S.?

P: Um, I think it's a major problem. Um, I think um, I think it- it needs huge reforms. Um, um, I think um, it's actually harmful to people, rather than having uh, um ... For the- for the folks that I'm thinking of especially, rather than having a focus on rehabilitation and um, sort of- sort of, a compassionate sort of, perspective on understanding, sort of um ... If- if a crime has occurred um, uh, certainly there's- there's like, society needs uh, s- some way to address when a person harms another person.

Um, or if there's some- some sort of action that happens um, that is uh, you know, uh, not in the best interest of- of- um, society. However, um, we have a major problem with um, like, institutionalized racism and how it impacts criminal justice system um, disproportionately um, people of color in the criminal justice system, uh disproportionately people with mental, severe persistent mental illness and severe mental illness in the criminal justice system.

It's become um, sort of uh- uh- sort of a de facto like um, "Asylum sort of for the United States." Um, quote, I put my ... I had quotes there in my um ... So I- I think it's um, has a lot of really serious problems. Um, uh, I don't know. I- I figure we're going to get into more detail-

I: Mm-hmm (affirmative).

P: So I don't know how much to go into now, but I um ... Unfortunately like, as a provider I don't- I don't um, trust it um, with uh, and for like the patients who I work with um, because I work with so many individuals who have been caught up in the system in lots of injust- in unjust ways. Um, and so uh, yeah. That's a little overview. (laughs).

I: Yeah. So next I'd like to discuss some criminal justice system terminology. Could you explain to me what comes to mind, when you hear the following terms? And there are a few that I'm going to go through, and the first is prison.

P: Mm-hmm (affirmative) Um, a prison would be a um, a- a- a building that is a locked facility, locked and you know, thought to be- thought to be secure facility, described as a secure facility. But secure for who? Um, and uh, um, where people who have been convicted of crimes. I think of a prison at a state level um, or a federal level versus uh, a jail more at a county level.

Um, so um, a place where people are um, have very limited um ... They obviously don't have much ... They don't have any freedom to- to move outside of that space, without like um ... They're in a locked facility um, and there may be um, shared cells. There may be individual cells. There may be sol- times in solitary confinement um, and it's sort of uh- a regimented like, institution setting where um, people are locked up. (laughs) Yeah.

I: And how about jail?

P: Um, the setting in a jail? I think the jail is uh, more at the county level. So, structurally maybe a smaller building but with the same sort of like, staffing that you would find like, you know, of- officers or people who are ensuring that um, inmates are following rules and um, staying uh, you know, maintaining with the behaviors that are expected of them.

Um, yeah again I think like bars, you know behind bars. Uh, cells where people are. Um, spending their time uh, sleeping and doing whatever daily activities perhaps with some spaces for you know like, communal like eating areas or spaces for outdoor ... I don't know.

Maybe there's like a basketball court or something, you know? But, not much in terms of recreational facilities. I think of it as very um ... Like from the ones I've been in, like very barren, very sterile, very institutionalized feeling.

I: Could you tell me a bit about how you distinguish between jail and prison?

P: Um, I guess I just have always thought of it as like prisons, I think of is more like a state for the federal level and the jail more the county level.

I: Mm-hmm (affirmative).

P: Um, and the- that who ends up in jail versus prison is determined by the severity of the crime, and of sort of the um, uh, sort of ... If it's a holding space for a while, I might imagine someone might be in jail as they're waiting for something, whereas prison is a little bit more of a- there's more of uh, f- uh, some sort of final verdict has been made and someone has been sent to prison.

Someone might be sent to jail as well, but I think a lot of people sort of, in jail sort of waiting too for a decision to be made around like a charge, or an arrest. Um, mm-hmm (affirmative).

I: What comes to mind when you hear the term probation?

P: Probation is sort of an, like an- alternative to jail. I would say, like a um, way ... Some agreement that has been come true between the judge, the ... I don't know. The person um, uh, that the potential um, person who may potentially be spending time in jail um, that they uh, have come to s- there's been some agreement that's been made um, to allow the person to remain in the community.

Um, uh and not be physically in the jail setting. Um, in the community but with um, sort of court ordered supervision. Um, and that can involve like, meeting with their probation officer. Um, perhaps it might involve some sort of mandated treatment, if they have a mental illness or substance use, uh disorder. Um, it might involve some other sort of community rehabilitation plan.

Um, I don't think it's- I don't think of it as diversion at all, but like, um, but it's an alternate to being in jail. Um, like the charge has not been dropped. Uh, they haven't been cleared of you know, um, by the court and there's still some sort of oversight the court wants to provide.

I: And then what comes to mind when you hear the term parole?

P: Parole. Um, I think of parole as ... I'm not sure if it's always early release from prison, but it is um, sort of someone who has been released from prison. I think often it's in court- potentially an early release. Um, uh, and so again there's a parole officer that is assigned to the person.

There's court mandated requirements for supervision in the community, for check-ins um, you know I think to ensure um, to ensure the person's doing okay, not recommitting any crimes, not um, you know, if there is some rehabilitation plan, like that they're um, taking steps towards that.

I- I don't think of a parole officer as like very, very involved in- in that piece, but um, like a probation officer. But someone who hopefully would have like, a person's best interest in mind to help them succeed in the community.

I: And then are there any other ways that you distinguish between probation and parole?

P: Um, I don't know. Distinguishing further? Um, I- in terms of the implications for the person or- or anything?

I: Anything really, yeah.

P: Anything? Um, I don't know. I wonder uh, I'm not sure I'm maybe there is a um, uh, like how public the record is, if someone is on parole versus probation. Um, because someone on probation has been convicted of a crime and is in um, has spent time in prison, and probably an early release from prison.

Um, I think that it can impact more um, there um, opportunities for um, finding employment, getting housing, um sort of what shows up on someone's legal record and a background check. Um, I think? I mean these are great questions. It's having me think-

I: Mm-hmm (affirmative).

P: Like how come there's a- these are things I really need to understand better. Um, but my sense is someone on parole um, who in my understanding has been convicted of a felony, and now has been released from their time um, in prison with that court sup- uh, ordered supervision. Um, like there's a lot more- there's a lot of barriers. Um, but those come up for people on probation too. I just don't know the difference-

I: Mm-hmm (affirmative).

P: In like what actually shows up in someone's record, probation versus parole. Um, 'cause I've had people who have spent time in jail and come out on probation. Um, it seems th- the nature of the crimes or the convictions-

I: Mm-hmm (affirmative).

P: Have been different like a misdemeanor versus a felony.

I: Okay.

P: I would love to take the course-

I: (laughs)-

P: When you guys put it together. (laughs).

I: Okay, so next I'd like to shift a little bit to learn more about your background and your education and training.

P: Mm-hmm (affirmative).

I: During medical school, did you ever receive any training, and whether this was formal or informal, on working with justice involved populations?

P: I don't remember any formal training.

I: Mm-hmm (affirmative).

P: Um, I mean, it was an interest of mine, so I sought out um, training and there's a lot of intra-section like in community psychiatry and with the mental health system. There is um, uh, like with mental health courts and um, so like that was something I needed to like, train myself on, and understand what options do my patients have-

I: Mm-hmm (affirmative).

P: Um, in terms of like if they're facing some sort of, legal issues and I'm worried that it was um, like, the actions that they took um, that led to an arrest or um, were due to like a decomposition in their mental health. And so trying to, like learning about ... In my residency program, we did receive education on like, what is mental health court?

Um, we also like, forensic psychiatry is a, like a, fellowship one can pursue in psychiatry. So we did have courses in forensics, um, forensic psychiatry in particular, and forensic evaluations and um, sort of how those ... Or you know, what value those add um, relative to uh, general uh, psychiatric evaluation.

I: Mm-hmm (affirmative).

P: Um, so I feel like in residency I- but I don't really recall in medical school, getting any training.

I: Mkay.

P: Mm-hmm (affirmative)

I: And were there any other types of trainings that you did in residency-

P: Mm-hmm (affirmative)-

I: In addition to what you already mentioned?

P: Um, yeah I mean there was the opportunity to actually like, shadow a psychiatrist in a mental health court um, or yeah ... Like who worked for mental health court-

I: Mm-hmm (affirmative).

P: And to see how those evaluations occurred um, which was valuable. Um, I myself didn't um, pursue this, but there were um, there were opportunities t- to um, like shadow and work with s- um, physicians who were going into jails and providing um, treatment like, in correctional facilities.

I: Mm-hmm (affirmative).

P: Um, and then like, there was also um, training available um, and that I sought out as well around like um, um, asylum and deportation and like how to do asylum evaluations. Um, you know? That whole piece. Actually, that- that- that- that component did happen in medical school too. There was an opportunity to learn about um, how to do asylum evaluations in medical school.

I: Did you talk a little bit more about that? I'm not familiar with-

P: Asylum process?-

I: With that, yeah.

P: So, when someone comes um ... So an asylee is someone like, uh, who is fleeing their um, home, native country due to fear of um, persecution based on religious beliefs, sexual orientation, um, like any number of factors that are related to their identity, their belief systems, who they are.

Um, and um, so there's uh, a status versus like refugees who fl- who flee their countries um, because of um, often political violence and fear of safety if they s- were to stay in their home country. Um, asylees um, are, have like a- a different legal designation because they're actually ... Also, f- fleeing like fearing that they personally will be somehow persecuted or harmed because of something about them.

Um, and so there's a- there's a better legal definition for that, but that's ... So, when asylees show up at one of our borders, um, without like, a visa to enter the United States, they can ask to seek asylum and um, rather than ... So then they go into like a different processing, you know?

I: Mm-hmm (affirmative)

P: Um, well a lot of times unfortunately, many of them end up in like detention centers because our system's so messed up. Um, but ultimately um, if they are seeking asylum, they're granted like, some period of time. Um, a lot of times like, their cases don't go up to actually be heard in front of a judge for like eight to 10 years.

Um, where a case would be made um, that um, based on their fear of like, persecution or torture if they were to return back to their home country. Like, it is not safe for them to do that. And the evaluation piece comes in because a lot of times there may be a medical examination or a psychiatric e- um, evaluation that will support uh, and corroborate the story that that person has, is sharing.

Um, so for instance like, if they were tortured, like scars that they have on their body that were seen in uh- in a medical evaluation and documented and a psychiatric evaluation. Um, you know, aspects of um, their story, the consistency um, you know, that they're like current PTSD like does make sense based on like, was caused by like, the events that they experienced.

So, so there's just ... A lot of times, like attorneys who are um, uh representing individuals seeking asylum like need support from um, medical and mental health professionals who can do these evaluations. So, I got some of that in medical training, as well as residency.

I: Yeah?

P: Yeah.

I: And then, as part of your training, would you complete a fellowship at all?

P: Mm-mm (negative).

I: Okay.

P: Mm-mm (negative).

I: And then, so now like, thinking about where you're currently employed or a past place of employment-

P: Mm-hmm (affirmative).

I: Have you received any training on working with justice involved patients?

P: Like since I finished residency?

I: Mm-hmm (affirmative)

P: Um, you know I have sought out, like when I attend different conferences, uh, community psychiatry conferences um, ... Like often I've- I seek out um, sessions. Um, so it's like my own interest. Um, I don't recall if we've done any trainings at our clinic specifically for justice involved.

I- I think we've- we've ... I'm sure we have. I'm not, I don't always, I'm not always able to attend the ones that happen at our clinic. Um, so we've had a lot of trainings on, um, uh like Immigration 101 and uh ... 'Cause we have a lot of our patients are refugees and immigrants. So, um, sort of like, what's the difference between a refugee and an asy- asyl- asylee?

Um, you know, what are the different legal pat- like ... You know when can people start applying for like citizenship and that sort of thing. What's happening to people if uh, they're on a deportation list and they're, you know, picked up. Where do they go? What rights do they have? Like, that sort of um, those- that kind of training we've had.

I: Mm-hmm (affirmative).

P: Um, offered at our clinic ... But like the question, the few questions you've asked me just now, like I don't remember about like probation versus parole, jail versus prison. I don't remember attending-

I: Mm-hmm (affirmative)

P: A session specific to that since um, being out of residency like, at my clinic.

I: Okay.

P: It's been like at conferences.

I: Okay.

P: And I, clearly don't remember very well because-

I: (laughs).

P: (laughs) Even though I've attended the sessions.

I: (laughs)

P: (laughs) I'm still struggling to give you good definitions. (laughs)

I: So then, during your day to day visits with your patients-

P: Mm-hmm (affirmative)

I: Are you asking them questions about whether they are currently, or have been in the past, involved with the justice system in some way?

P: Yeah. Yeah.

I: How do you like ask that question? What are you asking?

P: Yeah.

I: Mm-hmm (affirmative)

P: Um, like that. Um, so part of a psychiatric evaluation is um, also asking about th- their legal history. Um, and um, involvement with the justice system-

I: Mm-hmm (affirmative)-

P: Would fall under that. So I would ask um ... I mean there's a- a number of issues that come up. Um, so it's kind of like, uh ... There's a number of different categories I ask about. So one's like um, just around whether they've ever been um, committed um, um, by the court for mental health treatment, so like mental health civil commitment-

I: Mm-hmm (affirmative)-

P: So, that just like flows when I'm doing a psychiatric assessment of their past history, hospitalizations, past treatments. Have you ever been um, uh, committed for mental health issues? Um, or like you know, been ordered by a judge to um, participate in mental health care against your will?

Um, and then from there I- I can move into also asking about like, um, have you ever been involved with the justice system any other ways? I might ask um, have you ever been um- have you ever been arrested? Have you um, ever been um, charged um, with any sort of cri- uh, for any sort of crime? Have you ever been convicted of a crime?

Um, do you have any misdemeanors or felonies on your record? Um, have you ever spent time in jail? Time in prison? I mean, depending on where the interview goes, like ... I mean sometimes people are like, very early on, "No. No. No. I've never," blah blah blah. I don't have to go that deep into asking follow-up questions.

I: Mm-hmm (affirmative)

P: Um, but if there has been justice involvement, then I have to ask some more nuanced questions to understand um, and- and then that ties back to like their history as well, 'cause I'm also getting their history of their illness and so I'm trying to understand um, like ... I'm basically trying to understand their life course from like, you know, when they were little, to now.

Um, and so when the justice involvement periods happened ... Like I'm interested in how does that correlate with um, how they were doing? Like in terms of their physical and their mental health as well.

I: Mm-hmm (affirmative)

P: Um, how are they doing in terms of like, other instability or stressors in their life? Um, um, uh, you know? Substance use, um, employment, housing, like so it's all kind of ... It all sort of intersects and interacts and so um ... And then it's, I mean if they have had justice involvement, then I also want to understand um, what kind of care um, they received for their um, illnesses while they were in uh, while they did time. So.

I: Yeah.

P: Yeah.

I: So I want to like, learn a bit more about I guess, how that informs your care for a patient.

P: Mm-hmm (affirmative).

I: And maybe a more specific example might help to make that a little bit more concrete. But, how does that information kind of inform a treatment plan for a patient?

P: Yeah. I gotta do a little look around the room and see who-

I: Mm-hmm (affirmative).

P: Might be nearby.

I: Okay.

P: Um, so um, gosh in so many ways ... So like, I have- it depends like, I have people who are in and out the sy- of- of- the justice system frequently. Um, so when I- when they're with me now, like say today's visit, um, but they've been in and out you know, every few months, every year or two, um, like, I'm aware ... Like I have this like, time with them now, like that we're having this conversation-

I: Mm-hmm (affirmative)

P: Um, but that the risk of them returning um, given like the historical pattern, um, there are risk factors that they are going to end up back in the- uh, in- in jail um, or prison. So, I need to um, like think about um, in terms of treatment planning, one like um, like what's giving them a sense of meaning and purpose?

How connected do they feel socially? What supports do they have, how stable is their housing situation? Like what's your source of income? What are their own goals for themselves, in terms of employment, or like reconnecting with relationships um, family, children that perhaps have been interrupted um, and impacted.

Um, and so like part of it is just like, wanting to make sure ... Like we're tapping into like, what's most important to them, and then- and like, how are they seeing like, like, um ... Like what- what- how- what's- are they identifying they need most?

I: Mm-hmm (affirmative).

P: Um, to- to stay, to stay in the community? Um, knowing that like, some of it's out of their hands too, because of bias and discrimination. Um, but uh, so I mean that's like a big piece. I meant I'm often f- focusing on social determinants of health-

I: Mm-hmm (affirmative).

P: Um, and making sure we're thinking about who are the other people on a care team that can support them. Um, and making sure in terms of like, mental health treatment, substance use treatment, physical health treatment. Um, but the choices that we're making in terms of treatment options are like, um, are like, ones that can be sustainable too. You know?

I: Mm-hmm (affirmative).

P: Um, if- if they end up back um ... Like I'm not going to pick and I don't practice this way anyway, but I'm not going to pick like an expensive brand name medication, but I know they're never going to be able to get when they're in jail.

Um, so- so there's like that kind of situation and then I guess another example would be someone who's like out on um, you know who's been released from prison, he's on parole. Um, and again like um, has like, there are court mandated re- requirements.

Maybe they're seeing me because the judge says, "You need to see a psychiatrist." Um, "You need to get substance use treatment." So it's about like, for folks who are mandated to see a licensed health care professional, um, especially s- a psychiatrist which can be like a stigmatized um, uh, professional to see.

Um, it's really about like relationship building and um, uh, you know, really trying to understand who- who this person is as a person, what matters to them. But also, again like, y- you know. What are their goals? And like understanding what- what the requirements are of their um, parole like, what the stipulations are. Like what are the things that they- they cannot be doing that they violate, they'll be back? So that I can support them um, towards that. Um, so is that specific enough?

I: Yeah. Yeah. It's great.

P: I'm trying not to like, give-

I: Yeah. (laughs).

P: Yeah I can't really get into like, specific patients-

I: Yeah.

P: And, I'm thinking of like, amalgams. You know? In my mind of general approaches.

I: Yeah.

P: Um, but it's, and it's not always so ... And I often will ask, like depending on the situation ... This is- this is kind of a, little bit of uh, uh ... I'm- I'm not alway- it's not always black and white.

But sometimes it can be supportive to get a release of information so that ... And- and my patient wants it too so that I can be in communication with the probation officer, with the parole officer. Um, and that we're all on the same page. Um, and yet that individual is also, that ... The P.O. is also like, represents the system-

I: Mm-hmm (affirmative)

P: Um, and you know, um ... If there is a release on file, it's like how- navigating how much to discl- like if the person isn't doing well. Right?

I: Mm-hmm (affirmative)

P: It's not my responsib- I'm not the police. I'm not a judge. Like I'm a doctor that's taking care of my patients. It's not my responsibility to ... And I would never like, tell on my patient, right? That they have somehow violated their ... You know if they disclose to me, like they need to be able to share with me.

Like if they've relapsed on alcohol and that was like ... That's a probation violation or parole violation. Um, they have to be able to share that with me, without fearing that I'm going to take it to- to their P.O. Right?

I: Mm-hmm (affirmative).

P: So that, like trust is paramount and that confidentiality is paramount. Um, so I think that's- that's sometimes like, a little bit of uh, uh ... I haven't had much of an experience where I had like P.O.s calling me like, badgering me for information or anything like that-

I: Yeah.

P: But I think it impedes like it- you- can- ... It's hard to have like, like, collaborative care team relationships-

I: Mm-hmm (affirmative).

P: You know, with these people-

I: Mm-hmm (affirmative).

P: Because, the um, the priorities are- are different. You know? Not everyone at- has like the indiv- ... Like my patient is like the top priority. Um, and like what's best for them is a top priority. So.

I: Yeah and so would you ... So is the motivation behind um, communicating with the probation or parole officer, are you- are your patients asking you to do that? Um, or I'm just wondering a bit more about how that-

P: Yeah-

I: Like comes to be.

P: Sometimes it's my- sometimes like, the P.O. has said to my patient. You know, like if their- if it's been kind of rocky, or there've been some violations um, the P.O. will ask the patient if they'd be willing to have that channel opened up between myself and- and the officer. Um, sometimes it comes from the patient. They might think it's best for them. Um, I'm not always sure. You know?

Um, sometimes if I'm just not clear on like ... What is this legal situation? Like, is this a misdemeanor, or a felony, or like what- ... If like, for some reason a patient is not able to communicate to me like, what the actual, like what their legal status is um, and it seems like um, you know, it's been complicated.

It's been affecting their health and their mental health, like then I will um, sometimes request to have that conversation, but usually it's like, to get information um, so that it can help inform my treatment planning and not so much ... You know. Like unless, I mean it's a very rare situation ... Sometimes like, I have people who are like, sex offenders-

I: Mm-hmm (affirmative)

P: Um, and so there are scenarios where, having everyone meet together as a, in a care team like, has served to be helpful. I'm not saying this would be for anyone who's in an SO program, but um, it's one example I can think of where it helped, it served to be helpful because it was like everyone kinda including the patient, got on the same page about like, um ... And I was also able to advocate on behalf of my patient as well-

I: Mm-hmm (affirmative).

P: So, but it's, there's no like automatic, like if someone comes in and they're on probation or p- parole, we're automatically going to get a release of information. Um, I think there's pros and cons to- to doing it that way.

Like, I think we want our patients to be able to ... And there's a lot of cons. Like I think we want our patients to be able to come and trust us and not feel like, if they're gonna get care, that they have to sign a- or even be asked to sign a release to communicate with someone who's part of the justice system.

I: Mm-hmm (affirmative).

P: Um, so. If, I don't know. If the system were more compassionate and like, if like real reform happened, like you could imagine like, something like in a diversion type program or something where like, everyone really was rooting for the patient. You know? In a compassionate like, uh, way but that's not usually the case. So, hmm.

I: And then in addition to you know, what you've already shared, are there any other benefits to asking your patients about their involvement with the criminal justice system that you see?

P: Um, for sure. So, one like it can be very traumatizing. So um, I have so many people I work with who've experienced all kinds of trauma in jail and prison. Um, so that's an important piece for me to understand. Um, there are really negative effects of solitary confinement on health and mental health.

Um, so that's really important for me to understand. If they've ever had- if they've ever had uh, been in isolation um, uh, um ... You know there's other kinds of like ways that people have had to survive if you know, um, in those systems. And also like, especially for people who've- who have been institutionalized for years, like perhaps starting in their teens, and come out in their 50's or something, which I have worked with um, folks in that kind of situation.

Um, like, in terms of thinking about their emotional, social development, and um, you know, how they- how their- how they think about interpersonal relationships, like how um, how that experience has impacted their personality and um, ability to regulate emotions. Uh, you know?

There's just so much. So much has been um ... So many of those opportunities for like growth and learning, and the- just growing as a person, that were not available and um, the like institution of a ja- of ... For someone like that in a prison system-

I: Right.

P: So, and I'm- on the other hand there are people who I've also worked with who had opportunities for growth. You know? Like um, started to meditate or like, studied you know, got their G.E.D. or um, uh, started to work on some other skills um, perhaps got really interested in like, the- their spiritual life, um, or like had the opportunity ... It's not available everywhere but to like, actually do like, mental health treatment in that kind of setting and they learned a lot about themselves in the process.

Um, so, but I think um, I think it's an incredibly like, stressful and can be- it can be very stressful and traumatizing and so that has impacts on mental health. Also has impacts on physical health um, in terms of like risk for diabetes, cholesterol, all kinds of things. Um, all kinds of preventative care that perhaps was not properly addressed so that- there's just so many implications for health care for- for like for health. Um, and then social connections.

You know? Like people come out and they've been ... You know, part of an assessment for me is asking about who are your supports? Who are, you know, who are the- what are relationships most important to you? And, a lot of times those have, you know, been really hard to maintain. Um, so uh, yeah. I mean I- I- it's just a really important- important topic to ask about um ... So, for so many reasons. I- there's like-

I: (laughs)

P: So many other things coming into my mind.

I: (laughs)

P: I could talk about this for a really long time, but I know you probably have other questions too, so do I don’t wanna. Yeah.

I: Well my next is- is so are there any risks or challenges that you see-

P: Yeah-

I: To talking to your patients about this?

P: Um, sometimes people don't want to talk about it. Um, you know again, like a risk of ... Like I need to frame everything in terms of their health and their wellness. Um, because, a risk is, if I start asking questions or bringing curiosity to being justice involved, they will- they might think like, like, you know. Uh, like, "I thought this was a clinic. Like are you like uh, cop?" You know what I mean? Like-

I: Mm-hmm (affirmative).

P: It's um, it's really um, balancing that um, and like, really framing why it's so important um, that I as a physician understand um ... Because of all these ways that being involved with the justice system can affect health and mental health, why it's so important. I think a risk is um, a lot of people who I work with um, uh, who are justice involved um, may have challenges with trusting new people.

I: Mm-hmm (affirmative)

P: Trusting anyone who's part of an institution. Right? So a clinic, is a- is an institution, right? Like it's a health ca- part of, a health care system is an institution, so um, that's uh- that's a risk and a challenge. Um, people might get really um, upset even having to bring it back up, like it might be something, some part of their life they've been trying to like, bury and push away and move on from.

Um, so and then- and then another challenge is just like, if I'm not really familiar with what's going ... like um, even though I work with so many people who are justice involved like, uh, even just this conversation is making think like this is- this is still an area ... Like I need to understand even more deeply.

You know? 'Cause a challenge could is- is sometimes like, they tell me about something related to their legal status, or probation or if they were at the work- work house or they were you know. Um, but like I don't fully understand so, I'm- I'm- asking about it, I'm getting information but like, then what do I do with it? You know?

I: Mm-hmm (affirmative).

P: Um, so it's another challenge sometimes.

I: Could you tell me a bit more about your overall patient population and who you're seeing on a day-to-day basis?

P: Yeah. Um, so our population at the clinic is very diverse. Um, we see people who speak like eight, 10 different languages, 12 different ethnicities. Um, we get a lot of immigrants, refugees, um, people who are um, uh, Native American folks, African Americans, um, so it's uh, it's- it's pretty diverse. Um, I see also some people who are like referred um, just from the community too, like from community therapists or [inaudible].

Um, most of our patients are Medicaid um, under-insured, um, and then we h- have a grant through [county] um, to provide behavioral health services to people who have no insurance, which largely nowadays is like, people who are undocumented. So, um, we see a lot of people who speak ... Yeah, many different languages-

I: Mm-hmm (affirmative)

P: Um, just like the-

I: Okay.

P: Yeah.

I: How would you describe the disability status of your patients?

P: Um, in terms of like formally with social security disability, or-

I: Both. So we're-

P: Yeah.

I: We're getting to know a mix of answers-

P: Yeah. Yeah.

I: About this, so I'm like really interested in how you define disability.

P: Yeah. So like, disability technically is um, you know, having like- like a condition that impairs one's ability to um, sustain, maintain, um meaningful, gainful employment, which my understanding ... That definition is meaning like, maintain like 40 hours a week, like a full time job. Um, and through that like have gainful em- gainful employment and income will help support one's livelihood.

Um, so to me a disability um, is something that you know, is uh, uh, the person has an illness, a condition that is impacting their ability to um, to- to have that- that level of um, workability. Um, sometimes people can work part-time but they're still disabled. You know?

I: Mm-hmm (affirmative) Yeah.

P: Um, uh, but a lot of the people I work with have difficulties you know, in terms of like having depression or their bipolar illness, or uh, schizophrenia, or um ... A range of illnesses like, impact their ability to- to focus, to concentrate, to like interact in ways that you would need to in a- in a workplace with other people.

Um, so that's how I think of a dis- as a disability, but it's uh, um ... It's like disabled according to who? Right? Like, um, it's not like uh ... I think of it as like, for- for disability specifically it's like around, that kind of disability's around like, work disability.

There's a lot of other kind of ... You know people, there's a lot of other like, neuro atypical people who you know, can thrive in certain settings, but um, uh, not in like say a traditional school setting where they have to sit at a classroom but like you put them in a different kind of setting, smaller classroom. You know? Less students, more activity, more time to move the body, like you know, they can thrive so I think it's also ... There's also like social construction of disability.

Um, uh, but like for this- for the purposes of like, when people approach me for like, a disability um, you know to support their application for disability, like social security disability, it's often because it's impacting their ability to work. Mm-hmm (affirmative).

I: Yeah, and then specifically um, for your patients that have some type of justice system involvement um, I'd like to dig a bit more into you know, that experience and, you mentioned that some are specifically referred to your care. Could you talk a bit about that, and how your patients are finding you?

P: Um, I think our clinic is like probably on some list, in the county or something-

I: Mm-hmm (affirmative)

P: And the court system. Um, so, I think that's how- how we get referrals. Um, because you know as a community clinic and, a community health clinic can mean mental health clinic, uh federally qualified health care center. We don't turn anybody away. We have a sliding scale fee. You know?

I: Mm-hmm (affirmative)

P: Like, we work with people. We often eat costs that ... Um, like no one gets turned away from our clinic, so, uh, people ... I think the system sends people to us. What was the other part of the question? Other than referrals?

I: Uh, oh. I guess how they're finding you. Are- are all of your patients you know, do you think they're referred to you from [county] or somewhere else? Or do you just have people coming in-

P: Yeah.

I: Off the street?

P: Yeah.

I: Do you have a sense that-

P: Yeah. It's a mix-

I: Mm-hmm (affirmative)

P: I mean, I'm always so shocked that I have patients who like, there was no transition to community plan in place for this person.

I: Mm-hmm (affirmative)

P: At least nothing they can articulate to me. Um, and they got discharged from, released from jail with no medications um, nowhere to go, no place to sleep, um no like program to be connected to. Um, and people know about our clinic in the community so maybe they came to our clinic when they were a kid growing up, and so they've come back, because they're like maybe this clinic ... Someone here can help me.

I: Mm-hmm (affirmative)

P: Um, so, I think there's people who self present. I think there's people who get referred. Um, and there's people who are like, in a group home or some other sort of other residential treatment, like substance abuse residential treat- chemical dependency, uh residential treatment or in uh, a group home for people with mental illness, um, or a range of like, those kinds of community settings that would get referred. There's people sometimes that come to us because they're like getting ... Like they've managed to connect with like, community ba- based program-

I: Mm-hmm (affirmative)-

P: Like a community support program like [neighborhood community support program], or um ... And then someone there recognizes that they're not actually getting like, any medical care or psychiatric care. So they send them over. So, it's a range. I- I don't ... I mean if someone is mandated and court ordered that's, then like usually those folks, like the probation officer has like, helped make the appointment for them, and like then they show up, and if they don't it's like a violation of some sort.

So that's like, one category, and then there's oth- a lot of folks that are justice involved that- that ... It's- I just, it's like just so awful. Like they're just, they just come out and it's like th- they have, there's nothing. They have no structure, they have no money, they have nothing, and especially if they have like, physical or mental health problems, but like, you know, they should be receiving treatment.

Like to me, that's just like totally unethical that those folks like, come out and ... That anyone comes out and like, there's no plan in place to help connect them to services and supports. Um, you know. Not everybody's got like- like a family waiting, like, with their door's open saying like, "Come on home honey, to your bed." You know?

I: Mm-hmm (affirmative)

P: Like um, you know a lot of people are just- are sleeping on the streets. It's- I think it's- it's just so wrong. Yeah.

I: And then so for patients that have been incarc- so in jail or prison at anytime, um, do you have any insights on what their access to care while they were incarcerated so was- what it would've been like, or was like for them?

P: Um, it's something actually I've been wanting to like, understand more. Like I know there are guidelines for physical and mental health care in correctional facilities, but I don't actually know like, what is the bare minimum required, like-

I: Mm-hmm (affirmative)

P: I don't know especially with privatization of correctional facilities. Like I have no idea what- what by law is required, in terms of care delivery in that system.

I: Mm-hmm (affirmative)

P: I feel like it can really vary. Like, a lot of times, like my patients will tell me they've- they saw a psychiatrist um, in jail, but like I don't ... I know they certainly have very limited formularies, meaning like the drugs that they can access-

I: Mm-hmm (affirmative)

P: Um, so a lot of times people like, are stabilized on a particular regimen when they're in the community, and then when they go back- when they go to jail or prison, those meds aren't available. Um, so, but I don't actually know what the like, what's the m- what's the minimum we can expect?

I: Mm-hmm (affirmative)

P: I'd like to understand that actually.

I: And then, for your patients that have some type of involvement with the justice system, what are you seeing them present with ment- or medically when they're coming to see you?

P: Oh, it depends. All kinds of things.

I: Mm-hmm (affirmative)

P: I mean, from uh ... Oh like all kinds of things, hypertension, prediabetes, diabetes, being overweight, um, uh, you know, headaches, vision problems, hearing loss. (laughs) I mean untreated asthma, um, other sorts of breathing problems, um, GI issues, sexual health. You know, like perhaps have- have not been screened for sexually transmitted infections or HIV in a really long time-

I: Mm-hmm (affirmative).

P: Um, yeah. Body aches and pains, just like musculoskeletal, um, all ki- I mean across the board. I don't know if there's like, something that's comes to mind that's like, I'm seeing a lot of. You know? It depends on like the age range for ... I'm thinking like different age- age groups have different risk factors for different kinds of medical issues. So, um, yeah.

Among women um, uh, you know like- like whether they've had- ... When was their last like preventative health care screening? Their last pap smear, their last cervical cancer screening, um, overdue for mammograms. I mean, you know if there- if there was a- a pregnancy in, while uh, incarcerated. Um, that's a whole nother topic-

I: Yeah.

P: Like women in the justice system? I mean I ... It's interesting as we've been having this whole conversation, a lot of the people are coming to my mind are- are men.

I: Mm-hmm (affirmative)

P: Um, but the women that I've worked with too are ... And that's just interesting for me think about like, why has it been that way? But uh, there's all kinds of challenges I think that women face, also.

I: Mm-hmm (affirmative)

P: Yeah.

I: And then, are there any services or resources that you're seeing that your patients need but aren't available to them?

P: Yeah um, I mean one is like, ref- reform (laughs) of the criminal justice system. Prevent incarceration, like that's like the number one thing, like diversion programs like, identify like, you know, what kind of supports like community supports, social supports, uh health, mental health, substance use treatment, like would help, potentially help this person.

Offer it as an alternative, um, that would be mind blowingly amazing. Um, uh, and then in coming ... So, when they do um ... If when they are incarcerated like, uh, connection between ... Like I've never had a jail or prison reach out to me for my records or to say like ... Actually maybe, maybe once it happened, actually.

I: Mm-hmm (affirmative)

P: Actually one time. I can remember someone from like Stillwater called me. My patient was there. Um, and they were really struggling with like controlling his uh, illness and she needed like some guidance from me, and like wanted to talk with me about like, what I- how- how ... But it's because like, she was really stuck, but it- it wasn't like- like, "Hey I've got your patient. You know, I wanted to update you on."

Like there's- there's no collaboration, and I think that would really help, because I think our patients go in uh, to jail and then like, they get totally cut off from all of their community um, community people. You know? Like providers and uh, supports um, from like different agencies ... I mean it's one thing, maybe they can stay connected to like, maybe family th- through like phone calls and visits.

But those of us like, more in the professional space, um, there's no initiative that happens. A lot of times I don't know 'cause they just, they don't come b- ... Like they miss their next visit. You know?

I: Mm-hmm (affirmative)

P: I have no idea what's happened. If we- our client tries to call them, like, you know the number's been disconnected or their ... It goes to a voicemail box that's full or something. So like, it's just like, that would ... I think that would, that could be really interesting to explore, like how to have more like, intentional collaboration between the treat- the providers in the jail setting and their outpatient supports and providers.

And then, upon release, like the third, the third thing would be like, upon release, really doing more for transition to community. Um, I just, I don't, I'm like ... No person should leave jail and be homeless. Like, there should be a place where they go and, they can sleep (laughs) at night, and like some ... You know? I don't know uh, like this is me talking like I'm-

I: Mm-hmm (affirmative).

P: I know these conversations are happen a lot and like, you know? What- people who are going through this experience need and want, is like the most important thing to prioritize. Maybe they don't want to go to a home with like, 10 other people who've just gotten out of jail.

Like, but something like, where if it's not a group home, it's some- some way to ensure stable housing. Like without stable housing ... Like it is really, really challenging for someone to get out of um, come- get out of jail or prison and like, those- such high expectations of a human being. You know? To um, to just like, find one's way after being institutionalized.

Like, so, so I would like to see like, a lot more intentionality with connecting um individuals to those kinds of resources, like, the basic needs of the social determinants of health, as well as access to healthcare.

Um, it should be part of a like, you know, transition plan and there should be people responsible from the justice system who are ... Like, it is their job and responsibility to make sure ... Like if you're going to put people into the system, like you need to take responsibility to make sure that people safely um, uh, sa- I mean at minimum safely transition-

I: Mm-hmm (affirmative)

P: And also can thrive in the transition, because that's- and that- to prevent like you know, that like, bouncing back into the system. Um, and also it's best for the person. So, um, yeah and then- and then like I- I don't know the fourth area would be prevention-

I: Mm-hmm (affirmative)

P: Then a fifth area would be like, you know, really addressing institutionalized racism in the system. I mean I know it's not a direct resource for people, but like um, like that- that reform needs to happen. Yeah.

I: Yeah. So thank you again for your time today.

P: Yeah.

I: Before I officially wrap up, is there anything that I didn't ask you about today, that you'd like to add?

P: Um, no. I mean this was a really interesting experience. I appreciate it. I haven't had the opportunity to just talk with someone about like-

I: (laughs)

P: What do I think about these systems? Um, in a long time. I imagine as the day goes on, like, I'm going to have ... Little thoughts are going to come to mind, like, "Oh I could have talked about that." But, I don't know. Off the top of my mind, um ... I would say just like, a- a topic that is like, a really big problem in our population that we see is um, like folks facing deportation.

I: Mm-hmm (affirmative).

P: And how, their past felonies um are like, prevent them from being able to pursue a path towards citizenship. Um, and rather put them on ... It gets them put on a deportation list and like there's such an opportunity for rehabilitation um, that you know, just, like, our current government ... Even, even past governments have not really seen and appreciated.

I: Mm-hmm (affirmative)

P: So, those are like really sad scenarios for me too, like people who have like, families here who like, have lived here for years and years, like perhaps because of ... Often in a context of like, an untreated mental illness, because they didn't have access to care, or didn't understand what was going on with them.

They ended up um, like there was some assault, there was something that happened, led to a felony, time in prison, and now it's on their record. And even though they've like ch- like changed so much and engaged in treatment, no longer using, like- like the most amazing people you could meet. You know?

I: Mm-hmm (affirmative)

P: Um, they're still on that deportation list. You know? So some way like to uh, the expungement process. I guess we didn't really talk about that, but I think that that is like ... It's actually to me like a prescription for wellness. Like I think um, like clearing people's records like, when there has been evidence of ... Like you give people opportunities for rehabilitation, recovery rehabilitation.

Like clean the records, and like allow a fr- a clean slate. You know? Um it's just like ... I think it's such an injustice that like, those, that that history like, has to ... Like there isn't like an opportunity or potential for people to like, recreate themselves. You know? It's like their past continues to haunt them. There's no way they can get away from that. Um, and it has tremendous health, and mental health impacts.

I: Mm-hmm (affirmative)

P: So, that would be the other, just little thing I'd add. Yeah.

I: Yeah, so thank you. I don't want to hold you up too much lon-
