# Supplementary material for: Physicians’ perspectives on continuity of care for patients involved in the criminal justice system: A qualitative study
Source: PLoS One. 2021 Jul 14;16(7):e0254578. doi: 10.1371/journal.pone.0254578 (PMC8279398; doi:10.1371/journal.pone.0254578)
Supplement: S2 File — (ZIP) [file pone.0254578.s002.zip › Clean/Participant_19_Audio1_LJ_deidentified.docx]

I: So, thanks again for meeting with me. Um, this interview is designed to get a sense of what you know about the criminal justice system and any experiences you've had with working with patients that have some type of involvement with the justice system. And this is part of, uh, a project that's a joint partnership between [health system], the [University] and we also have some involvement from [county] on this project as well.

And to start us of, I just wanna get a very general overview of what you know about the criminal justice system. Um, could you tell me a bit about what you think of the current state of the criminal justice system in the U.S.?

P: Oh, wow, that's a very broad question. Uh, I ... Um, what I understand or what I've seen, is that the criminal justice system, um ... is somewhat dehumanizing for people involved in it. I guess that's kind of the, the sense that I get from talking to patients and, um, those who've been involved, um, but they somewhat, you know, um, obviously, kind of lose their rights to some degree. And have very little access to, um, some very important, uh, treatments such as, medication assisted treatment, um, for use disorders, etc.

Um, I, gosh ... What types of, what more information do you want? What types of, um, specifics, I guess.

I: Yeah, um, so I have, um, some criminal justice system terminology that I'd like-

P: Oh, sure.

I: ... to go over.

P: Yeah.

I: Um, so there's a list of terms, and could you tell me what comes to mind when you hear them?

P: Sure.

I: And the first is prison.

P: Yes, uh, so when I see ... hear prison I'm thinking someone who has been convicted of, a felony or something, uh, or like a longer term sentence, um, typically, uh, is what I think of as prison, I guess.

I: Mm-hmm. (affirmative)

P: Versus jail, um, and when I think of jail, oftentimes that's people who are being held prior to their trials or, um, who have been, um, arrested more, um, recently, is kind of how I, I distinguish the two in my head?

I: Mm-hmm. (affirmative)

P: But, um, that's, I don't know, if that's accurate.

I: And then what about the term, um, probation?

P: Uh, my understanding is that is, um, a ... Um, someone who has a, a sentence that they're completing outside of prison. So, it, it may be, um, that they are, are out, um, like, they were sentenced to 10 years and they were able to, um ... Nope. Now that I'm thinking about probation, you asked specifically, not parole.

Um, so, um, there's a, an increased, um, surveillance that, that happens or requires checking in if there's any other, um, breaches with the law, I guess or, um, interactions with the law, that someone may be sent to prison or back into the, um, more formal system. Yes, that's my understanding or my best guess.

I: And then my next term is parole, actually.

P: Yeah.

I: So, what comes to mind when you hear that?

P: Yeah, so, um, uh, the opportunity to, um, finish out a sentence outside of the walls of, of a prison or an incar- incarcerated state, but still requires quite a bit of surveillance and, um, potentially monitoring of some kind during that time frame.

I: So, next I'd like to talk a little bit about your background and your education and training.

P: Mm-hmm. (affirmative)

I: Um, during medical school, did you ever receive any training, whether it was formal or informal, in working with justice involved populations?

P: No.

I: And do you think that any training during that time would have been helpful to you?

P: Yes. (laughing) Very much so. Yes.

I: And so what kinds of trainings do you think would have been beneficial during that time?

P: Yeah, uh, I think one of the things that, that, um, could have been very helpful is, what are some of the, um, best practices in, um, working with patients who either were recently incarcerated, or have been, um, uh, jailed or, you know, that sort of thing. Kind of how to approach asking about that time.

Um, I have kind of sought out some of that, um ... more recently, but only after having, you know, made mistakes and other sorts of things, um, previously. So, really I think it'd be helpful to know, um, what actually happens in medical care, what actually happens for, um, people in the justice system. What does that look like? I mean it's, it's really not, um, something that I knew very much about, um, at all so, yeah.

I: Yeah, and then were there any trainings during your residency on this at all?

P: Not that I recall. Um, we had a, kind of, a community health, um, kind of longitudinal curriculum of, occasionally we'd have, you know, lunch lectures on, on various things, um, but I don't recall there being one specifically for, um, people involved in the justice system.

Um, but it would be mentioned somewhat in our, you know, um, our patients with unstable housing or, um, that a lot of times they'd experienced incarceration or justice system involvement, but we didn't specifically talk about that. And that, I think that would have been really helpful.

I: Mm-hmm, (affirmative) and as part of your training did you complete a fellowship at all?

P: I did not.

I: Okay.

P: No.

I: And then could you talk a little bit more about some of the trainings that you've sought out now, more recently?

P: It's mostly just been, um, like self-readings, so there-

I: Mm-hmm. (affirmative)

P: ... was an article, um, in one of the bigger journals, that I have saved in my inbox, (laughing) on, on, um, kind of, best practices and how to, to speak in... How to kind of identify, potentially, that, um, patients have been involved in the justice system if they don't explicitly tell you that. And how to, kind of, approach asking about, um, the health needs and how to avoid, um, stigmatizing and, um, alienating people who have been recently incarcerated, because sometimes, that is, um, that happens, um, for us and so how to, how to do that all a bit better.

So I, you know, read that article and, and have kind of sought out some, um, books and other sorts of things like that to just know a little bit more, but it's not enough, 'cause there's so many other things that, (laughs) I'm trying to keep up on too that I just, um ... sometimes it takes a back seat.

I: Yeah, and so ha- have you ever gotten any training from an employer where, whether it's a past or current employer, on this at all?

P: No, no definitely not.

I: And so now, like, during your day-to-day visits with your patients-

P: Mm-hmm. (affirmative)

I: Are you asking them whether or not they have been, or are currently involved with the justice system in any way?

P: Um, not routinely, so not with every single patient.

I: Mm-hmm. (affirmative)

P: Um, I ... I, I, would say that, um, a lot of patients are, um ... fairly, uh, do volunteer that information when asking about, you know, if I just say, “when was the last time you, you know saw a physician?” Or, “tell me about what, um, looks like you've been off your medications for six months, tell me a little bit more about that?”

I: Mm-hmm. (affirmative)

P: Um, or, “it looks like it's been little while since you've been here, um, is there anything I need to know?”

Then people will disclose that, um, but it's not routine, um, part of our, our, uh, screening process and I guess I don't approach it with every single patient, yeah.

I: Mm-hmm. (affirmative) And then could you explain a little bit about once you have that information, and maybe an example might be a good way to go about this, but how do you use that information to inform your treatment plan for a patient?

P: Yeah, um, so ... If I have, um ... I had a patient ... So occasionally we'll have patients who we know are going to be headed into, um, prison or who have an upcoming trial or something like that. We will, um, try to tailor the treatment to make sure that they, um, whatever we start, they're gonna be able to continue when they enter, um, the system in some way. Um, or trying to figure out, you know, like, what our follow up plan might look like or, you know, tailoring to that aspect. And then similarly if we have, um, someone who has been, you know, recently incarcerated as far as testing for infections, um, tuberculosis, those sorts of things. Making sure that we're tailoring our, tailoring our screenings based on potential exposures.

Um, and then also, uh, trying to help put people into services, um, and other types of community organizations that might help with the re-entry process or, um, finding housing or, um, in, you know, continuing insurance and, and, those sorts of things.

I: Mm-hmm. (affirmative)

P: Yeah. So, it depends on I guess what the ... (laughs) information that we have, yeah.

I: Yeah, and then you mentioned, um, about follow-up planning-

P: Yeah.

I: ... with the patient? Um, are you following up with patients while they're incarcerated or do you make a plan for afterward? I'm kind of wondering what that looks like.

P: Right. So, oftentimes it's, it's after. Um, I haven't had, other than pregnant patients, being able to, kind of, leave for visits.

I: Mm-hmm (affirmative).

P: Or being able to follow-up, kind of, while they're, um, in prison. Um, and so, sometimes for, for pregnant patients we would, you know, they ... there would be some follow up plans from that aspect, but most of the time it's, you know, following up for after. And a lot of times if people know that they're probably going to be in prison for years, we can't set up a specific plan, and, um, just try to document the best we can, uh, to hopefully continue the, what they need when they're, um, incarcerated, yeah.

I: And so, could you tell me a bit more about some of the questions or if a vol- a patient volunteers that information, that they've been involved with the justice system, or there's something in their history that, like, makes you think that there might be a possibility-

P: Mm-hmm (affirmative).

I: ... could you talk a bit more about some of the follow up questions that you might ask them?

P: Yeah, um, so, one of the things that I, um, always ask about is, um, like, safety as far as, um, what the, um, you know, whether or not they're in kind of, um, a living situation? That they're, you know, at risk for violence or other sorts of things, like what, what is their current, kind of, um, physical safety.

Um, I try to ask, um, if there's anything that I should know about. That would be helpful for, um ... uh, infection risks. Um, for, you know, substance use, for connections with community members or family members who might be good supports, um, from that aspect, um.

And then, um, I guess I don't, I ... don't typically ask, like, what the, um, reasons are that somebody was incarcerated. I don't think that's really any of my business, unless they, it has health risks, um, for them. Like, if it's substance related conditions, if, or, you know, drug possession or something. If there's ways that we can help support sobriety or, you know, from that aspect. But, um, I guess I, um, don't usually delve too much into that unless it's volunteered. Um, and then asking, yeah, there's anything that they think it would be helpful for me to know, um, to he- to help, um, optimize their care, yeah.

I: And are there any challenges that you see to talking to your patients about this?

P: Yeah, I think one of the biggest challenges that I have is that I, I want to try to convey that it's important, um ... That I want to create a relationship. And that I want to try to find a way to convey non-judgmental, um, approaches and that, that, you know, a history of incarceration isn't going to, like, change how I see them as a person. And, like, I really wanna still create a therapeutic relationship. And, um, and I know that there, uh, a lot of times is a lot of stigma and people do experience that.

And so, trying to figure out how to convey that is sometimes challenging. Like, what words to use. Or, you know, how to know kind of what, what to ask. Um, and how to ask it. What do I need to know, you know? And it's just like, trying to balance all of that, um, can be really hard, I think, um. Yeah, yeah.

I: Yeah, and then, on the flip side, are there any benefits that you see to having this information and talking about your patients? Or talking with your patients about this.

P: Yeah, well, I think so, because I think it helps gives us a really important context, to, you know, all of the things that we know, and, uh, determine health exposures to, um, trauma and stress and, um, also trust, you know. Um, people have experienced, um, being, um, you know, judged or, um, discriminated by the system that, that, um, can be hard to, to get by in if we don't know that, that's something that we need to be aware of as providers.

Um, and I want, you know ... I think there's a benefit then to being able to talk openly about that. And to, to share, that you know, we wanna work together and try to, try to come up with a treatment plan that is gonna be sustainable and can help optimize health, yeah.

I: And so, could you tell me a bit more about your patients that you see on a day-to-day basis, like, what your overall patient population looks like?

P: Population? Yeah. So, um, in our clinic we see about 80%, um, Medicaid, um, and Medical Assistance, um, insurance, here at this clinic. And, there are a few patients who have no insurance or kind of under-insured, but because we're not an FQHC we don't have, like, sliding fee. And so there-

I: Mm-hmm. (affirmative)

P: ... are many fewer of those patients, few private insurance patients. Um, very few Medicare, with the exception of, like, social sec- or, you know, SSI. Um, because of, we just, we don't have a, a very, um, old population.

Um ... 70% or so, at our clinic are, um, African-American, multi-generational African-American. About 15%, um Hmong, um, either f- kind of, first, second or third generation. And, um, and then another, like 10, um, percent or so, uh, U.S. born white, um, patients.

I: Mm-hmm. (affirmative)

P: Um, and, my clinic day I see, probably a third, um, children under the age of 18 and probably two-thirds adults, um, on average. I guess it really depends. (laughs) Some days I have no children, some days I have mostly kids, um, most of the ... Our pregnant patients are seen by the residents. So, I'll only see, you know, occasionally on the, um, walk-in if somebody needs something, acutely. But I don't have any, um, patients I follow for their pre-natal care, uh, regularly. Um, about half male and female. Mm-hmm, (affirmative) yeah.

I: And then for patients who are from, um, racial or ethnic minority backgrounds, in particular, are you noticing any barriers or challenges they are having in terms of accessing care?

P: Um, yeah, I, you know, I think, um, one of the things that, um ... Yes, I mean so, um, the majority of our patients are of an ethnic or racial minority, um, so, majority minority, I guess. (laughs) And, and so, you know, getting access to our clinic, maybe a little less. But I think, um, we have a disproportionate, comparatively, number of, um, white providers. And I think that that makes a difference sometimes and, um, in connection if we are, um, not, kind of, explicitly understanding that sometimes there's mistrust, um, based on some historical, um, obvi- obviously all of the historical trauma that the medical system has done to people.

Um ... I'm trying to think, like, specifically otherwise. I mean, I think it's harder for us to, um, find, uh, culturally appropriate mental health care, um, for, uh, patients who are not, um, of the, uh, majority white background, um. A lot of times that cultural congruency or, um, uh, having a provider who understands some of the ... s- particularly when it comes to mental health, can be very helpful. And, and most of the mental health providers, um, are white. So, that's a challenge, um, from that standpoint.

Um, and then some, some, uh, language, um, uh, for our, or Hmong patients. Sometimes it's a lot harder to get them to, um, have access to, um, care because of interpreter services or not being able to make appointments, um, because they can't themselves call to get the appointments.

I: Mm-hmm. (affirmative)

P: But, um, we do have a community health worker who is, um, Hmong speaking who helps, uh, patients make appointments and, and other sorts of things like that. Yeah, so, I'd say that's probably the mostly, um, the biggest challenges that I see, for access.

I: Mm-hmm. (affirmative) So, now I'd like to dig a little bit more into your patients who have some type of justice system involvement.

P: Yeah.

I: In particular, and what that experience is like for you as a provider?

P: Mm-hmm. (affirmative)

I: And, are there any ways that you're seeing that their justice system involvement is in some ways, possibly impacting their access to care?

P: Yes. Um. (laughs)

I: Tell me more.

P: Yes, right. Um, yeah so, um, I've had a number of patients tell me that, um, their medications were taken away when they were, um, arrested or brought into custody, for some reason. And then they were not given those medications again when they were let out on bail, or when they were released from, um, uh, custody. And then their insurance wouldn't cover another fill, so they had to wait until the next month to get their, um, prescriptions for medications, um.

Most of the time that happens with controlled substances or, um, something restricted, but I've had that, patients tell me that, that would happen even with their Metformin, or other types of, um, generic, not controlled medications. Um, and that, obviously is a problem when they had medications on them and then were unable to get them back, at the end.

Um, a seizure of, of, um, money or, um, things of value that were on their possession, that they didn't get back when they left, impacted their ability to, um, pay for their medications when they were due, for their co-pay, for their bus fare, for all sorts of, um, financial burdens, from that standpoint. Um, for some of my patients, they are arrested and then lose their jobs. And then their insurance is gone or they're unable to, yeah, pay all the, um, fees or bus fares or that sort of thing. Um, missed appointments, um, on, um ... Some places are, are less flexible on missed appointments. And so, if they happen to be arrested and, and miss a scheduled appointment with a consultant, they're unable to schedule another one. Um, and that's a huge problem.

Um, trying to think of ... others, I feel like there's lots of examples that I hear, of how it's negatively impacting care.

I: Mm-hmm. (affirmative)

P: Um, I don't know that I have any stories that have been positive, um, for people. I don't know that I've had any of my patients who have, have been incarcerated and come out healthier than they were when they went in. Yeah, yeah.

I: And then have ... Do you ever communicate with probation officers or parole officers or the courts at all?

P: Um, yeah, good question. Um, occasionally, are, we are, um, asked to write a letter saying that someone has been coming to their appointments for, um, uh, chemical dependency or addiction type treatments, an- and to, um, you know, share, like, lab information, etc. Um, but, otherwise I ... Unless they're actually at the visit, I haven't had many phone calls where I've been asked to communicate with, um, parole officers or, um, probation officers, yeah.

I: Mm-hmm. (affirmative) And are they coming to visits and ...

P: Not usually.

I: Not usually?

P: Yeah.

I: Are there ... But are, do you have experience where there has been-

P: Yeah.

I: ... a probation officer present?

P: Yes.

I: Could you talk a little bit more about the context of-

P: Oh, gosh.

I: ... how that happened?

P: It only happened one time.

I: Mm-hmm, (affirmative)

P: So, now I'm trying to think of what, what the actual context was. Um, I think it was a patient who, um ... who needed a, a drug test for something. Um, gosh this was a couple of years ago. I don't remember the, the details, yeah.

I: Mm-hmm. (affirmative)

P: Yeah.

I: And then, are you ever getting patients who are referred to you, specifically, from the criminal justice system?

P: I don't believe I ever, have had that, yeah. Um, I've seen patients, like, kind of, right after they've been released.

I: Mm-hmm. (affirmative)

P: But, they had been established patients here already. So, it wasn't like, a referral-type situation, yeah, yeah.

I: And then aside from justice system involvement, what else are these patients dealing with socially that you're seeing?

P: Yeah. Um, so housing challenges, for sure, is one of the biggest ones, um, for a lot of people, um, their, their family is on, um, housing support. Such that they ... If they're, uh, they can't live with them, because it ... they're in a setting where they're not allowed to have someone whose, has been formally incarcerated or has a felony charge or something like that.

So, there, there really isn't a stable place for them to go and, kind of, get back on their feet. Um, so housing is definitely one of the things, and there's, you know, obviously, restrictions on whether or not people can get apartments, based on their history. Um, not restrictions, but it's one of the questions that's asked, and so sometimes people are not offered, um, housing, from that standpoint.

Uh, financial difficulties, for sure. Getting jobs, um, and, um, I would say, um, uh, mental health and substance use disorders, um, are frequently, um, seen in my patients that have been involved in the justice system.

I: Mm-hmm. (affirmative)

P: Yeah. Um, stress, around, um, custody of children. Or, um, visitation and, um, transportation. I feel like it's all of the things, yeah.

I: Could you talk a little bit more about the substance use and mental health needs that you're seeing from patients?

P: Yeah, yeah. Um ... So, often, the patients I've seen with substance use disorders, who have been incarcerated due to, um, uh, substances, um, continue to have needs when they leave, um. And, um, I haven't had any, um, uh, like opioid overdoses, or anything that I know is more common as people leave, uh, prison or jail, um, with an opioid use disorder. Um, but I haven't personally had that with any of my patients. Um, oftentimes, um, I encounter patients who are really trying, um, to maintain sobriety, um, but have so many, um, stresses and social, um, contacts who are maybe still using, um. Or, just kind of restricted availability to, um, getting treatment regularly, that it's, it's hard to maintain sobriety, with all the stress and, um, limitations at getting treatment. Um, financially and otherwise, um ... Yeah.

And I would say that most of my patients, who, um, have been involved with the justice system and have other mental health conditions, um, uh, like, bipolar, schizophrenia, um, uh, independent of substance use disorders. Um, tend to be, not necessarily well-controlled when they are released and then trying to get them re-established with services as they're coming out is a challenge. Um, yeah. Just with, um, less stability.

I: Mm-hmm. (affirmative)

P: Yeah.

I: And what are some of the physical health needs that you're seeing-

P: Mm-hmm. (affirmative)

I: ... among jus- justice involved patients?

P: Yeah. Uh, the primary, um, ones we're seeing is diabetes and high blood pressure. Um, and kidney disease, kind of tend to be the, um, three that, uh, frequently we'll see, um, uh, as patients are leaving. Um ... pain, chronic pain as well.

I: Mm-hmm. (affirmative)

P: Um, is another one that is ... yeah.

I: And then, are there any resources or services that your patients need, that you're finding just isn't available to them?

P: Um ... I think it depends.

I: Mm-hmm. (affirmative)

P: Um ... like I kind of, um, mentioned earlier, the ... Hou- I mean, housing is, is, one that I think is, uh, can be a big challenge. No- obviously not every patient has, um, housing issues. But that's one that, um, can be really difficult, um, because of the restrictions on programs and, and how to help people find a stable place to, to be the, um ... May help with recidivism right? Um, so, yeah. So, housing is one.

Um, some patients are able to, to access some of, you know, resources. Um, but, I'm, we're ... I'm just not as aware as, of what's available for, um, patients who've been involved in the justice system, yeah.

I: And then, thinking broadly, are there any changes to healthcare delivery that you would suggest, to better meet the needs-

P: Mm.

I: ... of justice involved patients?

P: Mm-hmm. (affirmative) Yeah, so, I think, um, access to, um, buprenorphine, methadone or other sorts of medications that's the treatment for opioid use disorder would be, um, good or, um, naloxone or what, you know, um, um, whatever we need to, to do to help with, um, with that. To actually have access in, in the prison or jail systems would be really good. I think that would help, um, uh, with continuity when people leave, um, jails and prisons.

And then, I think it would be great if there was a way that we could have more communication, um, with people providing the care in, um, prisons and, and jails, um. S- I don't know if there's like, some, like, way of getting more connectivity within electronic medical records, or ways that it can be a little bit more seamless from that aspect, Sometimes it can take weeks to get records, if we try to get them. If they're, if we get them ever at all.

I: Mm-hmm. (affirmative)

P: Um, and, you know, potentially some sort of communication, um, when people are released, of like, their current medication list. Or what types of screenings and those sorts of things that they received. Or a phone number to call if we have questions, for that first clinic visit after someone has been released, might be helpful.

I: Mm-hmm. (affirmative)

P: Um.

I: In terms of the records of like, to ask a follow-up question-

P: Yeah.

I: .., are these records that are coming from prison, or some of the local jails? Or both? And, does that kind of vary based on where you're requesting information from?

P: Yes, right, yeah. It depends. Um, yeah, sometimes, sometimes we really don't get them.

I: Mm-hmm. (affirmative)

P: Um, yeah. And the short term, kind of local jails, I mean, sometimes a ... patients will go, like, three or four days without any meds at all, right? The things they're supposed to be taking, and then, and then they can't get 'em which, I know it's really frustrating for them, but, um. It can lead to some terrible withdrawal and other sorts of things, but, um, yeah.

As far as, like, getting the records, it varies, um. U- Usually, if, if we're clear that, um ... Prison is usually better at getting records, on average. But, it still can take a while.

I: Uh-uh.

P: To, kinda, get them, yeah.

I: And then, were there any other changes to healthcare delivery that you were gonna suggest, in addition to those?

P: Yeah, um, I mean I think, I think, um, access to mental health and trauma informed, um, therapy in, um, in prisons and then, um, like I don't think, like, solitary confinement should be a thing, so. I don't think that's related, or exactly what you're asking, but it'd be great if that was not a thing. (laughs) Um, for people's well-being, yeah,

I: So, thanks again for your time today. Before I officially wrap up, is there anything that I didn't ask you about today that you'd like to add?

P: Mm. No, I mean I'm just looking forward to some action. Some help down the road. So, I hope that, um, that, uh, this'll be, um, will come to some sort of toolkit or something for us. And, additional training, 'cause I think that would be really great. Um, clearly I don't know very much about the justice system (laughs). So it'd be really helpful.

I: All right, well, thank you.
