# Supplementary material for: Physicians’ perspectives on continuity of care for patients involved in the criminal justice system: A qualitative study
Source: PLoS One. 2021 Jul 14;16(7):e0254578. doi: 10.1371/journal.pone.0254578 (PMC8279398; doi:10.1371/journal.pone.0254578)
Supplement: S2 File — (ZIP) [file pone.0254578.s002.zip › Clean/Participant_1_LJ_deidentified.docx]

I: Okay, so thank you again for taking the time to meet with me today. Umm this interview is part of a larger project between us and the [University] and [County]. Umm we’re examining broadly the connection between health and criminal justice involvement and the goal of today’s interview is to gain an understanding of what you know about the criminal justice system and how it may impact your patients. Umm and so I want to begin by getting a general overview of what you know about the criminal justice system. And to start us off could you tell me what you think about the current state of our criminal justice system in the US?

P: That’s a pretty broad question. I think the criminal justice system in general has umm struggling with a lot of issues that have to do with umm the social difficulties in their setting. I think there’s umm a lot of background that biases the criminal justice that has to do with with race, it has to do with poverty, it has to do with who comes before the justice system. Even though we provide help with the defense system, it is not equitable and the distribution of of umm consequences or umm the the legal consequences are not necessarily equitable throughout the socioeconomic umm spectrum. There’s overrepresentation of black youth in the criminal justice system for criminal ah activity that compares to the similar activity from other more affluent groups and other ethnic minorities and it’s also true there’s a lot of concern about the overrepresentation of people with mental illness in the criminal justice system because of the nature of their behavior and the lack of other resources and services up front that result in the legal consequences.

I: Thank you and so next I’d like to discuss some criminal justice system terminology, could you explain to me what comes to mind when you hear the following terms?

P: Okay (clears throat)

I: The first is prison

P: So (short laugh) this is not a ?(psychiatric association)? so I will be more logical. So prison to me means a longer-term sentence of somebody who has been convicted of a crime. It’s a place where you are umm incarcerated away from society with subsequent umm capacity to be reintegrated, and that’s a word I use loosely, umm according to your behavior and according to your sentence.

I: And how about the term jail?

P: So to me jail is a more temporary ah placement until prisoners get processed they get the legal charges finalized and they appear before the judge and they come and get sentenced.

I: And could you tell me a little bit more about the similarities and differences that you know in terms of the difference between jail and prison?

P: Well I think the similarities are that people are taken away from society, form their family, from their work from their place of residence, ah they give up a lot of their umm their immediate rights, umm in terms of access to things, and their life is controlled by an institution that essentially regulates who they see, when they see them, where they go, and how they get there. Umm So they’re both places of umm… prison where you get withdrawn from society, they’re supposedly because of a justifiable legal offense that merits your your serving time for being deprived of your natural freedom.

I: And what comes to mind when you hear the term probation

P: So probation is something where someone can return to their social environment under stipulations or conditions that are dictated usually by a judge or court, it’s either a way of umm transitioning people to their community while still having (coughs) excuse me, some legal hold in case that they reoffend. It’s also times an option umm for people who don’t merit longer term institutionalization in the prison system umm to return back but with conditions and caveats about consequences if they violate the terms. Umm so it’s sort of release with conditions attached to it which you need to meet either meeting with probation officer, umm avoiding contact with the police and not reoffending.

I: And how about the term parole?

P: So probation and parole are never particularly clear to me the distinction between the two of them. Umm so to me probation really is a more active supervision with shorter term conditions and parole umm is refers to longer term sentences but I’m not really very clear on the distinction.

I: So next I’ll dive into some of your educational and training background.

P: Hmmh

I: Umm during medical school did you ever receive any training whether that was formal or informal on working with justice involved populations?

P: No, none

I: Umm is there any training that you think would have been helpful to you during medical school

P: I I think in when we’re looking at public health umm and medical school is a good opportunity for people to learn about the legal justice system and how it relates to the population because of all the consequences in terms of social, social consequences and health and health complications from the social consequences. So I, I really think in the public health system in general, I think when people come into the mental health field there’s a lot specifics that have to do with our involvement in the criminal justice system that we try to teach ah in the context of residency specialty training because we work ahm with a lot of civil commitment laws and so that’s a lot of the ah regulations around the institution of care and treatment against people’s will so at that point it’s essential that people learn those consequences but I think earlier consequences those, those facts, but I think earlier on in medical school a general sense for the impact of ahm the legal system and healthcare in general, the interface between the two of them is pretty important.

I: And so you spoke a little bit about training physicians or who are in residency, did you yourself receive any training during residency?

P: I did.

I: hmmh Could you speak to what that training looked like what it was...

P: The training ahm was, through my training through my training a number of years ago is relatively informal and based on specific mental health issues like (inaudible), civil commitment, in terms of civil commitment, and those were part of lectures series referred to as forensic psychiatry not particularly vigorous when I was a resident many years ago. I think we do a much better job now.

I: And if you completed a fellowship did you receive any training on this topic during your fellowship?

P: Umm my fellowship was in ahm so I guess [School of Medicine and Public Health], so there’s only again the reinforcement of the issues about the umm the civil statutes around commitment. It was more hands on dealing with particular cases, not lecture or presentations.

I: And how about any training at your current here at [Current Employer] or past places of employment?

P: So it depends on what you call training, we certainly have ongoing continuous medical education that touches on some of the general issues. I think a lot of the ongoing education has to do with our continuous contact with the court system. We’re we’re ahm exchanging information back and forth about what’s working, what’s not working, what we’re supposed to do, and hasn’t worked, case based discussions about ahm about what’s missing what’s lacking, we provide ahm services throughout the spectrum so we provide services such as (inaudible) we have people from the jail sent to us for care so uh a lot of discussions with our county attorneys who are an integral part of the hospital care, uhh case conferences, etcetera.

I: And so thinking about your day to day visits with patients during visits do you ever ask patients about their current or past involvement with the criminal justice system?

P: I always do during their initial intake

I: And how do you gather that information? Like what types of questions are you asking them?

P: so you I umm generally we’re asking about chemical health, we pursue questions about any previous history of commitments we do so also with um mental health, in the social history we usually ask about their social status, family, ah current stressors, and in the context of talking about stressors and duties we raise the question of in the current or past involvement with the legal system.

I: And how does that information influence your care of that patient?

P: I think it it influences my care ah in terms of understanding what people’s ah current circumstances, whether their um having to meet a number of requirements with supervised probation for example and it also raises questions with some of ahm situations about history of violence ahm violent crime in particular in terms of how we provide for care especially in inpatient settings

I: and so thinking about your overall patient population not just those who are involved with the justice system, um how would you describe them? Just some general patient characteristics

P: Well I work at the [hospital] so the majority of my patients have a history of some type of mental illness, likely psychiatric symptoms that bring them to my attention. Umm the bulk of the patients that I see have multiple psychosocial stressors ah that are not only relevant to their life in general certainly to the illnesses that they’re dealing with and many of them are struggling with a lot of very acute socioeconomic umm stressors.

I: hmmh. could you speak more to that?

P: yeah, ahh people who don’t have enough money to make ends meet, people who require public assistance, ahh people who don’t have access to medical care usually because they’ve lost their insurance and need to get (cough) reestablished with the system which is not simple for them to follow on their own. Umm we see a lot of people going through transitions in terms of their families, either going through divorce, separation umm having children taken away or being separated from their children because of chemical dependency problems or (inaudible)

I: And you spoke a little bit about insurance status,

P: Hmmh

I: How would you describe like your general proportion in terms of what types of insurance people have?

P: The majority of people that we serve have Medicare Medicaid. Umm yeah so (cough) probably I hear different numbers from the administration but I always think in terms of the people I see at least half, half of them do ah so the so called public programs we have a significant percentage of people who have no insurance coverage, ahm many of whom you hope will get enrolled for public assistance we certainly have a small group of people who are not insurable either because they’re undocumented although there’s some emergency programs umm and then we see a smaller proportion of people have some form of commercial insurance especially around the trauma areas with ah accessing our service because of that… so probably thirty, forty percent of people with some brand of commercial insurance.

I: And how would you describe the disability status of most of your patients?

P: Ohh boy umm…it’s it’s hard to answer that without providing you with identifiable ah details (cough) but it’s not I’m not concerned about that. I primarily function in the general surgery medicine floors doing consultations so so that means that umm my mix is probably a little bit different than people who work in the clinic. Umm so the the bulk of the people that I see have at least some temporary form of disability but the percentage that have long term disability either umm established or seeking long term disability is probably I would guess around 15, 20 percent.

I: And have you noticed any particular challenges or barriers faced by patients from particular racial or ethnic minority backgrounds?

P: that’s an absolute yes, how to quantify and what to tell you about it is a little bit different, so there’s um two ways to think about it there are cultural barriers that have to do with some of the umm groups of people that we see, cultural barriers that don’t just mean scientific scientific (inaudible) questions of trust, questions of ahm continuity of care there are subgroups of patients who (cough) not particularly good at the concept of long term term care taking medications long term ah because of where they come from and what they’ve experienced. Umm we see an incredible number of people who require translators and the interpreter system in our hospitals are always challenged and quite impressive in many ways. So when you deal with people when they see a translation that creates other barriers which have to do with not just the language but you can’t translate culturally. So for example if you’re dealing with ahm somebody who would like a lot more close personal assistance, somebody as opposed to somebody who wants you to treat their problem today and move on. So we have a lot of trust issues which have to do with the community we serve, people of color in general have had bad experiences with county hospitals in other states and the ones who are new to our systems think of us as a county hospital and therefore when either they’re not getting the care they feel they should get or there are decisions that are difficult that they umm seem distrust falls in to place. So for example number of years ago umm the uh…the the organ ah donation system kind of highlighted this for me probably fifteen years ago where the question really was, how do you approach people to ask if their loved ones will donate their organs if you happen to belong to some ethnic groups, minority groups, people of color there’s the question of whether people are trying to give my kid’s organ prematurely because they don’t care. And of course for providers that becomes a painful realization or a perception of their distorting what we want to do but that’s a reality that people have to deal with. When I look at the numbers of people that were placed in hospice right now I think hospice care umm the numbers of people of color is quite smaller and I don’t know the answer to that. I’ve asked it and the question is is it because people of color are doing a better job of taking care of their own at home? which certainly has been a cultural value in some of our cultures some of the Latino descent and that was how I grew up, or is it that they don’t trust the argument of somebody coming and saying your loved one will die within the next six months we can put them in in hospice where we’ll provide them a comfort so we have a lot of challenges that have to do with that. Ahm I also think that we see an overrepresentation of people of color in in the poverty groups and I think poverty is clearly a big challenge in terms of not only access to care maintaining yourself having an adequate diet, being able to afford copays even though they seem small so I hear people having to stretch fifty dollars over the course of a month uhh for any extras and to me fifty dollars is one days waste when you think of all the things that people of means have and they do. So there’s economic barriers and I personally tend to think of ahm economic difficulties as being people, poverty is overrepresented in people of color so you add poverty to the cultural issues it becomes a pretty different issue so your struggling with uh providing some level of preventative care in terms of health maintenance in populations that have to worry about will I have enough food to eat? People who don’t have food sufficiency or don’t know if their children will have money to be able to join the soccer team or whatever team, the football team. Umm there’s a lot of hard choices that people make on their own.

I: Okay and so shifting to your patients with justice system involvement in particular, umm in treating those patients what has that experience been like for you as a provider?

P: So um we see a fair, fair is probably not an adequate term, but we see a number of people from the jail system who end up on our medical floors and I think there’s a loss of confidentiality that makes my job hard. It is pretty difficult if I see somebody in umm the surgical floor who comes in umm because of some form of trauma they’re being constantly watched by a deputy. You can’t always ensure privacy or safety so I am somewhat limited in terms of how much I will find out about what transpired what brought em here and what their facing. People who have not been charged yet uhh I have to be very careful to not extract confessions from them since I don’t work for the justice system in front of the deputies who are taking care of them, and I’m very respectful of people’s right to due process so it creates a tension in terms of how do I provide mental health care while at the same time not exposing them to undue legal consequences regardless of what I think of what they’ve done. It also creates a challenge for physicians in general. Umm when you see people who have committed acts, illegal acts that you have ah a bad reaction to, people who have done quote bad things and you need to leave your judgment behind you and provide the best care that you provide anybody, so it is an ongoing exercise for anybody in our system to really be able to distance themselves from people’s behavior, actions, or legal events and provide compassionate care and that becomes difficult so we see people who are accused of ahm sexual abuse of children, people accused of rapes, domestic violence and we’re human beings and you have to struggle with your own reactions to that. We see people who challenge us because of ah… racial and ethnic differences. Umm we see people who respond to you with bigoted responses who don’t trust you because they perceive your ethnic background to be different than theirs and while that is less often I think for me the the bigger issue is umm making decisions about what they need, trying to sort out when people are trying to, quote, avoid their responsibilities and the same arguments that people make in the public in general about umm so ah criminal justice involved people needing to pay for what they’ve done, I suppose our job of understanding where people are at and what their emotional needs are. Ahm we provide care for people in the jail system and that’s somewhat difficult in that we can only reach people who want help in the jail system you can’t force treatment you can’t intervene even when there’s an emergency and those people end up having to get transferred (inaudible) care and then you have to worry about all the legal ramifications. So umm I like to tell patients that what they tell me is that I don’t work for the, for the sheriffs I don’t work for the criminal justice system but whatever they tell me will end up in the record and that at some point can be subpoenaed by the court. So I’m not extracting information for the sake of the prosecuting attorney but whatever they tell me will end up in the chart and that sometimes limits my access to information that I think is crucial in terms of understanding them.

I: And so you spoke a little bit about communicating back and forth with attorneys in the court. Could you speak to some what information is shared and how that process works?

P: So there are the two main areas which we share information with the court is when we’re requesting ah involuntary commitment of people to the hospital, somebody who comes to the hospital needs ah psychiatric care because they present a danger to self or others and they don’t want to stay. So in that case there’s a petition to the court for what’s called a commitment and that entails revealing information about in which ways the person presents a danger, umm what they have done in terms of accessing care, how they have failed or not in the past, and the absence of any other less restrictive options so that includes a lot of their medical history relevant to their commitment or chemical use. Umm exchanging information on chemical use. Umm when we’re seeing somebody from the jail ah who comes in with healthcare issues the only ah communication I have with the court system at that point is about whether it’s safe to return to the jail and what they need whether they need to stay in the psychiatric unit. The details of what they exchange with me is irrelevant in that communication umm unless the patient has been involved in the court process that’s called the rule twenty that is basically a umm a request to establish people’s capacity, in in that case we transmit to the court information about whether the person has capacity to participate in the trial, ah to understand the charges whether they need umm further psychiatric treatment or if they’ll never be able to understand their processing or not they’re not going to be able to participate in the court system process umm … we have at times voluntarily released information to attorneys and probation officers from patients for information that I feel will be helpful in their sentencing or in their (inaudible) but short of that we don’t provide information. So a patient comes in the hospital they’ve been picked up by the police umm they were altered acting bizarrely and I know from the records that they have been using an illegal substance; that is not information that I share with the legal system unless there’s a court order to obtain their records which include the results of toxicology screens etcetera

I: And then aside from justice system involvement for these folks that are either in jail or on probation and stuff like that (P coughs) umm are there any other things that you see these patients dealing with socially?

P: Well that’s a broad question so it’s it’s hard to distinguish your roll at work from your personal umm perceptions so what I see people dealing with socially has to do with people who have been incarcerated have had serious crimes ah essentially umm being deprived of the opportunity to to really umm umm be umm restored to full social function. Umm Being unable to get housing because of previous felony charges having difficulty accessing jobs and essentially being thrown back to a situation that will ah create circumstances that will not facilitate somebody being rehabilitated instead quickly going back to the behaviors that they have understood before, umm, will either give them comfort in an unhealthy way or provide ‘em with money for their survival. Umm so they have access to services difficulties with access to jobs, access to housing and frequently ah return to the same communities where their problematic behavior emerged so if I have a problem with recurrent charges relating to umm substance use I go back to the neighborhood where all of my addictions get triggered and that doesn’t provide a setting that provides good access. The transitions in and out of umm legal settings are very difficult and I don’t perceive as a I personally have a feeling that we don’t do a great job with that we’re having discussion about this a lot with transitions from just jail and we will see somebody there and all the sudden they’re gone the next day so if they have a need for psychiatric care there’s nothing you can do about it so there’s a lot more efforts I think in our county to try to identify those needs early on to try and create referrals or assist in transitions it’s challenging and I think in many ways. If it’s challenging in a major metro area, you can imagine that in more distant areas where there’s less resources people are not are not getting the help they need. So you get released. You have nowhere to go. You have no money. You have no medications and that’s when we see people in the emergency room frustrated because they’re not taking their medications their health has gone to hell umm… yeah

I: Could you speak a little bit more about what their dealing with medically… in terms of some of their physical health needs…

P: Yeah, yeah so my view is biased because I deal with people with mental illness and we know that the prevalence of medical problems in psychiatric patients is huge. Psychiatric patients die on the average ten to fifteen years earlier than somebody of their same age who does not have a psychiatric disorder so it is rare to see somebody who’s had a chronic persistent mental illness for more than fifteen years who’s not struggling with long term health issues including diabetes, hypertension, vascular disease, ah morbid obesity, hyperlipidemia all of the consequences of ah their lifestyle and or their treatment. So in our system umm most of the patients that we see have need for ongoing primary care in the old days we used to say if your young, you check in once in a while when you have problems. You don’t start getting regular visits to doctors until you get older. Our patients need ongoing access to medical care and either they don’t have the wherewithal in terms of their wellbeing to seek it out themselves ahh or they can’t afford it so when I look around and you think again of the social determinants of health if the the um legal system the um penal system is overrepresented there’s an overrepresentation of people of color and if there’s an overrepresentation of people who come from lower socioeconomic groups with higher rates of poverty you can assume that a number of these people will have some of the same public health challenges in the long run which have to do with um diet and health, diabetes, hypertension, chronic pain syndrome

I: Could you speak a little bit to the mental health and substance use needs that your seeing among your justice involved patients?

P: huge (short laugh) umm the the people that, this is become a a politicized issue um that has led to some not so helpful solutions but there’s no doubt that with the deinstitutionalization of psychiatric patients many years back people got returned to the community. We have failed to provide the support networks and systems that people need including housing, ongoing care, outreach to them so many of these people end up either destitute, homeless umm or in less than supportive environments so the rates of um substance use the rates of criminal encounters frequently um misdemeanors and just bad behaviors about um disturbing the peace or stealing, minor things, is quite common. But we know that in our state there appears to be an overrepresentation of psychiatric patients in the jails and some of them are not necessarily um minor issues. We do see some felonies. The the challenge of the question is that there are some people who have legal consequences that are really not directly related to their mental health, so I can end up with um an act of domestic violence heaven forbid that is not related to the fact that I have panic disorder um and how do you sort out where the interventions for the mental health have a direct impact on the rehabilitation or restoration of more acceptable behavior. Um we know that people who have had early exposure to trauma have all kinds of medical health, psychiatric consequences and medical consequences, psychiatric consequences and behavioral problems that ah result in criminal, may result in aggression or criminal behavior and those sort of things that you can’t change by time somebodies thirty to forty years of age or at least not very efficiently or not as easily. So when you have characterological personality umm factors that have at the outgrowth of social determinants in trauma when you’re young, that is pretty hard for any system to respond to that in a way that’s more rehabilitative so that’s a challenge, but I think if you just focus on the people who have ah chronic difficulties with substance use and mental health issues you reduce the jail population certainly by a significant percentage and that’s a challenge for all jail systems they’re overrun and um it’s not necessarily the most efficient place, the most restoring place for people with mental illness to be taken care of.

N: I’m sorry to interrupt. It’s 10:40, just so you know.

I: Okay thank you. Could you speak to and provide some specifics about the psychiatric conditions that you’re seeing as someone who doesn’t have a background in psychiatry I just want to make sure that I’m understanding

P: So in in my environment we primarily deal with chronic and persistent mental illness that means people who have umm a long term illness that is not short term related. So for example you see a lot of people with just recurrent major depression, but those patients many of them are able to be restored to normal function after an episode of depression. Um we see a fairly large portion of people with schizophrenia and bipolar disorder who essentially um have their symptoms managed and controlled. We engage them or try to engage them in psychosocial rehabilitation programs, but these are not necessarily people who end up um going back to school and getting ah degrees I’m not saying that that doesn’t happen but we have a long population of people with disabilities that we talked about before who really need to be in a restorative environment where the medications are provided where they get um taught how to interact with people, get taught how to do interviews so they can get a job and try to get in into some type of self-sustaining long term job so this is a a lifelong challenge these are not people just coming in with an isolated bout of a problem, like if you had pneumonia you come in get an antibiotic and go home. You’re done. Um most of the people we see have recurrent hospitalizations. They’re back at some point. They’re in the, quote, system um and try and optimize their capacity to function outside of the hospital which is more as I said before we probably have fallen short of providing what people need it’s not cheap to provide what people need in the community, unless you have your own means then you can create for yourself but that’s not generally the population that we serve.

I: And could you speak to what specific substance use or substances you see your patients using?

P: It depends on the year

I: hmmh

P: You know the ah everybody’s aware of the opiate crisis. Opiates have certainly been an issue for every branch of medicine people with chronic pain opiate use and with concurrent use other substances we see an incredible amount of people with alcohol dependence in our hospital system. It is rare that I will encounter somebody with chronic alcohol dependence with either medical complications and or psychiatric complications like deliria and confusion um etcetera. We see an incredible number of people in intensive care unit with methamphetamine and other uhh drug use and of course we see a surge here and there of of the so-called designer drugs but those are not the more common presentations. So alcohol, alcohol, a lot of alcohol um certainly a lot of opiates, meth, cocaine, ah crack cocaine are probably the biggest problems that we struggle with.

I: And are there any resources or services that you wish you could refer your patients to but just aren’t available to you?

P: So one of my pet peeves since I work with people with medical problems and psychiatric illness and since I see such a high rate of chemical dependency is that people with medical problems that require close supervision or are not physically fully functional don’t have access to chemical health treatment. So our chemical health structure seems to primarily focus on relatively young and healthy ambulatory people so (cough) if you are unable to walk, if you need to have dialysis, if you need to get insulin, if you ah have some ahh heart disease which impairs your capacity to go up and down stairs or um be ambulating around, um the access to chemical dependency becomes pretty narrow. I tend to think of that as prejudicial ah because the people who in many ways need the most help if you happen to be on dialysis and abusing cocaine, I guarantee that you’ll be dead in a short time. People defy that but the risks are very high and yet those are the people you can’t get into treatment cause nobody wants to bring them into their unit. They have to be gone three days a week to dialyze, somebody needs to escort them, it’s expensive and it’s disruptive to the type of programming that we have set up in our chemical health programs (cough) so I think to me that’s one of the umm hardest access issues. Umm we’re getting a lot more umm what we call the the longer term stabilization programs there’s a number of abbreviations for them ?(ERDS)? and all kinds of other names, so we’re starting to see a few more of those come up but that’s been a big issue for us is people can’t sit in a locked acute facility for four months and we don’t have access to the state hospital system like we used to. [County Psychiatric Hospital] now carries a relatively small number of beds. They are overtaken by the people being transferred from the jail, so we used to transfer forty five people, fifty people per year to go to [County Psychiatric Hospital] for longer term. Last year, we transferred three or four, so that means that we have people that we need to transition to longer term care and there weren’t enough facilities for longer term care. We establish our own crisis residential home with it’s called an ?(IRTS)? ah Independent Rehabilitation Treatment Services, I don’t remember what they stand for, so we’re seeing a few more of those coming up. We don’t have enough group homes and longer term living facilities for our patients

I: So thinking broadly umm are there any other changes to healthcare delivery that you would suggest to better meet the needs of people who do have criminal justice involvement?

P: I think, I think it would be from from the health standpoint, I think it would be umm important and there’s been efforts to do this that cost money and have been abandoned to create transitions out of the penal system, if I can use that word, ah for people who essentially have the opportunity to establish regular care, continue to stabilize their health and engage in in useful rehabilitative ah facilities, that means access to employers who are willing to take people with felony charges. The kind of programs we see in a scattered way I think we need to grow those. I think access to healthcare in the umm, in the legal system is very inconsistent throughout the country. I think in some ways in our county we do a pretty good job because of the relationship between [County] ah and [hospital] [health system]. So we have physicians going to the jail. We have psychiatrists going to the jail. Ah we have case managers there. I think if you spoke with people in that system, they would say that that’s not their role, that’s not their job, this is not a hospital, and that they don’t get enough of it. So they would prefer that those people be taken care of in a different setting, but I think when I look at the system in general, I think people who are in our jail system certainly have a lot more access to healthcare than ah a lot of other counties or states and yet I don’t think it’s enough. I think this hospital we’ve had discussions about creating a forensic unit so that people who are high risk of violence which is a threat to all of our providers, nurses, mental health workers, those people need very specialized care units with adequate security and the access to those is is umm suboptimal if not abysmal. Um so this a big challenge for our whole system is people who need psychiatric care but present a significant danger to others placed in short acute units where staff are getting assaulted, disabled um, and we don’t have enough facilities enough space to be able to provide adequate care for these people. So one of our proposals in the past has been to create a forensic unit for the people with um outstanding recurrent legal, criminal complications or in need of acute psychiatric care. There are some umm systems that have psychiatric units, umm, and that’s crucial because there’s a lot of people in those long term prison placements who will need ongoing psychiatric care back and forth so to be transporting them and dumping them in a different system just dropping them off is not good for them and certainly not good for ?(prison systems)? so that’s a big challenge for us. Aggressive patients and we’ve seen a fairly it seems like an increase proportion of patients with severe aggression difficulties who, unfortunately, have criminal records and criminal histories who have a history of being violent and we don’t have the right facilities to care for them. So I think in general when people like that show up in emergency rooms, people shy away from them and don’t want ‘em because they don’t, they’re not going to be able to find the place to place them when their done. They’re not going to find the group home that’s going to take them and more importantly it puts other patients and staff at risk when you admit ‘em. There is umm a hospital in our state called called [State Psychiatric Hospital] [State Psychiatric Hospital] is designated to do exactly that; to house people who have been found guilty, ah not guilty by reason of mental illness, or have a chronic illness who committed a serious crime or high potential for violence. It’s a highly structured, very controlled hospital which has had a lot of problems because of issues of umm how the patients they’ve managed and they’ve gone through a number of iterations to where they’re at right now. So I assume and hope that that means they’re doing a much better job without all the concerns about patient safety, but we don’t have a lot of access to that because the number of beds that they have is relatively small. So if somebody comes in the hospital and they’re horribly violent; they’ve beaten up a few nurses; we can’t control them and manage them safely. I used to be able to transfer those people to [psychiatric hospital] for acute management in a more secure environment. That’s rare. That’s very rare. So small group of patients but a big problem in terms of the hospital in general.

I: So thank you again for your time today

P: You’re welcome

I: Umm before we wrap up is there anything that I didn’t covered that you’d like to add

P: Not that I can imagine

I: (Laughs)

P: Without getting myself in trouble

I: (Laughs) Umm so we’ll also be like I said interviewing additional physicians here and at a few external sites. Umm once we have reports, papers that come out as part of this project would you be interested in getting a copy of those.

P: Absolutely. Absolutely.

I: Okay

P: So I will tell you that, not related but truly related to the penal system the the legal system is the whole issue of immigration and that it’s hard to talk about health in a [hospital] without addressing the ah the challenges of serving the immigrant population. Um we are lucky that we are we are able to do that without a number of the consequences that people have worried about. In the past there’s been a lot of discussion at higher levels in government about the duty of the medical system to um report people who are illegal umm or not have access to services to them and it does not matter if you put those things into place when there’s a community that’s terrified to come forth because they’re afraid they’re going to get deported. Ah it creates a lot of an extra barrier to care. I have a patient who is undocumented from Mexico who will not walk out outside of his nursing home right now because he’s terrified that ICE will pick him up. And he listens to the news. We have people who are worried that if somebody gets put in a transportation hold in a community hospital, that’s a way that we have to transport people against their will for care, that the police will get involved and that they may discover that the person is umm undocumented so we’ve worked with the authorities to try to make sure that that doesn’t transpire. But it doesn’t matter what you do when the perception is that we’re on the lookout for them and that they’re in jeopardy and it is very difficult to do that. I have a patient whose family gets their medication for him, chronic schizophrenia, in a private hospital, in a private hospital, in a private um pharmacy, these people really have to work for every penny and they can barely afford his meds. They’re terrified to get the meds here because we’re part of the government and even though we say we’re not, we’re part of a county and somebody will discover him. These are not people who have a mental illness. They’re people who are suspicious and scared ‘cause if their son who has schizophrenia gets deported he has nowhere to go. They don’t have family back there. So the immigration theme umm is not directly a legal issue but it is and it can become that very quickly so that’s the only area we didn’t talk about. Alright?

I: Yeah that’s good, thank you again

P: Thank you. Let me know, please share your information with me…

END OF INTERVIEW [49:45]
