# Supplementary material for: Physicians’ perspectives on continuity of care for patients involved in the criminal justice system: A qualitative study
Source: PLoS One. 2021 Jul 14;16(7):e0254578. doi: 10.1371/journal.pone.0254578 (PMC8279398; doi:10.1371/journal.pone.0254578)
Supplement: S2 File — (ZIP) [file pone.0254578.s002.zip › Clean/Participant_20_Audio1_LJ_deidentified.docx]

P: Two audio recorders, I like it.

I: Just in case one dies. (laughing)

P: Okay. (laughing)

I: Yeah so thanks again. We'll dive right in.

P: Sure.

I: Um, like I said, this interview is designed to get a sense of what you know about the criminal justice system and um any experiences you've had with working with patients who have had some type of involvement with the justice system.

P: Mm-hmm (affirmative)

I: Um it’s a joint partnership ah between [health system] like I've mentioned, Folks at the [University], and we're also working with [County] to explore this relationship between the criminal justice system and health and the overlapping disparities between those two systems. And so to start us off, I'd like to get a general overview of what you know about the justice system. Um could you tell me a little bit about what you think of the current state of the justice system in the United States?

P: Sure. Um I will say I don't profess to know much about it. Um, I only know about it in relation to my work.

I: Mm-hmm (affirmative)

P: I will say that my work does slant my view of the criminal justice system in that, I am also a boarded certified addiction medicine physician, so I deal a lot with people with substance use disorder. And so the United States in general still treats substance use disorder as a criminal activity and not a medical activity and so I have my own set of biases, just wanna say that.

I: Mm-hmm (affirmative)

P: Um based on that, because of that. And um it gets to be quite... it does color the way that I view the criminal justice system. That being said, um my understanding is, you know, how it’s supposed to be and how it actually is are two different things sometimes. Um, in general the criminal justice system, you know, you’re innocent until proven guilty and that this all theoretically you’re judged by a panel of your peers. Um but there's a lot of in in practice there's a lot of discretion by the district attorney’s office about what to pursue and what not to pursue. And in my experience they pursue things that are motivated either politically or economically that um doesn't seem to be balanced in the will of the society, to say. That's a long way of saying that I guess, the system is supposed to be blind and fair but I I have not found it that way. Essentially.

I: And so next I'd like to discuss some criminal justice system terminology.

P: Sure.

I: I have a few terms that I'm gonna go through. Um could you explain to me what comes to mind when you hear the term prison?

P: Sure. Prison (laughs) that's an interesting question. Prison to me, I don't know, it means this large imposing concrete structure that is heavily fortified and defended um its ah not very comfortable, over crowded, um far away from anybody typically. Um and where people that are serving longer sentences are typically sent to. For variety of reasons. I- I am aware that there's you know minimum, medium, and maximum security prisons. I think what comes to mind though when you say prison is the maximum security prison, at least in my mind. Um, not yeah they’re not particularly comfortable places, they're not particularly nice places. Not particularly safe places. Um, yeah I guess that's what comes to mind when I think of a prison.

I: Mm-hmm (affirmative). And then the next term is jail.

P: Sure.

I: So similarly what do you think about when you think of the term jail?

P: I think it more of like a- a county holding area. Um so, less imposing more, I don't know. Less imposing, less fortified, I- I don't know I think of a prison with like you know barbed wire and walls and think of the search lights and towers and kind of like the Shashank Redemption honestly, is the thing that comes to my mind. A jail comes to more of a ... I guess, I guess what I'm thinking there was a jail right by my high school actually, not too far and that's what kind of I think about, it was a large building um it had bars on the windows but other than that it looked a lot like an office building. I'm sure once you went, I never went inside, but I'm sure once you went inside it was different. Um in my mind jails actually I think are more crowded, at least in my mind, um a lot of ah mixing of people that may be innocent and may not be innocent and people that have more criminal history and less criminal history. There's less kind of segregation and medium I don't know if this is true or not, this is what I think about but you know the, you know the different types of criminal activity so you could have somebody that is accused of murder housed with somebody that's accused of forgery. Something like that and um can be a little bit ... and there's also shorter time stays so this can be a little bit more volatility there. This is what I think of when I think of jail.

I: Mm-hmm (affirmative). And then what do you think about when you hear the term probation?

P: Ah that's a good question. Probation um I think (laughing) I don't, I- I- I've not sat and thought about it. I guess probation I think of somebody that was in jail to be honest, ah prison sorry, that was released that is being, still being under sup-, supervised by a probation officer so somebody that they have to report to and they have to do a certain set of things that keeps them out of having to go back to prison. Um typically under the eye of a probation officer.

I: And then what do you think about when you hear the term parole?

P: Somebody who is being placed on probation. To be honest.

I: Mm-hmm (affirmative)

P: I don't, think of them being different.

I: So you, just to clarify, there, are there any ways that you distinguish between the two or?

P: As I said, I don't profess to know a lot about this but no I- I- I don't, really. No parole sorry say that parole and?

I: Probation.

P: Probation. No I view them the same. Yeah, I'm guessing that's not true but it’s alright (laughs) I, I've honestly never thought about it.

I: So next I'd like to dive into your background and your education and training. Um during medical school did you ever have the opportunity or any training, whether it was formal or informal, on working with justice involved populations?

P: No. That's easy, no I did not have any training on that.

I: Yeah. Do you think that there would have been training during that time that would have been helpful to you?

P: Yes, I do. Ah, it would have been kind of nice to know the unique challenges that people face either going to or coming from an incarceration state.

I: Mm-hmm (affirmative)

P: Prison or jail. Um I mean the, at the bare minimum. Ah and the unique mental health challenges that are associated with that, and socioeconomic challenges ah but no my ah medical school did not provide any training on that. I'm trying to think, I really don't think any like not even mentioned it. It was a while, it, it wasn't that long ago but it was, yeah was like 15-16 years ago so as far as I recall I don't.

I: Mm-hmm (affirmative).

P: I don't recall anything.

I: Yeah. And then what about during your residency? Was there any informal or formal training then?

P: I don't think that there was any formal formal training, like I don't think we had a formal didactics on it, we certainly had informal training and a lot of it because ah I did my residency at [community clinic] identify me or not, but I- I did it here and so we dea- dealt with a lot of patients in and out of the system and you had to, to kind of know a little bit about it, so I had a lot of informal training on it. I don't think we ever had a formal lecture though I could be wrong we could have had it and I just don't remember. Um but no the vast majority is informal and I was also involved in the underserved track here so you know I would go to the um, various shelters and um half way houses and um things like that to talk to people and so I had a lot of informal training from mentors here but no nothing formal. Ah and obviously I didn't have any legal terminology training (laughing)

I: (laughing) And as part of your training did you complete a fellowship at all?

P: I did, I did a fellowship in chronic pain and ... addiction medicine, chronic pain management.

I: Okay. And then similarly like during that time was there any type of training around justice involved patients?

P: A little. A little, because and that focused mostly on the rights and ways of people that are chemically dependent. So more around commitment for chemically dependent people.

I: Mm-hmm (affirmative). Could you tell me a little bit more about that?

P: Yeah basically ah, I mean this is gonna sound terrible but when and how to take away the rights of somebody who is chemically dependent. And basically to have the legal system force them to go to treatment. And when people qualify for that and when people don't qualify for that. That's the main training I got around that.

I: Okay.

P: To be blunt.

I: Yeah. And then thinking about, you know, your current place of employment or a past place of employment um was there any job training on working with justice involved populations then?

P: Informally. On the job training. Some I guess some, there was some. Again more around chemically dependent patients. So I did have some formal training on that yes.

I: Okay. And did that differ from your fellowship training at all?

P: Yeah.

I: Was it, was it the same thing?

P: It was more practical. You know just kind of how the system works. You know in fellowship it was very theoretical.

I: Mm-hmm (affirmative).

P: Um but once I started working it was much more like how do you actually do this, when is it appropriate, when is it not appropriate, that type of thing.

I: Mm-hmm (affirmative).

P: Not when is it appropriate but kind of how do you do it. Or how do you stop it if someone else has started it and you feel it’s inappropriate.

I: And this is focusing again on the commitments?

P: Yeah.

I: When to commit someone. Okay. And so now thinking about your day to day visits with your patients, do you ever ask them about whether or not they have been involved with the justice system or their currently involved in some way?

P: You know, I don't do it standardly. Which is interesting, probably should. But no it comes up more once I know someone, we start talking ... I honestly, it comes up mostly when I start developing relationship with a patient, I just do mostly primary care still. And we start talking about employment that's where it comes up. They talk about how it’s hard for them to get employed cause they have a felony and then I talk a little bit about their involvement in the criminal justice system.

It does come, sometimes come up ah for parole or probation or whatever (laughing) is it, I have to write letters to their parole officer and I do see, again cause I see a lot of chemically dependent patients, a lot of people are on the parole for that so I have to submit things to their officer and submit your drug screens so that- that- those patients actually come to me and say “hey look I'm on parole or probation” or whatever, its called. And say “ah I need you to fill out this for my parole officer, I need you to fill out this for this or that or the other thing” so. Um so it comes up in that, it comes up ... where it comes up is establish a relationship talking about their employment, talking about interacting with their parole officer, talking about um and then talking about their financial, housing, food security. Um because sometimes they have trouble accessing certain benefits that I would offer them because of their status.

I: And then once you have that information how does it inform your treatment plan for that patient?

P: Ah it does, you know, especially if they have food or housing insecurity, it does change the way that I will address things. It can change a lot of things because depending, especially on their financial situation, it may change entirely the medications I prescribe. And um some of the recommendations even, you know, if they don't have very good medical coverage then some of the medica- especially for the- the- the condition that comes to mind most is diabetes. Because um it’s so tied into, some of the medications are very inexpensive some are incredibly expensive. And also to lead a good healthy diabetic lifestyle costs more money than not and so I will, in diabetes it probably changes the most. But in all, in all of them even high blood pressure, addiction, everything it does change because, start thinking about the cost of the various treatments that I'm gonna recommend. Weigh more heavily I guess because I know that it’s harder for them to access services but that, that being said it’s not unique to just post incarceration patients. It is more, a- anybody with um financial insecurity. I would, I would do that for, and we do screen for financial insecurity.

That's a big one, the other one honestly is if someone is heading into a criminal justice system comes to me for um some sort of chemical dependency problem. It, that greatly affects how I'll treat them because I know that there's certain treatments they cannot continue while their incarcerated. I don't want to start them on something and then have that taken away. So we have to come up with a different plan.

I: And what are some of those treatments that aren't available to them?

P: For, you talking about the chemically dependent patients?

I: Mm-hmm (affirmative).

P: I mean the big one is a MAT basically Suboxone. Suboxone therapy so someone’s opioid dependent. Um and to start them on Suboxone and then have them go to jail or prison shortly afterwards I- I know it will be stopped, most of the time. [doctor name] will, working on that but it will be stopped most of the time. And ah leads to all sorts of problems, I- I don't want to put them into withdrawal, I mean if they've already gone through withdrawal I don't want to put them on Suboxone and have them go through withdrawal again in jail, it seems mean. So we've kind of had to try to figure out a- a way to manage it. Um also there's other medication Vivitrol is another one where you get monthly shots and that's not available in prison either so have to think of a way to manage that. Um yeah those are the two big ones.

And then of course ah, the big one to is if they, especially if they opioid dependence and they get released. That time when they're released from prison is a, is a, or jail is a, is a really risky time for overdose. Relapse and overdose and so we talk a lot about how to manage that and hopefully come back and see us before they get access to elicit opiates. But that almost never, I mean you know as soon as they leave ... in prison obviously they have access to elicit opiates and as soon as they leave its, all around them so. It’s hard but those are the, that's the big thing.

I: And so thinking again about your interactions with patients around this topic and how you’re talking about it

P: Mm-hmm (affirmative).

I: Um are you specifically or are there instances where you’re asking directly whether or not they've been involved or are you more waiting for the patient to volunteer that information themselves?

P: I would say, I- I- so I, you know thinking about it I don't have a standard way of doing it. I would say that my style is to be more direct. Especially when patients start hedging about their employment. A- again that's typically where I see it. Um and I’ll ask you know do you have barriers to your employment, have you been involved in the criminal justice system, do you have ... and I- I the other one that I see a lot is actually literacy, so I kind of lump it in with you know, can are you able to read and write. You know, can, are you involved in the criminal justice system, kind of give them a couple of things like that.

I: Mm-hmm (affirmative).

P: Um, but I wouldn't say I always do it. I- I don't yeah. I'm sure there's reasons why I don't on some patients and do on others. Be interesting to look at personally but I um, I am more direct in general. A lot of things.

I: And could you tell me a bit more about any benefits that your seeing to having this information from your patients?

P: Sure. I think the biggest benefit is to help tailor a treatment program that will be effective for them. I- I can prescribe whatever I want for patients as I'm a- I'm a doctor but if I prescribe something that's too expensive or they can't take it’s not gonna work. So I, kind of need to work with patients and work with them about what they can afford in particular. What they can continue to take while, if they're heading into the system. If they're coming out of the system offer them resources, job training, things like that. Um and just support. I mean I think a lot of patients that I've seen that come out of the criminal justice system especially ones carrying felonies, um there's a lot of hopelessness that I've seen. And to offer them you know, I see a lot of patients that have come through this and there is hope and you know you can build a life after this, can be helpful. Um, yeah, I think those are the biggest benefits.

I: Mm-hmm (affirmative). And then on the flip side, are there any challenges that you’re seeing to broaching this topic with your patients?

P: Some patients can be a, especially if you don't, if you do it at the, it’s a sensitive topic. Like chemical dependency right, [inaudible] hey you use heroin? I mean they, I can't, they're gonna say “no”, right. So the- the challenge is if I don't have a good enough relationship and I say something like have you been involved in the criminal justice system. The- the- the issue is I get the answer no. And then after several visits I'll get the answer yes. Um I'd say that's the biggest challenge is me screwing up the, the climate to ask. I think there is stigma around it and patients feel that they'll be judged by me if they answer in the affirmative and, and I think they occasionally are scared to do so. And it’s because I didn't appropriately set up the environment, to ask them correctly. Same with drinking and drug use, for me at least in particular. It’s something that carries stigma that um I have to set up the environment correctly otherwise you won't get a true answer. Things like that.

I: Yeah. And could you now tell me a little bit more about your overall patient population and who you’re seeing on a day to day basis?

P: Sure. Um it is, it is odd my patient population at [community clinic] in that, because I do a fair amount of addiction medicine and because there are not a ton of addiction medicine providers that my general patient population is somewhat different than the general patient population at [community clinic]. I literally just presented something like this at the American Society of Addiction Medicine. But at this clinic I don't, I don't, mean I don't have the numbers in front of me but I would guess roughly 70% are African American, in our addiction program 70% are Caucasian actually and only about 25% are African American. Um and I would say certainly looking through my panel, my panel does skew to be more Caucasian and frankly more ah higher socioeconomic status. Because people travel from long distances, and as I tell the residents, the people that have, that access treatment the most are the ones that can access treatment the most. And since it’s a limited resource it’s the people that typically are more resource that will access the treatment.

Um and so my patient panel does skew that way, that being said I do also full spectrum family medicine so I do have a, a cohort of patients that are not addicted patients that I see. And those do much closer line up with [community clinic] in general. But I would say because of that I would say, half of my patient panel is probably Caucasian. Roughly the other half is African American, of those patients in general I would say 80%-90% are the lower socioeconomic class. Maybe less than that cause the addiction patients. 75% are in the lower. And I would say also, again because of my background in addiction medicine more probably, I would guess more like 30% have been involved in the criminal justice system in some way. Um.

I: And then-

P: Yeah just a, it’s just a, broad I mean I- I'd have to look at the numbers, this is all guess work. Really.

I: And then how would you describe their disability status?

P: Can you clarify? I'm sorry.

I: How would you um-

P: Like in general?-

I: Describe their-

P: My patients?-

I: In general your patients’ disability status?

P: Its interesting, I have a fair number of patients on disability for their addiction. Not permanent disability, but temporary disability. Um to allow them to go to treatment, to allow them to access disability services while they’re in treatment, and things like that. I- I don't know if that's what you’re asking or are you asking more permanent disability? But I have a fair number on temporary disability. Permanent disability I have some, cause I also deal with some chronic pain patients also, though not a ton. Um and so I have more, I do have a significant number of chronic pain patients that are on some form of permanent disability. But I would say the v- ma- vast majority of my patients are not on disability. Like permanent disability.

I: Okay. And then again [crosstalk] your overall patient panel um are there any, are you noticing any challenges that racial and ethnic minority patients are facing in particular?

P: A- again I hesitate to make statements on this without data. And I haven't done a lot of digging into this. I will say that it certainly appears that patients with substance abuse disorder in our minorities have more involvement in the criminal justice system. I have patients that can vary to be quite wealthy that have had charges similar to patients that are of economically, its more economically, um but, at least in my practice that also follows racial ones. Not always but mostly. And so patients that are wealthier that have had similar, very similar charges to patients that are less wealthy that basically get either minimal penalty or no penalty. And patients that are poor have much more severe penalties.

Ah DWI's a good example I have um a woman who is quite wealthy and she's had three W- DWI's and has never been into, into prison or had any kind of ... she has two kids and never had her kids looked at with CPS or anything like that, despite the fact that I did report her to CPS. Um and she's sober now, and that's great and she, you know everything is great now. She's been sober for a while but I have a lot of patients who are not as wealthy as she is that have had DWI's that have lost custody of their kids and have spent time either in a halfway house, a work house, have been fired from their job because they can't go in because of court dates and things like that. So, I- I would say that's the biggest area of dispar-, disparity that I've seen. So other one is of course possession charges, that, again I hesitate to, I mean making a broad generalization I haven't looked at the data or analyzed it. But in my view typically poor patients and African American patients in particular are more likely to have much harsher penalties for possession charges than those that are not African American and poor.

I only have, like I can think of the number of African American patients I have that are on the upper socioeconomic status but they’re not addiction patients. So it’s hard for me to, I- I, there is a lot of that but the ... because of where the clinic is located and the way that my um practice brings in non-African American patients I do typically lump African American and poor together and Caucasian and less poor. That is certainly not true, I have certain, a lot of Caucasian patients that fall below socioeconomic status. So it does kind of, the racial and economic things get tied together as I'm sure they do everywhere. Um but yeah, that's what I'd say, I'd say basically African American, low socioeconomic status patients are clearly in my practice, I wouldn't say clearly but in my experience um suffer harsher penalties and have more barriers placed than those that do not. That are not.

I: And so now, thinking specifically about patients-

P: Oh sorry. The other thing is um-

I: Go ahead.

P: I was just thinking about this, CPS I mentioned them briefly but I- I have had some experiences where you know, I am a mandated reporter um and the law around substance use and pregnancy is very clear. I don't agree with it but I do follow it. And I have reported many women for a relapse when they're pregnant as is required by the law, again not alcohol, marijuana but other things. And I have to say that I have never had a Caucasian woman be, had an involvement with CPS but I have had several non-Caucasian women either Native or African American women that have been involved with CPS. It’s again the and the number is small but it’s not insignificant.

P: Sorry.

I: No problem. Yeah so I just wanted to shift a little bit and think, thinking specifically about your patients that have some type of justice system involve, justice system involvement and digging more in to that, what that experience has been like for you. And I know you mentioned before that you do communicate with some parole or probation officers and I was wondering if you could elaborate a little bit more on what information is being shared.

P: Sure. It is entirely what the patient wants shared.

I: Okay.

P: Um again its pred- ah I- I- I would say again, without looking at data but I would say, it’s almost always related around their treatment for substance use disorder. And that they want me to state that their under treatment for substance use disorder and to release their urine drug screens. Occasionally it’s because they, they couldn't meet with something or somebody because they were ill. I've written that letter, it’s not always related to substance use disorder. I have had um parole officers call the clinic once, that happened once, asking to talk about a patient. Um but I didn't have a release so I- I- I did not talk to them. I can't remember if it was a man or a woman, the parole officer so I didn't talk to them, but that's it. It’s mostly at the patients request to, and its mostly patients that are doing well that say "hey please send my urine's to the P.O. and tell them you know, my P. O. that I'm getting treated with this or this, that, or the other thing.”

Occasionally um I did have a woman who has pretty severe PTSD who was driving erratically because she had, was having a bad PSD, P- PTSD flare and uh got charged with drunk driving even though she wasn't drunk. And so I wrote a letter saying that she had severe PTSD and that this is likely representing a PTSD flare that's why she was, she was, she was driving away, she thought the cop was her ex-boyfriend and she was trying to evade him. But turned out it was the police.

I: Mm-hmm (affirmative).

P: Um things like that. It’s alw- its always at the patients request. I- I- I- the only time that any sort ... it depends if you count CPS as a criminal justice in which it really isn't. The only time I interact with that entire system is when I'm a mandated reporter by CPS. That's it.

I: And so now, I and I'm curious about this, especially because you’re an addiction medicine provider are you getting any patients that are specifically referred to you from perhaps the criminal justice system?

P: No. Not that I'm aware of. Least not that they tell me.

I: Okay.

P: No because most of the criminal justice system in my experience doesn't treat addiction as a medical problem. So they don't refer to physicians, they typically refer to other correctional groups and programs. Typically.

I: Mm-hmm (affirmative). And do you have a sense of in general, how patients are finding you and coming to you for care?

P: I don't know. That's a good question. Um I certainly there's w- w- w- word of mouth I mean that's the one that I hear the most is that patients will tell me, well you tr- helped my sister, you helped my cousin, you helped my friend, you helped a friend of a friend. Um it’s probably the mo- the- that. Um I do you know I give talks to a lot of primary care providers, some of them refer patients to me. So that there is some professional referral services. I- I- I think just that, those two things mainly.

I: And then how do you think that having some type of justice system involvement may have impacted your patient’s ability to access care?

P: That's a good question. I sort of think it makes it harder um so, though not always. I mean the interesting thing is some of my patients who have done the best are when the um, I'm thinking of one patient in particular, the- involved in the criminal justice system and basically placed on parole or probation I don't know (laughing) you’re gonna, after this you’re gonna have to tell me the difference.

I: (laughing)

P: Um and um were mandated to have random sc- drug screens through the parole system or probation whatever. Ah and um it actually really helped them stay sober because they have these kind of random drug screens. And so people that were relapsing constantly got involved. Actually got stabilized because they didn't want to go back to prison. And so were kind of, would come to me more with a clear head and they could start you know hearing about well I'm glad that you’re not using drugs but how else can we help your, you know your, life get better. I mean addiction medicine isn't just about stopping using drugs, it’s about the whole disease of addiction and the whole, all the other things that come along with it. Which is not just the- it’s the drug use is the least of their problems, most of the time.

And so it has helped push some patients along, not push, but gently motivate them to um a path of wellness. It does happen, that's the most common positive interaction I've had. The most common negative interaction I have is the patients that are going back to prison for one reason or another that um gets their Suboxone stopped. Go through terrible withdrawal, relapse, I don't see them for months or years after they come out of prison because they're back out using, or even die. I'm sure I've had patients die, I just don't know because I don't see them anymore. That's the worst interaction, to criminal justice system.

I: And then aside from possible justice system involvement, what else are you seeing these patients dealing with socially?

P: Um problems with employment is probably the biggest one, I- I have had patients, a couple, not many, ask for disability and again I'm not gonna lie on a Federal form, they're perfectly capable of working they don't really have, try ask, they don't have anything to be disabled for and I say well why do you want disability and they say well I can't get employed any other way, it’s the only way I can get money. It’s almost always because they have um a felony charge and it’s hard for them to find work. Um it’s not many I mean may- it’s like one to two so that's a huge problem. But there are a lot of patients that do struggle with employment because of the felony charge and so um socioeconomically you know again food security, medication security, housing security, it hard to ... I mean I'm not even aware of all the stuff with section 8 and felony stuff but I do know that my patients struggle to find affordable housing with a felony charge. Um yeah, it’s really hard for them, they struggle on those three aspects and it just, it makes everything more difficult. I mean how can you focus on your diabetes treatment if you don't know where your gonna s- s- spend your next night or where your next meal is going to come from and you can't. So yeah that's the, the biggest. Does that answer your question? I feel like I didn't answer your question.

I: Yeah. Yeah that, that answers the question. And then what are you seeing justice involved patients dealing with medically?

P: Again for me mostly it’s- it’s- it’s some sort of chemical dependency.

I: Mm-hmm (affirmative).

P: Mental health problems are probably second depression, anxiety, PTSD, insomnia. And the third honest is probably diabetes. A lot of diabetes, asthma, hypertension, kind of the normal stuff that family practice doc- docs see mostly.

I: And then what's perhaps the most common chemical dependency um conditions that your treating?

P: Alcohol. Alcohol’s the most common still, I know opiates get a lot of the press but alcohol is the most common. And- and theoretically nicotine, theoretically falls under that. Um not excluding alcohol and nicotine probably tied for opioids and meth. Then cocaine, then every- everything else. Few patients meet criteria, true criteria for marijuana use disorder, a lot of patients abuse marijuana but they don't technically meet criteria for marijuana use disorder which a little, it’s harder to get the formal diagnosis of that.

I: And then are there any resources or services that your patients need but you’re finding isn't available to them?

P: A- a- mostly housing.

I: Mm-hmm (affirmative).

P: Occasionally um SNAP benefits, food benefits, though that's been easier recently. The last couple years. And then employment, employment and housing I'd say. Voting too, I mean occasionally I'll discuss that and a lot of patients under the impression that once they have a felony charge they can never vote again but that's not true and we talk about that a little bit. Um yeah I think that's it.

I: And now thinking broadly are there any changes to healthcare delivery that you would suggest to better meet the needs of individuals who have been-

P: Man I'd love if they'd just let us continue MAT in prison, that would be the biggest, I mean that's my little, not my little, that's one of my big pushes these days is to allow continuation of MAT treatment in prison. Um a- a- you know it would be really great if I could, if people with felonies could get more support for employment, cause it’s a big issue. Almost everybody that I meet that struggles with employment has a felony charge, desperately wants to work. They just want to go back to being a productive member of society, and the barriers placed in front of them is challenging.

You know, somebody who has additional training in some mental health things I mean, you are asking someone that's leaving prison to do something different than they did, meaning it’s hard for their brain to do that. Their brain is, easier for them to do what they've done in the past, like it is for everybody. Nothing special about them. It’s easier for me to do what I've always done than to do something new which is the way the brain works. What they've always done or what they have been doing and then being involved in the criminal justice system is easier for them but you’re asking them to do something different, which is great, most patients do want to do that but then you put a bunch of barriers in the way of them doing something different it’s just gonna force them to go back to doing what they've done in the past anyway.

As a healthcare provider that's what I see. And its um its really frustrating when you have patients that ah that want to work legitimately and I use term because most of my patients that deal with this have had drug dealing charges. Distribution charges. Um and they want to find a legitimate, not- basically a non-drug distribution. Don't deal drugs, right they don't want to deal drugs, they want to find a- a like a legitimate job, and they can't cause of their felony charges. And they're not gonna starve, they're gonna find, I mean they gonna go back to dealing drugs cause they can make money doing that. And then they get picked up again and go back to jail. It’s just, makes me upset. Cause they want I- I- I mean they don't want to do this but they kind of, we are forcing them to do this. That's my two cents.

I: Yeah. So thanks again for your time today, before I officially wrap up is there anything that I didn't ask you about um that you'd like to add?

P: Yeah, I guess the one thing I would like to add is um, there's states, not Minnesota, but there are states that have more robust chemical dependency treatment in the correctional system. And I would argue that that is something that could pay a tremendous [inaudible] I mean, you have a captive, literally, patient population. They can't go anywhere, they're in prison. It’s much harder for them to find drugs so they are typ- I wouldn't say impossible I- I'm not naïve, it’s harder, its more expensive to use drugs in prison. Um and so you have them relatively clear headed in an opportunity for them to find addiction treatment and yet I feel like we squandered that time. It would be in a- relatively inexpensive and I think other states have proven, proven tremendously, I wouldn't say profitable but you'd save a lot of money on the other side by getting people treatment. Yes of course people are going to relapse but a percentage of them won't. And that percentage that doesn't will save the state a tremendous amount of money. There.

I: Thank you.

P: Ah huh.
