# Supplementary material for: Physicians’ perspectives on continuity of care for patients involved in the criminal justice system: A qualitative study
Source: PLoS One. 2021 Jul 14;16(7):e0254578. doi: 10.1371/journal.pone.0254578 (PMC8279398; doi:10.1371/journal.pone.0254578)
Supplement: S2 File — (ZIP) [file pone.0254578.s002.zip › Clean/Participant_21_Audio1_LJ_deidentified.docx]

I: Okay, so thanks again, um, for taking the time to meet with me today. The questions of this interview are designed to get a sense of what you know about the criminal justice system um, and working with patients who have some type of justice system involvement.

Um, and I want to begin by getting a general overview of what you know about the justice system. So to start us off um, could you tell me a little bit about what you think of the current state of the criminal justice system in the U.S.?

P: Um, the current state of the criminal justice system. Um, currently in the U.S., we incarcerate a high percentage of our population compared to other similar countries. Um, and uh, that is disproportionately uh, affecting um, different socioeconomic groups and uh, racial subsets and and ...

I think that there's a a long way to go, as far as ah, reforming the way our current system is. Uh, mostly uh, related to uh, sentencing guidelines and uh, around nonviolent uh, type offenses. Uh, that has been slow to occur in my adulthood.

I: Thank you. And then next I'd like to discuss some criminal justice system terminology. Um, could you explain to me what comes to mind when I share the following terms? There are several. Um, the first is "prison."

P: Prison. Um, long-term incarceration comes to mind.

I: And yeah, then what about "jail"?

P: Jail. Um, shorter term um, sometimes uh, used for uh, like I said, shorter term but also uh, oftentimes more local uh, type situations for incarceration.

I: And then what comes to mind when you hear the term "probation"?

P: Probation. Uh, probation is a h-, big wide term. Uh, I I always think of it as uh, an ongoing part of an individual's sentencing that is often in lieu of, or after the actual incarceration. Uh, where an individual has to continue to meet uh, certain uh, aspects of uh, their responsibilities or expectations to stay out of an incarcerated situation.

I: And then what comes to mind when you hear the term "parole"?

P: Parole. When I think of parole, I I think of uh, the ability to uh, remove oneself from either the incarcerated state or uh, probationary state.

I: Mm-hmm (affirmative). And could you tell me a little bit more about how you distinguish probation from parole?

P: Uh, I ... that, uh, my own distinction is not uh really grounded in a lot of fact, I guess. Uh, for me, it's uh, I think of probation as ongoing. If you mess up, you go back. Um, to, you take a step back or two steps back. Um, within the system. Whereas parole is the ability to uh, kind of remove oneself from that track that they're on.

I: And now I'd like to talk a little bit about your education and training. Uh, during medical school, did you ever receive any types of training, whether it was formal or informal, on working with justice-involved patients?

P: I received no training with justice involved. Uh, individuals.

I: And, do you think that there would have been a place for that in your medical school curriculum? Or would it have been helpful to you?

P: I believe in my residency training; so, post-medical school. Uh, that would have been the place where it would have been uh, effective, uh, for how to manage some of those situations.

I think that within medical school being introduced to uh, the topic, and uh, and maybe introduced to why this is an issue that physicians should be worried about, uh, uh, based on how it affects our uh, patients. Uh, that would have been an appropriate place to have it, would be in medical school for that aspect of it.

I: And then during your residency, um, in your experience, was there any formal or informal training them?

P: Uh, no formal training. Uh, but in my residency, uh, we managed a locked unit within a university health center. Uh, where uh, these, those patients um, were all within the uh, justice system. And so that was very informal training as to, "Okay, wh-, how do we manage these patients? What is going to be their next step when they leave the hospital? Who's going to be able to follow up on the care plans that we developed? Uh, and also why are, why are these individuals getting placed in our care within that healthcare system? Within a hospital? Uh, and having to be locked up?" So that, that was sort of my own experience.

I: And, as part of your training, did you complete a fellowship at all?

P: No fellowship.

I: And now, thinking about either your current place of employment or somewhere where you've worked previously, was there any training on the job on working with justice-involved patients?

P: No training. Um, on the job. Um, my only uh, brief bits of education have come from continuing medical education talks that were put out by other providers who provided this care.

I: And then where, could you tell me little bit more about where those are situated? Are you, are they like conference talks?

P: Yeah.

I: Or-

P: Yeah, mo, mostly just uh, conference talks. Uh, and uh, there was like [University Outreach Conference], where uh, I saw a talk on caring for uh, population who was recently incarcerated. Uh, but very uh, very uh, more much informational and like "Here's the big picture," and not so much "Here's some actual strategies to put into your own practice." Very generalized.

I: And then, during your day-to-day visits with your patients, are you asking them whether they've either in the past have been involved with the justice system, or have some type of current involvement?

P: Uh, not routinely. It's not part of my regular screening uh, discussions with patients.

I: And then when it does come up, could you give me an example of how it comes up, and maybe how you talk about it with the patient?

P: Uh, how it usually comes up is uh, when I'm discussing the social history of where they're living. Uh, what's going on with their job. What's going on with their family.

It it will come up sort of organically, like, "Oh, I have a court hearing," or, "I have to meet my parole officer." Uh, and when those things come up, uh, I just ask for more clarification. Kind of.

Uh, how long you've been in this situation. What's your next steps? How is this impeding those other aspects in your social life? Like, is this getting in the way of you getting a job? How is this going to affect your healthcare, your ability to continue to come to our clinic for healthcare in the future?

I: And then, how does that information influence how you approach a treatment plan for that type of patient?

P: Uh, I think that if we know that there's a possibility that the patient will not be in their current living situation in those type of aspects, uh, in the very near future, we often try to make sure simple things like, you have a year's worth of all your refills. You have a game plan of when you're following up next. We'll be a little bit more mindful of those type of aspects than I would if I didn't think that those type of uh, stressors were coming up to them.

Uh, so that's one aspect. The other thing that often comes up is uh, because we're a family medicine clinic, we'll often push them to talk to their family members to come into our clinic too, to talk to them about what's going on.

Do they need any other assistance that they don't have? Uh, and then oftentimes I will offer behavioral health services uh, because of you know, this is an adjustment type phase. And I'll offer our behavioral health services that we have here onsite. Uh, more acutely than I maybe would otherwise.

Uh, so we have the ability to do uh, hot handoff where the p-, where the behavior health scientist comes in the room during my visit. And we make it, we have a shared visit. Uh, and so I'll be more likely to do that rather than just refer a patient to behavioral health. If we think that there's a time-sensitive issue coming up.

I: And are there any challenges that you see to broaching this topic with your patients?

P: Uh, I know that I've had patients in the past who uh, feel a little bit either ashamed or shy to bring it up, because they're worried about how we're going to treat them. Uh, especially our patients who are on uh, controlled substances, they feel like you know, we may just pull those away. Uh, from them in that situation. Uh, and so I think that's been a barrier that I've heard verbalized from patients in the past, and why they didn't bring those things up.

I: And then on the flip side, are there any benefits that you see to having these conversations with your patients?

P: Uh, I think anytime we can get mental health services involved with people who could benefit from that, that's a positive thing. I think, the other thing is just having that coordination of care, and having that knowledge of what's happening. Uh, and talking, being able to talk to our patients about, "Here's all the services we have in our clinic."

Reaching out to us right away, uh, early on in the process, uh, before they get kind of put back into the sys-, the uh, justice system. Or even right when they are out of an incarcerated situation. Coming in right away, and stressing those type of things. Uh, could have benefit; just like if you have a patient who's hospitalized, and they leave the hospital setting. You want to see them soon once they get out of the inpatient setting to kind of help with that transition.

I: And then, too, could you give me a sense of, a bit more about your overall patient population and who you're seeing on a day-to-day basis?

P: Uh, would you like to know about our clinic overall population? Or my own individual?

I: Your own, yeah.

P: My own individual population. Uh, so I currently only see patients one or two half days a week, within the clinic. Uh, 90% of my patients have chronic pain diagnoses. Uh, and 60% of my patients have multiple comorbidities; uh, chronic diseases that uh, need to be managed more frequently than every three to six months. So, uh, lots of management.

Uh, my patients are coming to see me oftentimes for uh, part of their pain treatment is uh, non-medication treatment. So I'm an osteopath, so it's performing hands-on treatments for their pain. Uh, as an adjunct to their usual medical treatments. So that's my practice.

Uh, my patients uh, generally trend to be younger. Uh, a majority of my patients are between the age of 30 and 60. Uh, and about 75% of my patients are female. Uh, [inaudible]. I'd say that's my patient population.

I: Mm-hmm (affirmative). Then how would you describe the income levels of the patients that you're typically seeing?

P: Uh, majority of my patients, so, over 90% are on state-funded insurance. Uh, and I I cannot speak to their actual income, but uh, knowing that they are on state-funded insurance based on their income base gives you a sense of kind of where they're at.

I: And how would you describe the disability status of your patients?

P: Uh, my patients have a higher disability scores and lower functional uh, status than the general population here in the [cities name] and definitely Minnesota.

I: And then, in your experience, uh, are you noticing any challenges that racial or ethnic minority patients in particular having in terms of barriers to care?

P: Whoo. Uh, so. That, being here in our clinic, uh, a majority of our patients identify themselves as uh, black, African American, uh, and there is an obvious uh, issue with their ability to receive routine medical care and emergency care and hospital care. I think that that goes without uh ... For those of us who work here in this situation, we know that that's just a reality for our patients.

Uh, many of them express that they feel like even though our clinic is here in this neighborhood, they feel like they receive better care than if they went to a different clinic. They still feel that their care is less than if they were a white patient who lived in the suburbs three miles away.

Yes, uh, definite, obvious uh, social constructed racism that is affecting our patients in every aspect of their lives.

I: And then, shifting back to your patients that you're working with who have justice system involvement in particular, I'd like to dig into more of what that experience is like for you as a provider, and how it may differ from patients that don't have some type of involvement in the justice system. Uh, you mentioned earlier uh, a patient mentioned, you know, they have to meet with their parole officer.

P: Mm-hmm (affirmative).

I: So I'm wondering, do you ever communicate with parole or probation, or um, any parts of the justice system at all?

P: The only communication I have had with uh, in that type of situation is when a patient has told me uh, "I need to have evidence of my urine toxes for them." And I'll print out an extra form. But I haven't even, I I leave that up to the patient to pass that information on.

I, as an individual, I've never received a release of information from anyone in the justice system regarding one of my patients to release records or lab results or those type of items.

Uh, and I've never had a patient who's asked me, "Can you communicate with these individuals in particular?" I have had patients ask me to provide letters of support uh, that state what's going on with them medical condition–wise. And how they followed through or not followed through on their care plans. And those type of things. Uh, that the patient has taken individually uh, to either their legal team or to their parole officer. But I have not had direct communication with anyone in the justice system myself.

I: Mm-hmm (affirmative). And, are you noticing uh, any in terms of access to care that having some type of criminal justice involvement is impacting a patient's ability to get healthcare?

P: I haven't seen in the clinic. And the reason why is because our clinic uh, is, if you don't have insurance, you have to put a $200 amount down to be seen. So most patients who don't have insurance aren't being seen in our clinic.

In the hospital setting, where I take care of patients, we see that quite a bit. We see patients who come in weeks or months after uh, they have been uh, had a change in their situation where they're out of their previous living situation. And often they haven't had their chronic pain, or their chronic pain ... their chronic uh, conditions addressed. And we're taking care of them in the hospital setting.

Uh, things that if we had taken care of it two or three months ago, wouldn't have needed a hospital uh, hospitalization. Uh, so that's where we see the most acute need.

I: And are you getting any patients that are referred specifically to your care from the criminal justice system?

P: Uh, we, we have uh, we do provide medication-assisted uh, treatment for opioid use disorder in our clinic. Uh, and so we know that we have patients who have been told to come here. But I have not seen any actual documentation from the justice system that said, "Follow up at this clinic for these type of cares." It's just uh, patients who sort of were told, "You should go be seen somewhere where you can get things like Suboxone," which we provide here.

I: Mm-hmm (affirmative). And then, aside from justice system involvement, what else are you seeing these patients dealing with socially?

P: (laughs). Uh, housing, housing, housing, and food insecurity. Uh, I'd say those are my top four (laughs). Uh, uh, I think that the lack of stable housing is the the number one issue when I talk to my patients about social uh, aspects that they identify that is getting in the way of their healthcare needs. Uh, and then further down the line is uh, food insecurity.

I: And then, again, among your justice-involved patients, uh, what are they dealing with medically?

P: (sighs). Um. High, high amounts of uh, mental health issues, whether that be post-traumatic stress that was unable or was not addressed; uh, depression, adjustment disorder, anxiety, uh, uh, different atypical presentations of uh, psychotic type situations. Uh, that would be the top tiers of mental health.

The number two tier is uh, substance use issues which uh, are often related to that first tier. Patients are self-medicating uh, to address their mental health issues uh, oftentimes.

And then, as we move down in into the the next tier ... especially our patients who are a little bit uh, older, we're seeing a lot of unmet cardiovascular health issues. Uh, and then diabetes. So, those are the the ones that I I see on a regular basis.

I: And then, are there any resources or services that you're seeing that your patients need, but aren't available to them?

P: Uh, it's always hard to tell what's not available. Uh, l, like I said, I think the housing assistance issue is is huge. Uh, just the inability to have uh, appropriate housing for many of our patients is a is a big issue. Uh, so that that'd be the biggest one.

Um, in the hospital, what we see is a lot of patients who haven't settled their insurance yet. And so that just puts a delay on any type of care that they're receiving.

So, I think housing situation, uh, medical, uh, care. Uh, and then after that it's, I think it's uh, again, the food insecurity. Those type of issues.

I: And then, thinking broadly, are there any uh, changes to how we deliver healthcare that you would change or you would suggest should be changed to better meet the needs of folks that have had, or are currently involved with the criminal justice system?

P: Uh, so for patients who are currently incarcerated and are going to be changing their living situation, uh, I would love it if we could do a transitional care visit with their primary care provider. Uh, that involves not only their primary care provider, but a pharmacist, behavioral health scientist, and a care coordinator.

Uh, and that's a, that's something that we even do for our hospitalized patients who aren't within the social, or within the justice system. Uh, we have found that when they're, when people are discharged from the hospital back into the outpatient setting, having a shared visit with all of those individuals uh, often gets the needs met much better than if they just see a primary care provider who then refers them off to those other places.

So, that's one thing, is having a transitional visit. Uh, what would be even better than that would be to have some sort of care coordinator position who, or you know, a social work–type situation who is uh, watching over the medical aspects of that patient uh, throughout those transitions. So, having a go-to contact for the patient, um, no matter what setting they're in, would be very helpful.

Uh, I think from what we see in the inpatient setting, it's having those type, like having housing set up ahead of time, and insurance set up ahead of time. Uh, would be really beneficial in the long term. Uh, for our patients moving forward.

Uh, I think stepping back from our current system and what it looks like, uh, to have uh, more providers who are able to uh, provide a longitudinal type care, where they can provide care in different settings. Uh, would actually be a way to change our system, that could benefit patients even more.

So somebody who is able to see a patient in any of the settings, do house visits, uh, see them in a clinic, follow them in hospital. Uh, that's difficult but that that type of situation could be beneficial. Whether it's a physician or a physician extender. So, that's how I'd change the world (laughs).

I: Well, thanks again for your time today. Before I officially wrap up, is there anything that I didn't ask you about today that you think would be important to add?

P: Uh ... uh, I just, I think stressing uh, from you know, I'm one person who doesn't see that many patients. Uh, but stressing that the transitions seem to be uh, a place where uh, where we could do a lot of good.

And those transitions, if a patient is coming out to see us, and has never met us and doesn't have trust ... I mean, a lot of times they don't have trust in a lot of different uh, structured systems that are going right now in our society.

Uh, so any way we can stay, have some sort of continuity with with these individuals through uh, their situation, a, that trust, th, that continuity, builds uh, I think could assist the patient in moving through our complicated system. Yeah.

I: Thank you again.

P: Yeah.
