# Supplementary material for: Physicians’ perspectives on continuity of care for patients involved in the criminal justice system: A qualitative study
Source: PLoS One. 2021 Jul 14;16(7):e0254578. doi: 10.1371/journal.pone.0254578 (PMC8279398; doi:10.1371/journal.pone.0254578)
Supplement: S2 File — (ZIP) [file pone.0254578.s002.zip › Clean/Participant_22_Audio2_LJ_deidentified.docx]

I: All right. So thanks again for taking the time to meet with us. Um, this project, like I've said, involves us at [health system], the [University], and we're also working with partners at [County], um, to explore this relationship between criminal justice involvement and health, um, and how can we address overlapping disparities in those two systems. And so like, this interview is designed to just get a sense of what you know about the criminal justice system-

P: Mm-hmm (affirmative).

I: Um, your experiences as a provider working with folks that have criminal justice system involvement. Um, and so I just want to begin by getting a general overview of what you know about the justice system. Um, could you tell me a little bit about what you think about the current state of the criminal justice system in the US?

P: I think it's a for-profit industry that has almost, what, a quarter of black men population in it? I think it's an atrocity. (laughs) I think a lot of it is, um, it's trying to use a punitive system to address things that are deeply socially determined. And things like treating addiction as a, as a crime as opposed to something that could be treated. And I think it's the product of a lot of adverse childhood events. When I ... I used to live in [state]-

I: Mm-hmm (affirmative).

P: And when I was there, I was the medical director. One of my jobs was Medical Director of the women's prison, and um, if they would have been able to eli- eliminate child abuse and sexual abuse that entire place would have been empty. Every single one of those women had been abused in some way, in some very horrible ways. And it's just like, you know, they turn to things that help, that ... make bad choices. I think uh, the criminal justice system is disproportionally representing, uh, the failures in our social support.

Um, I look at, I look at ... I remember I had one mom bring her son in cause she wanted him tested for marijuana. He was like 14 years old or something like that. And I was like, "is it okay if I talk to him? Um, just him, and we'll chat and we'll discuss." And she's like, "no problem." And she went to the waiting room, and I sat him down, and I said, "listen. You're probably smoking pot." And he's like, "yeah." I was like, "you have to understand. You're a young black man. And if you get caught smoking pot, it could destroy your life." I was like, "some kid, some guy in Edina smoking pot. It's not going to destroy his life. Probably won't even go to jail."

So when you see that kind of disproportionate impact of a justice system, it doesn't seem very just. And I see it tearing apart neighborhoods and families. I mean, the adverse childhood events, you know, someone in your family going to jail is one of, was one of the more severe ones. This impacts people's long term health. And yeah, I don't think we do a very good job. So, that's just my thoughts.

I: Mm-hmm (affirmative). So next I'd like to discuss some criminal justice system terminology. Could you explain to me what comes to mind when you hear the following terms? And the first is prison.

P: Prison is where you go once you've been convicted of a crime to serve your sentence. Sometimes you serve part of your sentence but not all of your sentence. It's the step after jail. (laughs)

I: (laughs)

P: That's what I think about anyway.

I: Yeah, so then my next term is jail. So what comes to mind when you hear that term?

P: Jail is where you get put before you get tried, and sometimes you don't get tried. Um, jail is a place where a lot of people get stuck because they can't make bail. Uh, a lot of people get stuck because they don't know the right people, and sometimes they have to wait until their trial in order to get out. Um, I've actually been to jail twice. Um, mostly for driving while black. (laughs) Probably one of the few professors at the university that's ever been arrested for non-protesting things. Jail's a frightening place, and um, I, I ... Yeah, that's what I know about jail. You go there before you're tried. Sometimes you stay there a long time because you don't have the ability to get out.

I: Mm-hmm (affirmative). And then what comes to mind when you hear the term probation?

P: Probation. That's when a patient says, "I'm on paper." (laughs) And uh, so, probation, when I think about probation, I think about someone who has either served some of their time or has been put directly into their probation, which is someone watching over you. Various hoops you have to jump through in order to stay out of prison or jail. Um, someone typically I would imagine with a parole officer, who's evaluating them in some way, and that they have to check in with.

Um, so probation is sort of the, uh, it's an out-of-jail, out-of-prison penalty that also can impact your ability to vote. So, that's what I think about it.

I: And then what about the term parole?

P: So, so parole. So parole is, I ... I think of parole as the, the um, the act of getting someone on probation. (laughs) Sort of like the idea of um, if you're, you get paroled, then you can be on probation or parole I imagine. I see where that gets a little iffy. But yeah, so it's you're, you get let out but you still might be monitored in some way. And you have a parole officer. There you go. (laughs)

I: Okay. And so next I'd like to dig a little bit into your background, your education, and training. Um, during medical school did you ever receive any training whether it was formal or informal on working with justice-involved patients?

P: Well it's sort of interesting because I did my psychiatric rotation ... I was at [University] in [city name] and I did my psychiatric rotation at the, um, pri- at the jail. And it was actually at the time, um, when the uh, Unabomber was in the jail, but they didn't let us see him. Which is fine. I'm good with that. But yeah, so I spent a month uh, with the psychiatric unit of the uh, [city jail]. And so that was the formal one, and between my second and third year when I was driving from [city name] to [state], I was arrested in [state] and put in jail. So that was my informal-

I: Mm-hmm (affirmative). And then your rotation during medical school-

P: Mm-hmm (affirmative).

I: Was that experience that all students got or was that-

P: No, no.

I: Okay.

P: It was not. It was um, there were various locations that you could choose and you ranked them, and but, probably less than 10% of the class would have rotated, probably less than 5% of the class would have rotated through there. Yeah.

I: And then during your residency, similarly did you receive any formal or informal training?

P: Uh-uh (negative). Residency ... None, no. Nothing.

I: And as part of your, uh, training, did you complete a fellowship at all?

P: Yeah, I did a um, uh, health policy fellowship-

I: Mm-hmm (affirmative).

P: Through the uh, Department of Health and Human Services.

I: And similarly to that involv- include any type of curriculum around working with justice-involved patients or justice-involved policy?

P: Um, you know, it should have but it did not. I'm like, that would have, that would have been a helpful thing. Yeah, no, it didn't.

I: Mm-hmm (affirmative). And now thinking at, about your current place of employment or past place, has there been any formal or informal trainings around this topic at all?

P: No, and it ... Well, informal, when I uh, so, so the last residency I taught at was the [state] residency in [city name], and um, one of our practice sites as I mentioned was the women's prison. And for the longest time, um, my boss, she's like, "I need you to be Medical Director there." I'm like, "I'm not going to prison voluntarily." (laughs) It's just not going to happen. And then she finally was like, "I really need you to do it." So I, I started doing it and there was just like, there was a very informal training. Like they gave me a book. Like read the book. Do what the book says. And then I was, and then I was in charge of the healthcare at the prison.

It was a really nice place to do clinic though because I mean, most of the time people just needed to talk. And it was really, it was one of my best clinical experiences. I really enjoyed that. Which makes me mad that I didn't go sooner, but I wasn't, I wasn't about to go behind bars voluntarily, so, it changed.

I: Mm-hmm (affirmative).

P: Um, so very informal training even though I was basically in charge of the prison system for (laughs) the women there. So ...

I: Are there things you think you learned along the way as the Medical Director?

P: Oh yeah. Like that, that's ... Like I mentioned that whole thing about, you know, adverse, adverse childhood events don't just impact your health. They impact the choices you make, and like I said, every single one of those women ... If they were not abused, it would have been a hell of a lot less likely that they were there. And just ... most of them, most of them in that particular place were just addicts who were running from their internal demons. And they were, they were placed there by very bad childhoods. I ... literally not one of my patients wasn't abused as a kid. That was, that was sad. They're good people though.

Yeah, it was (laughs). One of the things I also learned is women prison, at least in [state], it's not so much violence but they are, they are mean girls. I mean, (laughs) I remember one woman came in, she's like, I was like, "what's going on? You seem down." She's like, "they're talking about me." I'm like, "they're talking about you?" She's like, "yeah, they're saying mean things." I'm like, "you killed two people. This should not be an issue for you." She laughed a little bit. We moved on. But it was, it was really sort of weird because you know, you grow up watching- well, prison movies? Crime shows-

I: Mm-hmm (affirmative).

P: And um, instead of violence, they were just very manipulative to each other. Hurt people hurt people. So. Yeah. Um, yeah, I don't remember the question. Did I answer it?

I: Oh yeah, I was just asking more about your, what you learned along the way as a Medical Director.

P: Yeah, and then, uh, the big thing. Hurt people hurt people. Um, I, I learned that it is really important to, when you're treating people with medications for chronic illnesses in prison, it's your responsibility to make sure you treat them with the best medication at the lowest price. Because if you get them stable in prison, you want them to be able to continue their medical care outside of prison. And one of the ... He's, he's retired. One of the psychiatrists there, he just could not grasp that. And he always put these people on the most expensive things. I was like, "they will not be able to afford that when they leave." You know, every blood pressure medicine I put them on, every diabetic medication, every thyroid medicine, was one that they could get on the $4 formulary. And that's what I made ... I was in charge of the PAs and NPs there, and I was like, "if you're going to do something that's not on the $4 formulary, you need to have it cleared with me because I want people to leave this place and still be able to, to have healthcare."

And this was before, um, ACA or Medicaid expansion, so I mean it's, it's out of pocket. So um, yeah, I learned that people in prison have the same diseases (laughs) and illnesses that they do everywhere else. I mean, they're folks. And the food wasn't really helpful for controlling chronic medi- uh, chronic illnesses. But, we did our best.

I: Mm-hmm (affirmative).

P: Yeah.

I: And now in your day to day visits with patients that you're seeing, do you ever ask them about whether they have in the past been involved with the justice system or have some type of current involvement with it?

P: Um, there on occasion, like uh, a lot of times you'll ask someone who comes in, and they're like, they're, they're history that they give you sort of gives you an idea that they were in jail or prison-

I: Mm-hmm (affirmative).

P: Whether it's uh, "I quit smoking last year." And you're like, "oh great. How'd you do that?" And it's like, "oh, I went to jail." You're like, "okay, well." Um, or they um, had a test that was positive while they were in jail and they need to come get follow-up and things like that. Um, the only time I directly ask is if there's some sort of indication that it might have, be um, a part of their history. And uh, and I'll often ask, you know, what it is they were charged with, mainly because I want to pursue things like addiction and um, see if we can help treat those things. But literally people most of the time are like, "I, I was on paper and I broke paper." Something like that. And I was like, "okay, I think I know what that means." (laughs)

So, um, uh, so I, if it's pertinent or if in some way it's indicated by the history, I'll ask directly.

I: Mm-hmm (affirmative).

P: Otherwise, not so much.

I: And then once you have that information, could you talk a little about how that informs your treatment plan or your approach to care for that patient?

P: Um, two things. Um, infectious disease risk, so things like increased risk for TB, and we unfortunately live in a time where things like TB and syphilis are just raging back. Um, so it, it's one of the things where I want to make sure I evaluate appropriately, and if they've been incarcerated, um, I want to make sure that their tuberculosis screening is up to date.

And also um, the other way, like I said is if I find out, uh, something that might indicate there's uh, addiction history then we actually have a pretty rigorous and effective, um, medical uh, treatments that we use at our clinic. We use Suboxone and we have uh, access. Did we Rule 25? No, we can't Rule 25 yet.

We have a licensed chemical dependency counselors and things like that, so if I feel that that sort of hints towards that, I'll try to steer them towards support that can help them.

I: Mm-hmm (affirmative). And are there any challenges that you see to broaching this topic in conversation with your patients?

P: Uh, you know, I'm a middle age black lady, and most younger people will just tell me whatever I ask them.

I: Mm-hmm (affirmative).

P: So I just have to be respectful. If I'm respectful, they're fine. Even older people. I'm like right in the sweet spot. Got a little gray. Excellent. I can do the like, lean the head thing. "So, can you tell me about this?" And people just spill. Yeah.

I: Mm-hmm (affirmative).

P: So, and I try to, I try to teach um, residents and interns and students uh, to be direct but you know, re- do it very respectful um ... (laughs) I try to let them know, you don't say what did you do? You say, what were you charged with? And um, and, and discuss why you would ask that of a patient. And be able to help them understand how to relate to a patient. Like, this is something that may have, may impact your health in some ways, and so I just want to know, and see if we can help you in some way.

So, um, respectful, direct, and a reason. If there, there should be some sort of reason directly related to their health. Yeah.

I: And then on the flip side, are there any benefits that you're seeing to having this information and having those conversations?

P: Um, well yeah, cause I think if you do it in a respectful way, the, it helps create your therapeutic alliance. Because they're demonstrating that they trust you in some way, and you're demonstrating that you're trustworthy. So I think it can help build trust if it's done in a way that the patient feels safer. Cause honestly, I don't, I don't need people being ashamed of their past and hiding things, cause once you start hiding one thing, you hide more stuff, so ...

I think it, it can contribute to creating a safe space.

I: And next could you tell me a bit more about the overall patient population that you're seeing on a day to day basis?

P: Yes, so we are, our outpatient clinic is [community health care clinic], and we are [community health care clinic address] right off of uh, well, [street name], and we're in the heart of [city name], and we've been uh, part of the neighborhood for I want to say about 45 years or so. And our neighborhood is primarily African-American and then it's about 20% um, immigrant of some sort, and then the random white jogger. Um, and uh, it's, it's probably going to be the last place to gentrify in [city name], but it's getting a little gentrified, which is not so great for housing.

um, it is a neighborhood in which 50% of households don't have cars. Uh, it's a neighborhood in which there is uh, significant challenges for on time high school graduation. Uh, the average age of the people I think is about 27, and uh, so we skew very young. We have a, a, a very, um, we have an active OB service, I'll just put it that way. (laughs)

We deliver lots of babies for our patients, which is nice. Um, we have folks that are in a low-wealth community, and uh, face a lot of the challenges associated with that including food scarcity, um, home instability, frequent school changes for the children. Um, so it has all the challenges that you imagine for multi-generational low-wealth community.

I: Mm-hmm (affirmative).

P: But it's also very vibrant. There's lots of different organizations that are um, from the neighborhood that are all about helping people help each other, and I think that's really cool.

I: And you mentioned, um, it's primarily low-income. I'm wondering, um, if you could talk a little bit about the insurance status that you're seeing?

P: Oh, we see (laughs). Our payer mix is mostly Medicaid.

I: Okay.

P: Except I think I got on some sort of Medtronic list. I got all these Medtronic people showing up, and I like walk in the room, and I'm like, "you're employed. with a good job." I'm always like, "so, what brings you here?" And they're like, "oh, I live in Northeast. This is close." I'm like, "okay." Um, but yeah, so we're primarily uh, uh, medicaid. Our, our private insurance mix is very low. And we actually don't have a ton of Medicare either because the neighborhood does skew a lot younger.

I: Mm-hmm (affirmative). And then how would you describe the disability status of your patients?

P: Uh, we have ... Well, it's sort of cool being a family physician, because you get to take care of people for their whole ... throughout their whole life. So um, but what we see a lot in our patient population is early onset of multiple chronic illnesses. Metabolic syndromes, hypertension, diabetes, um, cardiovascular disease, uh, all at much younger ages. Um, there's a very big obesity issue. Uh, there is um ... And then, and then like I said, we also get the, we do a lot of wellness and you know, we see a ton of kids for well child. There's uh, disproportionate amount of asthma in the community. Um, and we have diabetes presenting in some ... Type 2 diabetes presenting in, in much younger kids than most places.

Uh, we don't have a huge elderly population. Those that we do generally are either hypertensive and/or diabetic. And um, have all the sequelae of multiple chronic illnesses. Um, but then I also have a population of older people that are just like, you know, vigorous and employed and happy. So it's a, it's a nice, it's a, it's a ... You know you're needed, but you also get to be there for the healthful changes that occurred so it's nice. Yeah.

I: And in your experience, are you noticing any challenges that racial and ethnic-minority patients in particular are facing in terms of their access to care?

P: (laughs) Well, hmm. This is all ID identified?

I: Mm-hmm (affirmative).

P: Okay. So (laughs) they have a lot of micro-aggressions. Um, our patients so if you were our patient and you went to the emergency room (points at notetaker who is white), and you are a patient and went to the emergency room (points at interviewer who is African American), you would definitely get drug tested (points at interviewer who is African American). And you probably wouldn't (points at notetaker who is white). Even if you came for the exact same thing. Happens up on Labor and Delivery. Um, it, it's all the same assumptions that people make that, it's more, uh, our our African-American patients are more likely to have drug-seeking behavior as one of their problems listed or something like that.

And um, and there's ... This is one of the weirdest things that I've noticed is that um, grief is done very differently in the multi-generation Minnesotans versus African-Americans who, a lot came up from the south and things like that. A perfect example. When um, when I was first here, our clinic manager died just in a random accident. And they got everyone in, our whole staff, into the break room, and they, they announced it. And I just moved to Minnesota. And it was striking, the different responses. Our, most of our nurses and MAs are African-American, and most of our docs and staff, they're old school Minnesotan. And old school Minnesota, they just go into sort of quiet. And wailing and mashing of teeth is appropriate in the African-American culture when you're grieving.

It's appropriate to be loud when you're grieving. And um, that can be a challenge in a hospital uh, when security doesn't understand that that's just natural grieving. We're like, she just needs to be in a room. Take her to the family lounge. You don't need to call security. Um, but yeah, it's like, the differential, the cultural differences that drive a lot of misconceptions are just almost polar opposites. And that day, when I was in the break room, I was like, I was like, this is going to be weird because these people don't do emotions. And these people think doing emotions um, loudly and vigorously is appropriate, and it's just two different approaches.

But the white people have the security and the police, and if you're grieving too loud, someone might call the security person. And um, I have had a situation like that, and it was just one of those things where, you know, the security officer was, wasn't out of hand, but he was, he was a little more vigorous in addressing this woman. And I just had to stand between them and say, "listen, I'll take care of it." And we got her into a room and so she, she just found out that her husband died. Like in front of her. She's allowed to feel bad. Um, but yeah, so I think culturally, there's a lot of differences and there's just these presumed racist bullshit. It's like, we're all doing drugs and we're all criminals.

Um, I was walking from the doctor's lot to our clinic with my white coat and my ID, and the security guard's like, "show me your ID." And I'm like, "why do I have to show you my ID?" He was like, "well this is faculty parking." I'm like, "I am faculty. I've been here longer than you." Ugh. Anyway, he did not last long.

But yeah, so it's like, we always have to prove we belong where we belong, and um, I, I think our patients ... This, we have lots of patients who are just like, they love this hospital. They've had multiple generations in this hospital, and they'll keep coming back. But there, there's also is just these little, like I said, there's assumptions that are made, and um, and we're trying to work on it. And I, I feel like we're getting a lot of cooperation with administration here and nursing here and like things like that. But we just, we just keep have to bring stuff up because people don't see it. They're like, "what do you mean?" No, like that's offensive to do that. You only do those tests if you need it.

So. Anyway. Did I answer your question?

I: Yeah.

P: Okay.

I: Thank you. So next I'd, I'd like to shift back to thinking about your patients that have some type of involvement with the justice system and that experience and um, are there ways, um, that you think that having this justice system involvement has impacted patients' ability to access care?

P: Um, it's interesting, cause, cause I was thinking about a gentleman that I had a couple years back, and um, he ... I remember he came in and he wanted, like, "I want to get my diabetes under control cause I'm turning my life around. I want to start a business. I want to do" all these things, and I was like, "okay." Um, and he was like in his early thirties. And I said, "so why now?" And he goes, "well, you know, I want to be there for my kids." And he would just, you know, he just, he admitted, he's like he'd been involved all in his teens and twenties in and out of jail. And maybe even prison at one point. And um, he's like, "yeah, I don't want to do that anymore." I was like, "all right. That's great."

And then, you know, we worked with his diabetes and things like that, and then he just couldn't get ahead. Like his criminal record really got in the way of the choices that he was able to make. I mean, this is back when you couldn't even, even at Target you had to check the box if you were, um, at any point, uh, convicted of a crime. And, you know, it's really hard to get employed if that's one of the barriers. And this guy, he's like, "yeah, no, I messed up when I was young, and now I want to get my life together and I can't do it." And it did not go well for him. He eventually just, just spiraled out of control, but he just was like, there was like a couple years there where he just really wanted to do the right thing. And everywhere he went there was like a big roadblock.

And I was like, "could you maybe tell the younger guys about this?" (laughs) because if we started earlier, maybe we, we could avoid this. And he was like, "yeah, this is just not good." He so wanted to, like he so wanted to just do it right. And yeah, and then he sort of fell out of our um, clinic and uh, bounced around lots of different places, and then like, a couple years ago, I saw him again. And he'd had multiple amputations, and he was just wasting away. And I was just like, man, this is just, this person really wanted to do the right thing, and now he's just an invalid with no ability to even ... At that point he couldn't even care for himself. It's just, his diabetes got so bad out of control. He dropped out of like, uh, anyway.

But long story short, I see people who made bad choices when they were younger, and I see that it's um, it's impaired their ability to make the better choices as they get older. And also I see, like I said, ACES, one of the big ACES is someone in your family going to jail. And you know, it's, it shifts around families, the kids staying with grandmother, um, kids not necessarily having all their parents in their lives. And that, that is a challenge. So. And it's a challenge that affects your long term health. I mean, you're at more risk for chronic illness. You're more at risk for addiction. It's just this self-perpetuating cycle. Yeah.

It's so much easier to make the bad choices, and that's why I look at it as systemic. It's like we think it's like all these people making their individual choices, but like, if the ... Like that guy. Like if the system was set up for him to in any way achieve, he would have, he would have, he made the choice. He just didn't have the hoops to jump. They're just like, "no, there's not even hoops for you to jump through. There's no hoops." So I look at is like, there's a lot of things about this system that makes it easier to make bad choices, and it makes it easier to just ... it makes it really hard to turn your life around. So, anyway.

Does that answer your question?

I: And then as a provider, are there any instances where you've had some type of communication with the criminal justice system, whether that's talking to someone's probation or parole officer or um, connecting with the courts in any way?

P: Um, not in Minnesota. No, um, peripherally? Yeah, no, not me personally.

I: And did you have ... were you doing that outside of Minnesota at all?

P: Well [state name], I was-

I: Mm-hmm (affirmative).

P: In the system, so I was part of it, so yeah, in that point, um, and we even, we delivered the prisoners' babies too. And that, man, these ... Like, it was, we tried to make it not the case, but sometimes you'd like go in and you're, you know, someone's laboring, and they're like handcuffed to the bed. You're like, that's just not a good human thing to do. I was like, and they're like, "well, don't want people leaving." I'm like, "they're not going to leave. They're in labor." No woman leaves the hospital in labor. (laughs) It's just not a thing. So, but yeah, but they eventually stopped that.

Um, and it was interesting because I remember one of the things that was sort of cool is that a lot of the, a lot of the, most of the guards were just really nice people. They were good folks. And I remember this one guy, um, he'd brought, cause he had always escorted prisoners whether it was to clinic or they went to hospital. I remember him, he was, he was standing up for this patient. He was like, "you know what? She's not a complainer. There's a lot of folks who are complainers but she's not a complainer. If she's saying this is, something's wrong, something's wrong." And I was just like, you know, I was like, that was so cool. He like stood up for the person, he's, you know, supposed to be guarding.

I: Mm-hmm (affirmative).

P: But he's like, you know, they get to know folks and he's like, "yeah, no, there's something wrong." And there was. But it was nice to see that he cared. And so, um, did I answer that question?

I: Mm-hmm (affirmative).

P: Okay good.

I: And then aside from um, justice system involvement, what else are you seeing your justice-involved patients dealing with socially? I know you provided that one example with the patient who had trouble finding a job. Were there other things that you're seeing?

P: Um, I'd say employment, education, and addiction. So um, a lot of times kids get involved earlier than they'd like in the criminal justice system and it impacts their ability to complete their learning, which then subsequently impacts their ability to be employed. Um, addiction issues are other places um, school suspensions. I always get really scared um, the kids in [neighborhood] probably still ... this was a few years old data, but it was the um, neighborhood with more repeat middle school, uh, suspensions, which is often one of the first steps in school to prison pipeline because you don't keep up because of all the challenges of what's going on. And then once people just get behind, it just often spirals out of control.

Um, so yeah, addiction, education, employment, are probably ... and then, just like I said, not being there for their family or trying to get back to the family after not being there. Also an issue. I-

I: And then what are you seeing them dealing with medically in addition to the addiction that you mentioned?

P: Um, all the chronic illnesses.

I: Mm-hmm (affirmative).

P: Um, diabetes, hypertension, smoking. Big. Um, and uh, oh, multiple chronic illnesses. And um, even uh, obesity and you have sort of the sequelae of dietary choices. Yeah, so um, yeah. It's like all the, all the challenges all my other patients face, but it's almost sort of accelerated.

I: Mm-hmm (affirmative).

P: Yeah.

I: And are there any mental health-

P: Oh yeah (laughs).

I: Needs that you've seen?

P: Yes. Yes, yes. Yes. Depression anxiety and anxiety, I think are the ... And PTSD. So many of these folks are PTSD. It's just like, it's hard to tweeze apart what ... It's like I don't even feel like we need to have one label. (laughs) cause it just so feeds into itself. We have the folks who have PTSD, anxiety, depression. Um, the guy I spoke about, he actually eventually had, had a psychotic break, and that contributed to bad things. Um, but yeah, I think PTSD is rampant, and I think that major depression and anxiety ... A lot of the anxiety is untreated. I think more anxiety is untreated than the depression, although, it's hard to say. But yeah, not, yeah, those are the big three I'd probably say.

I: Mm-hmm (affirmative). And then are there any resources or services that you're finding that your patients need but that aren't available to them?

P: In regards to?

I: Um, any of the social factors perhaps that you mentioned or-

P: Uh-

I: Things that you think that you would wish that you could refer them to but you don't have a place to send them.

P: Um, I feel ... We ... Uh, we have a lot of resources and accesses, access to getting folks resources. I, I, between us or [community health care clinic], which is the closest FQHC, I feel like honestly the one that I think that I'd really, really like that even the folks in the neighborhood don't know that they probably need this. There's, there's a marginal level of literacy for some folks, and I really think it impacts a lot of people's choices. And I think that they don't quite know that that's one of the things that's impacting. I've ... A lot of patients who, you know, just survived high school, and whether or not they graduated, they're education level doesn't adequately reflect their intelligence. I mean, bright people who just didn't get the kind of support and opportunity that would have helped them better.

So honestly, I would like to do literacy stuff, but I don't even think there's ... The literacy programs that are around that I've done, sent patients to, are not in the north. Unless there's something new that's been happening, but in the last couple years when I've sent people, they've always had to go outside of the neighborhood.

I: And then thinking broadly, are there any changes to healthcare delivery that you would suggest to better meet the needs of patients that have justice system involvement?

P: Yeah. I think it would be really, really good if electronic medical records could talk to each other, and if um ... Like if my patient goes to jail or prison, that, you know, we know what worked for them, continue those medications or when they come out of jail or prison, you know, if they've been treated for something or you know, started on medications, it'd be great if it was like, if there was a little more seamlessness with the, with the records. Which would solve a lot of problems for a lot of people. But yeah, so, I don't know what goes on. I don't really know how to get information from the jail or prison. I would probably go to my medical records person, and be like, "can you get these?" And she might be able to get them.

But yeah, I don't eve- I don't think I've ever seen a medical record from jail.

I: Mm-hmm (affirmative).

P: Which, we should probably do differently. Cause we sort of just say, "oh, they received this and they said this." And I was like, "if they came from [community health care clinic], we would have gotten the medical record." That's a blind spot. That's not good. Okay, we're going to change that. Yeah, we never ask for medical records for prison or jail. That is lame. (laughs) Okay. Policy change.

All right. What other kind of stuff? Anything else? Stuff, more, did I answer it?

I: Yeah, unless you have any other suggestions on how you would change healthcare delivery?

P: Oh, well I've got ideas.

I: (laughs)

P: Yeah, no, all of this would be so much easier if we just had universal coverage for everybody, but um, that's, that's another interview. (laughs)

Um, yeah, I, I ... What, do you ... Here's a question. What was the impetus for the project?

I: Um, I, where to start? So part of it is that we ... Our hypothesis is that the same folks that are falling through the cracks in our healthcare system-

P: Mm-hmm (affirmative).

I: Are falling through the cracks in the criminal justice system, that they're not-

P: Come in. Sorry.

Speaker 3: Hi, sorry to interrupt. I just want to grab my lunch.

P: We're almost done.

Speaker 3: You can stay as long as you want. I just need this.

P: All right.

Speaker 3: Thank you.

P: Welcome.

So, that's interesting.

I: They're not, they're not mutually exclusive and the folks that are high utilizers in our ED or are being hospitalized are likely the same people that are spending the same amount or a large amount of days per year in the jail too.

P: Yeah.

I: And that if we want to try to tackle both, if we want to try, you know, like to address our high healthcare utilizers, we can't just focus on the healthcare system-

P: Yeah.

I: We need to be looking at the criminal justice system. Um, we need to look into housing, food and cash support, all these other social factors that are going on.

P: Yup.

I: Um, because it's not just one silo alone.

P: No. (laughs)

I: And there are multiple people who are moving through both of these sectors, um, and we just, we need to look at the big picture and not just focus on one.

P: Yeah. No, I'm a fan. That sounds good. And it seems like a good hypothesis. Yeah. Um, all right. Did you get what you needed?

I: Yeah, is um, before we wrap up, is there anything else that I didn't ask you about that you'd like to add?

P: Um, yeah, no. I'd probably say that I think one of the, the places we don't get as verbal about is the impact of incarceration on the family and the kids in the family. And um, like I said, jail is not a nice place. You don't, you don't feel safe or good there. And um, and I just yeah. It's really a super broken system. So, if you could fix it, that'd be awesome. I'd be good with that.

I: (laughs)

P: Yeah, yeah, no. That's all I got. I hope it was helpful.

I: Thank you. Yeah.
