# Supplementary material for: Physicians’ perspectives on continuity of care for patients involved in the criminal justice system: A qualitative study
Source: PLoS One. 2021 Jul 14;16(7):e0254578. doi: 10.1371/journal.pone.0254578 (PMC8279398; doi:10.1371/journal.pone.0254578)
Supplement: S2 File — (ZIP) [file pone.0254578.s002.zip › Clean/Participant_23_Audio2_LJ_deidentified.docx]

I: Started.

P: Okay.

I: All right. So thanks again for taking the time to interview with me today. Um, like I've said, this is a project that's a joint partnership between [health system], the [University], and [County], where we're exploring, um, this relationship between criminal justice involvement and health. And the goal of this interview to get a sense of what you know about the criminal justice system, how it may impact your patients. Um, so I'd like to be begin by getting just a general overview of what you know about the justice system. So to start us off, could you tell me what you think of the current state of the criminal justice system in the U.S.?

P: That's a big, big question. Um, I, what do I think of it? (phone rings) Excuse me. Of course. Um, well, I mean, I'm not very happy with the criminal justice system. I think that there, the focus on imprisonment, especially, like, private and for-profit prisons, is really, um, a terrible thing. Drives incentive to incarcerate more people and makes the focus less on rehabilitative processes. I'm also really, really don't like the, um, like, the punishment for drug use in this country, the country. Like, um, you know, in general drug offenses mean, like, causing long, um, prison sentences rather than leading to rehab and reintegration into society, and I guess I could say that about almost really every offense, um, in this country. It just seems like a lot of things are for, uh, or cause, um, long prison sentences, which aren't helping anyone, um, actually stay out of prison or have productive lives afterwards.

P: And then, you know, pretty, pretty clear, um, racial inequities in, in prison sentencing I think is also terrible. So, yeah, I think in general I don't have a lot of faith or confidence that our prison system is fair and equitable for Americans, or is really, like, effective at doing what, what the goal would be, which is to, um, keep everyone safe and so, yeah, I think that sums it up. It's a big question.

I: Yeah. Thanks for that. And so next I'd like to discuss some criminal justice system terminology. Um, could you explain to me what you, what comes to mind when you hear the following terms, and there's a few of them I'm going to go through, and the first is prison.

P: Uh, prison would be for longer term and more serious offense, uh, more serious offenses, for people that have broken the law.

I: And how about the term jail?

P: Jail, um, shorter term. I would say one, prison, too, I think more, like, state and federal, and jail I think more of county. So, like, shorter term, less or lower level offenses.

I: And then how about the term probation?

P: Um, some, the punishment for a law that was broken that doesn't require jail sentencing, um, and it, you know, requires something of the person, um, who was sentenced. Um, so some, like, set of activities and checking in with, uh, officers.

I: And then how about the term parole?

P: Oh, geez. Um, parole is, not exactly sure. It's, uh, when somebody is released from any of those three, um, any of those three places, yeah, but I'm not exactly sure the exact meaning.

I: Mm-hmm (affirmative).

P: Mm-hmm (affirmative).

I: Thank you.

P: Mm-hmm (affirmative).

I: And so are there any, in terms of thinking about probation and parole, um, are there ways that you distinguish between the two?

P: Um, well, it's hard, because I don't really know what the difference, or what parole is. So it's hard for me to really answer that question. I mean, yeah, my thought would be that, like, probation is like a set of things to do and then parole is something to do with release. Now I want to, like, look it up with my phone and figure out exactly what it is, but I'm not sure, so.

I: Yeah, so next I'd like to talk a bit about your background and your education and your training.

P: Mm-hmm (affirmative).

I: During medical school, did you ever receive any training, whether it was formal or informal, on working with justice involved populations?

P: No.

I: And do you think that there would have been any training or education that would have bene helpful to you during that time?

P: Um, yeah. I mean, I think that that's one of many misses in medical education.

I: And thinking about the content of that education and training, what are some things that you think you would have liked to have seen?

P: Um, well, I mean, I mean I think we just touched on one right, right? It's just like general terminology and ideas. Like, I think one data on, would be nice to know, get data and statistics about, like, the types of populations and, um, how those, how that populations in jails and prisons are cared for. Um, and then, like, I think any particular kind of special interest or areas of healthcare that are higher needs for that type population. I think, yeah, I think that would be a good educational start, you know, to get a little overall, like, demographic information. Um, I mean, in my mind, it would be nice to learn a little bit about the inequities that lead to some of, like, that, the demographic, um, that is mostly in jail and in prison, and then, uh, I think learning about any kind of particular healthcare needs would be, would be helpful as well.

I: And then during your residency, similarly did you receive any formal or informal training on working with justice involved populations?

P: No. Nope. Yeah, it's only, I mean, I guess you could say informal in that I had some patients that were, um, recently released from prison and I've had patients that I, you know, don't see for several months and then come back and say, "Yes, I just got out of prison." Care for, you know, continue caring for them. But that's usually the extent of what happens. Like, there's not a lot of, um, there's not a lot, I mean, it's just to focus then again on healthcare. It's not, it's not kind of going into any particular issues that might have brought up or anything like that.

I: And then as part of your training, did you complete a fellowship?

P: No.

I: Okay, and so now thinking about your current or past place of employment, again, did you ever receive any training in that setting on working with justice-involved populations?

P: Nope. I mean, the same. Um, yeah, yeah. It's the same thing, just kind of informally through patient interactions, but no, no specific training at all.

I: Mm-hmm (affirmative). Could you speak a little bit towards your patient interactions and what you're learning there?

P: Um, like in those informal-

I: Mm-hmm (affirmative).

P: ... settings? Well, a lot of that, for me, has looked like caring for people with a substance use disorder. Um, that, I mean, that kind of varies, you know. Sometimes they're stable and they have a relapse and end up in prison because of that, or sometimes it's an old, something old that's been following them for a long time. Um, so that's interesting, because there's a, like, generally there's not a lot of support for substance use treatment, at least in my experience with the patients that I have, while they're in prison. But once, you know, it doesn't, so that doesn't help them kind of progress or improve their healthcare while they're, while they are incarcerated. So on release a lot of times it's a scramble to try and get back and figure out how their life is going to work and continue the treatment that they had started previously.

P: Um, so, I mean, I think that's the biggest, but I know I've had several patients, when it's not substance use disorder, it's really just, I feel like it's a little, I'm not, um, I don't know. I think there's just enough stigma that it's not, like, it's not something that they're really willing to talk about, like, any experiences, and I think for the most part I've just been able, or just kind of, like, leave it there. I don't, I don't try and probe on any particular issue. So really it's just kind of standard visits and healthcare related stuff, and not kind of delving into any issues that may have arisen because of prison or being incarcerated.

I: And so related to that, during your visits do you ask patients whether or not they have some type of current or past involvement with the justice system?

P: Um, no. No, I don't have any, like, there's not, um, yeah, I'm not, like, screening or anything if that's what, there's no, like, kind of formal screening. Usually that just comes up as I get to know patients. I should say one other thing that I really have noticed is, um, housing a lot of times for people who especially have had felonies in the past, no matter what length of time that felony has been, housing is a real problem.

P: I think there's supposed to be some kind of limit around 10 years, like after 10 years you don't have that- or, I don't know if this is standard for all, if that's like a law or it's real estate or what, but according to the patients that I'm talking to, even if it's in the remote past, 15, 20 years ago, housing is a real problem. Um, like being able to apply to and get housing, among other things. I mean, jobs, et cetera. So that is another way that I informally brush. Again, it's not like I'm screening, um, for certain things, but as we're running into barriers to healthcare, it's like, well, yeah, housing is a real problem for me. I can't get housing because I get denied, because I see, they see I was a felon. So it kind of throws all of their aspects of healthcare into trouble.

P: So, yes, I double answered, or over-answered that question and the real answer is, no, I don't formally screen or don't have any process like that.

I: Yeah. Um, are there, um, any reasons, um, why you are, are you, why you're not screening or have you ever thought about asking that question?

P: Yeah, I don't, I mean, I don't, in primary care in general, I think that's a tough question, because there's so many things to screen for and we, at the end of the day, have such little time to screen for all those things. I think that is, that's one answer. It's just strictly function of screening for everything you should be screening for, and whether that really belongs, like, in your 20 minute office visit or if that's more, like, a function of a care coordination type position. That's a bigger structural question.

P: I'm not trying to be, because the other non-evasive part of that answer, rather than blaming systems, is, like, the stigma attached, I think, and worrying that that will, um, throw up some barriers, especially in patients that I don't know well. Um, that they may feel judged or something just by strictly, no matter what intent I have with asking the question which, for me, it would be, you know, strictly as an informational thing or to try and provide extra services to them, but sometimes I think coming, um, coming from a physician, especially a white male physician, that can be seen as, like, "Oh, you're going to judge me for my past." So, um, so yeah, that's, I mean, I think that definitely is another reason why I don't, I wouldn't routinely ask about that.

I: Mm-hmm (affirmative). And then for patients where you are aware, um, are there any benefits that you see to having this information?

P: Well, I mean, that's a good question. Like, yeah. I mean, there certainly could be. Um, so, I mean, in the, like, it just depends on what you think about healthcare in general again. Um, so, you know, it doesn't make a difference for me if I'm treating diabetes or high blood pressure or anything along those lines medically, but if I'm trying to assess barriers to care and, um, and trying to think about different things that might affect their ability to follow through with treatment plans or access certain things, um, that I'm suggesting, then it does matter. Um, and that's kind of, that, again, brings back, like, a bigger systems question of, you know, being formally in prison isn't the only thing that affects those types of things, right?

P: But, um, I think that is the same, or that, a good example of that is the housing thing with other patients, where they've had such a barrier to housing because of their, because they're an ex-felon, um, that then they have trouble doing or, you know, trouble accessing certain, I don't know, there's any number of things, but really following, following medical plans is very difficult because their housing is so unstable. So I do think that, you know, like in the, it depends on how you want to qualify doesn't matter. It doesn't matter for strictly medical treatment, but it definitely, or for the medical recommendations, but it definitely matters, like, following, being able to follow through with that and, yeah, just receiving the best healthcare that they can get. I think it makes a difference for that.

I: Mm-hmm (affirmative). And so you mentioned the stigma associated with this and talking to patients about it. Are there any other challenges that you see to talking about this with your patients?

P: Um, yeah, I mean, I think stigma is a big one. Like, I feel like patients aren't really forthcoming with the fact that they have been previously, and I'm, I guess my assumption is that that stigma or fear of judgement, but I'm also, I mean, I also think, I mean, there's potential trauma, like, reliving of trauma. I don't know, you know, not everybody has a terribly traumatic, well, maybe, I don't, I don't know. I don't know enough. I would think that any, any, any period of incarceration, interaction with the justice system, has some, probably has some negative connotations that might be somewhat traumatic to a person. So bringing, bringing that up, especially in a visit, like, in a quick family medicine visit, um, primary care visit, is going to, um, you know, are you, in that 20 minute visit are you going to have time to attend to any trauma that that brings up?

P: So I do think a lot of times we are careful because we, it, dealing with that stuff in a, dealing with trauma in and of itself, I mean, one, our training isn't the greatest for doing stuff like that, but we're certainly able to be a compassionate human being. But it's also, like, do I really have the time and the presence to attend to that right now as well? I think we need to be a little bit careful about that. Um, so, I do think, yeah, like, concern about bringing up stuff that we might not be able to fully attend to or care for, I think, is a real concern with that.

P: Um, I’m trying to think, anything else? Yeah. Not off the top of my head, anything else.

I: Okay. So next I'd like to zoom out a little bit and think about your overall patient population. Could you tell me about, more about the typical patients that you're seeing on a day-to-day basis?

P: Mm-hmm (affirmative). No other guidelines as far as, you just want to know general demographics?

I: Mm-hmm (affirmative).

P: At the clinic, um, it's probably around, I think especially for the patients that I am seeing, it's probably about three-quarters African American patients, and then I think at our clinic itself, the next highest racial or ethnic group we see is Hmong, and that's probably 19 or, something like 20 percent, and then just a smattering of a bunch of other groups. My own population, so because I have, um, a certain number of clinics and have been around a while, practicing a while, I think more of my population is African American. I don't have many, many Hmong patients.

P: Um, so, yeah, that would be, like, demographic as far as racial and ethnic. Almost all of our patient population is low income as well. Say, I think 80 percent of our population is on the state insurance, either PMAP or Medicaid or Medicare. Um, yeah, and then in general, the clinic population swings towards younger, so kids and young women. However, again, I think the longer you're here the more, I think my, or my personal clinic population is kind of a mix. I have several older patients that I see regularly as well as, you know, young women, children, families.

I: And how would you describe the disability status of your patients?

P: It's like what percentage is on disability or? Interesting. For the patients that I see, yeah, by disability you mean Medicare, like, SSI?

I: I've actually, I've been leaving the definition of disability up to-

P: Sure.

I: ... participants to define for themselves and how they think about it.

P: Yeah, yeah. Well, I mean, there's a bigger philosophical question then, too. I mean, so if you would use the, like, legal definition of disability and how many people are on, like, Medicare, SSI, one, I don't really know. I will give really a gross estimate of 15 to 20 percent of my personal patient population. Um, I mean, it's, it's interesting that when I think about the rest of my population, I think about just other, um, other parts of the clinic that I interact with. I think probably a greater percentage might, um, might be, might be disabled in some way in that, like, you know, by, like, unable to really be competitive in a work force without kind of intensive training, stuff like that, and a lot of that, I think, I would say is, in general what we run when patients are really struggling is generally mental health with a heavy trauma history that, I think, really makes it difficult for them to, um, to hold a job.

P: And some of those have gone through a process and have received, um, like the formal disability, or diagnosis, disability benefits, I guess? And some have not and, um, that's always, or, you know, it's kind of a tough thing to see, I think. Sometimes the people that function less well have more trouble, like, jumping through all the hoops needed to get that disability designation, which seems like it'd be helpful for them in general, to make sure they have the services that they need to keep surviving.

P: But, so, yeah. All that to say, you're, what was, the original question was how many?

I: How would you describe-

P: How you would describe-

I: ... their disability status?

P: Yeah, disability status. So, yeah. So, I mean, I think I see a lot of people that have some level of disability status, whether that's the formal diagnosis versus people that just really are struggling to function well. Um, it's hard to put percentages on that, though, but maybe a quarter of my patients or something like that-

I: Mm-hmm (affirmative).

P: ... have some kind of struggle. Is that enough?

I: Yeah, and then amongst your overall, um, patient population, have you noticed any particular challenges or barriers faced by patients from racial or ethnic minority backgrounds? Recognizing that the majority of your patients are from-

P: Yeah.

I: ... minority background.

P: Right, so anything particular because of that?

I: Mm-hmm (affirmative).

P: Well, I, the, you don't want just a yes or no, I mean, yes. Yes, I noticed that.

I: Yeah, and then an example or-

P: I mean, take your pick. I mean, um, I mean, incarceration rates is one of African American men, like, the structural racism that plays into that I think we see all the time. Um, I think, uh, the education disparities that we see in the kids, like, the services available, the schools that they're going to, everything surrounding education in Minnesota. I think we see that play out a lot in the young kids that we are taking care of. I don't know, couple examples.

I: Mm-hmm (affirmative), and then are there any in particular to their ability to access healthcare that you're seeing?

P: Um, hm. Specifically talking about are there racial barriers to accessing healthcare?

I: Mm-hmm (affirmative).

P: Hm. To accessing quality healthcare? You just said healthcare in general. Well, I mean, again, it's a, that's a tough question to answer, because is it, is it truly a barrier based solely on race? I mean, in a, again, what you just said was a zoomed out sense. Like, yes, like, there's a lot of just structural inequities overall that lead to poor healthcare for our clinic population. You know, accessing our system as a whole, I, you know, I don't think there's, like, healthcare in Minnesota, the, in general we have better access than a lot of other states. I mean, more people are able to get on insurance and get, um, and, and access healthcare, but I think, I think, yeah, I would come back to that kind of larger issue.

P: Like, it's just, there's, it's every, it's not particularly healthcare, but there's a lot of things kind of preventing the accessing of healthcare, whether that's inequities in housing, in loans and education, all that type of thing is what causes, is, you know, structural things that cause those types of inequities. And, yeah, and then all that leads to poor health outcomes. Um, yeah.

I: So next I'd like to, um, go back to thinking about and talking about your patients that have some type of justice system involvement in particular and what that experience is like for you as a provider. Are you getting any that, patients that are specifically referred to your care?

P: Um, okay. So referred to my care. Like, from other providers? Not currently, although that's in the works, especially with any, with substance use disorder in general and specific opiate use disorder. That will be happening, I think, more now, but our, um, but, no. Other than that there's no, I think, formal, uh, there's certainly no formal, like, mechanism of referral to either myself or our clinic from anybody involved with the justice system.

P: Now, you know, informally through our existing patients, certainly, and then I think, you know, sometimes the hospital we're associated with I think kind of views our clinic as, like, more of a safety net clinic and more for certain patients, will refer patients our way. So those would be the only two instances I can really think of, but, yeah, no, nothing formally.

I: Mm-hmm (affirmative), and are there ways that you think that having justice system involvement may have impacted your patients' ability to access and receive healthcare?

P: Yeah. I mean, I guess I covered that a little bit earlier with what I said about, um, like, just justice system involvement. It's really hard for somebody to engage in healthcare in any way when they are living in, you know, when they can't find a stable place to live or they, you know, can't, um, they aren't considered for steady jobs or anything, because they, because of their history. Um, and, you know, I think a lot of that kind of plays into just a whole, when those things are happening, it's hard to figure out what the meaning and purpose of life is as well, you know? There's kind of some kind of base level degradation that, again, makes it difficult to engage in, in healthcare. So, yeah, I think it's, I think it's a struggle for patients when they're, when they feel a little devalued in general by society based on something they did, sometimes in remote past. Makes them kind of, so then, yes, it's a barrier.

P: Again, it's more of an indirect barrier to healthcare. It's just like they have to feel like you want to be, want to feel good and you deserve to feel good and you should take care of your health, because there's goals that you're trying to achieve, and if those things aren't out, then it's, you don't engage in your healthcare, so.

I: And then are there ways that you might adapt your treatment plan for a patient to take those other factors into account?

P: Well, yeah. I mean, all the time. You're, I mean, you're trying to meet a patient where they're at, right? So if I'm recommending some treatment for diabetes and the patient is saying, you know, "I can't do that," or more often what happens, they say they can do it and then they don't, and then we discuss why. And it's, like, "Well, I've got all this stuff going on." Like, okay, well, we, you can't do that, right? So we got to figure out something else. So there's always an adaptation to patient circumstances, desires, wants, needs, you know. It's not, it's not usually about what I want as a practitioner. It's about what, trying to come to an agreement of what's best and, you know, that balance isn't always easy to strike either for me, because even if a patient doesn't want to control their blood pressure, I know how dangerous having high blood pressure is over the long term. So, yeah, we're trying to find compromises that will help them in the long run.

I: Mm-hmm (affirmative). And then earlier you spoke a little bit about substance abuse treatment-

P: Mm-hmm (affirmative).

I: ... and how incarceration impacts that. Could you talk a little bit more about, um, what that means for a patient and their treatment plan and how you adapt a treatment plan for someone who has a substance use disorder?

P: Yeah, well, um, it's, it's rare. So, I'm sorry, why don't you say your question one more time so I make sure I get it.

I: Yeah, so you mentioned, you were talking about substance use disorder and not having access to treatment while incarcerated and how that changes up the treatment plan.

P: Mm-hmm (affirmative).

I: Could you talk a little bit about how that impacts you as a provider and then how you go about addressing that patient's treatment plan?

P: Yeah. So usually in, I think there's only been a couple patients that I've known beforehand that they were involved or going to go to prison or jail or whatever. Um, and for those patients I would adjust treatment plan accordingly beforehand, before going in, because then we knew whatever treatment we were doing would not be available in going to the whatever system it was. Um, but most of the time it's me, like, it's strictly, like, somebody shows up on my schedule. I haven't seen them for six months. They come in and say, yeah, this happened, I'm back, and then we're just trying to, um, pick up pieces from there. So there's not a whole lot of plan that goes into it. It's just like kind of reacting to what is, what is in front of you, and trying to adjust and adapt from there.

P: Um, and then it's hard to know, I mean, it affects me because it's hard to know. It's kind of a black box. I have gotten records from prison healthcare system previously, but not easily. Not, like, definitely not promptly. It's always me asking and then receiving them sometime later. Um, it's not always exactly clear from those records what happened while the patient was there. So, yeah, I mean, I think it is a lot of just kind of picking up the pieces and, like I said, specifically substance use disorder. There doesn't seem to be a lot of treatment or management within the prison system, and I know that's changing, hopefully for the better, but, um, so, yeah, I mean, I think it's, I wish it was more planful and I wish it was, uh, kind of easier to have more fluidity in the care, but it seems like it's pretty disruptive and kind of, um, yeah, you kind of just do the best you can when you see a patient who has been incarcerated recently.

I: Mm-hmm (affirmative). And what substance or substances are you treating?

P: Yeah, I mean, usually the bulk of the patients that I see is with opiate use disorder. So, so mostly heroin use disorder, but occasionally pills as well, Percoset, Hydrocodone. Um, but alcohol use disorder, certainly treat a fair number of patients with that, and then there's several people with, um, poly-substance use disorder as well. So abusing usually some combination of alcohol, um, opiates and cocaine or methamphetamine, um, so kind of, I mean, a mixed bag. But predominately opiate use disorder currently is what we're, is what, I would say predominately in this clinic that's what we see, is a mix between opiate use disorder and poly-substance use disorder.

I: And then do you communicate with parole officers or probation officers or the courts at all?

P: No.

I: Okay, and then aside from possible justice system involvement, what else are your justice-involved patients dealing with socially? So in addition to, like, housing that you've already mentioned.

P: Yeah, um, I mean, I think housing and financial concerns are the biggest things. Um, we're, and, you know, who knows how many, for a patient to see us they have to either be self pay or have insurance, um, and so we, so the patients that I see have been able to enroll in some type of insurance generally. I mean, self pay is, again, generally speaking, broad sweeps, is that, like, you know, self pay is too expensive, quite frankly, to go to the doctor, so most people aren't able to do that for any prolonged period of time. So huge, by far the majority of our patients are on some type of insurance, so I don't even see patients who aren't able to access insurance after they get out of prison, so I wouldn't even know, you know. It's just outside my, um, outside of my wheelhouse, but I know that that is definitely a problem as well. But it's not something that I'm really exposed to daily on a clinical basis, anyway.

P: But, yeah, I think, yeah, financial problems in general are a huge, uh, are a huge concern. And I think, I mean, this often plays out with substance use disorder of any type, but I think probably generally is like, there's a social concern of you're being plucked out of an environment for whatever, either perceived or real breaking of the law, and then you're being placed right back in that environment with little or no rehabilitation. I think that, I see that play out in patients of, like, again, I say substance use disorder is probably the most present frame of, like, if you are clean from drugs for six months, you don't use any, any substance, and then you're put back into a situation with all your old friends that use drugs and everybody around you is doing it, it's really easy to relapse in that type of situation. I think you can say that for any number of other things as well. So, like, yeah, just being put back in those types of situations isn't helpful without some kind of, I don't know, some other types of supports.

I: And then, again, among your justice-involved patients, what are you seeing them dealing with medically?

P: Um, well, okay, substance use, mental health. Mental health concerns are, that's real broad, right? So, you know, ranging from anxiety and depression, which are, you know, probably the most common of mental health concerns, but PTSD is certainly, um, that would, a higher percentage with PTSD and then, I mean, some with more serious and persistent mental illness as well, things like bipolar disorder, some type of, um, psychotic disorder like schizophrenia or other things like that. So, um, those two are more prevalent.

P: Um, I mean, in general, I would say in general chronic disease, hypertension, hypertension and diabetes. I see more, which might be a side effect of chronic stress as well over the population. So, that was the question, right?

I: Yeah.

P: Which, which are you seeing more? For sure the, for sure, I mean, probably mental health on top and sometimes it's chicken and egg with substance use, which came first, right? A lot of dual diagnosis with that. Plus or, mental health plus or minus substance use disorder, and then from there just kind of a smattering of other chronic diseases, some of which I think you can really trace back to, um, stress. Um, yeah.

I: And then are there any resources or services that you are seeing that your patients need, but aren't available to them?

P: Um, it's always hard to know from my, from my perspective, how available they are, because I certainly hear about a lot of people both, I mean, both governmental agencies and non-profit agencies doing a lot of great work, like, for employment. You know, I know of several non-profits in the area that do reentry programs and really work with people to, um, yeah, work with them on getting gainful employment. So that's hard to know for me, is access to those programs limited? I don't know what the barriers are to things like that. But, yeah, like, I see, you know, people struggling with the things I said. You know, employment, housing, financials in general. Certainly, I think, access to healthcare. We talked about, like, I'm a little shielded from, but I think is a big part of it, and I don't know what the, what exactly the barriers are to a person engaging in those things. Um, and it's, it's, even when talking to a patient it's not always clear to me, like, why or why not they can't.

P: I certainly hear from patients about, you know, "Yeah, I tried this and they don't call me back or they, you know, I can't, they told me there was nothing they could do for me." You know, like, these are the types of things I hear and it's hard for me to dig into those.

I: Mm-hmm (affirmative).

P: Those interactions, and some people that are successful in engaging in that as well, you know, so it isn't like everybody has trouble, but it seems like there's a lot of people that have trouble really getting resources that they need.

I: And now thinking broadly, are there any changes to healthcare delivery that you would suggest to better meet the needs of folks that have a history of justice system involvement?

P: Yeah, yes. Um, I think, I think, um, I think anybody who's involved with the justice system, you know, in the months leading up to their release should have, um, a really intact plan for how that's going to look and what, so if you don't have healthcare, can you sign up for it? What do you need to go through? Get them signed up. Um, like, plans for either gainful employment or something along those lines we're working on. I mean, sometimes, I mean, even starting that process in prison of, you know, like, testing to see what type of, um, field or appointment they might be in or if they're really competitive for the workforce at all. Like, maybe they have either mental health or developmental disabilities that make them not really good candidate to stay employed. Um, and I think, yeah, so I just think, like, management like that prior to discharge, as well as, like, with healthcare issues specifically, you have, like, some kind of formal, uh, like, to use the hospital term, discharge summary that talks about the length of stay and what was done for their, um, their health while they were, um, while they were in the justice system.

P: And then having them identify someplace where they want to, like, you know, whether that's each or the state or the county forging relationships with community clinics that are going to be, take care of people compassionately or some other system, I don't know, but some kind of formal handoff system where that exists. That continuity of care is so valuable.

I: So thanks again for your time today. Before we wrap up, is there anything that I didn't ask you about that you think would be important to add?

P: Oh, no. This was, it was great. It was fun. Thank you.

I: All right.
