# Supplementary material for: Physicians’ perspectives on continuity of care for patients involved in the criminal justice system: A qualitative study
Source: PLoS One. 2021 Jul 14;16(7):e0254578. doi: 10.1371/journal.pone.0254578 (PMC8279398; doi:10.1371/journal.pone.0254578)
Supplement: S2 File — (ZIP) [file pone.0254578.s002.zip › Clean/Participant_2_LJ_deidentified.docx]

P: Am I close enough?

I: Yeah, I’m gonna position one close to you and one close to me. And take note of the time. . . Okay.

P: So, both of these are going-

I: They’re both going, yeah. So again, thank you for taking the time to meet with me today. I really appreciate it. Um just to give you a little bit of background about the study, this interview is part of a larger project between [Health System], us at the [County] and the [University] where we’re examining the relationship between Health and criminal justice involvement. Um and the goal of this interview is to gain an understanding of your perceptions of the criminal justice system um as well as the experiences you may have had treating patients with criminal justice system involvement. Um and so just to start us off today, I wanna get a general overview of what you know about the justice system, um could you tell me a bit about what you think about the current state of the criminal justice system here in the United States?

P: . . . [sigh] . . . uh i-it, there’s room for improvement . . . um and I know that there’s problems with uhh well, because I am a psychiatrist, uh. . . I am very aware of the uh criminalization of the mentally ill. So our largest psychiatric facilities are jails and prisons. So, that piece is very concerning and a-ah of course the whole piece about race and uh people of color being uh ins- you know uh, uh, placed in these uh prisons and jails at much higher rates than non uh you know white people. And I believe that uh the way things are going that there’s a, probably a effort to um privatize. . . these facilities and so then there’s a profit motive and uh. . . I have done consultation work uh outs- this is not a part of [County] uh with um [community organization] which is in ah uh uh specific, culturally specific treatment program for African Americans. And the uh CEO of that organization has worked 30 years with the department of corrections and in fact he was the first nonprofit to uh exclusively work with the DOC. Um in this treatment program and I was speaking to him today and he said that. . . he is gonna send me information from SAMHSA which is evidence based treatment for people with addictions who are part of the the criminal justice system and the Department of Corrections does not want anything to do with that program. And I said well does that have to do with money, he said no because it’s free. And then . . . you know he we talked a bit more and then the thought was that there could be a the reason this evidence based approach to uh helping people get set up six months before they’re discharged out of uh being incarcerated so they want to help set up jobs, and housing and all that and the reason this evidence based thing isn’t utilized he thinks is because there’s a financial incentive to keep people locked up. So….. that’s a long answer to your question.

I: Next I’d like to discuss some criminal justice system terminology

P: mhmm

I: Um could you explain to me what comes to mind when you hear the following terms. The first is prison.

P: What comes to mind uh a higher level of offense meaning if you’re in prison you had a higher uh more serious offense than if you were in jail.

I: Mhmm. So, what comes to mind when you hear the word jail and how do you distinguish between jail and prison?

P: Mmm umm well….prison meaning uh whatever Oak Park Heights, ummm Stillwater, St Cloud, those facilities, people who land in those facilities have levels of crime that um uh were felonies or worse levels of crimes. Many of my patients, mentally ill patients, who are vagrant or peeing, public indecency, whatever, they go to jail or they bring them to acute psychiatric services because they don’t know what to do with those people. So, the point I’m getting at is, you know, people generally who end up in prison or have been charged with a worse crime.

I: So, what comes to mind when you hear the term probation?

P: Well, uh it’s a way of monitoring people through drug testing contact with their probation officer uhhh monitoring them after they have done their time so to speak. Uh we work here in the addiction clinic with drug court, um, which I guess isn’t technically probation, but I work with a lot of people who have probation officers

I: And what comes to mind when you hear the word parole?

P: I, you know, I’m not entirely clear on the difference between probation and parole. I- I I- don’t know the technical difficult, difference between those two things.

I: And now I want to shift to your background and education in training. During medical school, did you ever receive any training whether that was informal or informal on working with justice involved populations?

P: No, other than I uh did some elective time with a forensic psychiatrist, but that was my choice.

I: Mm. Could you tell me more about that experience?

P: Um… and actually you know, I now, I guess, again both of my experiences interfacing the criminal justice and medical school were of my electives. So one elective was at the [city name] medical examiner’s office. And the other elective was with the forensic psychiatrist. Um with the forensic psychiatrist I went to court with him uh and listened to uh, he was involved with a particular court case, and so I talked to him and got a change to observe court. Uhm and then in the medical examiner’s office, I was there in the morgue, city morgue, and um had an opportunity to work with the staff there, forensic pathologists, so you, the people who are considered uh the uh medical examiners case are cases that involve either uh you know criminal act, homicide or accidental death you know hit, hit by uh car whatever uh so anyway, you know, it wasn’t like I was really involved with uh criminal justice but at least you could,…as an example, they would do uh what they call brain slicing once a week and there was uh uh forensic neuropathologist who would speak at that weekly conference and they were examining the brain of an infant and she, as she’s doing the brain slicing, you know, looking at the tissues said this child did not die of uhm SIDS. The parents apparently said they died of SIDS. She said, no, this it was very clear this kid was smothered. So anyway, that’s what little I, you know, was involved with.

I: Yeah. In addition to your electives is there anything you think could have been helpful to you in medical school in terms of training?

P: Well, you know, that’s an interesting question whether they uhm do community clinics, you know, homeless clinics, but whether they do a clinic in a, in a jail or prison you know it’s an interesting thought.

I: And so, do you think you would have liked to have that opportunity, would it have been something that would have been helpful to you at that time?

P: Mmmm, not necessarily because I uhh I wasn’t interested in uhh in working in the prison system. My father was a psychiatrist in the prison system, so I heard about it through his experience. Umm I deal with jail patients all the time in acute psychiatric services. I deal with people interfacing with the law, and on uh drug court and probation and addiction clinic. Um I do the consultation work at the program I told you about which is interfacing with the department of corrections. The point being I didn’t have to have training in medical school to do those things.

I: And so, thinking now about your residency, um was there any training that you received working with justice involved populations at that time?

P: (sigh) Not, not formally no. Mm.

I: Did you have any types of informal training during that time?

P: Yeah, I’m trying to think if we-I think uh I’m trying to remember if we had an opportunity uh to rotate with the jail psychiatrist cause we have psychiatrist here at [health system] that go to the jail. And I might have gone to the jail a couple of times with one of the psychiatrists. Um, it wasn’t like a formal, uh, whatever you wanna call it, legal uh criminal justice rotation. It wasn’t a formal thing like that.

I: Okay. And how about during a fellowship? Did you receive any formal or informal training at that point?

P: No. Uhmm I’m trying to remember if we went over cause I did my fellowship at [clinic name]. And I can’t remember if we went over to the federal medical center. Umm not fo-, not formally maybe a brief visit or interaction there with the staff. But I don’t think it was a formal rotation.

I: And so, thinking now about your current place of employment or any past places you’ve been employed have they done any types of educational trainings that have been helpful to you?

P: Trainings for?

I: For working with justice

P: criminal…

I: involved (inaudible)..

P: No I I think I’ve learned on the job.

I: Mhmm… And so now thinking about your day to day visits with patients.

P: Mhmm.

I: During those visits, do you ever ask patients about their justice system involvement?

P: Mhmm. Mhmm.

I: I see, and so how do you ask that question?

P: Have you ever been incarcerated? What were the charges? Are you on parole? Are you in drug court?

I: And is this a topic that you broach with all of your patients?

P: Ummmm. (long pause) Don’t do it in acute psychiatric services uhh. Usually I don’t do it there, um, but uhh in the addiction clinic, when I seek patients there, uh uh and I uh outpatient clinics addiction clinic and [primary care clinic for adults with frequent hospitalizations] uhh I… I…let’s just say 50 percent, 50 to 70 percent of the time because if I can see in the notes their history, then I might ask a question about it. Umm…so…

I: And once you have that information how does that form your care plan for that patient?

P: Ummm…. That’s a good question. If they’re in drug court, they’re getting urine testing for drug court, but I will still order testing for myself. If I want to see something and the addiction clinic, they have to conform to the rules there, and they have their schedule for urine testing. So, it doesn’t really change uh my care plan really it’s, it’s historical in- information or current information and um sometimes um they’ll have to sign a release if I have to talk to their probation officer or something. Um, but I don’t know if it really changes my practice.

I: Mhmm. Okay. So, what do you think some of the benefits are of asking whether or not your patients have any past or current involvement with the justice system might be?

P: I, It gives you better idea of trauma history. Um, it gives you better idea of, you know, their history like if somebody says, you know, I spent 20 years in the pen, you kind of have a better idea of what their life is like. Umm and then if they’re currently on parole, probation, drug court, that’s helpful to know what system they are, you know, having to deal with.

I: And are there any challenges you see in broaching this topic with any of your patients?

P: (long pause) Mm, I can’t think of any. I, I don’t know, when you say that challenges what comes to my mind is maybe non-psychiatrists or people who have different kinds of work than I do. Maybe it’s more scary for them or more whatever, but for me it’s sort of part in parcel of uh the patient population and the kind of care that, uh, you know, cause I’m interfacing like we have a LADC, licensed alcohol and drug counselor, at the clinic who umm is always going over the court and interfacing with drug court. So, these conversations are part of our conversation in the places I work. So, it’s just part of the routine.

I: Mhmm.

P: You know, there isn’t like ohh this deep dark secret or we shouldn’t ask or something. Uh there isn’t- the places I work, it isn’t, I don’t have that, you know, issue.

I: Could you tell me a little bit more of your overall patient population that you do see?

P: Mhmm. Umm I work with people who have mental illness and addiction. Um I work in acute psychiatric services so in that place uh people are brought in off the street or sent from the emergency room or sent from clinics or they self-report there for crisis or medication or what have you. So, some people can be acutely uh psychotic or uh intoxicated and dangerous umm withdrawing from something. Uhh all the way to someone that says, “hey I need a refill of Prozac.” So you kind of see a large spectrum. Uhh in the addiction clinic, uhh, the patients I see are on our methadone maintenance program. Uh so those are people that have uh opioid use disorder. Uhhh usually injecting heroin or snorting heroin and other substances. Ummm and so I

[Knock] [Interruption by nurse]

P: Hi come on in. Hi!

R: Hi. I just need to hear our [inaudible]. Okay see you next time.

P: Thanks [name].

R: You’re welcome.

P: Appreciate it.

R: You’re welcome.

P: Umm okay where did I leave off I’m just thinking umm

I: Yeah, you mentioned the methadone

P: Oh Yes! So that’s at the addiction clinic

I: Mhmm

P: And then in the [primary care clinic for adults with frequent hospitalizations] we are dealing with medically complex patients with addiction and psychiatric issues. So, these are people with serious medical illness and most of them have also addiction and psychiatric illness. So.

I: And what would you say the typical income levels of your patients are?

P: Zilch. Nothing. Low.

I: Mhmm

P: Whatever. You know, not much.

I: And what types of insurance would you say that most of your patients have?

P: Uh either medical assistance or no insurance

I: And what about your patient’s disability status?

P: Some are disabled, some are on social security disability

I: Mhmm

P: And I try to help people to get that [zippering]

I: And have you noticed any particular challenges or barriers to care faced by patients who are of racial or ethnic minority backgrounds?

P: When you say have I noticed that meaning uhhh in my work here at [hospital] have I noticed it, or do you mean just as a general way or?

I: In your work here at [hospital].

P: Have I noticed barriers for people of color? Not really, no. Do I know they’re there? I do.

I: Mhmm

P: But I haven’t, I mean, we take all comers in acute psychiatric services, addiction clinic, and [primary care clinic for adults with frequent hospitalizations]. Uhh my job here, uh, I deal with very ill people and they’re of all shapes forms and colors. Umm so…that being said I’m not denying inherent bias or what have you. I’m well aware that uh as an example people of color don’t get pain meds when they come into emergency room like white people. Umm but (long pause) this is [hospital]. Ohh! Were supposed to say [health system].

I: [Laughs] Yeah

P: Yeah

I: Okay. Um So for your patients that do have some type of justice system involvement could you speak a little bit to what that experience is like for you as a provider? I know you’ve mentioned that you sometimes ummm communicate with drug court and probation officers could you speak a little bit to that?

P: Umm, what is my experience?

I: Mhmm

P: Uhhh its hard, uhh the reason I’m hesitating it’s like uh, are you looking for a qualifier? Like is it good bad and different? I mean, I mean it is what it is. I I don’t know like are you wondering if I’m frustrated or do I think its lousy or I don’t know what you’re…

I: I’d be interested in hearing if you do if you think it’s good or bad or different

P: I’m kind of like neutral, it is what it is. You know I I can’t think of anything I mean I have to deal with uh mothers and CPS, uh Child Protective Services, um and uh, you know, I’m, I hear good things from uh what’s his name uh [name] who’s with, uh does the stuff with the drug court uh. So, um I what I do get frustrated with, this is what I get frustrated with, is in acute psychiatric services, there’s a ring around the rosy, uh, push the, you know, um. I’m trying to think of the right terminology uh (sighs, clears throat), uh, pass the buck. Okay so what happens. Someone that’s mentally ill does something. The cops pick him up. They take him to jail. The jail says well we can't do anything about them, we’re gonna take him to, drop him off at acute psychiatric services. So, the merry-go-round can be that the interface of mental illness versus legal and who’s gonna take responsibility for that. And that’s very frustrating cause we have a lot of people who, you know, they it just isn’t a very good system of cops picking people up, and saying, taking them to jail, and the jails says well we can’t do anything about this person. A guy in our waiting room at acute psychiatric services pulled out a big pipe and was threatening everybody yelling and screaming and, uh, you know, the people involved called the cops. He was charged with whatever, terroristic threats, or whatever. He went to jail, and jail just sent him right back to acute psychiatric services and said he needs to be there. But then the inpatient psych unit doesn’t want this person. So anyway, that’s, uh, I, because you haven’t worked in this field you may have to be careful about explaining this cause it sounds like oh inpatient doesn’t want a guy who’s obviously psychotic and waves poles around. And it’s not that, it’s that we have a system where that interface of people who are doing crazy stuff, illegal stuff and the jail says we can’t deal with them because they’re crazy and then we get them, but we don’t have adequate places for people that are dangerous and repeat offenders and so on and so forth.

I: Thanks for that explanation.

P: Yeah

I: Could you tell me a little bit more about what information is communicated between you and the courts and probation officers?

P: Oh, they wanna know like if, is the person taking their meds? Are they going to appointments? Um, you know, are they following through, uh, with their care. I don’t interface with them so much. It's more the addiction counselors that are interfacing with those probation officers. But on occasion I will, you know, be asked a question um so.

I: And so aside from just obviously the justice system involvement aspect, what other social factors are going on in your patients’ lives?

P: You mean the social determinants of health?

I: Mhmm.

P: Well you name it, they got it. Right, the historical trauma, current trauma, homelessness malnutrition, uh, you know, living under threat of violence, uh, no money, poor health, exposure, using abusing substances, smoking cigarettes, uh, being a person of color. I mean name ‘em all.

I: And what are some the more medical issues that you’re seeing among your justice involved patients in terms of physical health?

P: Well I don’t deal with physical health. I’m a psychiatrist

I: Mhmm

P: So, um. I mean, the thing to look at, and I’m sure you’re aware of ACES, right? Okay.

I: [overlapping speech] Adverse Childhood Experiences

P: So, you look at that and then you see what happens ten, twenty, thirty years down the line. They die sooner. They got more cardiovascular problems, diabetes, blah blah blah. They got more mental health problems, more addition problems. So, you know, the people we see in [primary care clinic for adults with frequent hospitalizations] are the end result of those ACES. They’ve had a lifetime that started there, a lifetime and then we try to, you know, help them as they basically they’re kind of, you know, I say end of life meaning they’re not all that’s not technically hospice, but we help a lot of people who um end up dying because of those health problems that started, the underpinning that started many, many years ago.

I: And so, this is probably more in your sphere, so what are some of the mental health conditions that you’re seeing among your justice involved patients?

P: Trauma.

I: Mhmm

P: Trauma, post-traumatic stress disorder. Uh depression, anxiety. And there are some with psychosis um there’s also personality issues, antisocial personality, uh borderline personality, um narcissistic personality issues. Um that’s not everybody but, you know, those are some of the issues.

I: And in terms of addiction, what are some of the substances that you’re seeing in your practice?

P: Methamphetamine, opioids, marijuana, alcohol, cigarettes, cocaine, stimulants, you know, cocaine, separated out, cocaine, methamphetamine. Those are prolly the major ones. IV, IV, uh tes- IV, uh, steroid abuse.

I: And are there any resources or services that you wish that you had at your disposal to refer your patients to that you don’t have?

P: Um I’ve worked in that addiction clinic for over 10 years uh we've never had a social worker. Love to have a social worker. Would love to have better integration of mental health uh and addiction. Um meaning groups and uh we do have some groups limited, but it would be nice. I'm constantly having to bridge between because as a psychiatrist I’m embedded in the medicine department because I’m addiction psychiatrist. And I’m constantly, we have these silos here, and I’m constantly trying to go between the addiction world and interface with the mental health world and make referrals and, you know, it’d be nice if we could be more integrated.

I: And so, in addition to those suggestions that you just made, are there any other changes to how we provide healthcare that you think would be beneficial to patients who have criminal justice system involvement?

P: Well, I just gave you an example of what [name] told me. You know, he he’s recommending uh SAMHSA evide- you know what SAMHSA is, right?

I: Could you-

P: Substance Abuse and Mental Health Services administration, so that sort of, you know, if you type that into Google, you’ll get um a uh (clears throat), you know, get to their website and you can order all sorts of free, in fact, you probably can order free they call it TIPs, T-I-P, but there’s all sorts of free, ‘cause it’s your tax dollars um paying for this, um books and stuff that’s free and I think they do have a thing in there for dealing with criminal justice or department of corrections, whatever. I think one of their uh things uh whatever books are uh they call ‘em TIPs but they’re like little pamphlets um anyway. Uh (clears throat) uh I I’m sorry I’m getting over this illness, so my brain isn’t working so good

I: [laughs]

P: But the um, you said, what would be better, what would help people in delivery of healthcare that have interface with the criminal justice system or whatever is that what you said?

I: Yes

P: I mean, I just told you something! I just told you what uh this, like the most established uh treatment program, at least according to [name, he’s been doing it for thirty years with the department of corrections and he made a recommendation for a particular uh evidence-based treatment and the department of corrections said no. So, I can tell you everything that I think should happen, but it doesn’t, thus far, I haven’t seen it happening.

I: Mhmm

P: People before they get discharged from jail and prison should have uh somebody there to help set em up. You know, place to live, vocation all this. (P starts talking faster) Instead what we get is people coming into acute psychiatric services saying hi they, they I I just got here by bus I got out of the prison in [city name] or wherever, I need my meds. I mean that happens all the time. People are discharged with one or two weeks of meds and then its bye. So, you know lack of integration. Lack of attention to how to do a wraparound thing, you know? Can you tell where I stand?

I: [Laughs] I can. Um so I’m getting to the end of my questions today before we officially wrap up, I Just wanted to ask, is there anything that I didn’t cover today that you think is important to add on this topic?

P: Um I think I’ve said my peace, and I appreciate you listening, and you ask, you have a very nice demeanor and um you ask your questions well. I understand that you are focused on medical providers in these three areas. I just want you to know that uh if you had any interest or wanted to speak to this colleague who has the culturally specific treatment program that exclusively works with department of corrections, I can put you in touch with him.

I: Okay. That would be great. (long pause) So, thank you again for taking the time to sit down with me today um like I said we’re gonna be interviewing approximately thirty more physicians from here um [clinic name] and [clinic name]. Once we have reports related to this project would you be interested in receiving some of those?

P: Oh sure, yeah

I: Okay. Alright. So, I can. We’ll turn these off, and then.

[END OF INTERVIEW 34:50]
