# Supplementary material for: Physicians’ perspectives on continuity of care for patients involved in the criminal justice system: A qualitative study
Source: PLoS One. 2021 Jul 14;16(7):e0254578. doi: 10.1371/journal.pone.0254578 (PMC8279398; doi:10.1371/journal.pone.0254578)
Supplement: S2 File — (ZIP) [file pone.0254578.s002.zip › Clean/Participant_3_Audio1_LJ_deidentified.docx]

I: All right. Um, so like I've mentioned-

P: Mm-hmm (affirmative).

I: This is part of a joint partnership between us, [health system], and the county and the [University] to understand um, what do providers know about the criminal justice system-

P: Mm-hmm (affirmative).

I: And how it might impact their patients. Um, I want to begin today by getting a general overview about what you know about the criminal justice system.

P: Sure.

I: Um, could you tell me what you think about the current state of criminal justice practices in the US?

P: Um, well I, I'll just preface that prior to probably about three to four years ago, I didn't know a lot. Um, but over the last several years, I've read and experienced I guess quite a bit. Um, I would say that uh, currently, from my perspective, I feel like the criminal justice system um, really is one more of punishment, um, and as opposed to one of rehabilitation or restorative justice. Um, uh, people commit what are labeled crimes, um, and there's sort of, once people get trapped in the system, it's really hard for, to kind of make a break and get chances.

P: Um, and I feel like it's a system that once you're in it, um, kind of keeps, not only keeping people uh, incarcerated, which happens, but also keep surveillance on people through things like probation and parole, and then also through collateral consequences of um, limitations and voting rights, and um, the access to food benefits and things like that. Um, sort of disenfranchises people for many years, um, even lifetime after they have done something wrong. So that's sort of my take. And then also I would say, I, I do think the amount of mental illness and addiction that is seen in the folks who end up in the system, um, to me speaks to sort of bigger issues that aren't being addressed, um, both by the criminal justice system and sort of outside that system as well.

I: Okay. And next I'd like to discuss some criminal justice terminology.

P: Sure.

I: Um, could you explain to me what comes to mind when you hear the term prison.

P: Sure. So, um, I think of a, it depends which one. I went to Shakopee actually, which is the women's prison not that long ago. And I actually thought that looked like a college. But in general, when I think of prison, I think of sort of uh, um, uh older stark building with cells where there's people sort of locked up behind bars, um, a lot of the time, um, while there are areas where people can work and, um, sort of eat and things like that. In general, it feels like a place where people are just sat and sort of put to spend time their time as opposed to a place that really helps give people skills and gets them prepared for a different kind of life than they had prior to being there.

I: And what comes to mind when you hear the term jail?

P: Um, so in general, I think of the jail where I work, I would say where, um, it's folks that have come and our pretrial, uh, they have been accused of crimes and, uh, are sort of going through criminal justice proceedings. Uh, there's a lot of folks who are kind of in and out a lot just based on socioeconomic issues or their own interface with police and, and where they happen to live and things like that. Um, and um, I think in general actually with both of them, um, it's something that I think, uh, the reality and sort of the popular media and sort of Hollywood defied perception of are, are quite different. And so that's something that's sort of, I think about a lot actually.

I: And could you tell me what you know about the similarities and differences between prison and jail?

P: Sure. Um, so in general, jails, um, are pretrial or pre judications so people are, um, it's general where people are held while they're awaiting trial. Um, uh, they, uh, there are some that uh, actually in many places in the country that are both pre and post sentencing. But um in [County] for example, they're separated, um, jails tend to be, um, more locally funded, so we usually on a county parish or whatever, smaller entity, city level, um, and uh, tend to get their budgets and resources in that level as well. Um, and then prisons tend to be, um, can occur also on different levels, but usually more of a state or federal level. People are sentenced from there. It's after sentencing and they typically over a year of time, so people go to prison for any where from a year plus a day to a lifetime depending, and there's various security levels based on the individual and sort of what, what their charges are, what their sentences and kind of what their behavior has been historically too.

I: Yeah.

P: It's probably just one(laughs)

I: And so what comes to mind when you hear the term probation?

P: Um, so probation to me sort of, um, it's kind of this trial period so people will go to prison um or they'll be pre or they’ll be in jail actually and um, kind of get this time where they're not going to be in custody. So no one’s going to be looking after them, but they have signed a fairly strict stipulations of things they have to accomplish during that time to be able to sort of stay out of, uh, a more locked facility, uh, basically. Um, and while a, theoretically I think it sounds good, um, my biases sort of become that it's many of the stipulations that are placed during that time are things that are pretty difficult to actually accomplish, uh, for many of the people end up in those situations.

P: And so it becomes a way to kind of keep a watch on people and then eventually get them back into custody to um, things like, people who struggle with addiction, getting drug tested all the time, people keeping housing, getting jobs with amidst a society that sort of continues to keep people out of jobs who had criminal histories and things like that. So yeah. Probation.

I: And then what comes to mind when you hear the term parole?

P: Um, so parole, I know a little less about it honestly. In Minnesota, I don't believe there's parole, but basically they, um, parole is similar to probation in a way, um, but it's um, tends to be after prison so people have served time, then they're on parole, they're out in the community, they're on parole for X amount of time before they're ultimately sort of free of the system. My understanding.

I: And so now I want to turn the transition to your background in education and training.

P: Sure.

I: Um, during medical school did you receive any training whether it was formal or informal, on working with people who have justice system involvement?

P: Sure. Um, I'll see a little bit, so I do remember in medical school, um, I don't remember lectures specifically, um, or like small groups discussing this kind of thing like we did with many other topics, but um, I do remember going to a local prison, um, and seeing what that looked like. Um, and talking with one of the physicians who worked there. But beyond that, I don't specifically recall any education about justice involvement-

I: Um-

P: ...other then I guess, sorry to cut you off, other than like people in jail are more likely to get TB, like that kind of stuff (laughs) like sort of very fact based, practical, clinical things.

I: And was your, um, experience with the trip to the local prison ... Was that something that all medical students did?

P: I don't remember (laughs) honestly. Um, I'm trying to remember why we did that. I don't think so, but I, I, I honestly don't remember.

I: Mm-hmm (affirmative). Okay. Do you think that there's any additional training or education that could have been provided to you during that time that would've been helpful?

P: Yeah, I definitely think so. So, um, in, in retrospect, um, I think there's a lot of things I now have perspective on in terms of just people not coming to appointments or things people are at risk for or I guess for example, um, that if I had sort of been trained about that I might have asked my patients different questions or just sort of had a different lens with which to look at the experiences they were going through.

I: And how about during your residency? Did you receive any training during that time period?

P: Um, not formally, um, I went to residency in Louisiana, so it was without question that we saw a lot of people who were incarcerated. Um, but there was no formal training specifically about it.

I: Okay. Could you tell me more about your informal training and what that looked like?

P: Sure. Um, I mean, mostly it was in the context of patient care. So there in the emergency department at [hospital]. There was a room where they would have people, much like they do here actually, who are getting sort of cleared for to be able to go to [prison]. Um, and so they, um, they would just hold them there basically, and then you would evaluate them medically. I remember, like treating someone who had been tased and sort of thinking about like, first of all, what does that even look like sort of medically, what does that mean? Um, uh, I remember evaluating someone who had chest pain and sort of who had been effectively malingering, sort of saying he had chest pain so he didn't go straight there.

P: Um, sort of thinking about what that meant. Um, and then, um, yeah, I think in my rotation there was like a few weeks. So those are a couple that stood out to me now in retrospect. And then certainly on the medicine wards, we treated people from the prison system and the jail system as well. Um, I distinctly remember, um, a case in the ICU I had where I had a patient who was at [prison], which is sort of a pretty famous prison known for sort of people who serve lifetime sentences in Louisiana, um, and they have hospice, they do all kinds of things.

P: But um, I remember sort of when he died having to figure out how, who to call to get his body released to his family and sort of the implication of the fact that they sort of, it felt like they had a hold on him even in his death and um, thinking about sort of what does that really mean on an ethical level of even a spiritual level, just those kinds of things. Um, so to me that was a piece of it. Um, there was less formal training at that point, um, around sort of transitions out of custody or communication with custody and things like that. Um, that piece I didn't know as much about-

I: Mm-hmm (affirmative).

P: Didn’t learn about.

I: And then did you receive any training during a fellowship?

P: I didn’t complete a fellowship.

I: Okay. And (laughs) so how about here at your current place or maybe a past place of employment? Did you receive any training then?

P: Sure. Um, no, I would say not. Um, I mean I currently am the [job position], so I, most of my training has been informal and then sort of um, self in like kind of interest, uh, personal interest I would say in the system as sort of a social justice issue kind of based on side things I do outside of work.

I: Mm-hmm (affirmative).

P: Um, but nothing that was sort of formally taught to me with the exception of like I just did a grand rounds like a couple years ago. But besides that, no.

I: Okay. And so thinking about your day to day visits with patients, um, do you ever ask about their current or past involvement with the criminal justice system?

P: Sure. Um, certainly the jails always. In my primary care clinics. Um, I would say I don't always, um, I have started to feel more comfortable doing it, um, and uh, have, have come up with ways that, that makes sense. Um, I have certain patients I've actually seen in clinic and in the jail. So for those patients it's easier to talk about it. Um, but for others it also has come up, um, folks who haven't been around for awhile. Sometimes I will ask if that's the situation, um, I tend not to do it. Uh, I see a lot of immigrants and refugees. I tend not to do it in that population, um, in light of political climate, but with other folks they do or I've started to more.

I: And could you give some background and how you broach that topic-

P: Mm-hmm (affirmative).

I: ....with your patients and how you ask?

P: Sure. Um, I guess when I have asked to try to do it in a way that is normalizing it, just acknowledging that it has a big impact on people's health and their ability to, to access healthcare. Um, again, I, I haven't probably made it a much a part of my standard practice as I could. Um, but I think that's, that's the way with sort of any more touchy subject. I've tried to breach it, approach it.

I: And then how do you use that information to inform your care-

P: Sure.

I: ... patient?

P: Um, I think just in terms of like, um, it helps with understanding health risk factors honestly, um, in terms of like infectious diseases, but then also, um, like mental health or addiction disorders that may, that just might have a higher alert for those kinds of things. Um, I think in terms of the acknowledging challenges with social support, um, if someone's coming out of prison for example, and getting back on their feet in terms of getting a job and having community and things like that, just having more awareness around talking to the social worker, getting them connected there.

P: Um, those kinds of things. Um, also just in terms of like actual care, um, acknowledging that there probably are gaps in records and screenings for example, and things that are maybe more likely to sort of a approach more quickly or just try to make sure we get on top of, um, from that perspective too. Yeah.

I: And so what do you think are the benefits of asking your patients about whether they've been involved with the justice system?

P: Sure. Um, I mean, I think some of the benefits are just, um, acknowledging it as a social risk factor, um, much like homelessness or an addiction use disorder or whether they have a family. Um, it does come into play, um, sometimes. Um, I think it, it also can if done in the right way, um, sort of help the patient understand that you have an understanding of at least some understanding of that system and how it may have impacted them. Um, yeah. I would say those are the things.

P: And I guess I should add too, I always preface it. I mean even at the jail I preface it with like, I don't care what people do, like I don't want to know what people have done, um, because I think that that piece is not relevant at all. But I think just knowing what connection they have is, is, is reasonable, um, in, in most contexts, it, as long as the patient feels comfortable sharing.

I: Mm-hmm (affirmative). Are there any risks or challenges that you've seen to asking patients about this?

P: Sure. I mean, I think, um, one risk is certainly, um, while it can help to establish report can also be very off putting, um, uh, patients and just nobody I think should be judged by the worst thing they've ever done. And, um, certainly, um, opening up the conversation or even, you know, after a few visits talking about it could, um, could jeopardize like rapport and things like that. Um, I think as physicians and I’ll speak personally, but I think this is somewhat generalizable that, um, we'd like to know what we're going to do with the information. So, um, in, I think a lot of, a lot of the subjects that don't get asked about, it's because people don't know what to do with the answer.

P: So in the global health stuff that I do, if you're asking me about all these crazy places, people have been not even crazy, just they're from Africa for them. Um, but you don't know what that really implies. Um, it makes it, I think people are less likely to, to ask. And so I think that's one of the challenges too, is, um, in creating more generalizable sort of everyone asking about it, is having people feel comfortable knowing what to do with the information or wrestling with just the idea that knowing is enough. And that's maybe where it stops.

I: So I'm going to shift a little bit to wanting to learn more about your patients that you see-

P: Sure.

I: ...on a day to day basis. Could you tell me a bit about your overall patient population?

P: Sure. So I have a very mixed practice. Um, I see about, um, work kind of a quarter of my time in a sort of a general internal medicine clinic and work a quarter of my time in, um, uh, what's more called like a high utilizer clinic or hotspotter clinic, patients with complex psychosocial needs as well as complex medical problems, tend to utilize a lot of healthcare. Um, and then I also dabble in tribal clinic or admittedly incarceration doesn't come up (laughs) very often. And then the, I actually worked at the jail as well.

P: So I’m kind of working all those settings. And so depending on the setting, it's a little different, I would say in the jail. Um, I mean, obviously it's the jail. So, um, I think just being willing to learn about what people are actually going through. And, um, I don't, I, I really make it a point that like, I'm not a lawyer. People will sometimes talk about their cases and things and I, you know, I'm the doctor, but I think just like being open to understanding what people are going through and sort of understanding a little bit of the process of court and the legal hoops that they're jumping through, um, is, is, is good. Um, and then it also impacts like how we care for patients in terms of follow up and where they're going next and those kinds of things. So that's something.

P: And then I think my primary care practices, um, it, I think it impacts a little bit just like understanding, um, you know, if I see that somebody is in and out of the jail a lot, um, maybe acknowledging that there's likely some psychosocial mental health addiction related issues that probably need to be better addressed, um, along with unfortunately more systemic things like systemic racism and bias and things like that, which are a little bit outside of my personal wheelhouse. But just acknowledging, sort of being a witness to how those are impacting those individuals as well-

I: Mm-hmm (affirmative).

P: ...uh, and trying in small ways in personal and professional ways to be an advocate for changes to.

I: And so you mentioned, um, systemic racism.

P: Mm-hmm (affirmative).

I: Could you speak a little bit about, and maybe provide an example of where you've seen this come up in your practice?

P: Sure. I mean, I would say in general, the jail, um, I see a very small fraction of the people in the jail just preface it with that. 700 to 800 people at any given time and I see 10 to 15 a week. Um, but I would say just anecdotally, uh, disproportionately, I see men of color, um, African American, native native, some combination thereof, Latino. And so, um, to me that just as a sort of speaks to a bigger system of policing and sort of surveillance on particular communities, um, when data supports that there is no greater crime of a no greater incidents of crime in each community.

P: Um, and so there's something going on there that's sort of keeping those particular groups sort of in that, in that place. Um, and that has to do with history and laws and bias of groups that are meant to surveil people, um, and sort of keep peace, things like that too, amongst other institutions that also are biased (laughs) and then sort of perpetuated in other ways.

I: Mm-hmm (affirmative). And so, thinking again about your overall patient population-

P: Mm-hmm (affirmative).

I: ...how would you describe the income levels of the patients you see?

P: Sure. Um, may I see in general, um, I see patients who are um, uh, kind of living at or below the poverty line and then certainly there are some who work full time and um, you know, are more middle class, um, as well. But I'd say in general, um, I see a lot of people who use public benefits whether it's general assistance or who are on disability or things like that. So there's that. So there's those folks which is a little different I suppose than people who are maybe like scraping by with the jobs on the side and things like that as well. But, um, most of the people I would say tend to be poor.

I: Mm-hmm (affirmative). And then if you had to estimate how much their insurance status is-

P: Sure.

I: ...and, um, different insurers and patients might have, how would you imagine that breakdown would be?

P: Sure. So it depends a little bit on the setting, but I would say like in general across sort of my primary care practice, um, I mean it's at least 50% medicaid. It's probably higher than that. I mean it to be as high as 60% or a quarter or 75% rather. Um, there's a smaller fraction that's Medicare. Um, so maybe 25 to 30%. Um, and then, um, I do see a lot of Spanish speaking patients and so I see a fair number of people on either emergency medical assistance or, um, [county] charity care program as well. Um, but for the most part, um, I do see patients who are on public type of insurances with Medicaid or Medicare. I would say I have a very small portion that have private insurance through an employer, for example or through a spouse.

I: And then because you speak to the disability status, of some of your patients?

P: Sure. Um, certainly, um, I take care of a fair number of people who are either currently on disability or applying and certainly many more who are applying for disability, um, because if disability process seems to take so long (laughs). I have many more that are sort of in the process of appealing and things like that. But, um, I would say maybe 20 to 25% certainly are on disability and then a decent portion more that are sort of in that process or considering that process.

I: And so for patients that have justice system involvement in particular.

P: Mm-hmm (affirmative).

I: What was, what has that experience been like you, been like for you as a provider?

P: Sure. Um, again, I think for me personally, it depends a little bit on the context. So in the jail in particular, I, um, it gives me pause a lot, um, because of, um, I think that the dynamic between, uh, a medical provider, particularly a physician and the patient, there are power dynamics that they're coming to us. There's sort of this expectation I think in some ways that you do, you know, I give you instructions or direct directives or orders and they're done. Um, so I think that that's there. I think at the jail that's magnified just in terms of my power and privilege in that context versus theirs, um, not just being sort of disenfranchised by virtue of maybe race and class, but then also being incarcerated.

P: Um, so I do think about that personally a lot. Um, and then I, I do think I actually practice in actual practice of medicine is slightly different in terms of um, the context it jail actually some ways allows for different practice around things like opiate prescribing or, um, certain medications that otherwise I would grapple with a little bit more in the community, um, just because of standards I would sort of say, um, aren't as big of an issue. And so then it becomes sort of a different conversation. Um, a lot more sort of just saying no to people, um, which is challenging, especially when again, folks who are disenfranchised and just, it's a different role.

P: Um, so there's that piece and then I would say with primary care patients, um, uh, it's, it becomes more about just figuring out whether this is an ongoing impact versus sort of a past impact. So if someone's out of prison on probation, sort of, are they still kind of in the system, are there, what implications does that have versus someone who maybe had a bunch of misdemeanors because they were using drugs and then now they're clean and sort of moved past that, and it's been 10, 15 years and sort of how do you keep supporting them in that piece of their life as well.

I: And, how do you think justice system involvement may have impacted your patients access to healthcare?

P: Sure. Um, I mean, I think it certainly does in a few ways. I mean, I think the insurance pieces part of it, the fact that MA gets turned off and people are getting out of custody without necessarily having insurance, um, definitely treat plenty of people at the jail that they kind of come back and they're like, yeah, I’ve been off my meds six months because I just didn't get around to getting insurance back. Um, so that's certainly happens in the jail and um, I know it happens sort of, um, at [hospital] too. Um, so I see that piece for sure.

P: And then I think in ways, um, I personally don't know how to quantify well and probably as a system we don't necessarily quantify all that well in terms of, um, the implications of court dates and like PO visits and sort of these obligations related to, um, cases, um, and sort of the impact that that can have on ability to follow up with treatment plans and make appointments and things like that. Um, then I'll also say too, like just at the jail, I feel like there's, um, the, there are lots of cases that sort of get to me admittedly, um, but some of the ones that get to me the most are people who are like actively getting chemotherapy for cancer and then sort of end up locked up and have to have their therapy disrupted basically because of something that they supposedly did or whatnot.

P: Um, and in those cases just gets me because it's in the end, I'm not sure them being involved with the criminal justice system at that moment is really like, is, is that relevant to sort of what else is going onto their life nor the impact that whatever they did is really harming society all that much depending on how debilitated that an individual really, truly is.

I: And do you ever communicate with, for instance, the courts or probation officers or anything like that?

P: Yeah. So, um, I, um, I don't very commonly talk to like judges, I have um, there was like one case I particularly remember, and that was just because of um, determining whether he, like the patient was in the hospital, whether they can actually present a court or not. Um, I, um, most of the, at the jail itself, most of the liaisoning between like me as a medical provider, even as the [job position] with court related stuff happens kind of through either, um, our nurse manager or um, sort of, uh, someone who's like a psych advocate. Basically is his role.

P: And so they will sometimes come to me about, you know, making sure we see a patient or concerns that the lawyer has or something like that. Um, so that sometimes happens on the, um, on the, on the outside in clinic I have, um, I had a patient that was in mental health court. I did communicate with her PO in mental health court pretty regularly for awhile. Um, and then we have had a couple patients in the [primary care clinic for patients with frequent hospitalizations] that were in homeless court. Um, and both of these are sort of alternative courts that are ideally diversionary. Um trying to address people whose mental health is really the, what impacted their crime and or homelessness impacting like what charges they were getting and ultimately trying to find a solution that's not incarceration.

P: Um, and so those couple of patients who were in homeless court, uh, we have actually met with their, like public defender to talk about sort of from a medical side like this is what's going on, this is what we think is probably the best plan. And then also talking to them about sort of can that be arranged. And kind of trying to work together um, to find a solution that didn't involve just like incarceration for someone that probably wasn't the right longer term solution anyway.

I: And then, aside from possible justice system involvement-

P: Mm-hmm (affirmative).

I: ....what else are you seeing your justice involved patients dealing with socially?

P: Sure. Um, I mean, housing instability is certainly one thing, whether it's related to prior history of renting versus having a felony offense that prohibits certain living in certain kinds of buildings, um, generally for sex offenders that's a challenge. There's not a lot of housing available for people who’ve committed sex offenses.

P: Um, I'm just making sure that's not a page. (laughs) (P checks phone)

P: And then, um, in terms of, um, other things related to, um, you know, keeping a stable job just with commitments to court or probation can be challenging.

P: And then just being hired in general can be hard partly because of barriers. Like, um, I know there are companies that have banned the box and things like that, but, um, there's also just the having actually the right skill set to have a job and keep a job and um, and that I think can be a challenge. Um, uh, ongoing issues with, um, you know, sometimes the communities that people came from that led them to a point where there was a crime committed, um, if they're going back into that environment and just struggling with those relationships and navigating those and sort of the same stresses that were there before didn't necessarily go away and sort of re acclimating to that chaos, um, can be also a big challenge.

P: Um, yeah, I'd say those were a lot of it. And then just, I mean, frankly, a lot of, a lot of folks have like kids or partners or our parent, I mean people that love and care about them that have, they've been distant from for X number of years also and sort of reconnecting that way and renavigating and negotiating those relationships. I think is really challenging also, um, for many people.

I: And again, thinking about your justice involved patients specifically, what are you seeing them dealing with medically?

P: Sure. Um, everything. Um, what I see particularly, um, you know, mental illness of various degrees, whether it's psychotic disorders like schizophrenia, but also insight, certainly anxiety, depression, post traumatic stress disorder. Um, and then I would say addiction disorders are also sort of the other big one. Um, particularly, I'd say alcohol, we're seeing a lot of opiates, meth use, cocaine, um, there's sort of, all of those I think are pretty prevalent right now. Um, all that seems to happen. And then I would say also, um, just chronic diseases that are suboptimally managed, whether it's hypertension, diabetes, um, heart failure, um, those are common as well.

P: Um, and then there is a subset who I would see at the jail, but not in the community who have like HIV, um, who, which I think you're just disproportionate as well. Um, then I also, I think we do see, um, uh, compared to probably the general population, a higher number of people identifying as trans or gender non binary, um, and that while I don't consider it a medical condition per se, is medicalized in the sense of needing and wanting hormone replacement therapy. And so, um, that can become a, that too. I feel like we're seeing more of. Those are the big ones.

P: And I guess a lot of traumatic injury. I guess that would be other things. We see a lot of like fractures related to violence, whether it's like punching, kicking, that kind of thing of fights. Um, and then also, um, actually like police related violence as well associated with arrest. So if we're talking about like the actual jail, so people getting bit up by police dogs, people with shoulder injuries from getting cuffed, those kinds of things and shot by the police.

I: Are there any resources or services that you wish that were available to you to refer your patients to you but just aren't?

P: Sure. Um, I do think there are a lot of resources I think, uh, but I, I definitely think there could be more. So I think one thing, I wish we had a better system of both in jail and I think out of jail would be, um, related to addiction. Um, I think the solution a lot of time just becomes treatment. Um, we send people to treatment, we did it, we checked the box and there whether they're sort of fixed or not, um, that sort of becomes an answer.

P: Um, and I think while treatment certainly does help people in some ways, um, uh, that in of itself doesn't necessarily, uh, fix everything else about what leads people to do what they do. Um, so I think that part is tricky. Um, I would say in terms of social services, um, I mean I wish we had like more dedicated case management for maybe not every single person but virtually every person, um, in a way to just help get them connected and sort of help them get through the system, um, in various ways. Um, I mean those two to me are really big. (long pause) I’m trying to think if there’s anything else. Um, those are probably the biggest groups.

I: So thinking broadly, in addition to what you've already mentioned, are there any changes to health care delivery that you would suggest to better meet the needs of patients who are dealing with some type of justice system involvement?

P: Sure. Um, I think a problem with healthcare in general in many ways is it the system is sort of set up, um, for many groups to fail. Um, and what I mean by that is it's in general, pretty rigid around you come to us at this time, if you're not here by this time, we're not going to see you and then if you don't come this time and we can't see you this time, then you can't be seen for X amount of time and you know, maybe the day you actually can come is a day you have to go to court or meet with your PO or something where you can't actually come to your appointment.

P: And so more flexibility around just like accessing providers. Um, and I, I'm not a huge proponent of like just having like Amazon takeover for accessing docs because I think there's something very valuable about the human interaction, but I think just more flexibility, whether it's walk in type system or um, sort of, uh, uh, just an acknowledgement of the challenges people face to actually access care. Um, and then also, um, I think just with like insurance sort of figuring out ways to better get people reconnected into insurance, um, having the state or the county for example, say we're just not going to turn people off for, just suspend people instead of canceling their insurance or just finding ways that would make continuing to access more accessible for people.

P: Um, and then with medications too, I mean I think medications in general, are way more expensive than they need to be, um, medic. Certain Medicaid products have copays, which I think is silly. Like just little things like that to just lower barriers for people to get the medications they need. Um, particularly when many of the folks that we're talking about are in groups that have their health, where there are health care disparities to begin with and then you add these other factors to it. It just broadens them.

I: So thank you for your time today. Before we officially wrap up, is there anything that I didn't cover today that you'd like to add?

P: Um, I'll just say this because I can I guess. Um, I mean one thing to me as I've learned about social justice or the criminal justice system and when I’m thinking about my patients and just like thinking about admittedly like sort of the world I would want to live in, um, the world I want to live in. Um, I think one thing that gets to me almost on a moral level is, um, having people sort of at all levels, whether it sort of be people in society, but then particularly judges, prosecutors, uh, people who actually make the decisions about what happens to people, um, sort of think about, I don't know, in a real way what is truly wrong and what is truly right. Um, and sort of being able to let that determine kind of how people are treated but then to also continue to treat them like people as they go through the system.

P: Because one thing that also gets me a lot about jail is like how dehumanized the language even is about our patients. Um, talk about. They kind of just call people male and female and use words like feeding for when they're having meals and stuff. That kind of like it, I feel like the language is much more like you'd use with an animal than with a person. Um, and I think that piece, if you can bring humanity back to the system, then even if it is like it is, there's a piece of it that would change to me. So I think that's probably one of the hardest parts about for people for seeing people who've been in the system a lot, is it just that part becomes normalized and to me that's not okay. I'm done. Off my soapbox. (laughs)

I: Thanks for sharing your perspective-

P: Sure.

I: ...today. Once we have reports and findings from-

P: Yeah.

I: ...the study, would you be interested-

P: Sure.

I: ...in receiving some of those?

P: Yeah. Definitely.

I: Okay, great. So...
