# Supplementary material for: Physicians’ perspectives on continuity of care for patients involved in the criminal justice system: A qualitative study
Source: PLoS One. 2021 Jul 14;16(7):e0254578. doi: 10.1371/journal.pone.0254578 (PMC8279398; doi:10.1371/journal.pone.0254578)
Supplement: S2 File — (ZIP) [file pone.0254578.s002.zip › Clean/Participant_4_Audio1_LJ_deidentified.docx]

I: Picking up voices which is great, um, so thank you again for taking the time to meet with me today. Like I said we just want to get a sense of what you know about the criminal justice system and how it may impact your patients and this part of a larger joint partnership between researchers here at [health system] as well as data analysts at [County], and investigators at the [University]. Um, to begin today I just want to get a general overview of what you know about the justice system, um, could you tell me a little bit about what you think of the current state of the criminal justice system here in the US?

P: Oh ha, well it's a...it's a wreck, right? I mean it's, it's biased and um, it has huge um levels of unfairness for people of color and lack of resources, and tends to incarcerate people for trivial crimes that are um, you know, should be not managed with incarceration, and um, I'm not sure it does a totally fair job of investigating and adjudicating more serious crimes that really need to be in the criminal justice system. Um, capital punishment is immoral and that we engage in it is terrible in this country, we should...that should be abolished. Um, so yeah. I'm not a, I’m not a huge fan. I think there are good people involved in the system who are doing the best thing, the system is stacked against the people who, uh, are otherwise disadvantaged in society.

I: Next I'd like to discuss some criminal justice system terminology. Could you explain to me what comes to mind when you hear the following terms, and the first is prison?

P: Uh, prison. So, similar people are locked up and um, warehoused. And it's not a setting for rehabilitation or...any sort of improvement in people's lives.

I: How about the term jail?

P: Shorter term, sort of uh, a holding, uh, space. Sometimes I - and sometimes they may have longer term but I don't know the county jail, I know sometimes people spend a bit of time there in the county jail, but it's...to me it's more often a sort of uh, a transit space where people go while they're waiting hearings or trials to sort of have disposition. But I do know people spend some-sometimes months and I think even years in the county jail.

I: Mm-hmm (affirmative)- and could you speak a little bit, um, towards how you distinguish between jail and prison?

P: Uh, jails are local, prisons are regional. Uh, prisons I assume are for more serious crimes, uh, and people have more of a - have been through the legal system and have been sentenced tend to end up, more exclusively in prisons and jails, again, I think there are people who stay in jail while they are awaiting hearings and awaiting the assessment in the criminal justice system.

I: And what comes to mind when you hear the term probation?

P: So proba...I'm gray on probation and parole, I can't keep them straight so, uh, I-I think probation - I guess if I had to try to define it - it's something people get in lieu..in lieu of time incarcerated. And parole is something that happens when people are finished with a period of sentencing and they get out. But those two terms, and I try to get my patients to tell me which they're on and they don't know the difference either most of the time.

I: And so I'd like to shift a little bit to your background in education and training. Um, during medical school, did you receive any training whether this was formal or informal, on working with justice involved populations?

P: Zero.

I: And is there any that you think would have been helpful to you during that time?

P: Uh, I-I don't know. It’s a pretty...it's a pretty focused population, you know not everyone is gonna come across it, so does make sense to train a broad group of trainees who are in medical school about something that most of them are never gonna bump up against to any significant degree, so, I think it's hard to tell-I think it's probably more suitable for kind of on the job training, post-graduate training.

I: Mm-hmm (affirmative)-

P: That would be my guess.

I: And have you received any training that you're-at this place of employment or past places of employment on this topic?

P: Nope.

I: Okay. Let's see and thinking of, jumping a little bit backwards again, but did you receive any training during residency?

P: No.

I: And how about during a fellowship when you...

P: Nope. Not there either.

I: Hahaha.

P: Hahaha.

I: Do you think that there would have been any unique, um, trainings either during residency or fellowship that would have been useful?

P: I - you know, again it all depends on where people choose to work, right? So for, in this environment I bump up against the criminal justice system a fair bit. So, probably someone should have given me some information at some point, maybe on work orientation here, would be a good place to put it in. Um, is it worth training, for example the residents who come through this program here? Maybe, maybe that would be some value in sort of, but the vast majority of them aren’t gonna remain engaged with this kind of popu...the populations that are here or have much connection with the criminal justice system so, um, but I think for people who work here in this setting it might be a valuable thing.

I: During your visits with patients do you ever ask about whether or not they're currently, or have formerly been involved, with the justice system?

P: Uh, on occasion. Mm-hmm (affirmative) but um, you know, it tends to be stuff often that you don't wanna know about. Um, it uh, for me, it has importance if it has implication for other problems I'm trying to manage. For example, chemical use problems or mental health problems. If they have a condition of, of from the criminal justice system, probation, parole, whatever, that mandates some engagement in those that's useful to know about. Some people have to get drug testing as a condition of their probation and so that's - that's useful to know about. Uh, I work in our high risk clinic here and occasionally we have connections with probation/parole officers to talk about how people are doing.

I: And when-

P: But I don't routinely ask about it and I don't...I try not to ask about what they did cause I, you know uh, and sometimes we find out and it has a lot of impact on people's housing right? So, people who have any sort of felony that's...it's a terrible problem for them you know breeds homelessness, right? So, understanding that is a factor that's a limitation to their being housed is an important thing to understand the limitation, right? Sometimes we find that out, I don't, but say if I work with, we'll find that out by looking on governmental websites. And there's stuff there about, you know, people's level - charges, sexual offenders, and whatever else. I've been aware of that information generating charges of murder on a patient I had to take care of. Those are, and you try to sort of, uh, distance yourself from that as you're thinking about, you know the medical problems people have.

Um, so its sometime just better not to know that. I like to know the implications of it, right? So if someone's mandated to have testing or mandated to have treatment or if it creates a barrier I like to understand that, um, that it's there in place. But I don't necessarily wanna know all [inaudible 00:09:29].

I: And when you do ask patients about their involvement, how do you frame that question?

P: They usually bring it up. So, um, so I don't have a standard approach it's sort of, wherever they start talking about it. But I try not to probe too much, you know I’ll ask them about if they've, again things that have an impact for my care for them. Do they have any required treatment or assessments? You know, how is this impacting houseability, those are kind of the issues that I focus on.

I: And you mentioned some communication with probation officers. Could you tell me a little bit about the types of information that you're exchanging?

P: I would say...I don't talk to them directly so it's usually people I'm working with, care coordinators, or, um, social workers, and usually I would say the predominant thing is around requirements for, uh, drug testing. That's kind of a prime...or drug treatment.

I: And what do you think some of the benefits are about asking your patients about their justice system involvement?

P: Well I think the more you can understand about, sort of, what factors are controlling people's lives - I mean, you know, if you're in the justice system you have a certain amount of limitations, right? Things you can and can't do, and so it's important to understand that.

I: And what are some of the risks and challenges that you see to broaching this topic with your patients?

P: Well then I think it’s, again I tread lightly so and I'm sensitive to that. They may rightly think it's not relevant to their healthcare you know, that they have medical problems and they want to treat it medically. And that's, I think, that's appropriate. Um, so I think you just have to be clear about if you're asking questions about that - why you're asking, why you're looking for that information. They usually understand particularly around - cause I'm concerned with people getting into drug and alcohol treatment, that people get their mental health problems addressed in a structured fashion, and so if criminal justice system can encourage that, I'm all for that, and they kinda understand that. But I do think they, you know I'm sensitive to the fact that they don't need to talk to me about this, you know their criminal activity whatever it was, sometimes they'll just-they'll tell you cause they want to sort of, uh, demystify it, you know. They had a UD, so what, you know, you didn’t pay [inaudible 00:12:24] right? You're selling marijuana, big deal right? Um, so but to me it's more the output of the system that's relevant to my interaction with the patients and not what got them into the system.

I: So you mention that you work in a high-risk clinic, could you tell me more about your overall patient population?

P: So we take care of people who are, um frequently admitted to the hospital and have a sort of-they have high needs for medical care, and are high cost patients. Because of those needs, typically um, the things that are causing it to happen are lack of shelter, um drug and alcohol use, untreated mental illness, um lack of community support, family support, poverty, those things. Um, so all those kind of social determinants are driving all this medical expense and so, so we work in the clinic to try to, as opposed to just sort of putting another mandate on people and giving antibiotics for the bronchitis that's coming around, we try to address the underlying issues and get people housed or into treatment or, connect them with mental health professionals or create a structured living environment where they can be more successful.

I: And have you noticed any particular challenges or barriers faced by patients of racial or ethnic or minority backgrounds in your work?

P: Uh, in general?

I: In general.

P: Yeah.

I: The patients that you're seeing day-to-day.

P: Yeah, I mean I think we do a pretty good job of opening the doors in this facility to everyone, can come in. But I think that people are human, and people take biases into their work, you know. So, I certainly do see that, as part of what goes on here. We all try to, you know, patch that up and make things as fair and equitable as they can be. But I think it's part of the system, you know, it's part of the criminal justice system, it's part of the healthcare systems, it's part of the United States unfortunately. So, yeah.

I: And one of the things we're also interested in learning more about in terms of your general overall population, is an estimate of the income levels of the patients that you're seeing or perhaps I think sometimes insurance status is another way of how to get at that?

P: Right, so in the high needs clinic that I work in, everybody has medical assistance so everybody's poor. Uh, and then I also have a clinic across the street; I would say that um, I don't know what the payer mix is in that clinic, but I would estimate that probably, well over half, have medical assistance. Uh, there's a percentage who have no insurance and the rest have Medicare or some other form of public insurance; very few people have private insurance. In the clinic I work across the street, I would estimate that less than 10 percent of my patients are employed, probably less than 5 percent. Many have disabilities, uh, public money pays for the healthcare that I provide.

I: And approximately..

P: And their income levels are super low, yeah, I mean you know, the single income limit for medical assistance is less than $1,000 a month of income, so many people are living with pennies. So they're heavily dependent on me, public services, um you know, the food stamp program, public housing, all these things are supporting people.

I: And how many of your patients would you say are impacted by disabilities?

P: Uh, well, um, I...broadly defining disabilities as the inability to keep a job, is that - so we're not talking physical disability, you wanna know - I mean chronic mental illness is a disability right?

I: Yeah, I'd be interested in - however you differentiate and explain to me how you're distinguishing between your [crosstalk 00:17:16].

P: Uh, I don't think, you know, less than 2 percent of the people in [high risk clinic] work. Um, I would say that uh, of that, of the 98% that are not working, probably half of them are on federal income support for disability - SSI or SSDI - um, or social security for disability. And the rest are probably making it on general assistance and um, which is nothing and so they uh...but in that group, um, most of them are not capable of employment um, and that tends to be more about social determinants as opposed to patient-specific determinants of their health. You know they're homeless and so they can't have a job, or only with great challenges would be able to have a job; or they have an untreated chemical health problem um, that would prevent them from participating. So they're able-bodied so they can't get through the social security barrier, right, but they're not really employable.

So I would say that's the case in the high-risk clinic is that there's almost no one there who really is capable of participating in the work force in a meaningful way. So they all have disability, they just don't meet the federal criteria. Then, across the street, there are probably more people who conceivably could work or did work, or you know are past retirement age, um, so, and uh...so it's a little murkier, it's a little murkier there. But there are still a lot of people on general assistance and general medical clinics, um, and a lot of young people who aren't working or on disability, one percentage.

So there's more aging, there's more of an aging population across the street, so it's hard to sort of tease them out. But of the less than 65, I would say, uh I don't know, maybe 10 to 20 percent are able to work and the rest are either technically disabled there's a very high prevalence of chronic mental illness in our medicine clinic here that is disabling, likely disabling, so um and a fair number of people with you know, physically disabling conditions and then a percentage who are in this sort of homeless ,chemical health, severe socially disadvantaged who just, who are just never gonna get into the work force. I hope that answered your question.

I: Yeah. Um, now thinking specifically about patients who have criminal justice involvement that you've treated, can you speak a little bit to what that experience has been like for you as a provider?

P: Uh, so if - one thing about it is they kind of just, there are huge discontinuities in their care, uh, both going into the system and as they get regurgitated out. There's no um, effort or system of re-entry healthcare planning. Many people who leave the criminal justice system don't have a lot of medical problems, but I take care of a significant group who do, and I'm thinking right now of three of the guys I take care of down in [high risk clinic] who've been in and out. Um, and how badly its gone on both sides, for both of them, uh - they sort of are set out into the system and they have some pretty serious problems with kind of fend for yourself. I mean they're pushed out into um, and maybe sheltered, but no one's sort of coordinating transfer of medical care back to a medical care setting.

I had a couple guys come out of the state you know, penitentiary, in the last six months and we have had relationships with them before they got locked up so they knew to come to us and they know they can call us. And we have...and so both of them have showed up, um but if we hadn't been there to...and had an antecedent relationship with them, they both have pretty serious medical problems, um, that would have gone untreated maybe with disastrous complications.

And another guy who came out of, uh, the criminal justice system who really had been in the penitentiary for most of his adult life, um, around sex crimes, which um, he got sent out and for some reason, and I think he even came from out of state, but he um, someone made an effort to coordinate his care coming out and identified our clinic as a place for him to go. So that...at least there was a, and they delivered medical records from the penitentiary and as I said he had been there for 20-something years on and off.

Uh so, they did a - made efforts to try to coordinate his care coming out but the problem for him is that he doesn't know how to live outside the penitentiary and so um, he has a whole series of kind of uh, maladaptive behaviors around his health, uh, that he created while he was in the system. And things aren't going well for him um, so, uh he's remained homeless and wandering the United States - goes out to Colorado, down to [clinic name]. (laughs) He’s a real vagabond. He's been...he's estranged from his family, uh he shows up in emergency rooms with self-injuries, behaviors for attention, things that might have worked in some way for him in the prison system but it's not, it's not working out very well. So that's kind of sad, it's kind of a sad story.

So, the discontinuity going out is a big issue, and then I spoke before about uh, you know, people get on um, parole and uh, I mean that might have some implications in connecting with their PO might be a useful thing to do so…

I: Um, are there any other instances besides the one patient that you mentioned that was referred to your care, where you do have other patients who are coming out of the justice system who do get referred to you? Or...

P: He's, he's really the main example that I can think of. A lot of people even criminal justice they don't have, you know I - uh, [high risk clinic] which is the clinic I work that's the high-needs clinic and you know we take care of people who are - have medical issues that have put them in the hospital all the time, so. You know I suppose an appropriate referral from the criminal justice system would be someone that spent a lot of time in and out of medical wards while they were in prison; that would be a good person for us to try to help. Um, so but I don't think that, that uh the needs of people coming out of the criminal justice system are always heavily medical, right? They don't necessarily have a huge burden of, of chronic mental illness that needs to be managed. They need a doctor, right? Um, and you probably know that there's um, um, I forget the guy's name – [name] somebody - who does this program around reentry, um, you know what I'm talking about?

I: I think so? I'm [name]?

P: No...

I: That may not be the last name.

P: No. He has a, he, and I met him about six or seven years ago, he connected to the Department of Medicine, but he, he runs a re-entry program that's centered around giving people place to live, and some meaningful activity. And he, and they were doing...sorting recycling, last I heard about it, and he had, I can't remember the name of the program.

I: Is it [community organization]?

P: I think it is.

I: That one?

P: Yeah, so anyway for a while he was bringing people to our medicine-direct care clinic, to just sort of connect with a doctor and get assessments. But a lot of them, again, they might need a check-up or claim that they need some [inaudible 00:27:29] care for whatever intercurrent illness they have. Some of them might have high blood pressure, diabetes, but they don't necessarily have a huge burden for the healthcare system, on an ongoing basis. And a lot of chronic pain problems, I'm sure.

[30 minute time warning from notetaker]

I: [inaudible 00:27:49], thank you.

P: Thanks.

I: And so you have, you've mentioned some of the social factors that are impacting patients such as homelessness, um, are there any that you haven't mentioned that you're seeing among justice-involved patients at all?

P: Uh, well the, uh - I mean obviously there's criminal stuff going on; some of them have um, weapons obviously and so that's an issu- that's a health risk right? Um, one of our guys who's been in and out, I mean they get in altercations and - so whatever drives all that you know, that's a..that's a health risk for them to be sure, right? Um, whether it's being in the wrong place at the wrong time, being in a situation where they feel like they need to arm themselves, right? For safety, you know, so...uh, I don’t know what the proportion of people coming out of the criminal justice system who might be carrying a firearm you know, that's a risk factors for a bad health outcome, to be sure.

Um, drug trafficking is something that I see in people who are you know, we have a fair number of people who are doing that and getting involved around, you know, selling drugs and stuff so that's an issue, so, um I guess those two things I would think about, there's not much I can do about those. (laughs) I try to tell people not to carry weapons but you know, people do what they do and um, if they have a felony and I think they're not allowed to have firearm, right, but that doesn't mean people don't have them right? Or other kinds of weapons or you know, clubs or bats or, uh, lead pipes or whatever you need to defend yourself, so.

But the homelessness is a...is a huge issue and it seems to be - you know, it's a huge uh, determinant of ill health, right? And um, the housing barriers for people who come out of the system are, it's terrible. They have no money, if they can get a job that's great and they can get a market-rate apartment but if they're living off whatever minimal income they might get out of that system, there's no way they can house themselves so they’re in shelters, or on the street, so it’s bad.

I: So we talked a little bit about your overall patient population. Thinking again to your patients specifically with justice system involvement, what are they dealing with medically?

P: Um, blood pressure, diabetes, lung disease, asthma, COPD, those would probably be the big - the big things.

I: And what -

P: Heart disease.

I: What are some of their mental health needs?

P: Oh, those are huge, right? So, uh, again I'm focusing in on these three guys who I know have been in and out; I mean they all have depression and anxiety, or other very complex mental health problems that are outside, you know, primary care management. It’s not like a little stress reaction. These people have longstanding issues: PTSD is common, as they mostly have been in some kind of trauma um, there's a lot of you know, personality dysfunction uh, and so they have a hard time fitting in with personality disorders. Uh, all those things are super hard to treat and it's nothing like people who might be uh, living out in [suburb name] and taking a little sertraline to kind of, get through the day.

These people have serious mental health problems, so. So, and to sort of swing back around their general needs around health. That's the mental health and chemical health issues; a system that could address those as people exit the criminal justice system if you were targeting your providers on those issues that is something that has robust need, unlike most of the other stuff, which I would consider is sort of, manageable, um, chronic, medical problems. Some of them are going to have some bad stuff, you know, people do get heart disease, stuff like that, but the mental health problems are huge, and the chemical health.

I: Can you tell me a little more about the chemical health needs of your patients?

P: So lots of...it's everything. I mean they use all kinds of substances, um, opioids, and cocaine, alcohol, uh, all of these things have effective therapies. People with opioid use problems coming out of, uh, out of the criminal justice systems should have rapid access into a methadone program, I mean that's a critical issue if they've been in the system, and have not being exposed to any opioids, when they come out, they're at very high risk of overdosing death. So, uh, if they feel like they um, want that kind of treatment, the doors need to be wide open for that or suboxone therapy, if they can find a suboxone provider.

Uh, so pharmacologic treatments for alcohol use disorders, people should, you know, get an assessment and if..cause those things if they go untreated and unmanaged, they're just gonna land people back in uh, the penal system, so. There needs to be some kind of way to kind of, assess and engage people around those things.

I: Are there any other resources or services that you wish were available to your patients?

P: Uh, the housing thing, I don't know how people navigate that. That seems like the biggest issue. Yeah.

I: And then thinking broadly about our healthcare system, are there any other changes that you would recommend to make it better meet the needs of patients who do have some type of justice system involvement?

P: Well, uh, I mean, everybody needs insurance right? So I don't know what happens to people when they leave the criminal justice system; uh, you know, how easy is it for them to get on medical assistance, which they should have as a bridge to something. The application process for medical assistance, as you know I'm betting, is horrendous right? Uh, you know, you need a PhD to navigate the system - it's not user friendly, it's not language sensitive. I mean at the end of this, is that we need universal coverage in the United States and the system's idiotic right? I mean that's - that's what's behind my comments.

But to have people coming out of the criminal justice system and facing the hurdle of not having insurance is ridiculous right? So that needs - that wheel needs to be greased in some way, um, and the ultimate solution of course is everybody gets covered all the time but, our country's too stupid to do that so, um. So we need some kind of a jury-rigged solution to keep people, you know, covered by public programs for X period of time, automatically. I don't know what's in place, but there shouldn't be a discontinuity, um; I see people leaving the criminal justice system with a list of medications and 27 day supply on [unrecognized drug name 00:36:56], cause that was what was left in the dispensary; and then they're supposed to figure it out - that's not gonna happen.

So, if there are untreated, chronic medical problems or there are, um, mental health issues that need attention, those are just all gonna decompensate abruptly, so.

I: So thank you for your time today.

P: Yeah.

I: Before we wrap up, is there anything that I didn't cover today that you'd also like to add on this topic?

P: I don't think so.

I: Okay.

P: Yeah.

I: So thank you again, um, like I said we're gonna be interviewing additional providers from several other, um, healthcare centers. Once we have any written reports or findings from this project, would you be interested in receiving those?

P: Sure, of course, yeah.

I: Okay, so thank you again.
