# Supplementary material for: Physicians’ perspectives on continuity of care for patients involved in the criminal justice system: A qualitative study
Source: PLoS One. 2021 Jul 14;16(7):e0254578. doi: 10.1371/journal.pone.0254578 (PMC8279398; doi:10.1371/journal.pone.0254578)
Supplement: S2 File — (ZIP) [file pone.0254578.s002.zip › Clean/Participant_5_Audio1_LJ_deidentified.docx]

I: Um, like I've said, again, we're interviewing physicians to better understand what they know about the criminal justice system and what they know about treating patients with criminal justice system involvement. Um, I want to begin today by getting a general overview of what you know about the Justice system. Could you tell me what you think about the current state of the Criminal Justice System in the United States?

P: Well I suppose that would be a pretty complicated question, huh?

I: Mm-hmm. (affirmative)

P: Um, uh, my, my general impression of the Criminal Justice System in United States uh, well it's large and in charge. And uh, you know, with, houses many, many, many t-, you know, tens, I don't know how many. If it's hundreds of thousands of prisoners, or tens of thousands, probably I would guess hundreds, prob-, probably even more, possibly more I should say. Um, I guess my overall impression is that I have a lot to learn about it.

I: Okay. And next I'd like to discuss some Criminal Justice System terminology, and I'll run down a list of several different terms. Could you explain to me what comes to mind when you hear the term prison?

P: Prison, long-, longer term uh, incarceration, longer than one year, uh, for serious offense. And uh, not a place where I personally would like to be, or not a place that I would uh, wish upon anybody.

I: And so what comes to mind when you hear the term jail?

P: More short-term incarceration, up to a year. Um, like a, a period of incarceration as a person's um, offense is sorted out, whether or not they will be uh, you know, I guess uh, released. If they're, if it's deter-, you know, if they're waiting for their court date basically. If they're, um, if the, if the time that they are to be incarcerated is going to be you know, lasting for any amount of time longer than that short-term stay, up to a year.

I: Can you tell me a little bit about more of the similarities and differences between jail and prison?

P: Well, I mean your rights are taken away from you, you're being held in a cell. Your uh, I guess a difference would be that uh, uh, you know, in jail you would be awaiting um, you know, an outcome of your uh, you know, of, of uh, whatever kind of legal situation you found yourself to, to be there in the first place. Whereas, in prison it might be uh, more defined and sorted out, in that you've been, presumably you've been uh, you know, tried and sentenced and you're serving your sentence for you know, a predetermined amount of time. Whereas, jail uh, would be potentially more indefinite. You're awaiting your, your, your uh, court date to see what the outcome of that would be.

I: And so what comes to mind when you hear the term probation?

P: Uh, probation, what comes to mind for me would be you've been, uh, you've had some kind of interface with the Criminal Justice System. You know, you've either spent time in jail and/or prison. And you've been released um, earlier than you might have uh, originally been planning on for a sentence. And it is a supervised um, I guess you know, relationship that you have with the Criminal Justice System in the community.

I: And could you tell me what comes to mind when you hear the term pro-, oh parole?

P: Parole, I was just thinking that at the end of my last ... Uh, about the same actually, probation and parole, I don't know the difference.

I: Mm-hmm. (affirmative) Okay.

P: And in my mind I would describe them the same that I, uh, you know, in or-, or the same or in a similar way that I did with probation.

I: Mm-hmm. (affirmative) Okay. So now I want to shift a little bit to talk about your background in education and training. Um, during Medical School did you receive any training whether that was formal or informal on working with justice involved populations?

P: I don't think so, uh, I don't, I don't believe I did.

I: Okay. Um, is there any training or education that you think would have been helpful to you during that time?

P: During Medical School or Medical Training?

I: Mm-hmm. (affirmative) During Medical School?

P: I think having access to uh, you know, um, I think having access to you know, a or the jail, like the County jail, or the County workhouse, or a prison uh, certainly would give you good insight into um, the experience that a person might be you know, living when you do see them in a different setting. Um, so whether that would be a rotation as a third or fourth year medical student or a first, first or second year medical student, or as a resident. I mean, I certainly think those could be fruitful. Um, you know, I have spent basically all of my clinical years at [health system], where there's more of an overlap with um, patients that have been in jail. Either by, like I'll see them in jail, I'll, I'll work at the jail sometimes.

Um, but I certainly have a lot of patients that have experienced incarceration at some point in their lives. Um, you know, I can't say that uh, that it's any more or less than you know, at another healthcare facility. It, it certainly could be more but I, I don't know for sure, you know.

I: Mm-hmm. (affirmative) And did you receive any training during residency on this topic?

P: Um, nothing formal regarding incarcerated patients. As a resident, I, I would moonlight at the County jail.

I: Mm-hmm. (affirmative) Could you tell me more about that experience?

P: Yeah. You'd show up early in the morning, like at 6:00 or 6:30 I think it was, for uh, the sick-call clinic. When you'd see probably six or eight patients uh, with varying concerns from, on their end you know, from you know, you know, “I have a cough” or “I, uh, my elbow hurts from you know, uh, you know, some fight I got in.” Or um, you know, “My back hurts, can I get a pillow,” um, to uh, you know ... I had a, “This patient had a, had a hospital stay and they need follow-up,” you know, and read their chart so you can figure out what you're supposed to do. (phone interruption) I'm getting paged, I'm sorry.

I: Okay, no worries.

P: Um, so that, and then that was one element of my experience there. I certainly, I think I, uh, additionally I saw a power dynamic there, that um, I think was hard to miss. One, like the nursing staff, not as a rule but it felt like that's where nurses went to put themselves out to pasture at the end of their career. And they didn't always appear to be engaged in the um, I guess the outcome or like the overall care, or advocacy of the, of the prisoners. And I'm not trying to sound self-righteous, I'm just trying to offer what I think I saw, you know.

I: Mm-hmm. (affirmative)

P: I think that people got blown off a lot with their symptoms. And um, uh, you know, sometimes among the deputies too, not everybody by any means. But um, it, it felt like you know, there certainly could be p-, you know, serious medical concerns that could easily be missed because uh, you know, the, the uh, inmates weren't being taken seriously at times.

I: Mm-hmm. (affirmative)

P: So that's also part of my experience there as a resident, and what I, what I saw and what I kind of informally learned.

I: Mm-hmm. (affirmative) Okay. And did you complete a fellowship?

P: No.

I: No, okay. Um, have you received any types of training here at [health system] or at a former employer?

P: Nothing formal.

I: Mm-hmm. (affirmative)

P: No.

I: Have you had some types of informal training?

P: The only uh, informal training I would have would have been watching [doctor’s name] grand rounds last year.

I: Mm-hmm. (affirmative)

P: Uh, describing some of the uh, demographics and initiatives you know, in the um, incarceration and post incarceration care of, of patients.

I: And during your visits with patients, do you ever ask them about their current or past Justice system involvement?

P: I don't ask them usually directly. Frequently you know, we'll have access to at least the, uh, their presence in the, in the [county jail], when I open up their medical records so I can see if they've been in jail recently or in the distant past I guess, as well. Um, uh, typically I don't ask that as a, as a routine question, like in their social history. Um, but also not infrequently you know, it's brought to my attention, like, "I was just released from prison, um, and I'm you know, here to establish care. And I need you know, medication refills" or, or whatever. Or, “Where were you for the last three years, we haven't seen each other for a while?” “Oh, I was incarcerated, and I had to be in Illinois and, and serve a sentence for whatever happened,” you know.

I: And are you ever asking that patients that question more directly?

P: Generally I don't.

I: Okay. All right, and for patients that do bring it up and volunteer that information, how do you use that information to inform your care?

P: Um, well I guess the more standard answer, the, the first one that will come to mind wo-, would be you know, if they're, you know, are they at risk for any communicable disease like tuberculosis, um, HIV infection. If there is addiction that we are addressing you know, I wonder are they at high risk now of uh, overdose necessarily, if they're um, you know, recently released. Um, you know, “What kind of medications were you uh, being treated with while you were, while you were incarcerated? And what have you been getting or not getting since then?” Um, and, “How long has it been since you've, you know, have been treated or not treated as you know, for your chronic disease? You know, are you housed, uh, was there any kind of uh, you know, aftercare as you were released or did they just open the doors and say, 'Go for it.'”

Um, you know, you know, housing like I said you know, “Can you get a lease? Who are you living with?"

I: Hmm.

P: "Can you work, are you working now? Are you looking for work? What are you doing to keep busy all day long?"

I: Okay. And are there any benefits that you see to asking your patient or talking about this with your patients?

P: Oh sure. I think just i-, uh, I mean, the first ones that come to mind are what I was just speaking of. You know, “Are you working, what are you doing to stay busy, are you stably housed? Are you ...” you know, in my mind, you know, “Are you at risk for um, is your, is your, are you at risk for uh, tipping one way or another with any issues in addiction?” Um, "Yeah, and how are your, are your chronic diseases being managed or not medically? You know, do you have insurance you know?"

I: Mm-hmm. (affirmative) Okay. Are there any risks or challenges to asking about this, that you see?

P: Well I, I mean, it's a, f-, it's uh, it feels like it would be a sensitive subject you know. Uh, maybe it's different for everybody too, but um, I could see there being a certain amount of um, shame and stigma with going to jail or going to prison. And somebody might not feel like talking about it. Um, you know, I am aware of, a power dynamic between myself and a patient, as I am in a room with them frequently, not all the time. But you know, I see myself as coming from a place of privilege in, in my day-to-day and um, I'm, I don't feel like I'm at risk of going to jail or prison. And in that way I don't have you know, as nuanced an understanding of what that might mean to be in that place. So I'm, I'm aware of there being certainly a power dynamic between myself and that patient.

You know and, and uh, so that would be another reason that I probably don't ask it is that it feels awkward to me also.

I: And could you tell me a bit about your overall patient population, just thinking about all the patients that you treat here-

P: Mm-hmm. (affirmative)

I: And some of their characteristics?

P: Um, I think there's a pretty good cross-section, um, I work in the internal medicine clinic. I feel like in the last two or three years, my patient panel as I look at them, have become um, people that we've kind of self-selected I think. Uh, people that have stuck around for a longer period of time, might be less transient. Um, um, (long pause) but I see a good cross-section of, of people both in the hospital and, and in the clinic I would say. Um, I don't know what the, you know, the actual numbers would be but it feels like I see a lot of people that are marginally housed. Um, either sleeping with fr-, friends or relatives or just home-, you know, on like streets or shelter homeless. Um, but a lot of doubling up um, you know, I think a lot of the patients that I see, I've, I've changed how I, how I um, I guess phrase my social history when I'm, when I'm speaking with patients.

Um, you know, in medical school you're taught to uh, move through your history and physical in a very prescribed way. And one would be you know, the social history, “Do you smoke, do you drink, what do you do for work, where do you live, you know, do you have kids?” And I generally don't ask people what they do for work, because I feel like many of the people that I see, don't work. And so I fr-, rephrase it at, “You know, what do you, what do you do to keep busy during the day,” you know, or, “Have you been, you know, uh, if you're working now what are you doing you know?” Um, you know-

I: Have you made any other changes to some of those social history questions in addition to the employment question?

P: Um, I don't say, uh, I don't say, I'll say, “Do you drink, or do you drink alcohol?” I don't say, “Do you use cocaine, do you use methamphetamine, do you use heroin, do you use opiates.” I say, uh, you know, “Do you drink? You know, do you smoke cigarettes, do you use any recreational drugs?”

I: And how would you describe the income levels of your patients?

P: Um, I think again, there's a cross-section. Probably more of the patients that I see are on medical assistance. Um, you know, and I don't know what kind of income people are actually taking in, uh, but I would think that a majority of the patients I see uh, are not making a lot of money. Um, if they are working or, and/or if they're not working they're either receiving disability or they're receiving assistance uh, for a short you know, term until they're working again.

I: Mm-hmm. (affirmative)

P: Which I know is not much money.

I: And in your practice, have you noticed particular challenges or barriers faced by patients from ethic minority backgrounds?

P: Well I, you know (long pause) Have I faced particular barriers or challenges by patients?

I: Or have you noticed your patients themselves experiencing any barriers or challenges um, in terms of accessing care?

P: Uh, yeah, I think so. I mean, I guess this, you know, it's like whatever I think is probably not necessarily what's going on too, I, I recognize that. Um, for example, I was, I followed a guy whose um, he's probably 60. He's been on dialysis for about ten years now. You know, uh, like a 60 year old African-American man, who has uh, you know, has hypertension, end-stage kidney disease, and uh, and a chronic pain syndrome. Um, so his f-, the fistula that he's dialyzed through was, was dilated and had this big aneurysm. It was like big, it's like the size of your fist.

I: Mm-hmm. (affirmative)

P: And it was kind of getting bigger and bigger. He had these skin changes over it, and he felt like he was being ... I was like, “Oh, what's going on there?” And I like physi-, the, I know the physiology of it. He has this, a stenosis closer to his heart than the fistula is, and it blows up and it gets all dilated, uh, and it happens sometimes. Um, his impression he told me, was that he's being experimented on by the people at the dialysis unit. So that wouldn't have ever occurred to me to uh, ask him that, you know.

I: Mm-hmm. (affirmative)

P: Um, very distrustful of you know, of me, it feels like of me. You know, even though I keeps-, we've known each other for uh, like eight years now or something you know. Um, but he keeps coming back to see me, you know.

I: Mm-hmm. (affirmative)

P: Um, you know, other barriers that uh, patients that I work with might have experienced due to their ethnicity (long pause) In general, I mean I think of a Somali guy that's been here since the early '90s. He's a citizen, has been a citizen and business owner for a long time. You know, he was, he told me he was uh, yelled at when he was filling his gas station, or he was, he was at the gas station filling his car. You know, somebody yelled at him, “Hey, packing your bags, getting ready to go back?” Um, I guess that's not necessarily a, clearly a barrier to medical care, but um (long pause) Um, what else? You know, language. You know, every time I'm talking with, there's a lot of older Russian patients that are in the medicine clinic. And um, there's like two uh, Russian interpreters okay, and uh, that are, that will come in person. You can talk to other interpreters on the phone-

I: Mm-hmm. (affirmative)

P: But uh, when they, and they all know each other. And so I'll say something like, "You know, have you been taking your diet, your, have you been taking your insulin? What dose of insulin have you been taking?" Just like a one sentence question. And then they start talking to each other and they start arguing with each other, and exchanging a fair amount of dialogue, for a while. Like a minute of straight up talking. And then the interpreter will turn to me, and they're like, “He's taking his insulin.” And I'm like, “What did, what did you guys just say? You know, just tell me what you said.” And he's like, “He's taking it, don't worry about it, you know.” So there's something lost in there I'm sure.

I: Mm-hmm. (affirmative)

P: Um, you know, so um, yeah, certainly in language you know. I mean, we have you know, the interpreter services are very strong, but you know, in language I'm sure somethings lost. Um, I am just always impressed by um, the uh, the experience that a patient can have, like, in, in terms of what they, you know, hear, how they interpret what somebody else is saying. Um, and what they, what they take with them. And something that I might say in passing has come back to me you know, like years later. Or I, of course, no recollection of ever having said that, and um, you know, like what I might have meant was interpreted in a way that I, you know, is just very different than I would have expected, you know.

I: Mm-hmm. (affirmative)

P: Um, and that's not, I suppose it doesn't have to be uh, related to somebody's ethnicity, but that you know, can be anybody and anything too. But um, oh yeah, there's, what did, yeah, so here's something, here's another one. This will be the last suc-, this will be another example.

I: Mm-hmm. (affirmative)

P: I was seeing a guy in the medicine clinic like another, he was, he was a guy, he was, he was like maybe 50, an African-American gentleman. Who, I was like, “Are you ready for a flu shot?” This was like three weeks ago. Uh, “Can I get you a flu shot today? You're due for a flu shot, and a tetanus shot, and a pneumococcal vaccine.” And he was like, you know, his words, he's like, “Black people don't like vaccines.” And I was like, “How come?” And he said, “They leave scars on your arms, and you know, you can see this little scar I had from when I was a kid. And uh, I don't like it and my friends don't like it and none of us are going to tell you that, but that's what's happening.” And I was like, “Oh, okay, so do you want the tetanus shot or, or not you know?”

So, that's something I never would have guessed. And uh, that certainly could be a reason that if he hadn't chosen to tell st-, to, say that to me today, or that day, then I would have just been like, "Oh, I don't know why he wa-, doesn't want a flu shot. He's not, he doesn't want the flu shot you know."

I: Mm-hmm. (affirmative)

P: "Next, let's keep going." So um, that would certainly be a barrier to his overall health if we're thinking about just vaccinations you know.

I: And I was thinking about your first example about the 60 year old African-American patient, with, and his distrust of the healthcare system. And was wondering if you could talk a little bit more about how you've responded or how that relationship has continued, um, in terms of I guess your response to that and him sharing that information with you?

P: Mm-hmm. (affirmative) Um, I didn't feel compelled to try to explain otherwise, you know, because who am I to tell him otherwise? Like, I don't think any, I don't think anybody's experimenting on him, but I also don't think that I'm going to be able to convince him otherwise necessarily. Um…I mean, he seemed pretty firm on that. And uh, you know, we could get into it, and I could just kind of talk at him for a while, but it's not doing anything except trying to make me feel better about the people at the dialysis unit. And uh, that doesn't serve him. I mean, I guess I could say, “Why do you think they're experimenting on you, you know? What makes you f-, feel this way?” We didn't totally explore it, but ...

I: Okay, thank you. And now thinking about your patients that have some type of justice system involvement. I know you've mentioned that you've worked in the jail clinic, could you talk a bit about what that experience is like for you as a provider?

P: Um, it's ... Working in the jail clinic?

I: Working I think, let's, working in the jail clinic and also any experiences that you've had here on the downtown [health system] campus as well.

P: And, and, and say it again, like in what context, how working in the jail and working here has ... ?

I: Uh, just a general, broad, ha-, what is that experience like for patients that have some type of justice system involvement.

P: Oh.

I: Whether they're here in the community and coming to [hospital]

P: Yeah.

I: Or if they're in the jail clinic.

P: What do I, what do I think their experience is like?

I: Or what has your experience been like as a provider for these patients?

P: Okay. Oh, I don't know. I mean, I would hope that, I would hope that I'm kind of uh, doing the, the same kind of work I would do for somebody that wasn't in jail. Um, you know, that being said, if, if uh, you know, if they were presenting with symptoms that would make me wonder about so-, you know, T-, you know, TB, um, or you know ... Or any other condition that might be associated with um, a population of people that are living closely to each other, then I suppose I would miss that if I didn't know that they had been incarcerated. Um, uh, yeah, I guess I'm not totally following the question.

I: Mm-hmm. (affirmative)

P: What's my impression from working with people here at [health system] or at the jail, in, with regards to them, know that they had been in jail at some point?

I: Mm-hmm. (affirmative)

P: I don't know, I mean I try not to, I don't ask why.

I: Mm-hmm. (affirmative)

P: Uh, sometimes they might tell me or you're, you know, you're informed that you know, they're not able to go to a nursing home because they're you know, a registered sex offender, or they've had so-, uh, usually yeah, something like that. Um, but I don't say, “Hey, what did you do and when did it happen,” you know. Um, uh (long pause) it's kind of their business you know, and I don't want to know. I'd rather just treat them, and try to treat them, you know, respectfully, rather than think of them as a murderer or a rapist or a pedophile, you know. Which that's who, that's who some people are that we're taking care of you know.

I: Yeah.

P: And uh, I don't want to, I don't want to think of, I don't want to bring that into the room.

I: Are there any differences in, that you've noticed in terms of working in the jail clinic versus how you work here at [health system]?

P: Um, well I mean, I, the parameters that you're allowed for are a little different. Like they really try to take care of the work there, rather than have somebody go to the emergency room, or go to physical therapy. Or you know, the care that somebody has access to there, is uh ... I mean, they're not in a hospital you know. But if they were in a clinic, if they were seen in the medicine clinic I'd say, “You know, your knee hurts, I'm going to get you to the Physical Therapy clinic, or I'm going to get you to see the Chiropractor, or the Orthopedist to you know, consider a knee uh, replacement.” Well that's not happening there, you know, so you know, maybe I'll, maybe I'll get an x-ray to make sure there's not an infection or some effusion or something like that.

Um, but they're not, they're not, you know, unless there is um, you know, an acute concern, they're not getting specialty care you know uh, similarly until they're out you know, uh, at best. And similarly um, you know, I can manage their in-, diabetes, or their insulin, or their blood pressure uh, while they're in jail, but it's hard to know like what's going to happen once they leave you know. I can tweak all these meds and get the right doses but you know, what's the follow-up going to be? And whoever sees them isn't necessarily going to see my, my notes or my thought process. So those are two ways that I think people are treated differently in jail.

I: Mm-hmm. (affirmative) Okay. And in your job do you ever communicate with the courts at all or probation or parole officers?

P: I have gone to Court um, to like be a witness for a, uh, Guardianship before. I guess that's not the Criminal Justice System necessarily, but it was in you know, the courtroom at least. Um, a lot of times uh, or people, I don't, I don't communicate directly. I could I guess, you know.

I: Mm-hmm. (affirmative)

P: Uh, sometimes people will come in and say, “I need to get a urine sample for my parole officer,” you know. Um, but I'm not calling the, the PO, and they don't call me either.

I: And that one example where you went to the Court for a Guardianship, um, could you speak a little bit about what types of information you were asked or what you were, you were sharing?

P: "Is this patient capable of managing his daily affairs, and making you know, rational, sound decisions? And is he aware of those decisions, or is he you know, speaking from no frontal cortex?" And uh, those were, that was basically the gist of it you know. “Do you think he can manage his money, do you think he's uh, his medical conditions preclude him from you know, managing his life safely? Or do you think somebody else needs to help him with these?”

I: Okay. And for your patients um, that do have some type of justice system involvement, um, could you, have you noticed any other factors that they're dealing with socially in their lives? Aside from either being incarcerated or on probation or parole?

P: Yeah, well housing.

I: Mm-hmm. (affirmative)

P: Afterwards, you know, years afterwards. Um, in terms of their ability to get a lease, to have access to public housing, um, and work you know, those would be the big ones. And then you know, people are of course, can be estranged from their family, uh, which can also make housing a challenge. If they're not getting along with their kids or their spouse. Um, yeah.

I: And what are they dealing with medically?

P: The same thing as everybody else. Um, diabetes, hypertension, you know, mental health, depression, bipolar, addiction and alcohol, s-, you know, tobacco abuse. Um, you know, and then any other ... You know, you know, take your pick for addiction. Um, any other, you know, a number of other substance abuse disorders. Um, but yeah, I mean, they, I've, they are dealing with the same medical conditions as you know, somebody who's, whose not incarcerated. But they're also like trying to find a job, or trying to f-, figure out how to, how to you know, make rent, or um, yeah, look for a spot to live or whatever.

I: And are there any resources or services that you wish you had available to refer your patients to, that just aren't available to you?

P: Well I think if people had housing uh, after they were let out, and I think if there was kind of a seamless um, system that allowed for medications to be uh, you know, prescribed and picked up after 30 days, by a provider somewhere, that would be helpful. Um, so that they're not getting you know, 30 days of their insulin and then they're go-, you know, just nothing you know, and they're not, they have to reapply for health insurance, and it's just a mess. Um, I think that if they were stably housed, I have the im-, I don't know but I have the impression that there would be less chaos in their life shortly after, in the months after being released. Um, those two would be nice.

Um, you know, I think probably depending on the, on the presence of, of an opioid use disorder, I mean, there's just tons of data that say, “The chance of somebody overdosing is you know, exponentially higher after being released from uh, prison and/or jail.” So you know, if there is any kind of support network in terms of either counseling or you know, suboxone management, or methadone maintenance, or uh, you know, access to, access to, to uh, treatment, you know, that would be nice. You know, for everybody, not just you know, to this uh, jail in this uh, you know, County or in this state. You know, take this on as an initiative or not, you know.

I: Mm-hmm. (affirmative) And going back to a point that you made earlier just to clarify, about getting medications, um, I think 30 days afterwards. Were you saying, did you mean that making medications available to people when they're leaving jail or prison, or something else?

P: Leaving and then having it continue, you know.

I: Okay.

P: Like for example, I just saw a guy in clinic about two weeks ago, who had been, he had been uh, in prison, in um, I think he said he was in St. Cloud, because he violated his, his parole, after having been in prison a few years before that. Or a year, it was a year before that. Well he's, this guy he's young, but he's got diabetes, he's got hypertension, he's had a stroke, he's got a blood clot for which he takes a blood thinner indefinitely. Um, so he's not, he's on like kind of a lot of pills, and some of them are higher impact than others. Like insulin, like if you don't get it you can get kind of, you can get sick quick. Um, so at discharge, he told me that he was given you know, a month’s worth of medication.

And he had been without his meds for about two weeks because he couldn't get in to see me, and it had been six weeks since had been released or two months or something like that. So um, you know, that, that could potentially be a problem. You know, if you don't take your blood pressure pills for a couple weeks, that's fine. But you know, if you don't take your insulin and you’re type one diabetic for a couple weeks, that can be a big deal. And that's a hospitalization, that's, it could be major illness. That's a lot of money that you know, we uh, you know, use to care for somebody that could have been used otherwise you know.

I: Now thinking broadly, are there any additional changes to healthcare delivery that you would suggest, to better meet the needs of patients who have some type of justice system involvement?

P: Yeah, I mean, I, that, that's it.

I: Mm-hmm. (affirmative)

P: I think a transition from incarceration to the clinic, with you know, insurance in place. I don't care if they have insurance or not, I just want them to get their insulin. Except when they show up and they're like, “I can't, I don't have $50.00 to pay for the insulin,” then they don't get it, you know. So I think if there were insurance that were in place as they were walking out the door, and you know, their medications in place. Granted, not everybody who's incarcerated needs to see the doctor afterwards. You know, I'm ju-, I guess I just see the people that, you know, have chronic conditions or they come into the hospital for some other reason. Um, but I think you know, both insurance and uh, I guess access to uh, medical care if they need it, would be awesome. You know, and housing would be nice too, but ...

I: Um, so thank you again for your time today. Before I wrap up, is there anything that I didn't ask today that you'd like to add?

P: Oh, not that I can think of. Uh, I guess I'm just curious what uh, yeah, what the outcomes will be.

I: Mm-hmm. (affirmative)

P: And ...

I: Yeah. So if you're definitely interested, we'll-

P: What's the difference between parole and probation?

I: (Laughs) Uh, well I actually, I, before I answer that question-

P: Yeah.

I: I do have one question that I was thinking about. And going back to the social history, and you're asking your patients about um, their employment and housing situations, are there resources in, within the clinics that you have? So once, if you find out for instance, someone is having trouble finding housing, are there resources available to you to help connect them?

P: It's just crappy. Yeah I mean, I can say, "I can get the social worker and the best that we can do in the clinic is to help them get to the right place to apply for a shelter voucher, to get to whatever shelter has the space that night."

I: Mm-hmm. (affirmative)

P: Um, the p-, and the patients usually already know how to do that, you know. Um, and it, it has no um, it is not a measure of anybody in the clinic's care for them. You know, or like desire that they had like a bed to sleep in and a roof over their head. You know, it's just a crappy situation all the way around, you know. You know, people come to the doctor or come to the hospital with problems that we don't have a fix for. You know, like their, their uh, there's you know, chaos in their life and so they either come to the hospital or they're sent to hospital. And you know, the, you know, what we have they don't need. You know, they need, they need like a social network to help care for them, and you know, they need (long pause) You know (long pause) they need uh, yeah they need some support.

You know, they don't, I mean, I can give them their insulin but it you know, it's, I wish I could give them a place to live you know. Yeah.

I: Hmm, yeah. So thank you again. Um, in terms of probation and parole, so um, probation typically occurs as an alternative to being sentenced to jail or prison. Um, folks who are on probation may have spent some time in jail, and then been sentenced directly to probation. Um, and then parole is for people who are getting early release.

P: Okay.

I: Um, for, from prison. And [County] actually-

P: I suppose that makes sense, now I can sort that out in those words. Yeah.

I: Yeah. [County] doesn't call it parole, they have supervised release-

P: Okay.

I: And intensive supervised release here in [County].

P: Yeah. Okay.

I: Yeah, but, but that's the gist of the difference.

P: Yeah, that makes sense.

I: Yeah.

P: I can, I can come up with that next time I'm around.

I: (Laughs) All right, well thank you again.

P: Totally, thank you.
