# Supplementary material for: Physicians’ perspectives on continuity of care for patients involved in the criminal justice system: A qualitative study
Source: PLoS One. 2021 Jul 14;16(7):e0254578. doi: 10.1371/journal.pone.0254578 (PMC8279398; doi:10.1371/journal.pone.0254578)
Supplement: S2 File — (ZIP) [file pone.0254578.s002.zip › Clean/Participant_6_Audio1_LJ_deidentified.docx]

I: So thank you again for coming in this morning for this interview. Um, like I have said briefly, this project is part of a larger project between us, the university, and [County], and we're examining the intersections between health and community supervision or, that is, probation and parole for individuals. And the goal today is to get an, a better understanding of your perceptions of the criminal justice system and how it may impact some of your patients, if you have experience with that. And I want to begin today by getting a general overview of what you know about the justice system. Um, to start us off, could you tell me what you think of the current state of the criminal justice system here in the U.S.?

P: Uh, that's a broad question. Um, I guess, it's ... um, what do I think of the general state? Um, I'm not even sure where to start with that. Uh, there ... I, I would say, parts of it, I think it's often described as being broken in different ways, although I think it's really challenging to figure out what's ... how to improve it. Um, I think that's where lots of folks struggle. Um, I think, in an ideal world, there would be a better way to, um, identify and determine who would benefit from different types of, um, for lack of a better word, rehabilitation or treatment, whether it be of mental health or substance use. I know since the decline of, um, state-run mental health facilities, the de facto mental health for many people who have severe mental health disorders is actually in the jail and prison system, I think, which is part of the many challenges in many different levels.

P: Um, certainly, I think the rise of, uh, the drug, you know, the, as Nixon described, the drug war, um, has also kind of given rise to, uh, whole lots of folks that may be in prison that may or may not need to be. Uh, that said, um, within that population, there are definitely people that, uh, that probably need some more protection, both from themselves and from the greater public, and the hard part is figuring out who is that and how do we differentiate between those populations. Um, so I think, probably right now, there is a significant degree of over-incarceration. Um, with that, there's some historical trauma that goes back a long ways, um, and so I would say that it's, it's not quite right.

P: There's definitely (laughs) ... it's the, um, prison industrial complex is definitely larger than it ought to be, um, and how we, as a society, want to deal with that, I think, um, well, obviously, it's still the way it is 'cause people are still struggling to figure out what do we do next. Um, so, I guess, for lack of a more specific direction to go in, I'll leave it at that.

I: Okay. And so thanks for that. Next, I'd like to discuss some criminal justice system terminology. Um, could you explain to me what comes to mind when you hear the following terms, um, the first being prison?

P: What do I think of?

I: Mm-hmm (affirmative).

P: Um, long-term incarceration. Um, usually it's not county run, usually it's more state or federal, uh, for people that are, yeah, in long-term incarceration, so it's usually jail for a short term and then prison for a longer term, uh, or depending on the type of the charge, um, that the person has been, uh, found guilty of.

I: And so, um, could you explain again what comes to mind when you hear the term probation?

P: Uh, so probation, in my mind, is the, um ... so it can be one of two ways. Either it, it's a, it's a step-down so you're not incarcerated, as it's people that are in the general community, but are followed and expected to follow certain criterion, um, in order to maintain their status as not being incarcerated. However, if they were to violate one of the criterion that is ... they're being held to, then they would be incarcerated.

I: And how about the term parole?

P: So parole is, um, is kind of the follow-up from ... So oftentimes, you can be put on parole, so like you're discharged oftentimes slightly early from a period of incarceration, um, and then, uh, then you're followed by a parole officer, um, for similar types of settings as, as probation, um, with the same ... if you violate certain criterion, then you may be, um, sent back to an incarceration, usually to prison.

I: Could you tell me a little bit more about how you differentiate parole from probation?

P: I guess I often think about it more as probation is, um, a deference of the sentence, of the incarceration, whereas parole is, uh, is, uh, afterwards is how I generally think about it.

I: And so now I'd like to shift a little bit to your background and education and training. Um, during medical school, did you ever receive any training, whether it was formal training or informal training, on working with justice-involved populations?

P: No.

I: And-

P: Well, I should say, I had like a few patients in the hospital who were transferred there for their medical care from prison.

I: Mm-hmm (affirmative).

P: That was it.

I: Is there any, like thinking back, is there any training that you think would've been beneficial to you during that time period?

P: Uh (long pause) probably not during medical school. I think it'd be more post-graduate education-

I: Mm-hmm (affirmative).

P: ... where it'd be more helpful, 'cause depending on what track you go down, then that's where a lot of your clinical training would become applied.

I: And so did you receive any training on this during your residency at all?

P: No, not that I can think of.

I: Mm-hmm (affirmative).

P: And then I would say that it probably would've been helpful.

I: And what do you think would've been helpful during that time period?

P: Um, just an overview of kind of how people would interface with the health care system.

I: Mm-hmm (affirmative).

P: Um, and it's, to a certain degree, it's hard 'cause even then you don't necessarily need to know anything about the criminal justice system in order to do direct patient care. Um, I think it's more the context in which you may or may not interface with certain populations. Um, and so I think, in most common conceptions, in order to learn how to practice medicine, there is no need to know anything about the criminal justice system. I think it's more when you talk about population health management, not patient care, that it becomes more helpful to understand more about the criminal justice system. But I would argue that for patient care specifically, how it's currently defined, then, no, you don't need it. But I think if you're starting to shift and think about population health and how do you manage people on a larger context beyond just their presenting complaint, then it's much more helpful-

I: Mm-hmm (affirmative).

P: ... to know about the criminal justice system. And so I think in the ... I personally would've appreciated that more, um, however, I can appreciate why people don't think it's necessarily a high yield topic in residency. But, personally, I would say that that's more helpful, partic-, especially if you're thinking about doing, um, primary care or if you have any semblance of interest in taking care of people in, um, underserved situations, whether it be in a rural or urban context, I think then it becomes much more helpful to think about the criminal justice system.

I: Did you complete a fellowship at all during your training?

P: I did. I did a fellowship in addiction medicine.

I: Mm-hmm (affirmative). And so, during this fellowship, again, I'm going to ask you a similar question, did you receive any training at that point?

P: I did not.

I: And is there anything that you think would've been helpful during that experience?

P: Oh, I actually take that back. Um, it was informal curriculum.

I: Okay.

P: So, no, I did not. There was no formal curriculum at all on the criminal justice system, um. And I would say, to go back one question for during residency, I had a very brief interface of a, in a self-designed month long rotation that, on addiction medicine, where then, um, more in the juvenile justice system, um. I had set up with a couple folks to, to work, um, one in, in the [juvenile detention center], so I, I learned more about, from a juvenile justice system, kind of levels of security associated with different types of education, given that they're both mandates, um, for how the state has set up with different counties, how did it structure that. Um, so I'd say there's a very brief informal curriculum that I had sought out specifically around-

I: Mm-hmm (affirmative).

P: ... because of the involvement with addiction medicine. And, that said, there was nothing during my time in residency that it was associated with the adult criminal justice system. Um, and then, during fellowship in addiction medicine, um, again, informal curriculum only, um, where there's more like research related to risk of overdose, um, post-discharge or post-release. But in terms of how that actually interfaced on the front end of how people got in, um, to a criminal justice setting, there really was no discussion points on that, um. Usually it was more related to if the person wants you to communicate with their parole officer, then make sure to get a signed consent (laughs), but it was never a discussion of who and how could you leverage that person and what context would that person be able to work with you and/or your patient to improve their outcome.

I: Mm-hmm (affirmative). And so now thinking about your current place of employment and past pa-, places of employment, did you, again, receive any on-the-job-training around this topic at all?

P: Um, to a certain degree, um, again, not really any like formal curriculum, but it was just kind of a ... you start figuring it out a little bit more. Um, again, even in my comments on parole and probation, it's kind of how I think about it, but in my ... if I, if you would tell me like make up a test question, would I feel comfortable testing, no, I wouldn't 'cause I don't know it know it. It's kind of what I've figured out or tried to like draw conclusions from based on my work as a primary care provider for adults, um, in a high risk population where there is oftentimes a, a history or a ongoing kind of looming incarceration or immediate post-dis-, post-release, so there's more discussion about that. Um, so you kind of picked it up on the fly, so to speak.

P: I have a lot more patients in my addiction medicine clinic that have charges, um, that they're either facing or immediate release or have parole officers associated with them. Um, so I'd say there's been more kind of on the fly, and I work with a counselor who's ... works in the drug court, so I'm more familiar with that, but I wouldn't say it's ... there's never been formal lear- ... I'm interfacing more with people for whom that's a part of their life.

I: And just to clarify, are you, are you saying you're learning more from your patients themselves?

P: Um, I would agree with that.

I: Yeah. And in your day-to-day visits with patients, do you ever ask them about their current or past criminal justice system involvement?

P: Uh, depending on the circumstance, um, not totally routinely, 'cause it's ... legal charges used to be one of the diagnostic criteria for, uh, an addiction. Um, that is no longer the case because that had lots of racial and, and socioeconomic biases inherent into it and so that was removed as a diagnostic criterion. So it often comes up, but I don't necessarily speak directly to that or ask kind of in-depth questions as to where they are in the state. It's that, oftentimes, it comes up as their parole officer needs to know or "I don't want you" or "I do want you to speak with him or her," uh, and so it ends up becoming a bigger deal.

P: And, subsequently, then people that have been more stable on medication ... some of them, I've definitely had the experience where now people then get caught and are charged and so then they're worried about the medications they're on because they may or may not be able to continue them should they be incarcerated. And so then it begs a whole different set of questions about what's coming and not necessarily the details of the charges 'cause they don't want that to change our, the nature of our relationship, but it does change the, um, ability and, and the planning, uh, for me to continue or adapt their therapy.

I: Could you provide an example of perhaps how you may adapt what medications you're giving if someone is worried about that?

P: Sure. So, um, for instance, um, people who have been stable on a medication for ... The best practice currently for, uh, people who have an opiate use disorder is one of two medications, either methadone or buprenorphine. Um, methadone is, has to be given through these kind of formalized programs, um, where you oftentimes dose six days a week. I'll spare you the details, but oftentimes the withdrawal from methadone is more harsh than it is from buprenorphine. So oftentimes people with a history of being on methado-, methadone, um, who are at risk or have a history of incarceration adamantly, refuse to be on methadone, um, or avoid it at all costs because they're worried that, if they do picked up and get incarcerated again, then they would have to come off it again, um, which is really, really uncomfortable and sometimes you can have withdrawal symptoms for several weeks, um, which most people want to avoid.

P: So even though they know that it would keep them off of whatever opioid that they're misusing, the withdrawal from, say, heroin or fentanyl is, is, while it's, um, intense, it's short and so people would rather go through a short, intense rather than like a drawn out, month long withdrawal, um, in their experience. And so most people then, even if nec- ... the kind of support available from a methadone program and perhaps even the dose of methadone would be more appropriate for them, they avoid that at all costs.

P: And so then it's trying to help people either transition out of a methadone program onto buprenorphine or, if they're not particularly stable on buprenorphine and it would be advantageous from a therapeutic standpoint to increase the structure, which would usually mean shifting them to a methadone program. Um, patients or I will avoid that because they may have gotten picked up and they know that incarceration's a, a real option, um, or is impending for them, in which case, then it's trying to taper them off, even though it's not the appropriate medical care for them, um, but because they're not going to be able to get it while they're incarcerated regardless. Then, in order to make that transition easier for the patient, then, uh, either I or they will, will request to be tapered down to a much lower or off of their medication.

I: That's interesting. Um, for some other patients, um, I know you said that you don't usually ask directly, but is ... are there instances where you would or where you have?

P: Uh, usually it's more around, um, like if they're ... if somebody comes in and says, you know, "I got ... I have a court date coming up," usually that begs the question, then what are we looking at for questions like I was bringing up. If they're looking at, you know, something that's likely a probation misdemeanor, um, or like a fifth degree possession or like even a fourth degree possession, it's ... usually there's not going to be a significant incarceration time, um, but it'll be probation associated with it or I'll ask what's their history. If they've already gotten previous felonies, the likelihood that they're going to be incarcerated versus being on probation is much higher, and so that kind of changes the trajectory of the conversation in terms of ... or, or at least it would ... it, it helps me understand why they would make choices they would, the way they would.

P: Um, sometimes it, it wouldn't otherwise make any sense why they're trying to avoid something that therapeutically makes a lot of sense, or I might push and create a harder contingency if they tried to avoid it, even if they had, um, unexpected or, or, um, aberrant drug use. Um, but if they were facing incarceration in like two months, then it's a lot harder to say you should escalate your dose and go into this more controlled program when you know that in two months that, in their mind, why plan for the future because, "There is no future, I'm going to be incarcerated for X number of months or years." And so I think that where's it's ... those are the cases where I would ask more, not necessarily about the specificity of the charge, but about what they're looking at, um, and what contingencies they've been offered by their, um, their defender or the prosecutor.

I: And are there any risks or challenges that you see in terms of talking about this with your patients?

P: Yeah. Um, I think, um, most people, I, I think, um, it's just a part of their life, so it's not a huge deal. So I, I think, in general, most people haven't had too many problems talking about it. I think what I ... why I avoid bringing it up in the very first interview is that sometimes there's a lot of stigma associated with incarceration. People are very wary, I've found, not all, but there's definitely, I would say, a significant minority of people that, um, that feel, as soon as you're asking the question, that means that you're judging them differently. Um, and I, I want to make sure that they don't feel that judgment and stigma coming from the clinic, that if it comes up, then it's a, it's just a piece of the story, but it's not something that I'm going to focus on.

P: And so, oftentimes, that's why I don't necessarily bring it up in the very first visit, but it'll come up as, uh, you know, something is changing in their life or, you know, when you think about these therapeutic options, you know, which option and for what reason are you picking that option, and oftentimes then legal consequences or legal kind of prospects start coming up in terms of how the patient thinks about what's coming forward and how they want to plan for their treatment.

I: In addition to that, are there any other benefits that you see to discussing this topic with your patients?

P: Um, and I guess the one kind of ... with that question, a little bit of an addendum to the previous.

I: Mm-hmm (affirmative).

P: Um, I always bring up a federal statute. There's a federal statute that makes substance use treatment records or any records with somebody who holds themself to be an addiction professional to a higher, uh, security standard then general medical records. And so I specify that for people because oftentimes, if the courts, uh, subpoena medical records, they will not get any of your substance use treatment records. That's a different standard, so I will point that out to people and ask if that ... I don't always ask it explicitly, but oftentimes, when I bring that up, then people will say, "Well, I do want you to share with my parole officer that I'm doing well," or "I, I am seeking treatment because I want to make sure that, my attorney said that, if I seek treatment proactively, then like the charge is less likely to be as severe." And so kind of all those contingencies often come up and so it's kind of, uh, an indirect way that I try to provoke that, um, the patient to solicit that or to offer that information, um, because it can definitely change how things are going.

P: Um, and in that vein, to your, to your latter question, then oftentimes the question becomes, uh, what records do you want to be released, um, is it all urine drug tests, is it how do you want this to go, um, because the federal statute, the 42 CFR Part 2, the way that it's written is that you can specify what types of records are released from a substance abuse professional or somebody who holds them that to be an addiction professional. And so around that, then oftentimes people will say, "Well, I definitely don't want you to talk to my PO," or "I do want ... like I do (laughs) or I definitely, I definitely do or I definitely do not want you to talk to my PO, um, in order to communicate how well things are going or that I'm really trying," or what have you, um, so it's a mix, but, um, but, yeah, sometimes people do specifically ask me to interface with their parole officer and be in more close communication.

I: Mm-hmm (affirmative). So zooming out a little bit, could you tell me a bit about your overall patient population that you see on a day-to-day basis?

P: Sure. So, um, currently, I, currently, I'm only practicing in, um, the addiction medicine clinic, so everybody that I, is, I'm seeing is either being evaluated for or has a known substance use disorder, um, primarily that's, uh, opiate related, however alcohol and other stimulants is probably close behind. Um, cannabis use disorder is also super common, although often it's difficult to say what's a cannabis use disorder versus just cannabis use. Um, so, yeah, substance use and all of its consequences are all over the place.

I: Mm-hmm (affirmative).

P: That's my day-to-day.

I: Could you tell me a bit about the average income levels of the patients that you're seeing?

P: Uh, I definitely think we have a large majority of people on, uh, medical assistance, so generally low income. That said, we've, um, got professionals. We've got lawyers, um, uh, a couple nurses, um, too in the clinics, but it's, it can be pretty variable, uh, but I would say, generally speaking, it's pretty low income.

I: And what about the disability status of your patients?

P: Um, I'm assuming, when you say disability status, you mean like social security disability?

I: Mm-hmm (affirmative).

P: Uh-

I: And then if ... however, I know some people have varying definitions of what they would define as a disability-

P: Yeah.

I: ... so also your own-

P: Yeah.

I: ... perceptions on that too.

P: Um, so it's ... I would say a fair number of people, a minority, but a fair number of people have social security disability. I've been asked to fill out a few forms speaking to someone's disability status, um, although not many. Um, I think it's always interesting because the way that disability is currently defined, it asks for physical disability, then, uh, psychiatric disability, I don't remember if it's like category C or D, then it says, um, "Does this person have a, a chemical health problem? Um, yes, no. If they were to stop using, would their disability be gone? Yes, no. Do they have a treatment plan? Are they following a treatment plan?" There's like one other question. I can't remember off the top of my head, but it's always kind of an interesting definition that, um, addiction unto itself is not considered a psychiatric ... um, it's not really a physical disability, um, or there's another one that's for developmental or learning disabilities. That's, that's C, and I think D is chemical health.

P: But it says like if this was just suddenly magically better, would they still have a problem? But, uh, isn't that the nature of like most disabled ability conditions? Um, so it's just magically gone and they wouldn't be disabled, true statement. Um, so I think that's, uh ... So depending on how you want to define that, either many or not many of patients would have a disability, um. Uh, as to how I define it, um, it's hard to define independent of all the social consequences from it 'cause usually people are looking for financial reimbursement associated with the definition of disability and it's the current reality. So, I think, independent of this, that societal association with disability, I think people definitely do have a disability when they have an addictive, um, disorder because their decision making is, by definition, skewed, um, for a variety of different reasons, uh, which will impair your ability to perform in a wide variety of situations. Um, and I think we're still trying to figure out, as a society, what's the right consequence for that, um.

P: So I think does it, does, does addiction, uh, disable people? Uh, yes (laughs). Uh, what to do about it and, and what's the right consequence, if people should get like an SSDI, it's hard to tell sometimes. I think we're ... I don't know what the right consequence would be for different types of disability, um. Yeah, I'm not sure.

I: So thinking about your patients who may come from racial or ethnic minority populations, have you noticed any particular challenges or barriers that they faced?

P: In terms of?

I: Care, accessing care.

P: Um, uh (long pause) well, I think there's an ongoing struggle with how to define that, um. Yeah, that's a complicated question.

I: Mm-hmm (affirmative).

P: Um, yeah, I mean, there's a, there's a whole lot ... that's more than an hour (laughing). Um, 'cause if you're talking ... I mean, yeah, there are so many different ways to approach that. Um, yes is the simple answer (laughs), um (long pause) So specific to addiction care, I think it's, um ... [inaudible 00:30:38] just going into like all the different layers that you could approach that question to, um, yes, there's a lot you can go into-

I: Mm-hmm (affirmative).

P: ... and you ... anything from, um, the neighborhoods people are coming in from, the, the resources of their neighborhoods, um, historical context in terms of like how does that play out since ... especially in ... if I'm talking just about substance use and addiction, um, kind of the st-, structural inequities, instances of isolation, um, historical trauma, all build into that, um, in terms of community identities, um.

P: Uh, right now, we're currently struggling with, um, one statewide program we run in tele-mentoring and tele-education. Um, there is calls for ... to have minority-specific echos, um, kind of these tele-education programs, um, which I both understand in one realm, yet whenever we've had people, uh, from these minority communities in the larger group, oftentimes somebody from the larger group is speaking and uses some term or describes a patient or ... in a way that the minority is uncomfortable with. So the minority then tries to reach out and correct that thought process.

P: And sometimes there's, sometimes there's both very beneficial and sometimes there's, um, a recoil from the presenter who may or may not understand the full depth of it or doesn't want to have the larger conversation in that time period, um, which then oftentimes there's a response that's negative, which, in my mind, is exactly the reason why everybody needs to be on the same conference call and which oftentimes is interpreted by the minority as the exact reason why they need to have their own and not be part of the larger population conversation, which inherently they're always going to be interfacing with the larger population, um, specifically around like Native Americans. Um, you can't have care discu-, discussions that are specific just to the reservations or the urban Native population because inherently they're reaching out and getting lots of care from people that are not on the reservation or just in that subset.

P: And I think the same can be held true for many different minority populations, particularly ones that have a strong cultural identity that's, um, distinct from kind of the larger media, um, presence and how people expect things. Um, and I'm using the large generalities just 'cause there's so much that we could go into depth, but (laughs) for the purposes of this (laughs)-

I: Mm-hmm (affirmative).

P: ... discussion, I'm just using very vague terms, um, but I think it's a constant tension, um, both in access, in terms of even the dynamic of the ... my and the cohort of providers in my, my group, in terms of where they come from, what they understand and don't understand from, um, our patients, depending on their past and backgrounds, what people ... what some people may understand and, and assume is, is like a trauma situation when, in fact, that's just somebody's general life and that's just what they realize, whether or not there's chronic developmental trauma inherent in that or maybe that's just what people expect as normative. Um, it's really hard to tell, but people kind of layer on expectations and assumptions in that and so there's ... And then there's access issues in terms of where are clinics and what types of clinics are available to you, depending on your language, um, cultural-economic situation. So there's lots and lots of disparities.

P: I think there's, there is no traditional addiction treatment program for someone who speaks Spanish. In fact, I don’t think there's any residential treatment programs for anyone that speaks anything but English, um, so if you have a substance use disorder, and you have unstable housing or housing that's high risk for relapse, then there is no option for you, um, so that's an inherent access problem.

I: Mm-hmm (affirmative).

P: Um, and, even then, I think there's only one program for people who speak Spanish. I don't think there's any for Somali, um, any ... pick your, um, pick your African dialect, um, or your cultural background. There's nothing out there. So if you have an indication to escalate your intensity of treatment, there, there is no escalation of treatment. You're, you're done (laughs). You get what we can offer you through the interpreter and what we have in our clinic, that's it, um, which is a big barrier.

I: Thank you for that. Um, thinking now about specifically patients who are involved in the justice system in some way, are these folks referred to your care or is it a mix of ... How are folks finding you?

P: A mix, you can either refer or they call in off the street.

I: Okay, I see. And how would you, I guess, on average or if you had to estimate a percentage, how many are referred versus how many are coming to you off the street?

P: Um, 40/60, maybe 50/50-

I: Mm-hmm (affirmative).

P: ... a large portion off the street, just seeking care.

I: And then you talked a bit about, um, speaking with parole and probation officers. Could you speak a little bit more about, again, some of the types of conversations you're having and what information is shared?

P: Um, usually that's in the interest of people wanting to share their, um, usually urine, but whatever body fluid, drug testing results to, to try to prove to their PO that they're doing well. Um, usually, I've rarely ever, if ever (long pause) I think we might have had one parole officer who reached out to us asking for a collateral information. It's almost always a one-way street, where they're rarely ever seeking additional information outside of their own interview with the patient, so it's usually the patient wanting us to become more involved in that process. It's rarely ever the PO reaching out and saying, "I want more information," to guide their own process.

I: Let's see. And from the court side, are they ever asking or requesting information from you?

P: Never, which is somewhat surprising.

I: Mm-hmm (affirmative).

P: I know ... so separate from me and my clinic, there is, um, one of the alcohol and drug counselors in our division, who works specifically with the methadone or the opiate treatment program, the 245G program, um, he is, uh, contracted by the county to be one of the alcohol and drug counselors representing, um, care within the drug court. And so that's kind of a slightly separate yet very distinct part of the criminal justice system that, I think, has been very successful, and so, obviously, he's a lot more integral to the outcomes for those patients, um, but I think he only carries a caseload of like 30. So he speaks to the care for more people than that when he's in the meetings from the drug court, but, I think, to his specific caseload, it's not huge. And he's the only one, so there's nobody that he like refers to other counselors.

P: Um, I know there's, uh, counselors within the opiate treatment program that have patients that have usually some sort of parole or probation involvement, um, but are not from that part of the drug court speaking. He's just directly involved in the drug court.

I: I see. And aside from possible justice system involvement or for your patients that do have some sort of involvement, what else have you noticed that they're dealing with socially in their lives?

P: Well, another huge question, um, chaos (laughs), a lot of chaos usually. Um, yeah, anything from young kids, pregnancies, sometimes older kids, um, kids' problematic behavior, "What am I supposed to do? This is how I cope. Um, I think I'm hiding it from my family and my kids. My kid's doing X, Y, or Z, so I don't know, but they can't get into it." Um, you've got partners or friends or other loved ones that are using or have mental health problems. Um, uh, I've got people with other really sick family members that they're trying to take care of and they perceive their substance use as part of the way that they cope with caregiving. Um, uh, lots of financial challenges, just "How do I get by?"

P: Um, one person who's got, uh, a very small child with a severe developmental disability, so "How do I cope with that?", the nursing care, trying to balance ... trying to get work, but can't work too much, 'cause then he'll work too much and then he got all of his, uh, financial assistance dropped for a few months, which negatively impacted the care for his developmentally disabled child. So then he's trying to figure out, "How do I do that?", trying to figure out substance use and it's to that. It's really problematic. His partner is ... drinks alcohol, which is a whole messy situation, um, lots of deaths in the family, sometimes for medical reasons, sometimes for overdose. Um, I've had a number of people that have died ... that their partners or close friends have died and that was the reason they came to seek care, since all the emotional grief and/or more significant mental health troubles that come from that. Um, so lots of financial, emotional, uh, structural, like societally structural, um, complications.

P: Um, housing is oftentimes a, a major issue. People that are ... I got one woman who is homeless, living with her daughter and son-in-law. Son-in-law is an abusive alcoholic, um, so finally she got her Section 8 for independent housing, but then her voucher is the only ... she had to move to a different city in order to get placed in, uh, independent housing unfortunately. And so she was sober for a year and a half amidst all the abuse and the homelessness, a little over. And then the guy next door to the new house, who happened to have free time, helped her move in and befriended her. It turns out he's a dealer, so now she's back on meth. Um, sounds like she's probably starting to turn tricks for him, um, all in the last like three, four months, yet she doesn't want to give up her independent housing because she struggled with that for a year and a half, but she's starting to feel her life starting to slide away again. So that kind of stuff is constant.

I: And, in addition to their addiction, are there other medical issues that they're dealing with?

P: Oftentimes. Um, on the complicated side, we've got people with, uh, recurring joint replacements. We have two guys with three, at least two, with avascular necrosis with one of their joints, so they've got constant severe chronic pain, um, for a very good reason. Um, we have people with varying states of control of, of diabetes and associated neuropathies, um, people with multiple types of traumatic brain injury and kind of sequelae from that, trying out how do we manage that. Some people with epilepsy and drug-drug interactions to sometimes complicate it, people with HIV or AIDS dealing with that, um, so for a variety of different medical consequences, some acute, some chronic.

I: And so thinking broadly, are there any changes to how we deliver health care that you would suggest to better meet the needs of these justice-involved patients?

P: Um (long pause) yeah, I mean, the short answer, from an addiction standpoint, is one thing that we know clearly, um, is that people, you know, upon release, the overdose and, and mortality rate from particularly opioid use is crazy high, um, and, right now, initiation of any medication, either during the course of their incarceration or prepar-, in preparation for discharge is, is essentially nil. And so the overdose rate is still markedly, markedly high in even the first two weeks after release, so trying to figure out how do you either maintain or dose, say, buprenorphine, maybe methadone, in prison. It seems like, conceptually, like a good idea. I know there's lots of operational challenges in that in terms of diversion and other issues. That said, even if it's just in terms of discharge planning, uh, there's a lot of room for improvement.

P: Um, uh, both in terms of like the pure medical risk of overdose as well as how do you actually integrate people into society, um, 'cause often times it's in the sense of isolation and loss combined with a previous history of substance use disorder. The, the chance of relapse is extraordinary, um, or without other kind of coordinated supports for that, like the risk of success being defined as, um, no return to use or minimal return to use and the stabilization is, is really low, um, given the current ... so it may not even be necessarily a coordination with the medical system and it's a lot of coordination of care with other social supports as well as the medical system, um, in part.

P: I think it would be beneficial. I think having probation or parole reach out closer. I think, right now, um, I both understand in some ways the, the theory behind 42 CFR Part 2, meaning that the federal statute that requires and mandates anybody that holds themself to be an addiction professional to secure their records, um, beyond HIPAA and all other securities. I think I get the conceptual background to that around stigma, however, I think, right now, the stigma's probably propagated more because of it, as opposed to, um, in spite of it, um, and I think the absence of any communication around people that have substance use then just propagates the problem because everybody fears it, nobody wants to seek care, um, and it handcuffs me and my ability to work with anybody that calls their primary care doc, says, "Hey, is this person getting help?" The primary care doctor can't say 'cause I can't communicate with the primary care provider.

P: Um, or if, you know, the PO were to call me ... you know, if they call me and I don't have a signed release on record, then I can't even acknowledge that that call ... I can't call back. So then the perception is that any addiction clinic working with the criminal justice system is they don't care 'cause "They never call me back," when it's really federal law preventing me from actually coordinating care for my patient, um, which is maddening. So I think part of it's the federal statute preventing me from coordinating care. Part of it, I think, is on the, the jail or prison side and post-discharge planning. Um, in an ideal world, although I appreciate the concerns about diversion and other risks is, is continuing people on an agonist therapy while they're in pa-, while they're incarcerated, uh, particularly for pregnant women, but people in general, I think, can really help their mental health 'cause people will go to desperate lengths, um, if they're in severe withdrawal, um, which causes lots of problems while they're incarcerated as well, uh. There's a few.

I: So I know we're running short on time. Um, thank you again for your time today. Before I officially wrap up, is there anything that I didn't touch on today that you think would be important to add?

P: No. Thank you.

I: No? All right. Yeah. So thanks again. Um, we're going to be continuing to interview physicians here, at [health care clinic] and [health care clinic] and, once we're done and start analyzing and writing up data, would you be interested in seeing any reports or publications that come out of this?

P: Sure.

I: Sure. Okay. Great. So I'll-
