# Supplementary material for: Physicians’ perspectives on continuity of care for patients involved in the criminal justice system: A qualitative study
Source: PLoS One. 2021 Jul 14;16(7):e0254578. doi: 10.1371/journal.pone.0254578 (PMC8279398; doi:10.1371/journal.pone.0254578)
Supplement: S2 File — (ZIP) [file pone.0254578.s002.zip › Clean/Participant_7_Audio1_LJ_deidentified.docx]

I: Alright. So thank you again for agreeing to participate. Um, this interview is part of a larger project between us here at [health system] as well as the [University] and [County], um, where we're examining the intersections between health and being on community supervision, so that's probation or parole. And the goal of the interview today is to get an understanding of your perceptions and knowledge around the criminal justice system and any experiences you've had with treating patients with criminal justice system involvement.

And I'd like to begin by getting a general overview of what you know about the criminal justice system. So, to start us off, could you tell me a bit about what you think about the current state of the criminal justice system here in the U.S.

P: Um, and is your question, just to be clear, is your question the state of it like how it's going, or like how it works?

I: Uh, both.

P: Um, well uh, I work here at [health system], so um, I see a lot of patients that interface with the criminal justice system. We're obviously aware that there's an intersection between some illnesses, um, that can be medically treated but that sometimes lead to um, criminal conviction. So um, examples that I would see frequently in my practice would be situations where someone has a drug addiction that leads to um, to, you know, um, incarceration related to um, use of drugs, selling drugs. Um, we also see the uh, intersection between um, substance use and violence, and uh, both patients being victims of violence or um, we also see, you know um, people who suffer from like food insecurity or homelessness, other things that can lead to ... or alcohol, that lead to um, intersection with the criminal justice system in different ways.

Um, my personal feeling is that obviously, a lot of people are punished criminally for um, medical problems, so an example being with addiction. Obviously there's not ... There's a huge problem with substance use and addiction, some of which is the fault of healthcare providers, um, and it then results in people being incarcerated and not treated.

And so um, my personal feeling is there's a lot of um, inequity in terms of how the criminal justice system is um, enforced, and how it actually treats a medical problem with a different type of, an expensive and punitive solution. So obviously, if someone has a drug addiction problem, being incarcerated is um, a form of treatment, but not a very effective form of it. So ... Um, so, and I also do feel that the criminal justice system has problems with institutional racism that's been perpetuated over a long period of time, that's been based on things like sentencing guidelines, um, um, things like, you know, how different people are treated by the criminal justice system in terms of how they're evaluated for crimes, how they're sentenced for crimes, how they're treated fairly for that.

So um, I think there's a lot of good people who work in the criminal justice system who are well intentioned individuals, but are also kind of working for it in a situation where they're ... It's kind of, they don't have good solutions to their ... The scope of that they can do is fairly limited. So, they ... A good person could work at the jail or in a prison system, but they're not able to, within that role, they're not the person who gets to decide how someone's treated. Or a good person could be a prosecutor or a judge and so forth, but they're limited by, for example, mandatory sentencing guidelines, things like that.

I: And so next I'd like to talk about some criminal justice system terminology. Could you explain to me what comes to mind when you hear the following terms, and I'll go through a list, and the first is prison.

P: So, um, so um, I'm not ... I don't have a strong background in knowing this. Um, I think of prison as a longer term incarceration. So just, um, that after someone has been sentenced to a crime, that they would go to a ... rather than their being held more temporarily, that prison would be for a longer term incarceration. And then, are you asking, do ... what are my associations with the word, or like, just what does it technically mean?

I: Both, Yeah, I'm interested in hearing both.

P: Um, well I mean, sometimes people use different terms in our job too, like someone's sent to the =workhouse= or something like that, which sometimes is used to mean prison, but um, the people I work with, I also work with trainees, like residents or medical students, so sometimes there's a little more confusion over the distinction between like jail or prison, of what's the workhouse and so forth. So um, yeah. So I think of prison just as being more long term incarceration. And I think, but I'm not certain, that this means that someone's been sentenced rather than just uh, being held while they're awaiting trial for crimes.

I: So um, you mentioned jail, so what comes to mind when you hear that term?

P: Um, so jail I think of as, just can be either where someone is, you know, kind of um, either, you know, brought to jail for like, there's a suspicion of a crime where the, they perhaps have not yet been sentenced, um, but there's kind of a presumption that there's a possible um, some ... They're going to have a charge placed against them. So sometimes people go to jail, um, and then a decision is made not to uh, prosecute them. So they're held for a period of time, and they're either released from jail to later be, have a court date and so forth. Um, but I think of jail as a shorter term.

So, like at the County jail here, that someone would be in the jail is not a long term thing, but that it would be, um, it would be a shorter term like that they're brought to the jail while they're awaiting kind of a longer term plan, be that they're awaiting something to happen, and sometimes they're released from jail based on different factors of like how ... What's the nature of the crime, and whether or not they're very, like, even like, are they a flight risk to leave before the ... before they would undergo sentencing for a crime and so forth, or a trial, that part.

I: Um, and so you mentioned um, helping some of your trainees distinguish between like the workhouse and maybe some other facilities. Could you tell me a little bit more about that, and how you make that distinction?

P: Um, well we, you know, I mean we actually aren't always privy to, you know, what the nature of ... So we sometimes have a person come from a facility, and they might have, um, they were either ... Um, so here's just a few examples. So someone's like picked up by police for something. Like let's say they were um, they were driving while intoxicated, or they were thought, to be more accurate, they were thought to be driving while intoxicated, and then, that when they're in a squad car, someone says like, the person says like, I'm having trouble breathing.

So you know, before they can ... The jail isn't, uh, although there's medical services, it's not a ... can’t acutely evaluate someone for asthma. So they might be brought to us to evaluate. Um, sometimes we get a sense of like how uh, if it's higher or lower risk based on whether or not an officer comes with them and accompanies them, and whether or not they stay with them. So sometimes when someone's released from our facility ... I don't work in the emergency department. I work in the hospital or the clinic. So sometimes they will just give us some window into what their status is. Whereas if someone comes from ... They might also come from a facility where they're accompanied the whole time. So perhaps they are in jail, and they are brought to the emergency room, and they come into the hospital where they have an officer who is at their room the whole time. And then when they're done they're, um, they go back with the officer.

So I think the trainees' areas of confusion over, are sometimes over like what our role is in terms of sharing information, um, with uh, with police in a broad sense of the term. Like, the officer who's there, be that someone from jail or prison. Um, and, what that's ... whether or not, you know, what we might be doing to ... how we would let them know like when they're leaving the hospital or what the nature of it is.

I mean, typically I think trainees just don't always have lot of exposure to know, like ... Someone might've ... We might have evidence of something that's criminal. For example, like their urine has cocaine in it. But we don't typically like share that information with like ... that's not ... what we're being asked to provide is like medical providers to an officer. Something like that. Um, and I think the distinction between like jail and prison isn't always like what a trainee would understand, saying it as a person who doesn't ... I'm not sure my definition is exactly correct.

So, sometimes people don't necessarily know beyond saying like, this person is dressed in orange, and like they're ... Or in, you know, some cases even more, you know, like they have, they're more closely guarded or something like that. So, I think the trainees' confusion is just like, what happens to them afterwards might be a little unclear. And then it's a little unclear sometimes if, for example, the person was picked up while intoxicated. Does it mean if the officer leaves, like are they still being tried, are they still being like served as like they have to appear in court? Or, is it like all ... Does that mean like the charges are, there aren’t charges against them. That's kind of ... 'Cause we don't see the interface on the tail end of like what happens from there.

Like does that mean they're going to later be um, uh, brought to court and sent to an area where they're incarcerated, or, um ... And it can be a little confusing as to the fact that like we're obviously sometimes privy to the, to something that's against the law. But we're usually, we're typically not asked to provide any information to officers and so forth beyond a ... Sometimes officers will ask us like when is this person, like when's their anticipated time to leave the hospital? And then with most patients, we would share that information with the person. But sometimes for someone who ... Occasionally we get the messaging that like, if there's a particular concern about flight risk for transportation, that we shouldn't share with a patient, like, you're going to be discharged from the hospital.

Whereas, if it were my family member, they would say like, “hey you know, tomorrow ...” a family member of mine that wasn't facing like a criminal situation-

I: Mm-hmm (affirmative).

P: Um, we'd say, “hey, tomorrow we think you'll be able to go home from your breathing problem.” We might not share that information with a patient who's there with an officer, because sometimes we've been, not consistently, but sometimes educated that like the day of discharge, if they're being transported, they don't want the person to know, like, in case there's some question of flight risk or insecurity for the person around the time of transportation.

I: And so, could you tell me what comes to mind when you hear the term probation? So shifting a little bit.

P: Um, so uh, probation. So uh, I think that probation just brings to mind that someone has like, either been ... had a potential for, like was charged with a crime, and that the punishment for the crime was that they're serving a probationary period where they're under like ... they, to see if they commit another crime during that period. So, under probation, let's say um, if they committed another crime that they would then be charged and have ... face a potentially heavier sentence, versus, if they passed through the probationary period, they would get uh, they would, you know, not be incarcerated at that time.

I: And then finally, what comes to mind when you hear the word parole?

P: Um, so parole would be when someone's released from incarceration, and they, they would be also kind of like, not to use the same word, they're under like a type of probation where like on parole, they ... They had a sentence, and when they were ... They had a parole hearing where like, they try to evaluate if someone was sentenced to three years of incarceration, after a year-and-a-half, they could say they'd be eligible for a parole hearing, and they would state their case before a, a judge, I assume, or some type of panel.

And then the panel would deem like, okay, well this is a reasonable um, economic decision to say this person could be released on parole, with the caveat that um, while on parole, the person has the ... you know, could have, because their sentenced was shortened to go on parole, they would have some potential to be kind of looped back into the criminal justice system, based on um, uh, inability to be monitored. Like, they have to be in certain contact with their parole officer.

So we might see a patient who says like, "I have to let my parole officer know I'm here," or something like that. Or, they have to undergo some type of testing, like my presumption, not knowing the system, is that you know, they might have to undergo, let's say, drug monitoring or some other type of thing. Or, they might even have like parole like, someone wears a anklet, and is kind of like, has to be within a certain range, or they have to check in, like, during that time.

I: Could you tell me a little bit more about how you distinguish between probation and parole.

P: Um, I, I assume, this is like my own level of knowledge is that when on probation, um, I sort of think like it's, it's, that the parole happens after a period of incarceration where you've ... you are let out of incarceration and then are on parole. So like you have the increased monitoring or some sort of period, and so forth. And being on probation could be where you weren't actually like, had a long term incarceration, but you were sentenced to like 90 days of probation where like you're being um ... You may or may not be monitored for that time, but if you do in fact get picked up for a crime during that period, you then are saying like, you were on kind of like a, um, on a watch list of something to saying hey, you know, you could ... You're at risk for incarceration when you're on this probationary period. And if you are charged with a crime again over that time, that you'll face increased scrutiny, is that ... That's kind of my sense.

I: Thanks. So I'd like to um, shift a little bit to your background in education and training. Um, during medical school, did you ever receive any training, whether it was formal or informal, on working with the justice involved populations?

P: Um, I, I can't ... I'm not certain, so I'll just say like, I don't remember getting a formal like, didactics about it. I did happen to, um have a few people who I worked with at a ... I volunteered at a homeless shelter that ran a clinic in [city name] where I went to medical school, and one of our clinic preceptors was a doctor who worked in the criminal justice system. So I do remember hearing about it kind of tangentially from this woman. So, um, but I ... And the [hospital] kind of the equivalent of [local hospital] in [city name], had a ward that was like a ward for inmates.

So you went ... During medical school I would see patients inside this ward where they have like double locked doors, and you went through almost like a psychiatric ward is here, where you go through a door, and then the door closes. You call a phone, the door is opened. You know, people are locked in the ward. Um, and you ... You know, so you had some exposure. I would say it was exposure. Um, I may have had formal didactics of like someone teaching me something. Um, I can't remember that.

I: Mm-hmm (affirmative). And so this um, the ward you said you worked in, was this an experience that all the medical students got there?

P: No, it's just if you happened to like, for your third year of medical school you, you do kind of like a little sampling of different areas so instead of being like in high school where you do like social studies and English, whatever. You do two months doing like OBGYN, and then two months of doing pediatrics, and two months of surgery, just to kind of do the major specialties, the idea being you get some learning about it, and you get a sampling of like, do I want to be a surgeon or a pediatrician?

And so if you happen to do it at the University Hospital, kind of the equivalent of the [local University], you wouldn't get that exposure. If you did it at the County Hospital which was [hospital name] you did get that um, you got that experience, um, yeah.

I: And then, in terms of residency, did you receive any training, again, whether that was formal or informal on working with justice involved populations?

P: I cannot remember any formal training on the topic.

I: Were there any informal experiences that come to mind?

P: Yeah. So um all ... I'm in internal medicine, so we, all our residents work in the Emergency Department for a period of time. So you see a lot of people who are brought in by police. Um, and they might be monitored while there, and some of them are monitored, you know, more closely. Like, there was a um, like I saw patients who had been shot, for example. And then like there's clear ... At that time, of course, like if someone is shot and they're gravely injured, they're not a risk to leave the hospital, or so forth. But if they are, um, you know, but if they aren't severely injured, there's some question of like, um, there might be increased monitoring that I might say like, oh we're actually ... People are kind of on heightened alert. Like there's a concern.

On rare occasion I remember someone saying, you know like, we're worried that this person is gang affiliated, and that someone might come to the hospital. And then um, I've had several patients during residency training that were, um, that were under some level of incarceration so that they were, they were guarded. In severe cases they were locked to the bed. You know, like they were handcuffed to a bed. Um, they were also monitored outside the room, or different patients outside the room, inside the room, so forth. So.

I: And, if you completed a fellowship, did you receive any training them?

P: Um, I didn't complete a fellowship so. Yeah.

I: Okay. And then, how about at any like, past place of employment or here at [health system], did you ever receive any formal or informal training around this?

P: Um, I, I work with [doctor name], so he and I work in clinic practice together. I have gone to a noon talk for one hour that he gave about his research in general, um, that was more about um ... It was more about the more uh, 30,000 foot view research on like where people end up, and related to insurance and um, what happens when people leave the criminal justice system, or how they often don't really get too far from the criminal justice system.

And um ... But I didn't, as a faculty member, um, I've only practiced at [health system], so outside of like a tiny bit of moonlighting, I don't remember getting any like other faculty development about the treatment of prisoners. And then I do take call where I'm ... I don't work at the jail, but I do take phone calls. So our clinic covers the [county jail]. So I field the phone calls for the County Jail where we previously had a contract with the workhouse, um, and used to get calls about patients who were both at the workhouse or jail.

Um, so that's my own experience. And then now I get calls of just saying like someone's in the jail, and there's a medical issue that we need to address over the phone. Not, I don't do clinic in the jail.

I: Okay. And thinking back, like over your, you know, med school, residency and then here, um, is there any training along the way that you wish you would have had around this?

P: Um, there's really, like the best type of training would probably be like, at the time and place of contact. You know, we're asked, like any job, to learn like a variety of skills. So if someone told me like how to use the fax machine, it would be like a useful information, but it wouldn't be that useful if I learned it now and then had to like fax something in March or something.

I: Mm-hmm (affirmative).

P: You know like, as an analogy, if I did get ... I may have received some didactics on this, or like teaching, but I think the challenge being like, what I might have learned would have been most useful like when treating a patient who is in a situation that there's kind of a question of like someone take ... A lot of our model is kind of like an apprenticeship. Like a resident works with me. We're seeing the same patients. So most of the teaching would be like, they're seeing the patient. What can I teach them at the point of contact with that.

Um, I cer ... I personally believe that most teaching that's done like that's far removed from the experience isn't gonna be like, very useful. So, while in general, all knowledge is like, good for me, I would find it most useful ... I, um, I think the general information about like, uh, some terminology may, may have been useful um, but I think that that terminology, if it were delivered to a medical student in a lecture format, would be almost certainly useless, um, because that person, it they're then trying to apply that knowledge a year-and-a-half later, they're not going be able to recall the knowledge in an effective way, unless it's really vivid, regardless of like, who's a great presenter, they've got like an amazing PowerPoint.

I: And so during your day to day visits with your patients, do you ever ask them about their current or past involvement with the justice system?

P: Um, it often is volunteered by a patient, um, to say like often people are seeking care from our clinic when they're released from incarceration, and basically they're often left with very like fragmented care. So they often are like, someone was given a period of time to ... Sometimes people are released to like transitional housing or something. So like, let's say they have an alcohol related conviction and they're released to like a halfway house, and at the halfway house they're brought in, and they have a limited period of time, or like, their medications were prescribed for 30 days, and then we're trying to like gather together that.

I would say I don't routinely ask about it. I think it's a little tricky insofar as like, patients want to have their privacy respected, and they also are like wary of saying like, what's my reason for wanting to know. So I do ask about things that are related to, um, criminal justice. So for example, like we ask if people are, um, are safe in their home, or have been victims of violence. Um, we do ask about guns in the home. Um, we might ask about, um, you know, like drug use that's illegal drug use, like buying drugs and so forth. Um, but usually, um, I wouldn't say I routinely ask people about that.

And when they have a history of like a criminal conviction that's been volunteered, like I just got out of prison for 10 years, um, I don't routinely ask like, what were you in prison for, something like that.

I: And when patients do volunteer that information to you, um, how does that inform your care or your treatment plan for them?

P: Um, well, if they're recently incarcerated for a drug related issue, um, we know that it's a particularly like vulnerable and high risk period. So, if it was like um, for certain things, like for, um, for opiates, for example, someone would be like a very high risk of death after being incarcerated if they've like, you know, used opiates or, um, in the broader sense of the term like Fentanyl and heroin, and there was a risk that they would relapse and could have an overdose, in that period.

So, it would inform us insofar as like I might be more um, wary to try to figure out if there was some way to get the person into treatment for this if they're not. Um, If they've been, you know, incarcerated for um, I think our goal is to provide some close follow-up for people knowing that they, you know, if someone's in transitional housing and so forth, they're often trying to piece together getting like some care, but perhaps not adequate care for multiple conditions. So what we do see is people leaving prison who have been incarcerated for eight years, often they come saying like, "I have, you know, eight things that I want to address," some of which were addressed in some way in prison, uh, but some things are off limits for treatment.

So some, uh, uh, prison care is also like privately contracted. And um, and this gets into my own personal beliefs. But I think obviously like, you, our goal is to do like the right thing, not the cheapest thing. So often someone who's like incarcerated in like a privately run prison might have fairly substandard care for like a ... what a common condition might be.

So they're given prescriptions for like things like blood pressure medication, but they might have a fairly inadequate evaluation, or for like how their diabetes is doing or so forth, or how their pain is being managed. And that's actually tricky too because you know, some prisons won't allow ... You know, nobody is prescribed certain medications, so.

I: Are there any benefits that you see to talking to patients about their criminal justice involvement?

P: Um, well I think it's important to know like, what people are struggling with, and it obviously informs like what their, what their ability is to do a certain type of care plan. So, as an example, like if some is coming out of incarceration and has no income, um, I can say it's a good idea to take like eight medications, but it's just not gonna happen if they're paying for these medications. So um, it's important to know like, um, what their, uh, as it relates to their financial status, their housing and so forth. Like all of these are related.

So I don't necessarily know, you know, what my ... What I would say ... My own feeling of my own role is unclear to say like, if the person was convicted for um, uh, sexual assault, I don't know like, what my role might be, beyond saying if this person is um, they're still, they're still worthy of care, and they're um ... And I would still say that they're um, even if I personally believe that this was like, the person did a bad thing in a more moral sense, it wouldn't inform like my decisions about like, what sort of treatment they should be provided with.

So I also think that my own knowledge is that, you know, if someone was incarcerated for murder, it's ... and they were released to me and seen as a patient, um, I would hope to not offer any type of different treatment insofar as that. I could see it helps me to understand like, what their challenges were, particularly around, um, substance use and treatable conditions that we have, and it also helps inform the risk of other things.

Like obviously there's a high incidence of people who have mental health problems that are incarcerated in part because of the mental health problem. So, they can't have their schizophrenia well treated, and this ... and their ... and the solution to their behavioral problems is incarceration rather than mental health treatment. So as result when we're seeing them, it's important to know like, what sort of challenges they might have, and to say, how can we help them or connect them with care.

I: And are there any risks or challenges that you see to asking your patients about their criminal justice involvement?

P: I think the risks are just that um, a person might feel that, feel uh, concern about the confidentiality of the information, and like the kind of therapeutic relationship with the provider. Um, so um, I, I could feel that asking about it could, could be problematic insofar as someone might either feel that they're gonna be treated adversely like well, if I have a history of addiction, you'll treat my pain less seriously, because you'll think I'm seeking pain medication rather than truly in need of help.

Um, I could see someone also feeling like that it's a breach of boundaries insofar as I would want to know, um, someone would usually be willing to tell me if they're homeless. Um, but if they had been convicted of something, um, there's a larger social stigma around, um, um, even though the reason for conviction might be due to a medical problem, um, that's still like being uh, convicted of something. Obviously isn't something we hear about, as like people, you know, don't share this information as freely.

So I would only worry that like, it's kind of ... Um, the risk just being that the person would have of fear that this information is used in an adversarial way, either by a provider, mine or otherwise, and bias against someone who has a criminal history, or that this information, you know, that I would treat them differently, or that it's a boundary that this person may or not feel that like, it's my business to know, like why they were convicted, or something like that.

I: So now I'd like to know a little bit more about your overall patient population. Could you give me some descriptions or like some characteristics of the patients that you see on a day to day basis?

P: Sure. So we have um ... So I um, I work with just me. I work with residents and students in the hospital and the clinic. Um, I don't have my own panel of patients, so I don't have like P's patients that I follow exclusively, so I see them with residents. So I'm helping them learn about their patients. So there are some patients I've seen for um, 10 years, but um ... And I know them a little bit. Like they know me and have seen me, but in the context of working with residents.

Um, the demographically, um, we have um, we have uh, a lot of patients on government insurance, so we have Medicare patients and that, who ... we don't have a large amount of elderly patients on Medicare, but some who are on Medicare because of a disability, and then a large percentage of Medicaid patients. And um, we probably have a larger mix of um, just ethnic background patients. So, I mean it's Minnesota, so there are a lot of Caucasian people. But then, a mix of African American and Hispanic or Latino patients and some other immigrant patients. So we have a fair amount of East African patients, some Southeast Asian patients. Um, other West Africans. Um, those are kind of the main groups.

Um, most of the people in our clinic are fairly, um, complex medically, so they have a lot of medical problems. So we don't have a lot of well 28-year-olds who comes to clinic, and we also don't have a lot of well 65-year-olds who come to clinic. So a lot of the people who use our clinic and services are using it in part because they are a ... They're an underserved community in the sense of like an immigrant community, or impoverished, so forth. So that's ... Is that enough info, or-

I: Yeah. Could you tell me a bit more about your patients that come from racial and ethnic minority backgrounds, and have you noticed any particular challenges or barriers that these patients face?

P: Specifically related to criminal justice, or just in general?

I: In general and related to criminal justice system.

P: Um, sure. So um, just, and say again, so just about like ethnic background and so forth. So I think one is just, you know, people who are immigrants often have difficulty just knowing like what is the healthcare system and what is it offering, and like what are the ways to interface with it. Um, some people are, you know, have ... We have a, a large percentage of clinic patients that have a language barrier, so that creates some challenge. There's the cultural norms that are different between different um, immigrant communities.

So, um, while on the one hand, like you could generalize about, like we have a large Somali population or Ethiopian population. There, a given individual may or may not have the same ... You know, they're part of a group that generally has ... Some things might be different. Like for example, a lot of our Somali patients are Muslim, so they might have some cultural beliefs about um, certain medications or medical practices, or things that are nonmedical. I think as far as ethnic groups, um, I think there's um ... We, you know, we have some, you know, underserved minority communities that I think are understandably distrustful of medical care because of um, past and current medical disparities and how people are treated.

So I think a lot of our patients are wary of how they are treated because of how they look or their past history, be that criminal background, or poverty or they're unlike me in some way. So I think people, understandably, have been treated poorly in the past, and also then are distrustful of like, doctors' motivation, or distrustful that they're receiving the same care that others are receiving. And um, and also then, as a result, you know, may or may not have the same ... And their level of education, just the background.

I mean, just to state kind of the obvious, I mean, to be a doctor you have to go to school for a while, and then you're often working with people who have a lower level of literacy. They're literally speaking, they can't read, or um, couldn't read the medication bottles, or they um ... or for their understanding of a medical problem is more limited. So they don't read on, uh, the [health system] website about like, what their medical condition is, and what the treatments are, but are just trying to, um manage the condition as best they can with a limited understanding.

I: And for patients who do have justice system involvement in particular, um, you've already mentioned some examples, but could you share a little bit more if there are other examples that you haven't shared already about what the experience is like for you as a provider?

P: Um, well I think it's challenging in that, you know, someone, I think just understandably like, could be ... There's a ... There's a mix of feelings someone might have about it. So I think that while in, um, in general you might have feelings about like, everyone should be treated equal, you know, it is just as a person, like if someone is like um, aggressive and like locked to a bed, you might have your own like, you know ... Um, it might be scary, or it might be like unclear like, is this person threatening to me.

So I think providers can feel necessarily a little bit of like um, harder time developing like some sort of therapeutic relationship, um, within that. I don't personally feel like, you know, someone with a criminal justice history is deserving of any less than the same care as someone who doesn't. But I think um, but I think it is natural, um, although not ideal, that someone has harder time connecting. Just in the same way I might have built a strong connection with a Somali woman, it, I think people have an easier time, myself included, connecting with some who, um, on a superficial level is like them.

You know, like it's easier to, say, to spend more time in the room with someone ... If someone's there and they're guarded, a provider might, um, not intentionally, but spend less time to say like well, do I feel comfortable in the room with the person. Like, is it okay to be close with them? Um, I think we have had situations where, you know like, security is called for a patient who's like, verbally abusive. Or you feel like, well, you know, it's harder to then say, or potentially physically abusive, you know, just say, what's my ... Do I feel safe just providing care for this individual.

Um, I don't know, does that answer your question okay?

I: Yeah, yeah. And how do you think justice system involvement may have impacted your patients' access to care?

P: Well um, one is that you know, on the front end, a lot of times people are um, brought into the criminal justice system as the first point of contact for a medical problem. So someone who's um, who's having agitation at a fast food restaurant might be demonstrating evidence of a medical overdose or an untreated psychiatric illness. But the first person who goes out to see the person at the McDonald's isn't a doctor, you know. Like it would be, you know, they'd be incarcerated and so forth. Um, uh, I think that patients have been treated ... Um, tell me your question one more time. I feel like I was getting off track [crosstalk 00:42:06].

I: Oh. (Laughs). How do you think that um, criminal justice system involvement may have impacted your patients' access to care.

P: Oh, so I think for access, I mean I think on the front end, people are often sent to ... They're incarcerated first, when clearly their condition is medical. I think, when they leave incarceration, they're often um, they're often left with limited or fragmented care, and often have like ... often have a lack of direction on like what services that are even available to them, or that um, how their conditions, medical conditions might be treated. And I also think that they have experiences that are very negative with um, with providers either within the criminal justice system or with just a ... You know, they're, they're used to feeling treated less, um, less than humanely. So I think people have a lot of barriers to building trust.

Like normally, if I bring my kids to the pediatrician, I don't assume that the pediatrician would, you know, have anything against them and so forth. But I think people often have a natural sense, which is appropriate, that like the provider is coming from an angle that's not truly in their best interest, because they've often had experiences where they feel, rightly or wrongly, that they were give treatment, or they were incarcerated, or um, sent to prison and so forth for, um unfair reasons.

And so when they're in a situation where they're within an authority figure, being me. They often are wary that this person is like acting from that. And they often, understandably, are wary that that provider has like some understanding of where they're coming from, or like, what their needs are, so.

I: And you spoke a little bit about communicating with the corrections officer, the security personnel. I'd be interested to know more about what type ... Additional information on what information is shared, and in addition to that, whether you're interfacing with probation or parole at all, as well.

P: We usually aren't interfacing with probation or parole. Occasionally we're asked like, if someone's in clinic, and they're just saying like, well I need to contact my parole officer to say like I was in clinic. Occasionally we're asked to provide like some documentation that like this person, you know, was unable to do this for this reason. Or someone will be like incarcer-, uh no, in the hospital, and they'll be like, well I'm supposed to be in court tomorrow and so forth. And we might be asked to provide some documentation.

That documentation, um, even if they're in the hospital. Let's say they had a ... used cocaine, and had a problem related to that. With the communication we would provide is, is stripped of that information. So we would say like, the patient was seen in the hospital for a medical condition. Um, when someone is there with a, you know, security guard or an officer, occasionally we're asked like, is this person like, leaving today or tomorrow? And we would try to provide that information in a kind of like ... Again, like a kind of, stripped of medical details of like why they're here and so forth.

Um, I think on occasion, people are ... there's, there's a little grayness to like what people feel like they're supposed to share. But I think in general, my general practice has been that, you know, um, my job ... And sometimes officers are like, well this person, you know, what's going on with X, Y and Z? And we're just sort of like, well our job is really over here. We're not ... Our goal isn't ... I don't have a, a, a obligation to share information that's a patient's information. No more so than I would for um, uh ... No more so than I would for like a family member who's like, "How's my brother doing?" If the patient didn't want that information shared, we wouldn't instantly just say that. Um, yeah.

I: So I'm a little curious about where the security guard is positioned, in terms of like the physical space. Are they like in the exam room, outside, and how does that work?

P: Yeah, so in the clinic, I mean, it would be more common that like someone would um, be like escorted to clinic, or someone's like ... Often, they might be in the room with them. Um, uh, in the hospital sometimes someone's just like stationed outside the room, and they're just like hanging out there. Like the, there's a, you know, a Sheriff's deputy or someone with a badge who's like hanging out there, and um, sometimes they're in the room.

So, sometimes as far as like, you know, the person's privacy, we don't know in terms of like ... I also don't know the nature of like what their story is. So, sometimes we get ... We get a report, like so we would receive a police report, like this person was picked up by police for acting strangely at the McDonald's, and so like we found some like baggies on them. Or they're like, well it is, it is evidence that we're using for a medical purpose. You know, like saying, like well what is it? Like, because we might have a concern like ... You know, we have examples where like, I treated a patient who like, was being chased by police and ate the baggies.

Where you're like, well what's like the thing? You know, is it, is this a risk to like explode in their stomach and kill them, or like, what's the treatment for it? Um, or, what do we do for that? Or, um ... But then it's challenging if we're like, if the person's in the room with the officer, um, you know, normally we would try to protect a patient's privacy in other ways. So if you were there with your brother, I might ask your brother to say like, “Well I'm going to listen to your sister's heart and breathing. Would you mind stepping out of the room?” And then when I had like a little private space, I could say like, "Is it okay if I share information with your brother," Or something like that.

Otherwise, the person who's incarc ... who has the guard with them in the hospital, um, they don't typically have as much pri ... they don't always have, I won't stay typically. They don't always have as much privacy to say like, to field their questions or concerns, because they have someone there um, who's with them, and we don't always know the nature of like where they're at in the situation. Does it mean they're going to jail? Um, sometimes it's a little more clear that they're like, in prison, or they're like in some longer term situation. But we don't always know, like, what the nature of the crime was to know like is this a,

Is it okay for me, like as a provider, to treat them? And there is, you know, there is harm, like I've ... I mean, I haven't personally been like very harmed, but in a small way, like harmed by a patient, you know like through uh, physical act of violence or something like that, so.

I: And for your patients that do have some type of justice system involvement, what else are you noticing that they're dealing socially?

P: Well I mean, they have a lot against them. So if they're in the system, you know, it's a little less clear. Like, if they're in the system, sometimes it's a question of like, uh, let’s say the call from the jail is a question of just utilization of resources. So if they're in the jail, they might have against them just the fact that like there's a little bit of barrier to do the treatment that you would do otherwise.

So if they have some medical care in the jail, but we have to make a decision whether or not it can be treated there or brought to the hospital.

If they're not in the ... If they have had some contact, like the person says, "I've been released to a halfway house from prison. You know, then they're up against a lot of other stuff. So obviously, there’s, you know, we have a lot of disparities in our country, and so, the people who are, you know, incarcerated are by and large impoverished and also more, you know, more commonly minorities, and so they would have problems with um, getting a job, um, based on their criminal history. They would have problem you know, getting the ... having a larger community. So some of them are, have been incarcerated for a period of time, so they don't have a larger social network. They would have trouble with family support as a result of like, if they're dealing with a medical condition, they might have trouble with where they're at with, with housing. So those are just a few of the challenges, but not all of them, so.

I: And among your justice involved patients, what are you seeing medically with them?

P: Um, well uh, there's you know, there's an over representation of um ... in general, our clinic already is taking care of a lot of people who have mental illness and substance use problems. Um, but I think among those with some contact with the justice system, there's probably an even higher representation of, uh, mental health and substance use problems. Um, and then difficulty with just adhering to a medical plan based on the complexity, along with managing these other factors. I mean, in terms of your hierarchy of needs, you want to make sure like you have a safe place to be, or housing, is probably more important to someone than treating their high blood pressure.

Um, the high blood pressure obviously can cause long term problems, but taking pills for your blood pressure or cholesterol, um, those things are hard. And then we also know that like within the justice system, you know, you can't just sort of tell someone it's ... It would be silly to say, like if you're in jail, you can't just be like, well, you should take like a half hour walk per day for like your blood pressure. So I think they have trouble like managing chronic illness. Um, a lot of times people are managing, you know, substance use or mental health disorders that have been under treated over a long period of time.

Um, people have been victims of violence, um, during that period of time of incarceration, and are managing kind of the aftermath of that, in terms of how that affects their mental health. And then lastly they're trying to figure out, like with the chronic medical condition, nobody is like, worried about where they're sleeping at night, and then worrying more so about their blood pressure. You know, like where they're sleeping, it's just gonna be like, are they safe, that's gonna be like a higher need for them at the time.

So, they might, in theory, um, want to treat their, their diabetes well, but a lot of them have just, just the common chronic medical conditions, but they're often managing multiple things at that time. And we do know that stress relates to chronic medical conditions. So, we know that like long term stress, be that stress related to housing, violence, et cetera, leads to that. And a lot of the people have been victims of violence where they're related to medical problems. Like people have chronic pain because they have bullet fragments in their hip, or things like that. So they've also been victims of violence.

I: And, are there any resources or services that you've noticed that your patients need, but aren't available to them?

P: Well in general, um ... So the big ... Um, there's a lot. I mean, I think in general, um, our, our medical system, you, you know, you get medical care through your job, primarily. So, many people don't have a job, so that's difficult. They have a harder time getting a job. And um, and then the resources that are missing are, we have substance use treatment for a portion, but not most of the patients who are in need of it. Um, we lack housing, that sort of a benefit for ... that would allow people to have more stable living situation. So often, people are um, funneled back into the criminal justice system because there aren't viable alternatives in terms of employment, housing, um, some way to have like a stable life going forward.

I: And so thinking broadly, are there any changes to healthcare delivery that you would suggest to better meet the needs of patients that do have criminal justice system involvement.

P: Um, well we do have like a ... We have a social worker, you know, who works in our clinic. But we don't always know like what the resources are that are available to a given individual. So housing is like an insanely big problem. But we don't, you know, we don't run a series of apartment ... own a bunch of apartment buildings that people could live in. But we also don't know like what program someone might be eligible for. And some of it is, um, and so the…we don't always know, even if there are set services like, where they're at, or whether or not the given patient would be able to access them.

So, you know, sometimes for my job it's always a little, it might be a little unclear like, if someone has [medical assistance provider] as and insurance program, which is not exactly insurance, but it's like sort of a form of coverage, what they would have available to them. So, what's lacking is a little bit of like the coordinator, like, what can coordinate with this patient when they need it, the mental health and substance use services are broadly needed and not available. And, the ability to kind of like coordinate follow-up for them. So some of our patients have a care coordinator who's a nurse, who would work with them to sort of say like, "How do you navigate? How to get to these appointments? Can we help you scheduled them?" You know, how does someone manage all of this, often without some simple things like, you know, you if person doesn't have a telephone, um, it's tricky to figure out how they would schedule appointments. Keep track of a schedule, et cetera.

I: So, thank you again for your time today. Before I wrap everything up, is there anything that I didn't talk about or ask you about today that you think would be important to add?

P: Um, no, I mean I think there's like an amazing unmet need for just individuals, and I think that people in the criminal justice system are, are treated really poorly. Um, and I think that in our health care system at [health system], I think we, we might be more understanding than some, but I still feel like there's, you know, people are also just, most of their care is not related to healthcare. So most of someone's wellbeing is really like not related to me in any way. So, I think someone doing better is mostly related to like being able to get a job, and being able to be integrated into a community, and being able to have housing.

And so while I think the health care as a community could do much better, I also think that like, our community as whole, um, is also where like, most of people's challenges lie insofar as like, most of your health as an individual is not if your doctor is like the most amazing person. It's really that like, you have family that supports you, and that you have food that's healthy to eat, and that you have a job that provides you with meaning and so forth. So I think that people in the criminal justice system often are faced with not only challenges within like the small piece of the pie that's health care, but like largely just with all the other stuff that is challenging, um, for them, so.

I: Thank you again. Um, we're gonna be continuing interviews for the next several months. Um, once we have any reports, peer reviewed publications at some point, would you be interested in receiving those?

P: Sure.

I: Okay. Thank you.
