# Supplementary material for: Physicians’ perspectives on continuity of care for patients involved in the criminal justice system: A qualitative study
Source: PLoS One. 2021 Jul 14;16(7):e0254578. doi: 10.1371/journal.pone.0254578 (PMC8279398; doi:10.1371/journal.pone.0254578)
Supplement: S2 File — (ZIP) [file pone.0254578.s002.zip › Clean/Participant_8_Audio1_LJ_deidentified.docx]

I: Alright. Um, so, thank you again, um, for participating in this. This is a joint partnership between us here at [health system], the [University], and we're also working with [County] on this project, um, to better understand this relationship between health and being on community supervision. So, probation or parole, and criminal justice system involvement more generally.

And, the goal of this interview is to just gain an understanding of your perceptions and knowledge of the criminal justice system, and the experiences you may have had with treating patients with some type of justice system involvement. And I'd like to begin by getting a general overview of what you know about the justice system. Um, so, to start us off, could you tell me what you think about the current state of the criminal justice system here in the US?

P: Um, things are a mess, and since the psychiatric ... I'll put it this way, from ... I'm a psychiatrist and as a psychiatrist, since they've closed down the state hospitals they were supposed to provide other services that these people would go to and they didn't, and now they're all ending up in the jails.

So all my psych patients are ending up at the jail, and they've closed down so many state hospital beds, and they did not make provisions for providing the needs of my patients. So, now they're ending up in the criminal justice system, where they really shouldn't be, but, um, that's where they're getting their clothing and food in the winter and their medicines and that's a mess. And that's my perspective, as far as criminal justice, as far as say domestic assault, and those kind of things.

Um, there's issues with what's going on with the way women are treated, and the justice about what happens ... the disparities between justice for men and women, is, uh, something I see. And, um, and then as far as experience that I've had with it, I worked at the [psychiatric hospital], the [psychiatric hospital name] for 15 years. And that was the civil side of the mentally ill and dangerous patients were there. And then, after leaving that, I've worked at, uh, the [county jail] every Friday for quite a few years. I also worked down at the women's, uh, women's part of the workhouse, the [county workhouse], some. So, that's my experience level.

I: Okay. Thanks. And, next, I'd like to discuss some criminal justice system terminology. Could you explain to me what comes to mind when you hear the following terms that I'll go through, and the first is prison.

P: Prison is where somebody has been convicted of the crime and they go to stay there. Usually, they're crimes of a sentence longer than a year, 'cause people go to the workhouse usually, or just even stay in jail when the sentence is less than a year.

Is that what you meant?

I: Mm-hmm (affirmative), a little bit. Yeah. And then, the next term you already brought up is jail.

P: Oh, yeah. Jail is where people are kept that can't bail out, and sometimes my patients don't even have the $20 to bail out. And so, they end up stuck there for some misdemeanors and sometimes they're too ill to deal with the whole process. But the jail ends up becoming a psych hospital for those people. Um, I ... up on my high horse here for you (laughs).

I: (laughs)

P: Anyhow, so, the jail is usually an option ... there's some option for people to bail out for most people. Um, lot of the people don't have money to bail out. And it can also be no bail if it's something like a murder charge. Um, is that what you meant?

I: Mm-hmm (affirmative). And could tell me a little bit more about how you distinguish between jail and prison?

P: Well, jail is, the jail is detainees, they're not convicted yet. And, so, once somebody's convicted, they're supposed to go the next step, which would be prison. Is usually the way it works. The jail is usually more short-term. And also jail is where they come right into. So the people coming into the jail could be intoxicated, and they have to be searched for weapons and everything. Whereas, by the time you get to prison, you've been sobered up and detoxed, and, uh, people don't usually go right to prison. So, it's a step, jail and then prison, usually.

I think sometimes they can ... people ... if some, if somebody's out on, say, parole and they violate their parole, they might get yanked right back to prison, but I think they always go sit in jail first for the processing of it.

I: And what comes to mind when you hear the term probation?

P: Well, probation is the one that's a little, um, not as ... something about probation is better than being on parole. Parole, you've done something far worse, but yeah, (laughs) that I don't really know the details.

I: Mm-hmm (affirmative).

P: I know guys that have murdered somebody end up on parole, and the people with a lesser crime can be on probation. But they're still both being kinda monitored and helped through the system, least they're supposed to be. Beyond that, I don't know anymore technical legal details.

I: Mm-hmm (affirmative). And so, next I'd like to talk a little bit about your background and education and training. Um, during medical school, did you ever receive any training, whether that was formal or informal, on working with justice involved populations?

P: No. I did not. I went to med school at [medical school name]. I'm originally from [state name], and we didn't have any of that kind of experience there. I, uh, came here to Minnesota, matched here for my residency, and I was always interested in the correctional side of things 'cause part of me always wanted to be a detective. (laughs)

I: (laughs)

P: And so, well, uh, that's sort of how I, sort of, crossed the boundaries.

I: Yeah, so could you talk more about your training during residency?

P: So, in residency, my first year at the time was here at [health system], and then the rest of it was at the [University]. And so, at [health system], we had detainees coming in here that we provide care for. And also, there was, uh, uh, when I went over, then the next two years at the [University] ... this was before the [joint county] Psych program was invented ... when I was at, um, the [University], [doctor name] was over there as an attending. And I went with him down to the [psychiatric hospital] because he worked down there, and I thought it was all cool and got to go hang out, and thought I wanted to do that. So, that's how I found out about that with him.

I: Mm-hmm (affirmative).

P: But I think that's the extent of the residency experience.

I: Okay. And did you complete a fellowship at all?

P: I did a year in consultation liaison psychiatry, but that doesn't count as a fellowship, technically.

I: Okay. Could you tell me a bit about that. Did that involve any type of training related to criminal justice involved populations?

P: No, it didn't have anything to do with criminal justice-

I: Okay,

P: ... it had to do with, um, uh, [doctor name] and [doctor name]. Both were doing consultation liaison psychiatry here. And then, you hang out with them and learn from them specifically-

I: Mm-hmm (affirmative).

P: ... for a year. So, that was a cool opportunity, but it had nothing to do with criminal justice.

I: Okay.

P: I think that's really the extent of my criminal justice experience, was I sort of got thrown into it when I went down to the [psychiatric hospital]-

I: Mm-hmm (affirmative).

P: ... and learned down there.

I: And, so, no training at all here at [health system], or at a former employer?

P: As in criminal justice?

I: Mm-hmm (affirmative).

P: No, uh-uh (negative). No. What do you mean? I'm ... I guess I'm missing something.

I: Yeah, I mean, just any ... in, we're trying to get a sense of what folks have learned about the criminal justice system through their formal-

P: Mmm.

I: ... or even informal types of, like, presentations. Or, like, similar to, like, residency, where you, um, receive some.

P: I chose on my own to go get continuing medical education with the forensic psychiatry specialists.

I: Mm-hmm (affirmative).

P: There's an association. In fact, there're two associations for forensic psychiatry. And because I was interested in it, I went to those conferences-

I: Mm-hmm (affirmative).

P: ... and learned that stuff on my own-

I: Okay.

P: ... 'cause I wanted to, but, um, I didn't ever formally do that fellowship either. Because by that time I had been out and I had expenses, and you'd have to go back and be a fellow to make residency pay for two years. I couldn't afford to do that. Once you've got family expenses, you can't go back for fellowship very easily.

But yeah, I took ... on my own initiative, I went and did a bunch of continuing medical education classes that you could get with, uh, it's the, um, psychiatry AAPL, is what it's called. American, um, Psychiatric Association of Law and something. I can't remember (laughs).

I: Okay.

P: But it's a interesting organization that provides really good conferences and that's where I learned all those things from them.

I: And so during your visits day-to-day with your patients, do you ask them about their current or past involvement with the justice system?

P: Not day-to-day. Um. The jail visits show up in the computer.

I: Mm-hmm (affirmative).

P: And so I know if somebody's been hauled in, and then I can see what it was for. And so sometimes I'll ask them about the relapse they probably had that then landed them in jail, and how they're taking care of themselves and that kind of thing. Um. My mentally ill and dangerous patients, when they come through, they're whole legal history is spelled out along with their case when you accept them. So that's information already provided. And then I would find out if they've, like, the group home would say he hit somebody, he's going back to jail. Or he's refusing his meds and he's going back to [psychiatric hospital], those kind of things. So, a lot of it you have to base it on collateral information.

I: Okay.

P: But, you know, most of my people, if they've ended up back in jail, they're not gonna tell me. They don't say it, I just see it in the computer.

I: Mm-hmm (affirmative).

P: A lot of them are embarrassed or angry or all sorts of things, but, I just talk about it with them.

I: So, could you tell me a little bit more about, like, how you broach that topic with your patients and how do you approach that conversation?

P: Well, say it's somebody who has a substance trouble. And I could see that they were taken into the jail for a new DUI. I ask them, "Did you get to keep your drivers license? How are you doing? How are you functioning in the world? What happened with your job?" And I, we just talk about it and see how it's affecting them. And um, if it's something like a domestic assault, then I do that a little differently, that, you know. Quite often, guys that are domestic assault perpetrators try and blame it on the woman. And I, it's like, "Whoa buddy. No. We're not even going there. It doesn't matter what somebody says, you don't get to hit 'em. No violence. What are you doing to learn different coping techniques?" And then we'll talk about it from that point of view.

Um. My guys that were like from the [psychiatric hospital], one of my guys is this poor sweet soul who's really psychotic and that he, at the time, he was off his meds and on street drugs and he killed his wife, the mother of his kids, and he is miserably guilty about the whole thing to this day, he feels so bad about it. So we talk about things, about how he's doing with taking care of himself, and, and how he's doing with the new relationship, and how that's going, and, if he'll allow himself to be happy in a relationship, and ...

So everybody's different based on their situation.

I: Yeah.

P: Did that answer your question?

I: Mm-hmm (affirmative). Yeah. (laughs) And then, you kinda touched on this, but does having that information, knowing that one of your patients involved with the criminal justice system, does that impact your approach to their treatment at all?

P: Uh, yes, it does. I mean somebody that has trouble with substances, like alcohol, I know I want to avoid giving them benzodiazepines, if at all possible. Um. People that, um, tell me one thing but the record shows another, then I know that I've gotta get collateral information. To keep an eye out for what's documented versus what they're telling me. And then, um, there's people that there's just embarrassment about ending up there, and if they've had a relapse, well, relapse is part of the course of substance troubles, and people end up in the jail for possession. I mean that's a misuse of the criminal justice system, but I can't fix that. So. Is it? [inaudible 00:13:44]

I: [inaudible 00:13:45] And are there any benefits that you see to asking your patients about their justice system involvement?

P: Well, it helps know that like if, say somebody's relapsed and drinking heavily, then that helps me know that they've been denying it all along. And then I see that they were picked up with a DUI, then I know that they weren't telling me, and then that's why their antidepressants weren't working, or their liver enzymes could be up and they're blaming it on something else. So knowing all the aspects of somebody's life helps you understand where they're at.

Like one of my guys who was back today is a fellow that consistently won't take his psych meds and he becomes manic and psychotic and violent. And so he ends up at the jail and he know he's stopped his meds every time he ends up at the jail. That's consistent. He doesn't go to jail unless he's stopped his meds and relapses. So from my point of view, it helps with all those kind of things.

You feel like you're wanting something else (laughs)

I: (laughs) No, uh. My next question is gonna be focused on asking you about, are there any challenges that you see to talking to your patients about this?

P: Well at first, in the beginning, it was uncomfortable, because how to you broach this. But I always just look at 'em like, there but for the grace of God goes me. And so since I could be the one sitting there in that chair, I'm not gonna, it's no place to judge. It's how can I help you turn this around? That's what I'm here for, to help with, not giving you any medicine that's gonna make things worse. To try and help you stay outta trouble and to help you do better in life and have a successful life, that's what I'm here for. And if I don't know about it, I can't help 'em with it. And I certainly don't wanna do something like give 'em a medicine that gets 'em into more trouble. So knowing that you shouldn't give them benzos 'cause that can precipitate a relapse is part of taking good care of people. So I've become more, sort of, just forthright and with no judgment.

I mean like some of the poor women that are in the jail over there for prostitution. They get picked up on prostitution and it's like, this is ridiculous. They don't belong in there. They're people that but for the grace of god, any of us could've ended up in that situation, and putting them in jail isn't helping the situation. But, if you can, if it's because somebody kidnapped 'em and got 'em addicted to drugs and is making them pay their drug debt by trading drugs for sex, those people, if you can break 'em out of that, there should be some other way to break 'em out of that mess rather than jail. But that's our system. That's another broken part of our system.

If I could fix it, I'd do all sorts of cool things. (laughs)

I: (laughs) Well, could you tell me a little bit more about your just, overall patient population? Who are you seeing on a day-to-day basis?

P: Here at [health system], I've got everything from depressed little ol' ladies to people with schizophrenia. And then because of my 15 years at the [psychiatric hospital], they tend to give me the people that are on release from the [psychiatric hospital]. So I follow up more of those people than most people here do. Just because of I'm interested in it and my experience. And then every Friday I work at the jail here at the [county jail]. And other than that, the population's just your typical psych population, except we've got more seriously persistently mentally ill people here, than say, oh something like [community mental health care provider], where you have to have a different kind of insurance and, um. People would have to have insurance to go there. And they don't have a sliding fee scale, and all those kind of things. So they accept a totally different population.

I: Mm-hmm (affirmative). So you mentioned the like, a typical psychiatrist you know, patient population. I don't have very much background in that at all. Can you kinda describe what that means?

P: So, if you work someplace like [community mental health care provider], mostly what they take would be an entire, whole bunch of 15 minute visits with depressed little old ladies or kids on Adderall, and they just, nothing's real complicated. It's a quick one med decision, and get 'em in and out of there, and they'll see 20 people, and everybody's got insurance. Whereas [this health system] population's typically got, ours have serious and persistent mental illness like schizophrenia, bipolar, and they use substances, and they become violent and end up in the criminal justice system, and ... it's a lot more involved, our people here at [health system]. And so everybody knows if you work here or say at [county hospital], that you have these more complicated cases, is what we are interested in that, and that's what we expect to get.

I: Mm-hmm (affirmative).

P: But if you want something where you just whip through a bunch of boring cases, it's all Adderall for kids and you do it every 10 minutes, then you go to a different kind of practice. So people know what you're getting into when you accept a certain job.

I: Mm-hmm (affirmative).

P: So the typical [health system] population of patients is, ours are mostly seriously persistently mentally ill. A lot of 'em are on public assistance. And we have things like food bags here. We've got a social worker that can help people with housing. So it's a totally different feel here than [community mental health care provider] clinic waiting room. I think you missed the waiting room while it was out there full just awhile ago, and there were a whole bunch of people out there all talking really loud. And the, your suburbian housewife doesn't, they're scared in that setting out there. So they don't come here very often.

I: And you mentioned that many are on public assistance. When you say public assistance, what are the thing that you include in that?

P: They've got um, like medical assistance. And then that can be farmed out into ... there's different contracts that people have. Like, so there's [insurance provider] that's not the insurance one, but it's a farmed out medical assistance from the state. There's all sorts of different stuff that people come here with. But it's government stuff, it's not from like insurance from your job.

I: And how would you describe the disability status of your patients?

P: The population here is way more disabled than say a community clinic would be. Say, I'm using [community mental health care provider] as an example, but, um, there was a mentally ill and dangerous guy that I had worked with him at the [psychiatric hospital]. And uh, I had to be out for knee surgery for six weeks, and his case manager decided to take him to the [community mental health care provider], um, which is a different bunch of providers, psychologists and psychiatrists and clinical nurse practitioners. And they wanted him to be seen sooner and faster and stuff. And he got in right away there, but the psychiatrist told him, "This case is too complicated for us. You need a whole team to deal with this guy." That's the kind of patient we're used to dealing with here, whereas there, they wouldn't even accept him. They just looked it as a consult and sent him back.

Also, you know, I think that case manager expected meds to be changed, which you have to get approval from the [psychiatric hospital] medical director to change meds on an MI&D patient. And how they could not know that was beyond me, but, that doesn't have anything to do with corrections.

I: And have you noticed any particular challenges or barriers faced by patients who are from racial or ethnic minority populations?

P: I see that there's a whole lot more people of color in the jail and the white people get bailed out somehow, so something's going on there.

I: Mm-hmm (affirmative).

P: Something really is different. It's just really visible, um, but how it ends up that way or ... I don't know. But as far as the access here, anybody that, um, we're actually not taking new referrals here because we're full and short on providers. So, nothing has anything to do with any ethnicity or anything coming here. But if you mean like people landing in the jail or staying in the jail, definitely the amount of people I see in the jail when I'm asked to see the psych list, they make up a list of people they want me to see, and it's more people of color. Totally, obviously imbalanced. So something's ... I don't know what's going on, but it's clear, something's there. Whether they can't afford to bail out or there's more put there, I don't know.

I: And thinking now about your, in particular your patients that have some type of justice system involvement, when it comes to treating them, how do you think that their involvement with the criminal justice system may have impacted their ability to access and receive healthcare?

P: My people actually end up getting better healthcare when, like say they're homeless and they're out there running around using drugs. A bunch of my guys end up being sobered up in the jail, end up back on their meds, or end up committed and put up to [psychiatric hospital] where their meds are restarted. And so they get the healthcare they need by going that route, which is a shame. But, it actually gets 'em the healthcare they needed. Because a lot of them are out there living in a tent somewhere doing drugs, and became violent, and, and, so it gets 'em into a path of getting some help.

And some of 'em having a parole officer is really beneficial as far as helping 'em get services and getting 'em ... But there's others that I hear stories about, tales of parole officers that are really nasty and don't help people. So I don't know to believe, who to believe on some of those cases.

But um, there's a variety but a lot of my people get the care they need through the route now of the justice system, because the mental health system's failed. There's not enough beds. There's not enough providers. And you end up in jail to get forced the care you needed.

I: And do you get patients that are specifically referred to you? For instance, from maybe a probation officer linked them to you here or a parole officer or something like that.

P: If, when we were accepting patients, I did. But because we're short of providers, we're not taking anybody new.

I: Mm-hmm (affirmative).

P: But yeah, there would be people that like I'd seen 'em in the jail and then they would just come over and I'd keep 'em.

I: Mm-hmm (affirmative).

P: Um, if I got to know 'em over there, over a long period of time, yes, they would sometimes come over here and see me. So we had continuity of care.

I: And do you communicate at all with parole officers or probation officers or the courts at all?

P: They apparently read my notes. Like say when I do notes at the jail, I am told that they wanna read my notes about what I'm saying and seeing, but I don't go talk to 'em or anything.

I: Yeah.

P: I just document psychiatrically what I see and I make my recommendation that this person is forwardly psychotic and needs to be on their anti-psychotic meds. And, and I petition for the Jarvis order. And so we get that all started over there while they're sitting there in the jail when they refuse meds and they need 'em. So we get a lot of commitments and Jarvises started over there while people are there and refusing meds.

I: And so what's a Jarvis?

P: The Jarvis is the State of Minnesota's legal hearing to force anti-psychotic meds on people that refuse 'em. So they usually, you have to specifically list what meds you want to give the person and, um, it's a whole long ... it's too hard to show you. It's a whole bunch of paperwork and you have to do a court hearing-

I: Okay.

P: ... in order to give people the meds they need in this state. Most states have a thing where if you're committed to treatment, then that also covers that you would get the meds that you would need. Say you're committed to [psychiatric hospital], then normally normal states would mean that you also have the right to treat them by committing them to [psychiatric hospital]. Because you assume if you're gonna send 'em to [psychiatric hospital], you can't say, "No, I can't give you any meds." That's stupid. But that's the state of Minnesota. We've got Jarvis proceedings to give meds.

I: And when you say they are reading your notes, who are they? What ...

P: Um, my ... I don't know, technically.

I: Mm-hmm (affirmative).

P: But from talking to my patient, the detainee, afterwards, the attorney for the detainee reads them, and tells the detainee, and then the detainee says, detainee says, "Why did you say that?" So they know and the prosecuting attorney knows, because that goes through the ... There's a chain of communication. And so I hear through the, the grapevine that all of the attorneys and judges and everybody are reading the notes.

Like when somebody's obviously malingering, and I will say that the, that this is blatantly not a real psychosis. Because somebody, we've had several child offenders that they offended against kids. And they would suddenly say, "Oh I'm hearing voices, the voices made me do it." No prior history and they were doing stupid things like claiming a little man was walking on the edge of a book. I was like, ugh, get real, buddy. And, you know, they want to read what I think about those things.

I: And aside from justice system involvement, what else are your justice involved patients dealing with socially?

P: There's the thing if you've got a felony on your record, it affects ability to rent and ability to get a job. And so they have a nightmare trying to get a job and have to start at things like, I was gonna say a dish washing facility, (laughs) car washes. Apparently car washes will take anybody. And so they start there to get a good record of employment and they have to work up way harder than most people. Um, housing is more of a problem. But on the other hand, if they've got a good parole officer, a good probation officer, that can give 'em services and give 'em help.

They've got this cool mental health diversion program here in the state. And the mental health diversion program, and there's also a veteran's diversion program. And those two programs help people get the services that they need and help them stay on the medicines they need to be on. And so those are really beneficial programs. Those are cool. Um, for everybody else, it seems to be sort of just the luck of the draw as who you get and how much help you get and ...

And then you know, some people don't want help and they sabotage everything. So everybody's different.

I: And what about medically?

P: So, the guys in the jail end up getting all sorts of medical stuff offered. They're screened when they come in.

I: Mm-hmm (affirmative).

P: They get sobered up. Um, they get (laughs) regular meals and clothes and moved out of their tents. And uh, so they can end up having some of the first medical care they've had in a long time, for some of them. Other people, that's not applying to, but. So, what I see, the clinical nurse practitioner they're providing is pretty darn good. In fact, some of 'em get more than you get on the outside with insurance. (laughs) So, it can be a little weird.

I: And what are some of the medical conditions that you're typically seeing among justice involved patients?

P: Well they have, like, say abscesses from shooting heroin or tuberculosis from being on the streets and homeless. Um, there's wounds and sores and broken bones. And then there's one dude that killed, I don't know who he killed, but he's got some horrible cancer that he's gotten state of the art treatment and stays there in the jail for this treatment he's getting. And so, he's getting way better treatment than he probably would've gotten if he'd been out there in the community taking care of himself. Because the deputies take him over for his radiation and everybody's there checking on him regularly. If he were at home, he'd be drinking and he wouldn't be taking care of himself that way. But you know, it looks to me like they do a very good job at the [county jail]. I don't know about other places, but ...

I: And what are some of the more common mental health conditions that you're seeing?

P: The most common thing I see at the jail, unfortunately, is guys trying to get me to document something they say to provide a defense for them. And I know that's going on. Um, and then there’s the real genuine people that are really have schizophrenia that stop their meds and become ill and end up locked up there. And so I go see them and start the court process to get 'em committed and get 'em to, back on their meds. Um. Then there's a lot of guys that are saying they have insomnia. They can't sleep there. And the sheriff didn't want us to provide anything for insomnia, so you end up in this kind of ... What do you do? 'Cause you've got this person who's suffering versus the fact that they don't want to treat insomnia there. So then I try and sort out is it anxiety or depression and you can call it depression and anxiety and treat that. (laughs)

I: And so, what are some of the substance use treatment needs that you're seeing?

P: Oh, one of the people heroin, methamphetamine, um, those are the big two right now that are landing people there. A whole lot of meth. People do violent things and become psychotic with methamphetamines. Something about the way they're cooking methamphetamine right now is making more people psychotic. And then when you're paranoid, you do violent things that you think are self-defense. So there's a lot of that.

But then there's also people that are just picked up for possession of a little bit of meth and they land in there too. And then there's alcohol. And then the heroin people, they're usually really poor souls, too, that, um, they have to be cold turkey withdrawn through just a couple days of methamph, err meth, methadone. They give 'em just a couple days methadone taper to come off the heroin. So they're always miserable and we're trying to help them through that.

But yeah, the alcohol, meth, heroin, I think are the big things that I see for substances.

I: Okay. And what about the physical health needs that you're seeing?

P: I don't see physical health stuff. I just work down the hall from the people that do that. I don't see physical health. If there's anything at the jail that's physical health, I shoo 'em down the hall to the right person.

I: Okay.

P: Because they don't want me crossing boundaries. There's such a shortage. There's lots of people that want to be seen. And so physical health's gotta go down the hall and see the physical health people. So they've usually got a two page list of names to try and see each Friday. (laughs)

I: (laughs) And are there any resources or services that your patients need but aren't available to them, that you're seeing?

P: Housing is the ... In the [city name], housing, affordable housing is just an utter nightmare. The rent has gone up so high and the line to get like Section 8 housing is very long. And I have my patients living in tents and crashing on people's couches and living in shelters for a year. It's become ridiculous. I don't know what happened, but it's way worse. So the housing needs.

We also trouble with people being able to afford their meds sometimes. Because they've got some sort of insurance, usually we can fiddle with the meds and change things to where it's affordable for them. But when they have no insurance at all, they have to go through these programs like Walgreens might have a $2 med list and you have to go through that. Um. Sometimes people are embarrassed and don't wanna tell you they don't have any insurance, they just sorta disappear and stop their meds because they had no money for it. And until things go wrong and you hear about it, because of their embarrassment, that's um, they end up off their meds and in trouble back in the hospital. And that can be how you hear about it.

So yeah, the housing's a mess. Affordable meds is bad. Transportation's getting better. Because like [health system] here's doing something where they can get you, um, those drivers that come to your house and get people. Help 'em get to their appointments. So that's doing better. Those are the main things.

I mean some people have trouble getting enough food. We have food bags here. We've got a social worker here. So we can get 'em pointed in the right direction to go get what services are out there. But the main shortage is housing and appointments. There's not enough appointments. There's not enough providers for psychiatry. There's really a severe shortage.

I: So thinking broadly, what changes, or are there any changes to healthcare delivery, that you would suggest to better meet the needs of people who have some type of justice system involvement?

P: Oh it'd be great if we could afford to have more providers there to see people and see 'em more often. But with the shortage of providers there's nobody out there to, you know, it's hard to get anybody to work there. Because think of it, if you can work someplace with some nice little housewives from Suburbia versus some guy who's gonna spit on ya, and try to hit ya, and scream at ya, "you fucking bitch I’m gonna rip your head off." How much of, which are ya gonna choose, unless you're a nut like me? (laughs) You know? Ha. I like adventure and stress and that kind of thing, but most people don't. And so, getting people to be interested in providing there is the tricky, very tricky thing. My wanting to be a detective and being interested in the whole thing from childhood has put a different aspect in the way I view it, but ...

So it's hard to find providers and providing psychiatric care in a jail can be tricky. They did give me the dental office there. So you have this nice big room. It's not like you're in a cell or something. It's pretty cool. They’ve got a whole floor where they have all the medical providers there, and the clinical nurse practitioners. Did that answer your question?

I: Mm-hmm (affirmative). Yeah. So um, thank you again for your time today. Before I wrap up, I just wanted to ask, is there anything that I didn't cover today that you'd like to add?

P: I think I blathered everything I was thinking about.

I: (laughs) And then, are there any other providers here that you'd recommend that we reach out to and interview?

[Censored to protect confidentiality]

I: Um, and like I said, we're gonna continue doing interviews here as well as at [health care clinic].

P: Okay.

I: And, hopefully [health care clinic]. Once we have some reports and things to share at the end of this project, would you be interested in receiving them?

P: Yeah, sure.

I: Okay.

P: Absolutely.

I: Alright. Thank you again.

P: Sure.

I: That's all the questions that I have.

P: All right. So you guys have ...
