# Supplementary material for: Physicians’ perspectives on continuity of care for patients involved in the criminal justice system: A qualitative study
Source: PLoS One. 2021 Jul 14;16(7):e0254578. doi: 10.1371/journal.pone.0254578 (PMC8279398; doi:10.1371/journal.pone.0254578)
Supplement: S2 File — (ZIP) [file pone.0254578.s002.zip › Clean/Participant_9_Audio1_LJ_deidentified.docx]

I: Alright. So, thank you again for taking the time to talk with me today. Um, this interview is part of a larger project with the [University], [health system] and [County], where we're examining the intersections between health and being on probation and parole. And the goal of this interview is to get an understanding of your perceptions and knowledge of the criminal justice system, and any experiences you may have had with treating patients with a history of crim- criminal justice involvement.

And I'd like to begin by getting a general overview of what you know about the criminal justice system. Um, to start us off, could you tell me what you think about the current state of criminal justice practices in the United States?

P: Um, system that needs to be fixed. Um, it's unfair, most likely. It's probably unfair regionally, and sectionally, and according to allocation of our attorneys for, um, people who can't afford it. So, I don't sus-, I suspect there's not equal justice for all. Um, system may still be ... And like it, it's about democracy is not great, but no one's come up with anything better. So, I, I think it works, and gimps along, and is unfair in many other ways. Um, that generality is okay.

I: Mm-hmm (affirmative).

P: Uh, I should say I've worked two years in a prison. You gonna go through that later?

I: No. Feel free to ...

P: So ...

I: Yeah. Talk about it now.

P: ... in the military. So, I worked at the [military prison]. That's the, the big house for the Army and the Air Force, where people convicted of either military or criminal crimes get sent. So, I did that. And I consult with the law school, and I work on deportation cases, things like that.

I: Mm-hmm (affirmative).

P: So I have some, some familiar area. I'm not, I review articles, war journals. I, I'm not a forensic psychiatrist. That's not my specialty. That's it.

I: Okay. Um, and next I'd like to talk about some criminal justice system terminology.

P: Yeah.

I: Um, could you explain to me what comes to mind when you hear the following terms? And I'm gonna go through a list. And the first is prison.

P: Prison? Prison ... the big house. (laughs). Prison is usually a state or federal. I think of it as a penitentiary. Um, usually, um, the ones I'm more familiar with, probably an obsolete model. A, a central rotunda and, uh, cell blocks coming out, uh, from them. Um, that's somewhat, uh, that description would fit, um, I'd say maximum and medium level security. Uh, I know there are, obviously, other minimum security prisons and trustee prisons, stuff like that. But I, I think of it as, uh, I guess, along with Goffman's total institutions, in which, uh, you're under watch and under control 24 hours a day, and you have very little control over, um, when you eat, when you sleep, when you get to do things.

Um, infractions are dealt with swiftly and surely, usually. Um, there may not be much justice in a prison, in terms of, uh, ... if you have a point of view that, or disagree with what’s happened to you. I don't mean why you got in, but what's happening inside the prison. And it's, also, a dangerous place to be in terms of being assaulted by other prisoners. Um, especially if you're, um, in special categories, whatever that may be. Child abuser would be one. Um, sexual crimes, probably, another. Anyway, that's my, uh, sense of prison.

I: Mm-hmm (affirmative).

P: And I think ... I'm probably (laughs) wrong. I ... my, mine's always been at the fed. Federal-level prisons are probably run better than the state-level prisons. I don't know if that's true anymore, but state-level prison would be more vulnerable to budget cuts, um, state-level corruption, not that it can’t be federal and, and so on. If I had to be in one, I'd, I would prefer being in a federal as a prisoner, I mean, than a state level prison.

I: Mm-hmm (affirmative).

P: Despite that, some people do well, they get out somewhere in a trade. Um, some need to be there and others not. Okay.

I: And how about the term jail?

P: Uh, I see jail as a local facility, a lock-up, as opposed to the big house. Jail is I, I see as jurisdictional. Counties have jails, cities have jails. They get pretty big. Um, I go up to [County Jail] to see, um, refugee detainees who are one step away from deportation. So, I, I go into there, and it, it's okay run. Um, if you don't make yourself obnoxious, I think this jailhouse rules. Um, and that's because it was a contract with the feds. There, there's probably four or five hundred people. I think that's unusual. I don't know how much [local county] holds.

So I, I think all local facilities are limited by, by budget, and over-crowding, and things like that. And by whatever training, or the lack of training the staff get. Um, and whatever local, and federal, and national prejudices are going on. Um, not just ethnic or racial, but, also, prejudice about sexual orientation, or mental illness, and things like that.

A, a quick anecdote. When I was at [army base], which was, I'm sorry to say, [year], [year], during the Vietnam War. But, um, we had some psychotic prisoners and we, we, we sent them ... we thought we were sending 'em to, let's say, [city] or [city] to the hospital. We found that they were being locked up there. So when we protested, um, they put a sign up over one of the jail units that said, mental health unit number 13 or something, and that was the extent of what they did differently to take care of psychotic prisoners. So there's jeopardy all over. Okay.

I: Okay. And how do you distinguish between prison and jail?

P: Um, probably initial distinction from the war point of view, um, and I, I suspect it has to do with length of sentence and, and finality of sentence. So you could be arrested and put in jail. You can't be arrested and put in prison. You need to have a trial and be found guilty to be put in prison. And, um, then the, uh, the length of time that you're there would be dependent on a lot of variables.

Um, your access to the public is different and you ... I, I mean, prison is much more controlling. Um, but I, I see jail as, um, that it ... relatively temporary place. Um, can be e-, can be dangerous, but since it's more transient, I, I wouldn't see that gang formations can't be as, as, as solid as well constructed. Um, people don't stay as long, you can't have king of the roost.

On the other hand, I don't know about, from the staff point of view, how much surveillance, how much supervision, um, what they, what jailers have to have credentials in being hired. Um, I assume it's, uh ... they're not social workers. They're law enforcement people. So I don't know what kind of, um, training they have. Uh, what the ethics of the person who runs it is, what the political issues are. Uh, but there should be more access to the public in jail. I could be wrong.

I: And what comes to mind when you hear the term probation?

P: Um, probation ... I always get the two mixed up. Probation and that other thing. Probation means you are released from confinement, uh, and you get a probation officer, and, uh, you're released for a number of reasons. Um, obviously because your, your behavior and, um, contrition in the prison has, has been documented and, uh, you're deemed to no longer be of great risk to the public, so you're released under probationary conditions. Which means you see your probation officer, and if you break the rules of probation, which probably some generic and some particular in each person's case, then you go back and you may serve the full sentence, or have something added to you. So that's my understanding.

Um, uh, you may have to post bond, but I'm not sure, uh, about that. I know that the bond needs to be posted by ... when the, um, deportees, people who are, are ordered removed from the country, but the country isn't ... doesn't have a government that can accept them. So they're ... prisoners like that are, um, uh, set free on bail bond. So they're free and, uh, they have to check in. But on, uh, a bond is required. I don't think probation is an, uh, is required. A bond is required. But I, I don't ... shaky on some of those details.

I: Okay. And how about the term parole?

P: Well that's ... I think that's where, um ... So you're gonna have a probation officer, a parole officer. And I'm not sure of the difference frankly. I, I ... Um, in both cases you're released before your sentence is up. In both cases you have to hang around, you have to check in with your parole officer. Um, parole may be for the lesser offenses, but I don't know. If you know, you can tell me, but ...

I: (Laughs)

P: ... that may not be part of our bargain. I'll ...

I: (Laughs)

P: When I get home I'll ... I’ve wondered about it before, but I've never been curious enough to, to think about it.

I: Mm-hmm (affirmative).

P: Um, parole may be easier to make, whatever that means. But, um ... well, when I go home I'll look it up, ...

I: (Laughs)

P: ... or call a lawyer.

I: (Laughs) Okay. And, so, now I'd like to shift a little bit to your background, and education, and training. Um, during medical school, did you receive any type of training, whether it was formal or informal, on working with justice involved populations?

P: Um, we're talking old history now. Um, I don't recall. I, I ...

I: Mm-hmm (affirmative).

P: There was very good psychiatry. And I think, um, people ... not really. I, I don't remember any formal lecture on justice prison system. I think s-, if someone wanted to, they could have done an elective.

I: Mm-hmm (affirmative).

P: Yeah, and they ... but that would have taken some ferreting out of resources, contacts, names. Um, I don't think in med school was high up on any, anyone's agenda, or, or getting through med school would be.

I: Mm-hmm (affirmative).

P: But I don't wanna demean it. I mean ... but getting through med school ... I mean pursuing things that you're interested in, I don't know that at that early stage. I can't think of people who were, and I can't think of any formal mechanism for it.

I: Do you think that there would have been any training at that time period that would have been helpful to you on this topic?

P: Y-, y, um, yes and no, um, in the sense of a medical school curriculum is enormously crowded, and, uh, there's enough, uh, there's a lot of competition for air time, um, between anatomy and biochemistry. I mean anatomy ... no one were taught the arms and legs. They taught the head and trunk, and the internal organs, seriously, after I finished. So, um, here ... they, they said, well, you know, if you need it, you'll take it as an elective or go to hand surgery. So, in other words, a lot of s-, a lot of the, uh, programs, uh, were cutting hours in order to include other hours as they became important.

So, it was ... I don't think it would've hurt, but it would have been an hour or two lecture at best. I think, I think it might have been interesting. It would have had a, it would have had to be at the expense of taking two hours out from something else. And, maybe, parasitology would have been okay. But, I, I don't know. Um, yeah, I think, yeah, it would have been helpful, of course, but, uh, one has to see if [inaudible 14:35], see the competition ...

I: Mm-hmm (affirmative).

P: ... for classroom hours. Um, tryin' to think now. No, I, I think we once went to, it might have been med school, to a local health, health department for a lecture on, um, sexual transmit, uh, sexually transmitted diseases. I don't know that I got that much. I'm rather impressed by the, the wise guy who giving us the little conference that when he opened the door, he held his suit jacket against the door knob, so as not to have his hand touch a door knob. So and that’s 50 years ago and I remember it, um, I thought he was showing off. But, um, I don't know. Yeah I guess maybe one has to be ready for the program.

But I, you know ... So to come back, bottom line, obviously, anything we get would be helpful. It may have, you know, touched some people more than others, and opened up some po-, possibilities earlier. That's about it.

I: Mm-hmm (affirmative). And how about your time during residency? Did you receive any formal or informal training on criminal justice involved populations at all then?

P: I don't think so. I think it's a guide ... My training was it's ... Well my internship was pediatrics.

I: Mm-hmm (affirmative).

P: And, absolutely, not. Uh, no formal training in criminal justice. Psychiatry, there may have been a few lectures, um, having more to do with the overlap of mental illness and, um, and criminality, and, and hospitalization. Uh, you're talkin' to an old guy now. Um, there really wasn't the, uh, the enormous problem, whi-, which was first beginning in the 70s. My training was in the 60s, uh, in which people were let out of hospitals, sent out of mental hospitals and ended up on heating grates in the street, and homelessness, and, uh, not being well taken care of, despite the, the, the pledge of ... that community psychiatry would take care of people out there. So, a lot of people began ending up in jail.

But that ... my training was before that. Um, so there, there was some awareness of, you know, overlap of criminality and mental health, but, in fact, everyone was given a statistic that the criminal rate of men, people with mental illness is lower than the population. And, um, I think we, probably, had some lectures on forensic psychiatry. Um, maybe the criminally insane. But I don't think we had anything from a, kind of, sociologic point of view that this is the system, and this is how it works, and this is what happens, what doesn't happen if you're mentally ill and end up in a hospital. Or if you're healthy and this is the, kind of, justice you can expect or not expect. I don't think this was the case.

I: Okay. And did you complete a fellowship at all, ...

P: Nope.

I: ... in your training?

P: No. (laughs) I, I had a ...

I: (Laughs)

P: Yeah, I, I'm chief resident for one year. So I did my one-year pediatric internship, three years general psychiatry, residency, state of fourth years chief resident. But I didn't do one in, um, chemical dependency, or counsel liaison. Child, and no ...

I: Hm.

P: ... general psychiatry.

I: Okay. Let's see. And, then, about your ... in terms of thinking about your current or past places of employment. Did you ever receive any training, um, around this topic then?

P: Uh, in residency, um, uh, um, as an elective I went out to state hospitals. I, I ...

I: Oh.

P: It was every Thursday. I was in [city name]. So I'd go out to [ County]. [inaudible 00:18:41]. Um, and had some mobile app with me at a criminal unit there. But that was, kind of, what I arranged for myself.

I: Mm-hmm (affirmative).

P: Um, I would meet with whoever was running it. They were pleased to have a resident come by. Maybe we, we can recruit this guy. Um, afterwards, I, I think there's been occasional lectures by either, either forensic psychiatrists or sociologists. But I don't, I don't think much as a ... I, I, I have to really search ...

I: Mm-hmm (affirmative).

P: ... long and hard to come up with something. Um, okay.

I: All right. And so, now, thinking about your day-to-day visits with your patients, do you ever ask them about their current or past involvement with the justice system?

P: Um, sure. Um, not all. Um, I work two-thirds with refugee immigrant population. And, um, that's, that's, that, that's what [health care clinic] does. And, um, I'm very concerned with, uh, their getting into trouble, especially if they don't have citizenship. So we, we, push very hard. That's not criminal [inaudible 00:20:04] to, to get them citizenship. And sometimes they, they, they just resist. No I'm busy, no I am, am ... I can't do it, I'm doin' this, and, and I don't think people appreciate the, their, the vulnerability they're in. Certainly young men. Young men of color, but, yeah, young men.

Um, so I ask and then, then as we talk about citizenship ... well, with young men in general I ask do you have a police record. That's, probably, usually the way I put it. Have you been arrested?

I: Mm-hmm (affirmative).

P: Um, now not everyone tells me the truth. So some ... I say that, 'cause sometimes as I get to know them better, then some, uh, they'll something that, you, you know, is different than I was told. So, um, my, my own, use a strong word, disdain for, uh, initial questionnaires, except for today, uh, you know, is that people, um, with something to lose, is self-protective, and they'll filter what they want to tell you. Not sure they tell me the truth. I, sometimes, have to say to people, um, I am not the FBI, I am not ICE.

I: Mm-hmm (affirmative).

P: I am ... you know what ICE is?

I: Mm-hmm (affirmative). Mm-hmm (affirmative).

P: I am your doctor. I'm here for you. But, you know, uh, it depends. So I, I try, I go into a criminal history, police history. Uh, but even other things, um, I don't ask 'em. I ... you can't think of all, like, how ... are you back, are you in rear, arrears in your child support payments? I mean that will go in your arrest sheet. So that will be, uh, evidence of moral turpitude, and can add to the list ...

I: Mm-hmm (affirmative).

P: ... of why you got deported. So there's all of these, these things. But in general we ask, and, uh, we will also tell people we can provi- ... know [health care clinic], [health care clinic] has an arrangement with a, a large law firm that does enormous amount of pro bono work, but they don't do criminal work. Okay, they will work on, um, housing, citizenship, uh, rights, benefits, uh, SSI appeals. A whole lot of, a whole range of things, but, ...

I: Mm-hmm (affirmative).

P: ... uh, I think they, you know, they, they are civil lawyers and corporate lawyers, and they put in enormous amount of hours with us. But they don't do criminal work. But they can get us, they can help us out in that sense. So we, we, try to tell 'em, look, if, if you're in trouble, or if you need help in your housing and other stuff, um, then, uh, you know, we can help you. We, we, also, will work sometimes with the, the public defender in a criminal case. So, we, we, we do that, our own pro bono, um, when it, when it comes up. It's not a major focus by any means, ...

I: Mm-hmm (affirmative).

P: ... but ... Well, as you know, uh, when someone becomes homeless, they're risk of being arrested, just on trespassin' and all this kind of business, really goes up. And so they, they need, uh, whatever help. And there are times I'll, I'll, even, see someone who got arrested three years ago, when, you know, I have seen them three years ago and they clearly were manic and had poor judgment. I'll say to them, you know, if this thing is gonna get in the way of your citizenship, uh, we can help you fight it, get it expungeable, without being grandiose. Possibly, if you were interested because ... as far as I know, you were manic at the time you did all those things. Like running naked in the street, or whatever.

I: Mm-hmm (affirmative).

P: Well, and other kinds of trespass. So we have an awareness at the clinic of the population, and their vulnerability, their, oh, difficulty in being represented well. Uh, I just went up to [County] Friday to see someone who was on the airplane at ... was deporting Somalis. I don't know if you remember it. They got as far as Senegal and Senegal wouldn't let them off the airplane, and they sat on the tarmac 20 hours or so, chained, and then they were flown back to Florida. So I have seen two of those. Then they will all work with the lawyers to, um, try and get their green card back, you know, um, permanent resident back. So, we do that at the clinic. And, um, I work with two other psychiatrists. No, four other psychiatrists. I work part-time, so to speak, ...

I: Mm-hmm (affirmative).

P: ... and, four nurse practitioners. And we all do it, um, so we have this awareness of it. That's about it.

I: Yeah. And, so, when you're making the decision to ask versus not ask a patient, what factors into your decision making around that?

P: Implicit bias. (laughs) Um, well in general I, I mean, you know, little old lady, 50 years and older, I'm not gonna ask. I mean, no reason why I would say that, but, I, uh, got other stuff to do with female.

Uh, with younger people, more male than female, with females, uh, it would just be part of it, it. Uh, I try and make it part of just, uh, uh, that you fold the question in, so it's not, “and now we're gonna go to your criminal history.” You know, it's ... or it might be, um, “Where did you live?” Or “what kind of housing did you have? Did you have jobs? Did you get in any trouble with the police? Did you?” ... So it, it would be just folded into, um, uh, an, an interview, an assessment.

In, like, all assessment interviews, you know, a lot of the question ... I like it to walking down a corridor opening doors. If the room's empty, you walk on next. So a lot of the questions don't come up with any yes, and you move onto the next. If there's something there, then you go spend a few minutes and, uh, registering it. Uh, of course, you have to move on. I'm not following a ... I'm not following a paper outline, but I'm following, you know, a mental health line. There's a ...

I: Mm-hmm (affirmative).

P: ... clock up there and that's the reality. And you, kind of see it, and you note it, and you file ... or you spend more time at the moment and, uh, recognize and ... recognizing as I've seen. Um, that not everyone's gonna tell me that much. Working with refugees I work with, um, interpreters and I, I ... and, and we have our own interpreters so that, that's a lot better than having a, you know, um, an ex-, an outside interpreter, what I call parachuting in doing ... And some of them are very good too, but, uh, we get to know the interpreters well, and they appreciate what we do, and we appreciate what they do. So I get some of my cultural training from them. Uh, but they can, also, vouch for us. It's okay. Not that, that, necessarily, works.

So, um, I ask it, but I, I don't, I don't make a special point. And I hear ... I make it a yes and I may keep moving on and then double back to it.

I: Mm-hmm (affirmative).

P: Um, things like, uh, uh, what [inaudible 00:27:50]. You get with young men is, uh, domestic abuse. So I, I may just say what happened, or I may move on and come back to it. Um, so you know. And, um, one has to be careful what, uh, what you write down in notes, other than ... Well, two reasons. You have to be careful, but the, the main reason is, if you start noting it and then a person tells it, then they say, oh my God, you know they're concerned about confidentiality, and it's legitimate.

Um, the, the other reason I'll come back to it if I can. Or else I'll have to remember it on my notes I make that evening. And, uh ...

I: Mm-hmm (affirmative).

P: So I ... it, it works.

I: Let's see. And, then, how does ... once you have that information, how does it inform your care of that patient?

P: Well, it's a piece of it.

I: Mm-hmm (affirmative).

P: Uh, I just ... am, am I talking too long? Are you gonna get through your outline?

I: Yes, I think so. Yeah, we're doing good.

P: Okay. Um, it depends on what happened and what's reported.

I: Mm-hmm (affirmative).

P: And, um, rarely we're gonna have to say to a patient, I need to give you a warning that if you say much more, I'll be obliged to report it. I mean that rarely happens. But, you know, if somebody is talkin' about assaulting, uh, uh ... Not just, uh, I beat someone up in Texas, but, you know, I did this to my girlfriend is [inaudible 00:29:26]. You know, the, the mandated reports go.

I: Mm-hmm (affirmative).

P: Other than that, it just becomes a, a piece of, um, of the assessment. And I would say the main thing is to, um, uh, whatever criminal behavior is told, told to us. And I think, I, I was speaking with all the others too. I don't think I’m different there. Uh, you probably, you try to put it into context of what was going on in your life then, and what was your mental state then, and what was your living situation and, uh ... I, I ask that early on.

I: Hm.

P: Like, who, who are you living with, and who are you living with? And, uh, again, that, that's a question that they say, oh, uh, this guy is the housing inspector, gonna find out too many people are in the apartment. Hey, that's not it. We want to know what your support system is. So, um, we, we would, we would do that and then, um, again, double back to it. Um, I lost my track of where I was ... my anecdote threw me off.

I: (Laughs)

P: Um, ...

I: How does it ...

P: Yeah, yeah what?

I: ... inform your ...

P: Yeah.

I: ... care of the patients.

P: And, and, and, so, uh, and I was ... Yeah, I was talkin' about two contexts or the con- ...

I: Mm-hmm (affirmative).

P: One is that the context is, as much as I can see, of when their criminal behavior occurred and what was going on. And then I try to go back in context to this person's life experience. Um, how much PTSD was there? Who was caught up in a civil war? Who was a boy soldier? Who was assaulted and raped? Who got his parents shot in front of him or her? Uh, and just see how the past might affect present behavior, including violent behavior, aggressive behavior, or going along with the, the crowd behavior.

I: Mm-hmm (affirmative).

P: So, I, I would say that. So it informs. It, again, it's ... I'm not, you know, these are, these are psychiatric clinics. It's, it's not a criminal court clinic. And, um, I, I, I, I try to take the, the long, the long view that, uh, these are things that are gonna take a lot of time to unpack. So, it just, it ... If I just know it's there, and it's jotted down, and I ask people would ... uh, are there more things that we should talk about, about that.

You know, the, the, the events themselves, or what happened, and, you know, people want to tell you things. Uh, I may not be, it may not be my priority, but it's theirs So, if you talk to 'em, “I was on that airplane to Senegal,” and they want to tell how the guard slugged him in the face.

So, you, you have to listen. I don't mean that is unimportant, but, um, it's not the centerpiece of if I can help the person. But I'm ... what ... but it's an important piece.

I: Mm-hmm (affirmative).

P: So, um, so you listen, uh, listen. I don't know how to put it. Um, I don't want to get preachy.

I: (Laughs)

P: We're all raised to, um ... You know, I got an outline and I put ticks in these boxes, which I don't do. Uh, but you know, you have ... and, and so you ask a question but within two minutes you're drumming your fingers on the table, moving on. But I think people from other cultures, um, go with narrative stuff they want. You ask a story though ... you askin' a question, they'll give you a story. Then you'll be prepared to listen. So, depends how much time I have, I'll hear their story, and not get to what else I need to get to and have them come back, uh, assuming they will.

Or else I'll say that's very important, but I need to get a few more things and we'll, we'll s-, have you come back and we can talk more about it, we'll look at it. Or I will immediately make a referral to a caseworker, or an emergency social worker, um, sitting in the lobby. Um, so we're, kind of, geared for it. So, that's how we would do it.

If I'm not answering your question, you need to let me know.

I: (Laughs) That's great. Um, are there any benefits that you see to asking patients about this?

P: Well, yeah, it's important. If it's real ... if it's there, it's a critically important part of their life.

I: Hm.

P: And if you don't ask, a lot of people won't volunteer it. And then on this, are you hiding it? Some are. Well why shouldn't they not? But, they don't know that's what they're there for. So they don't know why they're there maybe. Because someone said go, you know, the, the internist says well we're settin' you up to see a psychiatrist, with a, what I would say is an inadequate referral. Preparation.

So, people, you, you know, um, they're waiting for you to ask questions. And, um, they wo-, won't necessarily volunteer. So, I, you know, I think it's a critically important part of their life. It, it really, uh ... It's the boulder in their front ... yeah, on the road that they, they, they are facing. Um, and it, it may shape many other things that's happening to them. So, yes, I think so.

Even the question of homelessness, where we, when we ... I think for a while we didn't ask everybody. Uh, I did, but indirectly, but I would say where are you living? But I didn't focus it, and now we're much more aware of. So I would say the criminal, uh, history and homelessness. And these are things that if you, if you can't work on those issues, then there's a lot of other things you can't do because of it. So yes.

I: Mm-hmm (affirmative). And are there any risks or challenges you see to asking patients about this?

P: Hm, physical to me? Usually not. Um, not unless someone is very paranoid. Uh, no, I, I think the risk would be that the person will think you're being too nosy, too inquisitive. Um, or you know something and how do you know it? Well I don't. I'm just going on my fishing expedition, walking down the hall, opening doors. So, I, I think the risk might be if you're timing isn't right, or your way of asking sounds judgmental, or you, the faces you make. Well, ew, that ... um, I, I think those are risks to the, uh, development of a, I'll call, therapeutic relationship, working relationship.

Um, if you show too much interest in something, that is a sensitive and loaded area. So, but I, I think, uh, the risk to not asking is greater. So I think ... yeah I, I think you have to really, uh, you have to ask it as, um, probably, given the situation, your assessment of the person. Well you have to ask sometimes, is there a gun in your pocket? (laughs) Hand keeps goin' in the pocket, or a knife, or something.

Um, I, I think if, if you play it straight people will be okay with it. And I, I don't try to protest, oh, it's okay, you can tell me, I'm safe. Uh, they, they need to figure that out for themselves. Um, so I think that would be the risk. The, the, the, you know, the worst risk in a sense is the person who gets offended and leaves, literally on the spot, or doesn't say so and finishes you, you ... and doesn't come back because of some mistrust.

I try to, uh, process what's going on in the, in the interview, during the interview. So, if I pick up some, you know, like in all the things you say with your face. Eyebrows going up, or just looking back, or whatever, I'll, I'll say it, did that last question bother you? Or did ... is something wrong with that question? Or how are we doing right now? And I'll try to process it right then and there to, to see what happened. And if I, I think I did so insensitively I'll say I'm sorry I put it that way, or whatever else. Here's what I was looking for. And then it usually helps knowledge, that if someone is paranoid enough, you just ... or I've been obnoxious enough, they just, sometimes, can't get over it. Okay.

I: Yeah. Could you tell me a bit more about your overall patient population that you're seeing?

P: I think I've said it all.

I: Mm-hmm (affirmative).

P: Um, yeah, uh, uh, [health care clinic] is a, um ... [health care clinic] it, it, ... Internal workings are not always great as it looks on the outside. Uh, we have our internal conflicts with administration, and they hate it when I say that, but that's it. Um, the population is ... I mean the whole clinic was set up, and I've been there 42 years.

I: Mm-hmm (affirmative).

P: It was, it was a project jointly from the [University Medical School], and [County], and the [local neighborhood] because the university had been criticized of not caring about patients, especially not caring about poor patients, but only being interested in doing their research and getting their grants. And it was a fair criticism. I mean, um ...

So it was put in and, and I think it is pretty much stuck to those principles. Obviously the world has changed and the costs of medi-, the cost of medical care has gone up. On the other hand, there's been a great expansion of medical coverage through Medicare, Medicaid. But the population remains pretty much the same, and [health care clinic], at some extent, even, reflects the demo-, the changing demographics of the [city] [ County] and larger state.

So, um, it reflects the, um, population of, um, Latino, citizens or not, moving up from the south or southwest, uh, in the, I guess, the late 80s. Uh, a large influx of, uh, African-American from [city] and [city], thinking that [new city] would give 'em a better to city to live in and I, I'd like to think so. But, you know, some people thrive and did well.

So it reflected that and it has a Native American population. Um, not, uh, not just ... It somehow never coordinated itself enough with the [Indian Health Board] two blocks away. And when I asked about it, they smiled and they ... Uh, I think it was tribal politics, and they weren't gonna tell me about it, you know.

I: Mm-hmm (affirmative).

P: Um, and then starting in, um, oh '77, '78, um, a huge southeast Asian population and to give you the sense we had, um, by 1980, just in the, the interpreters for mental health alone, we had four Hmong, two Laos, two Vietnamese and two Cambodian. So we, alone, had eight interpreters for the southeast Asian population. And, then, as, as they assimilated, settled in that population, some remain chronic and others ... and moved out. And, um, then starting the civil war in Somalia was '91. So by '94, '95, that population moved in. The Lutheran Church had something to do with it in a positive way. (laughs)

I: (Laughs)

P: I mean going back to southeast Asians, uh, so, making, uh, combining, um, Minnesota's, um, regard for social programming, health programming, and the Lutheran providing housing and stuff. So, uh, you know, uh, I think Minne-, Minnesota and [County], oh, the [cities name], we're already geared for refugee populations in a positive way. So we're probably the primary clinic that people go to. Not all of 'em, ...

I: Mm-hmm (affirmative).

P: ... but I think we see less mid-east people than, maybe, some other clinics. Syria, Iraq refugees, we see some. Um, a [inaudible 00:41:59] new Somali, Ethiopian, and a few Eritrean's are our most ... and some west Africa. So, um, that's the pop- ... And, then, um, poor whites, poor blacks. By poor I mean un-, underfunded. Um, some working, some not. That's the population.

I: Mm-hmm (affirmative).

P: Um, and the diagnoses have changed, partially with what's happening out in the city, so, much more drug addiction, drug abuse. Um, but in general people are schizophrenia, manic depressives, uh, depressions, and alcohol, drug abuse, in combination with schizophrenia and manic depressives. Uh, some brain-damaged people, and if you look you find, um, more brain damage than, than meets the eye. You have to, you know, go up and set up MRIs and electroencephalograms.

Um, we have case managers, too, who work with patients. And we're pretty much set. We are geared to take care of that population, and not as much as I thought. Funding seems to matter in a way that it didn't. Uh, but since we had, in the early 80s, we had a very good community, um, group programs for the southeast Asians, so, we had a, uh, Hmong, uh, cooking group and a Cambodian grief talking group. And, we would have to use buildings outside, because we didn't have room in the clinic.

And then, um, probably, the feds. Uh, god bless ‘em. (laughs) They changed the funding, and they said, no, we're gonna ... everyone in the [inaudible 00:43:52] is a case manager. We're not gonna pay for groups. We want an individual case manager for each individual patient to make sure that they're okay, like they're getting their breakfast in the morning. Now that's important, but they ... like overnight they reverse, let's say, ... Uh, I'm getting a little speechy again.

I: (Laughs)

P: You know, they reversed like 30 years or more of, uh, psychiatric, psychological knowledge about the importance of groups and support groups, and they just done it in. So, um, uh, we, you know, we make in-, make individual visits to people in their board and care homes. But it, it, it's very hard to get in. So it, it got replaced, you know, that there's support groups now. But, uh, yeah, that was dead for about ten, 15 years. So those things have changed somewhat, not for the better. So we try and get people involved in groups and visit more outreach in, as the community develops more things. So that's the population.

I: Mm-hmm (affirmative).

P: They have a lot of medical problems, and it's nice to have, um, medicine and the psychiatry under the same roof. You know, like, uh, here. If someone needs to see someone, I have to ... they either… across the street. I think there's a, a, um, kind of, refugee, uh, clinic across the street. But it's across and, you know, we'll take 'em there because the acting, that's the person that will do it. But if you just tell someone to go, or to get a lab tested, most people just won't show up.

I: Mm-hmm (affirmative).

P: Whatever. Or you don't have control of the funding [inaudible 00:45:29].

I: Yeah.

P: Okay.

I: And, so, in terms of your patients that have some type of justice system involvement, um, are they ever specifically referred to your care?

P: Um, yeah I think so.

I: Mm-hmm (affirmative).

P: We also offer it sometimes, uh, a referral system. But we'll, we'll get some referrals in from churches, from lawyers, that so and so, uh ... But the care, would, would be, primarily, um ... someone would see that there's ... uh, someone in the criminal justice system who looks disturbed enough that the lay person notices it and says, you know, this isn't right. This, this person, uh, is mentally ill and should be getting psychiatric care. And they're gonna re-offend, or they'll ... uh, and I've seen this, even when it worked at low-level, or you, you go in for the sentence of five years and you come out with ten years because you socked the guard, you know, because you were disturbed. And so if you can get to people first before they, they do that, then you save them much trouble, et cetera.

So we get some referrals. Um, frankly we had a [inaudible 00:46:41], I think about it. Didn't work well. Um, it was probably a bad ... We, we, we agreed to have, um, one of the work houses, or jails send their prisoners to us that they thought needed help. And, um, maybe predictably, maybe not, um, they all came in saying, uh, I need val-, I need Valium, I need Librium, I need amphetamines. (laughs) I don't give them to anyone, reason nothing doing. That's ... no, and we got threatened and very unpleasant situation. So I, I think it got seen as, um, a place to continue your, your, your street meds, um, and, and so. And after too many threats, we just canceled that program.

Um, you know, we don't have, we don't have security at the clinic, other than a, a panic button, ...

I: Mm-hmm (affirmative).

P: ... and an overhead, just a balloon type of thing. So, um, so there was an example where, um, uh, uh, I think we didn't, we could have done better than the jail. The authorities, or whoever set it up, could've done better. They didn't think about who they're sending and how it gets used. Uh, when we, uh, instituted a walk-in policy, maybe ten years ago, and nothing to do with jails. But, again, what we got was a population of drug users. Hey, you know, they're givin' out prescriptions down at [health care clinic]. Um, and, then, you know, that's not what we were doing. And, and, so, I, I think that when these program get set up, more thought has go ... preparation at, at, at the staff levels, but also at, at, at what you're telling people the clinic does and ...

I: Mm-hmm (affirmative).

P: ... why you want them to go there. It's not blamin', I'm just ... it didn't work.

I: 'Kay. And in your role do you ever communicate with probation, parole, or the courts at all?

P: Oh sure.

I: Mm-hmm (affirmative).

P: Well you com- ... Communicate with the court is something else. You gotta stay away from the judge. I mean the judge will see you, so tryin' to influence. I got yelled at once. Um, you know, I made a recommendation that someone be allowed to stay in the country.

I: Mm-hmm (affirmative).

P: The judge said, why did you do that? And I said, well, that was my recommendation. But now I can't trust your objectivity. I said, well, um, I'm gonna have to go practitioner. I, I ... what I think is correct. I didn't mean to take your prerogative, but it didn't ... went off on a bad foot.

Um, so, yes, we, we get calls. Well, we'll, we'll, we'll ... Uh, if I have someone who is in ... coming up to trial, or got a hearing or something, I'll, I'll ... and I, and I understand. I'll say to the person, would you like me to contact your lawyer? And if they have the name then I'll just send an email, or a call, saying, um, seeing so and so. Um, and if you're, if you're interested in collaboration, give us a call.

I: Mm-hmm (affirmative).

P: And usually they are. Um, so, I think we do, we were hopin' to do a fair amount of that. Um, I don't know if ... I think we may have had someone go down to some of the, um, jails or something, uh, to do some education stuff. I'm not positive. I know we're involved with the, um, homeless encampment that's across the street from us. You know, it's some people who have that as an interest doing more than others. We set up some medical people there. And, okay that ... you know, does that cover it?

I: Yeah. Mm-hmm (affirmative).

P: I, I'm okay with time. The ...

I: Okay.

P: If we, if we want to ...

I: I have a few more questions ...

P: Yeah.

I: ... to ask.

P: Uh, they'll, they'll, they'll ... we have a meeting now,

I: Okay.

P: ... but they just cut ...

I: Feel free to cut me off whenever.

P: No, no.

I: Yeah.

P: They discuss my patients, but they also discuss, uh, [other provider’s] patients. So they'll just begin with his.

I: Okay. All right. And, then, aside from possible justice system involvement, what else are you seeing your justice-involved patients dealing with socially?

P: Oh, I know, it's a ma- ... all the things that ... Again, we're, if we're talkin' about refugees. But even not. People ...

I: Mm-hmm (affirmative).

P: ... out of prison. They can't get housing. Um, landlords won't take them. No, I think there's a, um, one of the non-benefits of computers. Here is you can look up someone's stuff right away if you know where to look. And, so, people have trouble getting housing, getting jobs. Um, those would, probably, be the two major things.

I: Mm-hmm (affirmative).

P: Um, and in its own little way, someone who dropped out of college, or because say they were depressed, but they had a student loan, then they dropped out of college or, or whatever else they ... They can't get the loan again, they can't get back in. And, you know, one has to figure out what's going on as best one can. It's not that they bought a car with the student loan, um, and, so, we'll help them out at that level.

So it, it's, it's not criminal justice but, uh, it has to do with helping someone out from the, the economic problems. So, yeah, I, I think there's enormous fall-out, ...

I: Mm-hmm (affirmative).

P: ... of, um, legal difficulties that play, play across, um, what else one wants to do, in-, including, y-, you know, a treatment program. So, uh, if someone doesn't make child payments, um, I'm not, I'm not taking that person's side. But if they take his car away, then he can't go to work. And this has always struck me as just incredible. Uh, you know, that does happen.

I: Mm-hmm (affirmative).

P: So, then the person's saying, well how can I go to work, blah, blah, blah, and, of course he's right, but he's wrong. But, so, you know, we'll see if we can help out with a letter or something.

Um, it may sound bad. I try to avoid goin' to court, uh, just because, uh, I can't ... I'll say I can't ... I don't wish to let myself be vulnerable to their scheduling system. So they can say well can you show up at one o'clock? The hearing begins and you show up at one o'clock, and you're waiting there until three o'clock, we only had to cancel four patients. And then the judge says, well, we're gonna continue this for the next month. And I said, hm, I know it's not about me, I'm not the key player. But that, you know, I'll spend my whole week, uh, sitting around courts. Well I try to avoid that, and make do with letters, or telephone. Um, [inaudible 00:53:38] testimony ...

I: Mm-hmm (affirmative).

P: ... rarely will I go to court. And usually it's for something like deportation. It's, uh, it's all or nothing. Uh, occasionally for citizenship, things like that. And I try to, uh, always know in advance, uh, what my limits are. But we try to do a lot. That's about it.

I: And, then, what are your justice involved patients dealing with medically?

P: Oh, well diabetes is equal opportunity offender, and the poor people, probably, have a higher rate of diabetes because of, uh, a, dietary problems and, you know, uh, fast foods, sugars, and stuff like that. And the, the, the avoidance of health systems, or not being, not getting in the health system, right diagnosis, so it's underdiagnosed. Illnesses are more complicated. Chronic obstructive pulmonary disease, smoking, drug use, diabetes. Poor teeth. Uh, we have a very busy clinic run by the dental school. Um, and poor teeth is really a major problem. Hey, uh, if you can't afford whatever else, you certainly can't afford dentists on the outside.

So they have the same problems, but more so. Um, sometimes, um, sexually transmitted diseases or damage from being assaulted. Uh, but I would think that the more, just, that, the same diseases that we're all prone to, but, um, they don't get the care. So ... and, um, they might get the care in jail. I mean they don't get the mental health care, but they might get someone making rounds.

I: Hm.

P: And, and ... and there'll be a psychiatrist in jail too and ...

I: Let's see. And are there any resources or services that your patients need, but you found that aren't available to them?

P: Oh we, we try to be pretty resourceful, so I don't know. Here, here are what we work with, um, legal aid and several other things. Um, yeah, there's sometimes drug treatment programs, uh, which might have to do with funding. But it is ... it has to do more with funding than other things. Um, I, I don't think so. I, I think, um ... there's, there's very good social workers, case workers we saw. So, care coordinators who, who try very hard to, uh, get someone connected to some community agency that they would need. And we try to offer help to families too.

I: And now I'm thinking broadly, are there any changes to how we deliver health care, that you would suggest to better meet the needs of folks that are criminal justice involved?

P: Oh, I'm gonna shoot my wise-cracker. Fire half the administrators. Um, but not criminal inve-, health care. You need more people seeing patients and less administrators. But that's not special.

I: Mm-hmm (affirmative).

P: Um, and ask the questions again, because I short-circuited it.

I: Yeah. So thinking broadly, are there any changes to how we deliver health care that you would suggest to better meet the needs of folks that are criminal justice involved?

P: Well it, it, it would ... Yeah, well, I don’t even how to begin it. I ... having, um, people, um, located within a jail. You know, like, a half-time psychiatrist or, you know, two internists, or whatever. Um, women's doctors, gynecologists, stuff like that. I, I think accessibility, so you don't have to put someone in handcuffs and put 'em in an ambulance, or, or a paddy wagon to go down to [County]. So I think bringing medicine to the jails would be better than ...

It may be very impracticable, but it, there should be the business there, you know, uh, three clinics a week type of thing. Um, it might be a good idea going back to your, one of your earlier questions to have students rotate through, residents rotate through. But again, you know, you'd deal with curriculum that is in no less demand, demands for time to do. Um, those are, kind of, uh, common sense things.

Um, having good working arrangements with some jails might help, like we tried to do, but, but not successful. That would do, um, whether hiring, um, ex- ... I don't know what the nice word, the ex-felons, ex-criminals, um, that, yeah, is, people in drug treatment are often, you know, recovered from that. I, I, think, uh, peer counseling at the ... in, in ... we have a peer counsel now on the ACT team who [inaudible 00:59:00] bipolar illness, and, and does very well with patients. So, that might be good.

Um, possibly work with the, uh, public defender's office. But, uh, (laughs) it’s not like anyone’s sitting around looking for more work. Um, that's why I, I, I thought to meet ...

I: Mm-hmm (affirmative).

P: ... you here, because to meet you Tuesday or Wednesday at [health care clinin], well on the days I'm there, I ...

I: Mm-hmm (affirmative).

P: ... couldn't, couldn't count on it. Like I could say come at noon, but I might have two walk-ins right then. So, anyway, um, [inaudible 59:45] awareness, uh, is an important uh, under, underserved population.

I: So, uh, thank you again for your time today. Before we wrap up, is there anything that I didn't ask that you'd like to add?

P: No, I think I did along the way.

I: (Laughs) Okay, so I'm gonna start prepping the survey that we have. Um, to gather, demographics. Um, but are there, are there, do you have any other providers in mind that we should interview? We definitely ...

P: I'll just email you a list.

I: Okay.

P: Don't tell. Yeah, I mean uh, my, my colleague I ... I, I'll just go through a list and give you, um ... how many do you want, kind of?

I: Uh, as many as you, as you have that come to mind.

P: Alright, sure. Let me just email you.

I: Okay. That sounds great. And when we have any reports and findings from this study, would you be interested in receiving those?

P: Sure. Yes.

I: Okay.
