# Supplementary material for: Physicians’ perspectives on continuity of care for patients involved in the criminal justice system: A qualitative study
Source: PLoS One. 2021 Jul 14;16(7):e0254578. doi: 10.1371/journal.pone.0254578 (PMC8279398; doi:10.1371/journal.pone.0254578)
Supplement: Attachment — Submitted filename: PLOS ONE - Physicians Perspectives_ Re-Review.docx [file pone.0254578.s003.docx]

PLOS ONE-Physician’s Perspectives Re-Review

GENERAL: Several concerns of the reviewers were addressed

METHODS

1. For validity, at what point did Continuity of Care emerge as a theme? Was it one of your guiding questions that drove this process or did it emerge in the interviews,?
2. It would be helpful to clarify that among the many themes found in the interviews, you chose Continuity of Care as the theme of focus for this paper, in Methods section, and then that you subdivided this as you already described.
3. The added information on recruitment is clear and informative.
4. To help in understanding your analysis methods, in addition to citing the references, suggest summarizing the method in 2-3 sentences.

RESULTS

1. We note that the authors chose not to list the numbers of a particular subtheme which were found in the data. While the qualitative approach is not based upon numbers, it can be helpful in the interpretation of the data. We leave this to the editors to consider in importance.

DISCUSSION

1. Improved organization.
2. Generally, the authors seem to be drawing more definitive conclusions than the data warrant, though the data are compelling.
3. The discussion should: summarize the results, contextualize results in light of prior data, indicate where future work is needed, and carefully describe implications that are rooted in the data.
4. In that light, consider moving some portions of discussion (the last two paragraphs-- “in order to improve health outcomes and reduce recidivism…” and “Medicaid and managed care organizations…”) to the implications section.
5. And consider less definitive statements of “should;” these statements can be moved to the implications section as well.
6. This is particularly important for policy change recommendations which could perhaps be described as worthy of examination and to then study outcomes.

LIMITATIONS

1. The authors rightly and importantly point out that rural physicians are inadequately described in this area. It could be stated as a limitation in this study and/or point in the direction for future research.
